# Contribution to the Orophilous Cushion-Like Vegetation of Central-Southern and Insular Greece

**DOI:** 10.3390/plants9121678

**Published:** 2020-11-30

**Authors:** Carmelo Maria Musarella, Salvatore Brullo, Gianpietro Giusso del Galdo

**Affiliations:** 1 Department of AGRARIA, Mediterranean University of Reggio Calabria, 89122 Reggio Calabria, Italy; 2 Department of Biological, Geological and Environmental Sciences, University of Catania, 95125 Catania, Italy; salvo.brullo@gmail.com (S.B.); g.giusso@unict.it (G.G.d.G.)

**Keywords:** phytosociology, flora, high mountain vegetation, Greece, taxonomy, *Astragalus*, *Allium*

## Abstract

The results of a phytosociological investigation regarding the orophilous cushion-like vegetation occurring in the top of the high mountains of central-southern Greece and in some Ionian (Lefkas, Cephalonia) and Aegean Islands (Euboea, Samos, Lesvos, Chios and Thassos) are provided. Based on 680 phytosociological relevès (460 unpublished and 220 from literature), a new syntaxonomical arrangement is proposed with the description of a new class, including two new orders, eight new alliances, and several associations (many of them new). Compared to the previous hierarchical framework usually followed in the literature, this study provides a more realistic and clear phytosociological characterization of this peculiar and archaic vegetation type, which is exclusive to the high mountains of the north-eastern Mediterranean. The new arrangement is mainly based on the phytogeographical role of the orophytes featuring this very specialized vegetation, which is essentially represented by endemics or rare species belonging to the ancient Mediterranean Tertiary flora. In addition, taxonomic research on the orophilous flora occurring in these plant communities allowed to identify six species new to science (i.e., *Astragalus corinthiacus*, *Allium cremnophilum*, *A. cylleneum*, *A. orosamium*, *A. karvounis,* and *A. lefkadensis*) and a new subspecies (i.e., *Allium hirtovaginatum* subsp. *samium*), and two new combinations (i.e., *Astragalus rumelicus* subsp. *euboicus* and subsp. *taygeticus*) are proposed.

## 1. Introduction

The orophilous cushion-like vegetation colonizing the cacuminal stands of the highest mountains of the Mediterranean territories has always aroused a lot of interest from botanists, mainly for the occurrence of a peculiar and specialized flora. It is represented usually by relict taxa (species and subspecies), mainly endemic adapted to hard environmental conditions, which are aggregated in physiognomically well differentiated plant communities [1,2].

Many plants that characterize these phytocoenoses (usually localized at high altitude) belong to the ancient Tertiary Mediterranean flora. They are represented mostly by dwarf nanophanerophytes and chamaephytes mixed with caespitose hemicryptophytes which form plant communities often covering large surfaces. This ecologically specialized vegetation is associated and adapted to long wintry periods of lasting snow cover (sometimes till late spring), as well as to prolonged summer droughts with intense winds. It occurs mostly on rocky places with undeveloped and immature soils due to the prevailing harsh climatic conditions and wide diurnal and annual variations. Such factors seem to converge to climatic conditions of the temperate cold climate inserted in the Mediterranean context [3].

This leads to a strong contrast between the winter-spring period, which is very rigid and cold, and that summer-autumn one, generally very hot and dry. Therefore, the plants that thrive in these high-mountain stands (generally ranging from about 1000 to 4000 m of elevation in Mediterranean area are evolutionarily adapted to very peculiar climatic conditions shaping significant ecological specializations and concomitant range restrictions, that, in most cases, not allows them to live outside of these habitats. Therefore, they can be considered as typical Mediterranean orophytes that often have well circumscribed or punctiform distribution patterns and are well differentiated taxonomically from other closely related taxa. Among these high-mountain plants, it is possible to identify vicariants due to speciation processes and geographical or ecological isolation.

The occurrence of dwarf shrubs in these Mediterranean mountains is very significant; such species have a typical thorny and compact cushion-like habit, inside which many delicate herbaceous species take refuge due to higher humidity compared to the exterior one, with their vegetative and floral structures protruding from them. Among these, an important physiognomic-structural role is played mainly by thorny species of *Astragalus*, which often dominate in many plant communities.

In the central and western Mediterranean few of species belonging to this group of *Astragalus* usually occur. They are mainly represented by endemic species disjunctly distributed among some mountain tops, for example: *Astragalus granatensis* Lam. in Spain and northern Africa, *A. nevadensis* Boiss. in southern Spain, *A. genargenteus* Moris and *A. gennarii* Bacchetta & Brullo in Sardinia, *A. greuteri* Bacchetta & Brullo in Corsica, *A. calabricus* Fischer in Calabria, *A. sirinicus* Ten. in southern Apennines, while in Sicily there are *A. siculus* Biv. in the Etna volcano and *A. nebrodensis* Guss. in the Madonie massif [4]. Conversely, *Astragalus* species with tragacanthoid habit are much more numerous and widespread in the mountains of the eastern Mediterranean territories, which reach their maximum diversity in Anatolia. In particular, in the mountains of the Balkan Peninsula and of some Aegean islands the species of thorny *Astragalus* are more frequent, such as: *A. angustifolius* Lam. s.l., *A. rumelicus* Bunge s.l., *A. creticus* Lam., *A. cylleneus* Boiss. & Heldr., *Astragalus calavrytensis* Beauverd & Topali, *A. cephalonicus* C. Presl, *A. thymphresteus* Boiss. & Spruner, *A. parnassi* Boiss., *A. taygeteus* Persson & Strid, *A. thracicus* Griseb., *A. condensatus* Ledeb., *Astragalus lesbiacus* P. Candargy, *A. dolinicolus* Brullo & Giusso, etc. [5].

Apart from the *Astragalus* sp.pl., there are also other orophytes belonging to other genera or species complex that are characterised by morphological peculiarities and ecological adaptations, which are well distinct from other ones closely related taxa found in coastal or hilly places (geographical or ecological vicariants). In the eastern Mediterranean area, the following groups of taxa can be quoted as examples:

(a) *Cerastium candidissimum* Correns, replaced in the Apennines and Sicily by *C. tomentosum* L., in Sardinia by *C. supramontanum* Arrigoni and in Corsica by *C. soleirolii* Duby [6,7,8].
(b) *Marrubium cylleneum* Boiss. & Heldr., distributed in the northern Peloponnese, which is vicaried by *M. velutinum* Sibth. & Sm. in the central Greece and *M. thessalum* Boiss. & Heldr. in the northern Balkans [9].
(c) *Sideritis clandestina* (Bory & Chaub.) Hayek is represented by the subsp. *clandestina* in the southern Peloponnese, and by the subsp. *peloponnesiaca* (Boiss. & Heldr.) Baden in northern Peloponnese, while it is vicariated by *S. raeseri* Boiss. & Heldr. subsp. *raeseri* in central and northern Greece, *S. euboea* Heldr. on the island of Evvoia, *S. scardica* Griseb. in the north and central Greece and former Yugoslavia, *S. sipylea* Boiss. in the eastern Aegean area, *S. syriaca* L. subsp. *syriaca* in Crete, *S. sicula* Ucrìa in Sicily and *S. italica* (Miller) Greuter & Burdet in the central-southern Italy [10,11].
(d) *Nepeta argolica* Bory ex Chaub. is distributed in the Peloponnese and Sterea Ellas, replaced by *N. dirphya* (Boiss.) Heldr. in Euboea, *N. parnassica* Heldr. & Sartr. ex Boiss. in the Mts. Parnassus and Chelmos, *N. spruneri* Boiss. in the North-central Greece, *N. camphorata* Boiss. & Heldr. in Taygetos, *N. sphaciotica* Davis in Crete, *N. orphanidea* Boiss. in Mt. Parnon and *N. italica* L. in Samos and West Anatolia [12].
(e) *Carlina frigida* Boiss. & Heldr. widespread in central-southern Greece, which is replaced by *C. biebersteinii* Hornem. subsp. *brevibracteata* (Andrae) K. Werner in the northern Balkans, *C. curetum* Heldr. in Crete, *C. macrocephala* Moris in Sardinia and Corsica, *C. nebrodensis* Guss. in Sicily and South Italy [13,14].
(f) *Sesleria vaginalis* Boiss. & Orph. widespread in Greece, replaced by *S. robusta* Schott et al. in the northern Balkans, *S. anatolica* Deyl in Samos and Anatolia, *S. achtarovii* Deyl in Thassos and eastern Balkans, *S. nitida* Ten. *s.l.* in Sicily and central-southern Apennines [13,15].
(g) *Erysimum pusillum* Bory & Chaub., endemic to southern Peloponnese, replaced by *E. cephalonicum* Polatsc. in northern Peloponnese and central Greece, *E. parnassi* (Boiss. & Heldr.) Hausskn. in the Parnassus, *E. olympicum* Boiss. in the Mount Olympus (Greece), *E. mutabile* Boiss. & Heldr. and *E. raulinii* Boiss. in Crete, *E. bonannianum* C.Presl and *E. etnense* Jord. in Sicily [13,16].
(h) *Viola graeca* (W. Becker) Halácsy, widespread in central Greece and northern Peloponnese, replaced in the southern Peloponnese by *V. parnonia* Kit Tan & al. in Mt. Parnon, *V. sfikasiana* Erben on Mt. Taygetos, *V. euboea* (Halácsy) Halácsy in Euboea, *V. epirotica* (Halácsy) Raus in Pindos ranges, *Viola stojanowii* W. Becker in Sterea Ellas, *V. fragrans* Sieber in Crete, *V. nebrodensis* C. Presl and *V. aethnensis* (Ging. & DC.) Strobl in Sicily, *V. corsica* Nyman in Corsica and Sardinia [13,17].
(i) *Armeria orphanidis* Boiss., distributed in southern Greece, which is vicariate in other Mediterranean mountains by *A. nebrodensis* (Guss.) Boiss. on Madonie in Sicily, *A. aspromontana* Brullo, Scelsi & Spamp. in Aspromomte (southern Calabria), *A. brutia* Brullo, Gangale & Uzunov in Sila (northern Calabria), by *A. sardoa* Spreng. in Sardinia and *A. multiceps* Wallr. in Corsica [18,19].

From the dynamic point of view, these plant communities reach full expression and maturity in the cacuminal stands above 1700–1800 m of altitude, where they usually play a climatophilous role, replacing the forest vegetation that generally stops below the aforesaid altitude, usually corresponding to the timberline.

Examples of this type of vegetation are also frequent between 900 and 1700 m of altitude, in correspondence of summit areas or ridges of mountains in which there are very harsh conditions, due to several environmental factors. In these peculiar environmental conditions, these communities will not constitute typical climatophilous associations, but assume an edaphophilous role or sometimes secondary one due to the processes of degradation of the woodlands.

This type of vegetation, occurring in the several mountain ranges of the Mediterranean area, was studied previously by several authors, using the phytosociological sigmatist approach. In particular, object of these researches regarded the pulvinar orophilous vegetation of several massifs, as: Sierra Nevada in southern Spain [20], Atlas Mountains in North Africa [21], as well as many high mountains of Corsica [22,23], Sardinia [2,24,25], Sicily [2,26,27,28,29,30,31], Calabria in southern Italy [32,33,34], Greece [35,36,37,38,39,40,41,42,43,44], Crete [45,46], Anatolia [47,48,49,50], and Cyprus [51].

In order to improve the knowledge of this type of orophilous vegetation, a study on main mountain massifs of Central and Southern Greece (Sterea Ellas and Peloponnese) was carried out. These phytosociological investigations were also extended to the mountains of some islands of Ionian area (Lefkada and Cephalonia) and of eastern and northern Aegean, such as Euboea, Samos, Lesvos, Chios and Thassos, where there are mountains colonized in the top by pulvinar orophilous plant communities. The aim of this paper was to investigate the orophilous pulvinar vegetation of this area of the eastern Mediterranean, since this region still lacks a detailed approach from the phytosociological viewpoint and there is a need to fully clarify the issues regarding their syntaxonomical arrangement and related nomenclatural aspects.

However, it has been noted that in some cases these plant communities can make catenal contacts with orophilous conifer forests having often a prostrate or pulvinate habit, which can be considered as the last vestiges of the ancient dwarf forests, that at the end of Miocene covered in a massive and widespread way the peaks of the high Mediterranean mountains [52,53,54,55,56,57]. It is possible to observe in this vegetation the dominance of conifers (mostly big shrubs or small trees belonging to the genus *Juniperus, Pinus* and *Abies* that are usually associated to several other orophilous, often thorny, shrubs), among which there are various species of the genera *Berberis*, *Cerasus*, *Ribes*, *Rosa*, *Sorbus*, *Daphne*, *Rhamnus*, etc. These relict forests belonging to the class *Pino-Juniperetea sylvestris* Rivas-Martínez 1965 are widespread in all the Mediterranean area and, in particular, in the eastern territories, they are represented by the order *Berberido creticae-Juniperetalia excelsae* Mucina in Mucina et al. 2016, with several alliances and associations described by Brullo et al. [57].

### 1.1. Study Area

**Figure 1:**
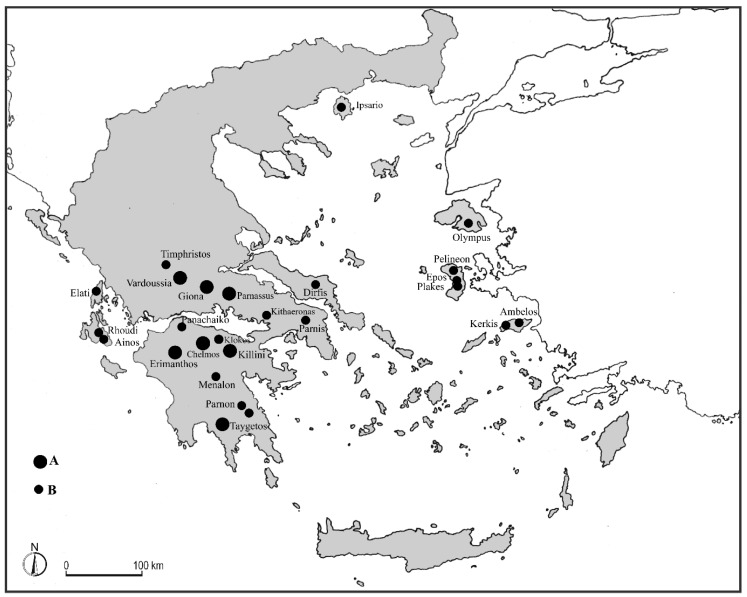
Distribution map of the massifs (**A**) and mountains (**B**) investigated in Greece.

The mountain ranges and massifs, that have been investigated in this study, are located in Southern Sterea Ellas and Attica (Parnassus, Giona, Vardoussia, Timfristos, Parnis (or Parnitha) and Kitheronas (or Kithaeronas)), in Peloponnese (Panachaiko, Erimanthos, Klokos, Chelmos, Killini, Menalon, Taygetos, and Parnon) and in some Islands of the Ionian (Lefkas and Cephalonia) and Aegean area (Euboea, Thassos, Lesvos, Samos, and Chios) (Figure 1).

#### 1.1.1. Sterea Ellas and Attica

From the geographical point of view, the high mountains of central Greece distributed to the north of Gulf of Corinth, falling in Sterea Ellas and Attica, are represented by the three main mountain massifs: Parnassus, Giona and Vardoussia, all characterized by peaks with altitudes above 2000 m, as well as other lower mountains like Mt. Kitheronas and Mt. Parnis.

- Mt. Parnassus

This mountain range is spread from north to south for about 25 km and has numerous peaks, many of which exceed 2100 m: Liákoura (2455 m), Kotróni (2428 m), Tsarkos (2415 m), Gérontovrakhos (2395 m), Koukos (2234 m), Mávra Lithári (2334 m), Raïdhólakka (2328 m), énneza (2328 m), Kalogiros (2327 m), Tsarkoraki (2322 m), Arnóvrissi (2259 m), and Sési (2120 m). The substrates are essentially constituted by carbonate rocks, consisting mostly of Mesozoic dolomites and limestones. The landscape is mainly rocky with walls and dolines. The bioclimate between 1000 and 1500 m falls within meso-Mediterranean sub-humid, while up to 2000 m is of supra-temperate sub-Mediterranean type; over 2000 m it is replaced by oro-temperate sub-Mediterranean one. The average annual temperatures range between 11 °C and 5 °C, related to the altitude, while the annual rainfall average ranges from 800 to 1000 mm. Up to about 1700 m scattered forests dominated by *Abies cephalonica* occur, which are usually mixed to dwarf shrubs conipher communities and cushion-like orophilous vegetation: the latter, becomes dominant above 1700 m of altitude. Previously, these shrub communities were investigated by Quézel [35].

- Mt. Giona

It is a large mountain massif with rather blunt peaks spread over a large area. The highest peak is Piramídha (2507 m), followed at lower altitudes by Traghonoros (2456 m), Makrivlakos (2302 m), Plativoúna (2316 m), Profitis Ilias (2298 m), Pirgákia (2191 m), Vraïla (2177 m), Kastro (2176 m), Stállos (2128 m), and Plativouni (2122 m). The substrates are mostly constituted by Mesozoic dolomites with numerous plateaux and valleys and scattered dolines. In this area the bioclimate has the same characteristics as that shown for the Mount Parnassus: in particular, the annual average temperatures range between 10 °C and 5 °C, while the mean annual rainfall is between 800 and 1000 mm. According to Quézel [35], the timberline of *Abies cephalonica* forests is around 1700 m, while above this altitude, the landscape is dominated by cushion-like shrubs, often mixed to grasslands.

- Mt. Vardoussia

This massif is long 45 km, with various peaks oriented from north to south, separated into two blocks by the valley of Kanavorema river. The highest peak is Korakas (2495 m) which is followed by Kókkini Tsoúma (2414 m), Skórda Ptimalikoú (2413 m), Klisoura (2403 m), Kokiniás (2383 m), Vouno Chomirianis (2293 m), Korakia (2148 m), and Sinani (2054 m).

The substrates consist mainly of Mesozoic limestones. The landscape is characterized by rocky cliffs interspersed to ridges with rocky slopes. Screes and snowy valleys are also frequent. The bioclimate below 1700 m ranges from meso-to supra-temperate in the sub-Mediterranean variant, while at higher altitudes it falls in the supra- and oro-temperate belt.

The annual average temperatures between 1000 and 1700 m of altitude range from 11 to 8 °C, reaching values of 5–4 °C above 1700 m. The annual rainfalls range from 800 to 1000 mm in relation to altitude. As highlighted by Quézel [38], the forests between 1600 and 1800 m are represented by woodlands of *Abies cephalonica*, while at higher altitudes the communities of cushion-like shrubs are dominant. Between 1800 and 2000 m, this vegetation is associated to *Juniperus foetidissima* with scattered individuals.

- Mt. Timfristos

This mountain is located at the northernmost of Sterea Ellas, at the northern side of Mt. Vardoussia. The highest peak is Veluchi (2315 m), which is followed by Symbetheriako (2104 m), Anemos (1998 m), Kumbi (1863 m), etc. Geologically, Mt. Timfristos is represented by Mesozoic limestones mixed with schits and cherts (Mountrakis 1985). As concerns its bioclimatology, it shows the same characteristcs mentioned for Mt. Vardoussia.

- Mt. Kitheronas

It is an isolated mountain located west of Mt. Parnitha, reaching in the Profitis Ilias the altitude of 1409 m. It consists of Mesozoic limestones with very steep slopes and a small plateau at the top where an orophilous shrub vegetation is located.

- Mt. Parnis

This massif located north-west of Athens, also known as Mt. Parnitha, is characterized by several peaks: Karavola (1413 m), Ornio (1350 m), Mavrovouni (1091), etc. The bioclimate of this area falls within the meso-Mediterranean subhumid belt, with an annual average precipitation about 790 mm. Geologically it consists of Mesozoic limestones and schists. Examples of orophilous dwarf-shrub vegetation are usually frequent at an altitude above 1100 m. Some observations on the orophilous vegetation are reported by Aplada et al. [58].

#### 1.1.2. Peloponnese

The Peloponnese is the southernmost part of mainland Greece, separated from the mainland by the Gulf of Corinth which in the past was united by the homonymous Isthmus (now Channel). This peninsula has a surface of 21,400 km^2^ that, apart from few coastal plains, is almost entirely mountainous. The main massifs are located in the northern part of Achaia, along the coastal strip corresponding to the southern side of the Corinth Gulf, among them there are Mt. Panachaiko, Mt. Erimanthos, Mt. Klokos, Mt. Chelmos, Mt Killini, and Mt. Menalon, some with peaks higher than 2000 m. Other mountain ranges occur in the southern part of the Peloponnese: among them the most important are Mt. Taygetos in Arkadia and Mt. Parnon in Lakonia. In particular, on Taygetos the altitude of 2400 m is reached.

- Mt. Panachaiko

Panachaiko is the mountain range with the northernmost position than any other mountain in the Peloponnese. It is located south of Patras, peaking almost 2000 m of elevation. Its summit reaches, in fact, 1924 m and is characterized by ridges with rocky walls overhanging extended screes. The substrates consisting of dolomites and limestones dating back to the Mesozoic, usually with very sloped and rocky surfaces. From a bioclimatic point of view, this mountain area falls mainly within the meso-Mediterranean belt, and only in the highest part, above 1600–1700 m, it tends toward the supra-temperate sub-Mediterranean one. The ombrotypes range from the sub-humid to humid, with average annual rainfall reaching 1000 mm. The annual average temperatures range between 8 °C and 10 °C. Due to the remakable acclivity of the surfaces, the orophilous cushion-like communities are quite widespread and well represented from the 1500–1600 m of altitude. Currently, there are no phytosociological data about this type of vegetation on this mountain.

- Mt. Erimanthos

It is located in the northwestern part of the Peloponnese, south of Panachaiko, and forms a range oriented from NE to SW with various rocky peaks, including Mt. Granitis (2221 m), Mt. Barba (2169 m) and Mt. Profitis Ilias (2124 m). Other peaks forming part of this massif range are: Mt. Pirgako (2050 m), Mt. Kallifoni (1996 m), Mt. Lepida (1893 m), Mt. Psili Tourla (1891 m), Mt. Gnaikes Tris (1834 m) and Mt. Lambia (1793 m). The substrates are usually represented by limestones, radiolarites and, sometimes, scists. The bioclimate, between 1600 m and 1900 m of altitude, falls within the supra-temperate sub-Mediterranean belt, while above 1900–2000 m in the oro-temperate sub-Mediterranean one. At altitudes below 1600 m, the surfaces are affected by a meso-Mediterranean termotype. As regards the ombrotype, it ranges from the lower to the upper humid, with average annual rainfall of 1000–1400 mm. The annual averages temperatures are around 8–5 °C, with significantly lower values on the eastern side which is characterized by a higher continentality. The tree vegetation is represented by woodlands of *Abies cephalonica*, that are widespread up to 1600–1700 m, while at higher altitudes, not exceeding 1800–1900 m, there are examples of open and spaced dwarf woods of *Juniperus foetidissima*, usually mixed with cushion-like communities, that in the higher peaks become dominant. Previously, a study of this hemicrypto-chamaephytic orophylous vegetation, was carried out by Maroulis and Georgiadis [44].

- Mt. Klokos

This mountain is located at south-east of M. Panachaiko, characterized by carbonatic substrates consisting mainly of dolomites. The summit reaches 1778 m in altitude and coincides with the uppermost part of a large rocky face. The landscape is very rough due to the presence of ridges, very steep slopes, and screes. This mountainous area is characterized by a bioclimate falling mostly in sub-humid meso-Mediterranean belt which, at the summit, tends toward the supra-temperate sub-Mediterranean one. Average annual temperatures range between 8 °C and 9 °C, while the average annual rainfall reaches 900–1000 mm. The highest part of the mount is essentially characterized by thorny pulvinate communities, covering the most part of the surfaces. So far, there are no studies on the vegetation of this mountain.

- M. Chelmos

Mt. Chelmos, also called “Aroania”, is one of the main massifs of northern Peloponnese, with several peaks topping 2000 m in altitude, such as Psili Korfi (2355 m), Neraïdorachi (2339 m), Kato Kambos (2318 m), Profitis Ilias (2282 m), Ghardhiki (2182 m) and Augo Anghio (2138 m). The substrates consist mainly of Mesozoic limestones and dolomites, sometimes with outcrops of marls and clays. The landscape is very harsh and rugged with numerous ridges, very steep slopes, screes and valleys. At elevations higher than 1500–1600 m, the bioclimate falls into supra-temperate sub-Mediterranean belt, while above 1800–1900 m of altitude it is of oro-temperate sub-Mediterranean type, with average annual temperatures between 9 °C and 5 °C. The ombrotype is comprised between the upper sub-humid and the lower humid, with annual average rainfall reaching 900–1200 mm. In the mountain belt, at an altitude lower than 1500 m, the bioclimate is attributable to meso-Mediterranean sub-humid. The forest vegetation is represented by *Abies cephalonica* woodlands or, limitedly to marly substrata, by pine wood of *Pinus pallasiana*. In the higher stands, coinciding with the peaks and the steep rocky slopes, the surfaces are covered by orophilous pulvinate communities. Investigations on this vegetation, were previously carried out by Quézel and Katrabassa [40].

- Mt. Killini

Mt. Killini, also known as “Ziria”, is a mountain range with several peaks topping 2000 m in altitude, including Megali Ziria or Simio (2374 m), Profitis Ilias (2259 m), Kokinovrakos (2168 m), Michri Ziria or Kioni (2082 m), Paraga (2032 m), and Tsouma (2021 m). It is located in the north-eastern sector of the Peloponnese, at south-east of Mt. Chelmos. The substrates are mostly of carbonatic origin and are represented by dolomites and various types of limestones (bioclastic blackish, in plaques or compact). The landscape is quite soft with smoothed summits, interspersed with dolines and plateaux, while poorly developed are the rock walls and screes. Below 1800 m, the bioclimate falls into supra-temperate sub-Mediterranean belt, while above 1800–1900 m falls into oro-temperate sub-Mediterranean one, with ombrotype upper sub-humid. Average annual temperatures in relation to the altitude, range from 10 to 6 °C, with average annual rainfall comprised between 900 and 1000 mm. The mountain forests between 1400 and 1800 m are represented by open woodlands with *Juniperus foetidissima* or sometimes *Acer monspessulanum*, usually mixed with orophilous pulvinate communities that above 1800 m become dominant. Previously, phytosociological investigations were carried out by Quézel [35] and Georgiadis and Dimopoulos [42].

- Mt. Menalon

It is a small mountain range, located at north of Tripoli, in the north-central part of the Peloponnese. The highest peaks are Ostrakina (1980 m), Tzeláti (1875 m) and Kendhrovouni (1730 m), showing not much sloping and bland surfaces. The substrates are prevalentely represented by limestones in plaques and dolomites. The bioclimate above 1500–1600 m of altitude, falls into supra-temperate sub-Mediterranean belt, with annual average temperatures of 9–8 °C, and annual average rainfall of 900–1000 mm. In the highest part, the vegetation is mainly represented by orophilous pulvinate communities, while at elevations lower than 1500 m occur *Abies cephalonica* woodlands. So far, this mountainous area had not yet been investigated from the phytosociological point of view.

- Mt. Parnon

The mountain range of Parnon occupies the eastern part of the southern Peloponnese and consists of not very high peaks (below 2000 m). The highest peaks are Megali Tourla (1934 m), Psari (1839 m), Gaïdanórrachi (1801 m), Profitis Ilias near Agriani (1780 m), Profitis Ilias near Polidroso (1762 m), Koulochera (1760 m), and Prezesi (1701 m). The substrates are represented by Mesozoic limestones and dolomites. The massif has a north-south direction with peaks rather mild interrupted by wide valleys that give a marked discontinuity. The bioclimate falls within Mediterranean Oceanic Pluviseasonal with thermotypes between meso-Mediterranean, at altitudes lower than 1500 m and supra-Mediterranean at higher altitudes. The ombrotype is attributable to sub-humid, with annual average rainfall of 900–1000 mm. The annual average temperatures are around 10–9 °C or even lower (7–8 °C) at the highest peaks. Currently, the woodlands appear very degraded with patches occurring up to an altitude of 1600–1700 m, and are characterized by the dominance of *Abies cephalonica*. Instead, the pulvinate thorny communities are widespread and well represented in the summit stands. Currently, no vegetation data are available on this mountain range.

- Mt. Taygetos

Mt. Taygetos consists of a long chain of about 50 km on a north-south direction, located in the northern part of Mani Peninsula, in the southern Peloponnese. The highest peak is Profitis Ilias (2404 m), with numerous other peaks topping the 2000 m, as Halasmeno (2204 m), Neraïdhovoúna (2025 m), Spanakaki (2024 m) and Aghios Paraskevi (2019 m). Geologically it is mainly constituted by compact limestones, with schist outcrops especially at lower altitudes. The landscape is very rough due to the presence of numerous ridges and peaks with slopes quite steep and rocky. Screes and cliffs are common, as well as plateaux with scattered dolines. The bioclimate is Mediterranean with oceanic pluviseasonal thermotypes ranging from the supra- and oro-Mediterranean in relation to altitude, while the ombrotype is in the top sub-humid. Annual average temperatures above 1500 m vary between 9 °C and 7 °C, while the annual average rainfall of between 900 and 1000 mm. On this mountain the forests occurring at high altitudes are represented by *Abies cephalonica* woodlands, which are frequent up to 1800 m. They usually are linked to carbonatic substrata, while on scists they are replaced by *Pinus pallasiana* woods. Some example of orophilous pulvinate vegetation can be observed from 1200 m of altitude limitedly to the areas with rocky outcrops, penetrating inside of the forest belt. These communities become dominant above 1800 m up to the highest peaks. This kind of vegetation was previously investigated by Quézel [35].

#### 1.1.3. Island Mountains

- Lefkas Island

It is about 100 m from the mainland, with which it is connected by a floating bridge. The highest mountain of the island is Mt. Elati (1158 m), also known as Mt. Stavrota, charaterized by some peaks, as Agios Elias, Pirgos, and Mega Oros. This mountain is covered with phrygana communities, which is replaced by orophilous pulvinate communities in the summit, while the forests are currently absent. The substrata are mainly represented by Mesozoic limestones and the bioclimate falls within the meso-Mediterranean with sub-humid ombrotype.

- Cephalonia Island

Mount Ainos, or Black Mountain, constituted mainlyby Mesozoic limestones, is the highest range on Cephalonia, which has a crest long about 14 km with a south-eastern direction. It has its highest peak in Mt. Megas Soros with an elevation of 1628 m, while the second peak towards north-west is Mt. Roudhi, which rises to 1125 m. The bioclimate in the higher stands is typically oro-Mediterranean with sub-humid ombrotype. The slopes between 700 and 1200 m are covered by pine forests and above this altitude there are forests dominated by *Abies cephalonica*. The very windy ridges and the rocky plateaux, located at an altitude not lower than 800 m, are charaterized by a pulvinate dwarf shrub vegetation very rich of endemic orophytes. Observations of this type of vegetation are reported by Knapp [59].

- Euboea Island

The mountains in the Euboea Island, or Evvia, that for dimensions is the second largest island in Greece after Crete, are numerous and well represented. The main peaks are Mt. Dirfis (1743 m), Mt. Ochi (1394 m), and Mt. Pyxaria (1341 m), constituted by metamorphic substrata (scists) mixed to triassic marbles. The highest summits are usually affected in by a oro-Mediterranean bioclimate, tending to meso-temperate sub-Mediterranean one, with sub-humid ombrotype. The orophilous dwarf shrub vegetation is well represented in the mountain summit of this island and in particular on Mt. Dirfis. No data on these orophilous communities are reported in literature.

- Samos Island

On the Island of Samos (East Aegean), the peaks with altitudes above 1000 m are Mt. Kerkis (1433 m) and Mt. Ambelos (1153 m). Geologically, Mt. Kerkis consists of Mesozoic limestones, while Mt. Ambelos (or Karvounis) is mainly represented by schists and marbles. The bioclimate affecting these mountains falls within the meso-Mediterranean belt, with sub-humid ombrotype. In the summits of these mountains, above 1000 m of altitude, are located orophilous pulvinate communities, often dominated by echinophytic shrubs. Previously, some observations of this vegetation in Samos were reported by Christodoulakis and Georgiadis [41].

- Lesvos Island

Lesvos (or Lesbos), near to the Turkish coast, is mainly mountainous with an important large peak, represented by Mt. Olympus (967 m), located in the southern part of the island. The top of this mount is constituted by an outcrop of Mesozoic crystalline limestones, with very steep and eroded slopes. From the bioclimatic point of view, this area falls in the meso-Mediterranean belt with sub-humid ombrotype. The thorny orophylous shrub vegetation is circumscribed to this cacuminal habitat. No data on these orophilous communities are reported on literature.

- Chios Island

The island, separated from Turkey by the Çeşme Strait, is prevalently mountainous with numerous peaks occuring mainly in the northern part. The largest of these mountains are Mt. Pelineon (1297 m), Mt. Epos (1188 m), Mt. Oros (1186 m), M. Plakes (912 m), and M. Marathovouno (796 m), which show markedly rocky surfaces, often very sloped and rugged. The substrata are prevalently constituted by Mesozoic limestones or more rarely by schists. The mountain area is affected by a meso-Mediterranean sub-humid bioclimate. The cacuminal stands are usually colonized by orophilous dwarf shrubs communities. No data on these orophilous communities are reported on literature.

- Thassos Island

This island is the northernmost of the Aegean Sea, in front of Kavala (N-Greece). The highest peak of Thassos is Mt. Ipsario (1208 m), characterized by schists and Mesozoic marbles. This territory is affected by meso-Mediterranean sub-humid bioclimate. The orophilous thorny shrub vegetation is exclusively localized on limestone outcrops. No data on these orophilous communities are reported in the literature.

### 1.2. Geology

The mountains of central and southern Greece with peaks topping 1700 m are found mainly in Sterea Ellas at the north of Corinth Gulf and in Peloponnese. They are represented mainly by carbonate mountain ranges, characterized by numerous peaks with variable altitudes, many of them reaching 2000 m. As regards the islands, apart from Crete that is not treated in this work, only those reaching altitudes above 900–1000 m have been surveyed by the authors. In particular, among them there are the islands of Cephalonia, Lefkas, Euboea, Samos, Lesbos, Chios, and Thassos, which are characterized by orophilous dwarf shrub communities in the summit of their mountains.

According to literature data [60,61,62], the investigated mountains are geologically constituted in the highest parts by limestones and dolomites dating back to the Mesozoic, or more rarely carbonatic rocks of the Miocene. In some islands, the cacuminal stands are charaterized by outcrops of marbles and schists dating back to the Mesozoic or Paleozoic.

Based on our personal observations in the field, due to the marked erosion, the soils are generally very shallow, accumulating mainly in the cracks and crevices of the rock, as well as in the small depressions or dolines. In the less inclined or often flat stands, the soils show usually a scarce maturity and are mixed with a rich skeletal component, often quite coarse. In these mountains, the screes are also quite frequent, especially in the highest parts, consisting of clasts with varying granulometry that are originated from gelifluxion phenomena in correspondence of the highest peaks or at the base of the cliffs, for the fragmentation of overlying rocky walls.

### 1.3. Bioclimate

According to the classification proposed by Rivas-Martínez [63], Rivas-Martínez and Rivas-Saenz [64] and Rivas-Martínez et al. [65,66], the bioclimate affecting the investigated Greek mountains falls, limited to the highest stands, in the Temperate oceanic sub-Mediterranean one, while as concerns the lower ones in the Mediterranean oceanic pluviseasonal one. Regarding the thermotypes, they ranged in the first case between the supra-temperate and oro-temperate belts limitedly to the sub-Mediterranean variant. At altitudes below to 1500–1600 m, the territories are affected essentially by the meso-or supra-Mediterranean thermotype. On the most southern mountains of Peloponnese and islands, the bioclimate tends to assume connotations more markedly Mediterranean, with thermotypes referring to supra- and oro-Mediterranean in the highest peaks, and meso-Mediterranean in low altitude ones. In particular, on the mountains localized in the islands of the eastern and northern Aegean, the thermotypes fall almost exclusively in the meso-Mediterranean termotype. With regard to rainfall, the tops of the mountain ranges of these regions are affected by ombrotypes between the upper sub-humid one and the upper humid one, tending in the slopes most exposed to moist marine winds, towards the hiper-humid. In fact, the cacuminal stands, and those at altitudes usually above 1600–1700 m, are characterized by rather moist and cold winters, with more or less long periods of snow cover, while the summers are quite hot and dry. Throughout the year, these areas are normally affected by strong winds, as well as by extreme daily temperature ranges and fog regimes.

**Figure 2:**
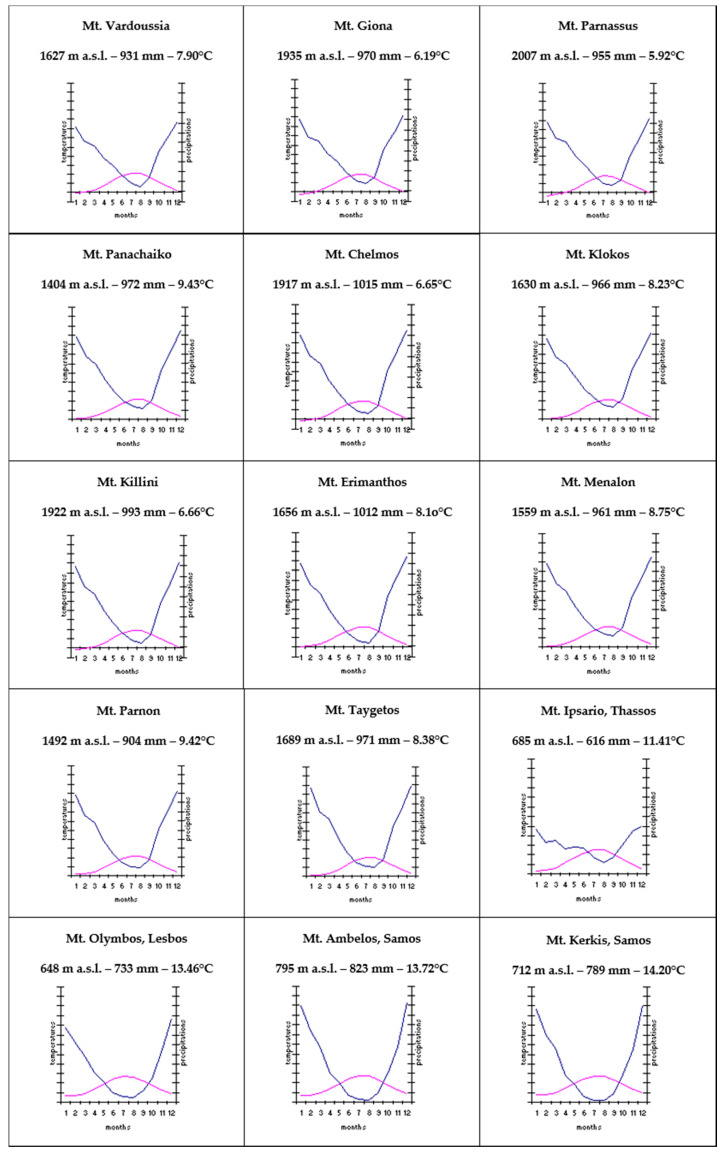
Climograms of 15 thermo-pluviometric stations of some continental and insular mountains from Greece, obtained from data interpolated by WorldClim according to [68,69].

Just as an example, the charts built according to the scheme proposed by Walter and Leith [67] are provided, using the interpolated data published by Hijmans et al. [68,69], which are listed in the “Global climate surfaces” and relate to the period 1950–2000. These data have been taken from a map grid of 10 km^2^, in which the toponym is not given but only the geographical coordinates of the centroid of the square (Figure 2).

### 1.4. Floristic Considerations

The floristic set, involved in the orophilous pulvinate vegetation occurring in the mountains of central and southern Greece, as well as some Ionian (Lefkas and Cephalonia) and Aegean islands (Thassos, Lesbos, Chios, and Samos) of this country, is here investigated. Based on the phytosociological relevés used for this study, both literature and unpublished data, a floristic checklist has been created (Appendix A, Table A1), where all the taxa at specific and infraspecific level (634 taxa) are reported. As regards the nomenclatural aspects, life forms and chorological elements, the most recent floras and checklists were used [70,71,72,73,74,75,76,77]. In the cases of very complex taxa, belonging to critical species or groups, when possible, specific revision treatment were followed, or a taxonomic update based on herbaria researches and literature were carried out (see Taxonomic Remarks).

In the context of this orophilous vegetation, some dwarf shrubs, showing a thorny pulvinar more or less compact habit, are physiognomically very important, since they often tend to cover very large surfaces. They are mainly represented by tragacanthoid plants belonging to the genus *Astragulus*, which are usually endemic and often confined to one or a few mountain ranges. Among them there are: *Astragalus rumelicus*, represented in Greece by three subspecies distributed one in the center-north of Greece (subsp. *rumelicus*), another in the Peloponnese (subsp. *taygeticus*) and a last on the island of Euboea (subsp. *euboicus*); *A. cephalonicus* restricted in some Ionic islands (Lefkas and Cephalonia); *A. corinthiacus* in Mts. Parnassus and Giona; *A. taygeteus* circumscribed to Mt. Taygetos; *A. tymphresteus* distributed on mountain ranges of the central-northern of the Balkan area; *A. cylleneus* occurring only on Mt. Killini and Mt. Chelmos; *A. calavrytensis* exclusive of Mt. Chelmos; *A. parnassi* known from some massifs of Sterea Ellas and Mt. Ossa; *A. creticus* subsp. *samius*, *A. lesbiacus* and *A. condensatus* restricted to on Eastern Aegean Islands.

Another thorny and cushion-like *Astragalus* distributed on the mountains of Greece is *A. angustifolius* Lam., a species having an eastern Mediterranean range, which shows a high polymorphism. According to Brullo et al. [78], within *A. angustifolius,* it is possible to distinguish various taxa differentiated at subspecific level, such as: subsp. *angustifolius*, exclusive of Anatolia and Caucasus, subsp. *balcanicus*, distributed in the northern Balkanic Peninsula (N-Greece, Bulgaria, Macedonia, Serbia, Albania), subsp. *erinaceus*, from central-southern Greece (Sterea Hellas, Attica, Peloponnese and Cephalonia), subsp. *echinoides* from Crete, subsp. *aegeicus*, occurring in some eastern Aegean Islands (Lesbos, Samos, and Chios) and subsp. *odonianus* from the Thassos Islands (N-Greece).

Other tragacanthoid shrubs, or otherwise thorny, occuring in these habitats are: Acantholimon graecum, A. aegaeum, Silene urvillei, Atraphaxis billardieri, Minuartia juniperina, M. stellata, etc.

Many other orophilous endemics belong to genera or species complexes, often representing geographical vicariants, such as: *Marrubium* (*M. cylleneum*, *M. velutinum*), *Nepeta (N. argolica, N. spruneri*, *N. parnassica*, *N. camphorata*, *N. orphanides*), *Sideritis* (*S. clandestina* subsp. *clandenstina*, and subsp. *peloponnesiaca*, *S. raeseri*, *S. sypilea*), *Anthemis* (*A. cretica*, *A. laconica*, *A. samia*, *A. spruneri, A. aciphylla*).

Other taxa well represented in the Greek mountains belong to some critical groups, such as: *Koeleria mitrushii,* closely related to *K. splendens*, *Armeria orphanidis*, related to *A. majellensis* and *A. canescens*, *Stipa endotricha*, closely related to *S. pulcherrima*, and *S. holosericea*, related to *S. fontanesii*.

## 2. Results and Discussion

### 2.1. Taxonomic Remarks

During the phytosociological investigation carried out in the high-mountains of Greece, we have collected several orophytes belonging to the genus *Astragalus* and *Allium*, which are very peculiar from the taxonomical point of view and traited as taxa new to science. Moreover, the taxonomic rank in some of them was modified. They are the following:

(1) *Astragalus corinthiacus* Brullo, Giusso & Musarella, sp. nov.

Holotype: Greece, Sterea Hellas, eastern slopes of Mt. Parnassus, on the bottom of carbonatic dolines with deep silt-clay soils, ca. 1800 m a.s.l., 07.VII.2006, *S. Brullo, C.M. Musarella & G. Giusso del Galdo s.n*. (CAT).

Diagnosis: *Astragalo cephalonico affinis sed stipulis coriaceis, uninervatis, sparsim piloso-ciliatis dorsaliter, aristis triangularibus, 3–6 mm longis, foliolis lineari-ellipticis, 1–2.2 mm latis, viridibus, pubescentibus vel laxe lanuginosis, bracteis subulatis vel lineari-subulatis, dense ciliatis dorsaliter, numquam glabris margine, bracteolis praesentibus, tubo calice 4–4.5 mm longo et dentibus subequalibus, 9–10 mm longis, corolla roseo-purpurescenti, vessillo 16.5–18 mm longo, hastato, tubo staminorum 15 mm longo.*

Description: Dwarf shrub forming a loose, spiny cushion, 30–60 cm tall. Stems woody, tomentose-lanuginose, with hairs 0.2–1.5 mm long, loosely branched, tough, with persistent stipules and rachis in the old parts of the branches. Stipules coriaceous, straw coloured, 8–12 mm long, usually 1-nervate, adnate to the petiole for 4.5–7 mm, ciliate at the margin, sparsely lanuginose dorsally, free part triangular, acuminate, 3–6 mm long. Leaves paripinnate, 2.5–4 cm long, with ivory rachis, covered by sparsely lanuginous hairs; petiole 8–20 mm long; terminal spine 3–5 mm long. Leaflets linear-elliptical, dark green, acuminate at the apex, 3–8.5 × 1–2.2 mm, more or less paired, covered by sparsely and appressed lanuginose hairs. Leaflet peduncle 0.2–0.4 mm long. Inflorescence crowded in subsessile racemes up to 8–10-flowered. Bracts subulate to linear-subutate, hyaline, usually curved dorsally, exceeding calyx tube, 8–10 mm long, 0.5–2 mm wide, dorsally ciliate-pilose, often glabrous laterally. Bracteoles subulatis up to 8 mm longis, cilate-pilose. Calyx cylindrical, white-hyaline, densely covered by rigid hyaline hairs 1–3 mm long, up to the teeth apex, tube 4–4.5 mm long, teeth subulate, subequal, 9–10 mm long. Corolla pink-purplish: standard hastate, 16.5–18 mm long, minutely emarginate, with blade 9–10 × 5.5–6 mm; wings 13–14 mm long, with blade 6–7 × 1.5–1.7 mm and auricle 0.6 mm long; keel 14–14.5 mm long. Staminal tube 15 mm long and free stamen 13 mm long; anthers o.8 mm long. Pistill 15–16 mm long; ovary 4–4.5 mm long, densely hairy; style hairy at the base. Pod 7 mm long, ellipsoid, densely pilose-appressed.

Etymology: From “*Corinthus*”, the Latin name of the city of Corinth and its gulf between Sterea Ellas and Peloponnese.

Distribution: The new species occurs in the mountain places of Mt. Parnassus and Mt. Giona where it is localized in the carbonatic dolines on silt-clay soils, mainly on the eastern and northern slopes at 1600–1900 m a.s.l.

Notes: This new species shows close relationships with *A. cephalonicus* C. Presl, occurring in the Ionian islands of Cephalonia and Lefkada. In particular, *A. cephalonicus* differs from *A. corinthiacus* in having stipules membranaceous, linear-triangular, plurinerved, densely ciliate-hirsute, free part 5–10 mm long, leaflets oblong, up to 3 mm wide, greyish-green, densely villose, bracts ovate-lanceolate, long ciliate, 2.5–2.8 mm wide, bracteoles lacking, calyx with tube 5.5–7 mm long, teeth unequal, the three lower teeth 5.5–7 mm long, the upper two 7–9 mm long, corolla whitish to pinkish-white, standard spathulate with blade 13–16 × 5.5–6 mm, staminal tube 14 mm long. Previously Strid [73] also pointed out that the populations of *A. cephalonicus* of Cephalonia differed from those ones occurring in Sterea Ellas.

(2) *Astragalus rumelicus* Bunge, Mém. Acad. Imp. Sci. St.-Pétersbourg, Sér. 7. 15(1): 81 (1868)
  (a) subsp. *euboicus* (Širj.) Brullo, Giusso & Musarella comb. et stat nov.
    Bas.: *Astragalus rumelicus* var. *euboicus* Širj., Repert. Spec. Nov. Regni Veg. 47: 200. 1939.
  (b) subsp. *rumelicus*
  (c) subsp. *taygeticus* (Širj.) Brullo, Giusso & Musarella comb. et stat. nov.

Bas.: *Astragalus rumelicus* var. *taygeticus* Sirj., Repert. Spec. Nov. Regni Veg. 47: 199. 1939.

Syn.: *Astracantha rumelica* (Bunge) Reer & Podlech subsp. *taygetica* (Širj.) Reer & Podlech, Mitt. Bot. Staatssaml. Munchen 22: 544. 1986.

Notes: According to Širjaev [79] the two subspecies differ from the type in some morphological characteristics. In particular, the subsp. *euboicus* differs in having leaflets denser, outspread white-villous, calyx with short teeth, and corolla 11 mm long, while the subsp. *taygeticus* apart from having leaflets denser outspread white-villous, is differentiated by a calyx with longer teeth, and corolla 13 mm long.

(3) *Allium hirtovaginatum* subsp. *samium* Brullo, Pavone & Salmeri, subsp. nov.

Holotype: Greece. Samos, Mt. Kerkis, esemplare coltivato, 22 July 1993, *S. Brullo s.n.* (CAT).

Diagnosis: *A typo differt scapo usque ad 35 cm alto, foliis 5–6, pilis subadpressis 0.3–0.4 mm longis, florum pedicellis usque ad 7 cm longis, spatha 3.5–7(−9) cm longa, appendice usque ad 40 mm longa, perigonio 7–8 mm longo, tepalis e purpura superne albo-roseis, exterioribus lineari-lanceolatis, obtusiusculis vel rotundatis apice, 2–2.5 mm latis, staminum filamentibus subulato-triangulis, exterioribus usque ad 1.8 mm longis, interioribus 2–2.5 mm longis, annulo 1.2–1.4 mm alto, capsula 4.2 × 4 mm.*

Description: Bulb ovoid, sometimes bulbiliferous, 15–20 × 8–12 mm, with brown tunics, fibrous slightly reticulate, split at the base, covering the stem up to 2 cm. Stem erect, flexuous 15–35 cm high, covered by the leaf sheaths 1/2–2/3 of its length. Leaves 5–6, filiform, semicylindrical, shorter than the inflorescence, 4–20 cm long, hairy with dense subappressed hairs 0.3–0.4 mm long. Inflorescence fastigiate, unilateral, with 5–10(−12) flowers on pedicels 1–5(−7) cm long. Spathe 1-valved, longer than the inflorescence or subequal, persistent, 9–11-nerved, 3.5–7(−9) cm long, with an appendage 15–40 mm long. Bostryces 2. Perigon cylindrical-suburceolate, 7–8 mm long; tepals white-pink, tinged with purple in the upper part, with a brown-purplish mid-vein, the outer linear-lanceolate, entire, subobtuse or rounded at the apex, 2–2.5 mm wide, the inner linear-oblong, rounded and feebly gnawed-undulate at the apex, 1.2–1.8 mm wide. Stamens with white filaments, yellowish below, subulate-triangular, unequal, the outer 0.9–1.8 mm long and 0.8–1 mm wide at the base, the inner 2–2.5 mm long and 1.2–1.5 mm wide at the base, below connate with tepals into an annulus 1.2–1.4 mm high; anthers straw coloured-yellowish, linear-elliptical, apiculate, 1.4 × 0.6 mm. Ovary greenish, subglobose-pyriform, smooth, 1.5–1.8 × 1.3–1.6 mm. Style white, 1–1.8 mm long. Capsule trivalved, subglobose, 4.2 × 4 mm.

Etymology: From Latin “*Samius*” = of Samos, Greek island of E Aegean area.

Distribution and habitat: It is exclusive of Samos, Aegean island near the Turkish coast. It grows in the semirupestrian stands, where it is frequent within ephemeral meadows placed among the phrygana, from sea level to submountain belt.

(4) *Allium cremnophilum* Brullo, Pavone & Salmeri, sp. nov.

Holotype: Greece. Thassos, Ipsario, 26 June 2003, *S. Brullo & G. Giusso s.n.* (CAT).

Diagnosis: *Allio hirtovaginato simili sed bulbo bulbillifero, scapo flexuoso, prostrato-adscendentis, vaginis foliorum per 1/3–1/2 longitudinis tecto, pilis lanuginosis 0.5–1.4 mm longis, perigonio cylindrico-campanulato, tepalis 7.5–8 mm longis, staminum filamentibus omnino luteolis, exterioribus 1.4–2 mm long, interioribus 2.8–3.5 mm, ovario globoso-ovoideo, rugoso superne, 1–1.1 × 1.1–12 mm, capsula obovoidea, 3.7 × 3.4 mm.*

Description: Bulb ovoid, often paired, bulbiliferous, 8–13 × 6–10 mm, with brown tunics, reticulate-fibrous, split at the base, covering the stem up to 2 cm. Stem flexuous, prostrate-ascending, 6–11 cm high, covered by the leaf sheaths 1/3–1/2 of its length, often bearing two inflorescences. Leaves 3, filiform, subcylindrical, longer than the inflorescence or subequal, 4–8 cm long, hairy-woolly with patent hairs 0.5–1.4 mm long. Inflorescence fastigiate, unilateral, with 4–8 flowers on pedicels 10–30 mm long. Spathe 1-valved, longer than the inflorescence or subequal, with 7 nerves of which 4 are incomplete, 8–32 mm long, with an appendage 5–20 mm long. Bostryces 2. Perigon cylindrical-campanulate, 7.5–8 mm long; tepals whitish to white-pinkish, with purplish mid-vein, the outer lanceolate, entire and acute at the apex, 1.8–2 mm wide, the inner linear-oblong, with purple striae above, subobtuse and gnawed-undulate the apex, 1.6–1.8 mm wide. Stamens with yellowish filaments, triangular-subulate, unequal, the outer 1.4–2 mm long and 0.5–0.8-mm wide at the base, the inner 2.8–3.5 mm long and 0.8–1 mm wide at the base, below connate with tepals into an annulus 0.8–1 mm high; anthers straw coloured, elliptical, apiculate, 1.5 × 0.8–0.9 mm. Ovary greenish, globose-ovoid, rugose above, 1–1.1 × 1.1–1.2 mm. Style white, 1.3–1.4 mm long. Capsule trivalved, obovoid, 3.7 × 3.4 mm.

Etymology: From the Greek words “*cremnos*” = crevice and “*philos*” = fond of, in reference with its habitat.

Distribution: At present, this species occurs only in Thassos at the top of Mt. Ipsario, a N Aegean island near Kavala, Greece. Usually, it grows in depth of calcareous crevices at c. 1200 m of altitude, mixed with chasmophytic vegetation or more rarely inside the thorny dwarf-shrubs of *Astragalus angustifolius* subsp. *odonianus*.

(5) *Allium cylleneum* Brullo, Pavone & Salmeri, sp. nov.

Holotype: GREECE. Peloponnisos, Mount Kyllini, 5 July 2006, *S. Brullo, G. Giusso & C. Musarella s.n.* (CAT).

Diagnosis: *Allio cremnophilo simili sed bulbis aggregatis, tunicis fibrosis leviter reticulatis, usque ad 4 cm scapum tegentibus, scapo e vaginis foliorum per 3/4 longitudinis tecto vel totaliter, foliis usque ad 11 cm longis, pilis curvatis, subappressatis, 0.3–0.6 mm longis, spatha 5–7-nervata, nervis completis, perigonio campanulato-urceolato, tepalis 6.5–7 mm longis, omino eroso-undulatis et rotundatis superne, staminum filamentibus albidis, subulatis, ovario ovoideo, laeve, capsula 3.5 × 4 mm.*

Description: Bulb ovoid, clustered, 12–20 × 8–12 mm, with pale brown tunics, fibrous with subparallel fibres feebly reticulate, split at the base, covering the stem up to 4 cm. Stem flexuous, erect or erect-ascending, 4–10 cm high, covered by the leaf sheaths from 3/4 of its lengt to totally. Leaves 3, filiform, subcylindrical, normally longer than the inflorescence, 6–11 cm long, hairy with curved subappressed hairs 0.3–0.6 mm long. Inflorescence fastigiate, unilateral, with 3–6 flowers on pedicels 5–25 mm long. Spathe 1-valved, shorter than the inflorescence or subequal, 5–7-nerved, 18–35 mm long, with an appendage 6–13 mm long. Bostryces 2. Perigon campanulate-urceolate with tepals white-pinkish, with purple striae and mid-vein, gnawed-undulate and rounded at the apex, 6.5–7 × 1.6–1.8 mm, the outer linear-lanceolate, the inner linear-elliptical. Stamens with white filaments, subulate, unequal, the outer 1.2–2 mm long and 0.6–0.8 mm wide at the base, the inner 2.8–3.3 mm long and 0.7–1 mm wide at the base, below connate with tepals into an annulus 1–1.2 mm high; anthers straw coloured, ovate-elliptical, apiculate, 1.3–1.4 × 0.7–0.8 mm. Ovary yellow-greenish, ovoid, smooth, 1–1.1 × 1.1–1.2 mm. Style white, 1.2–1.3 mm long. Capsule trivalved, obovoid, 3.5 × 4 mm.

Etymology: from Latin “*cylleneus*” = from Mt. Kyllini (N Peloponnese).

Distribution: At present, this species seems confined to the top of Mt. Profitis Ilias, the highest summit of Kyllini massif in N Peloponnese (Greece). It is quite rare and occurs in the orophilous dwarf-shrub communities with *Astragalus rumelicus* subsp. *taygeticus* on Mesozoic limestone, at 2200–2400 m of altitude.

(6) *Allium orosamium* Brullo, Giusso & Musarella, sp. nov.

Holotype: Greece, Island of Samos, Mt. Kerkis, near the top at 1100 m a.s.l., 02/07/2003, *S. Brullo s.n.* (CAT).

Diagnosis: *Allio stamineo simili sed tunicis bulborum fibroso-coriaceis, scapo usque ad 27 cm alto, spathis 5–6-nervatis, inflorescentia 20–35 floribus, perigonio campanulato, tepalis brunneo-viridibus, max. 5 mm longis, 2–2.2 mm latis, staminum filamentibus albidis, 3.5–4.5 mm longis, ovario obovoideo, papilloso, 3.2–3.5 × 2.2–2.4 mm, stilo 0.5 mm longo, capsula subglobosa 5 × 5.2 mm.*

Description: Bulb ovoid, 12–15 × 7–9 mm, with outer tunics fibrous-coriaceous, dark brown, the inner ones membranous, whitish. Scape glabrous, erect, 9–27 cm high, covered by leaf sheaths for 1/2–2/3 of its length. Leaves 3–5, green, semicylindrical, costate, with blade 10–20 cm long. Spathe persistent, with 2 unequal valves, longer than umbel, the larger 5–6-nerved, 3–8 cm long, the smaller 5–6-nerved, 2–5 cm long. Inflorescence lax, diffuse, 20–35-flowered; pedicels unequal, flexuous, 7–20 mm long. Perigon campanulate, with tepals unequal, brownish green tinged with brown-purplish, oblong, rounded at apex, the outers 4.5–4.8 × 2.2 mm, the inners 4.8–5 × 2–2.1 mm. Stamens simple, exserted, with filaments subulate, 3.5–4.5 mm long, white, connate at base into an annulus 0.6–1 mm high; anthers oblong, straw, rounded at apex, 1.2 × 0.7 mm. Ovary obovoid, yellow-greenish, papillose above, 3.2–3.5 × 2.2–2.4 mm. Style white, 0.5 mm long. Capsule widely subglobose, green, 5 × 5.2 mm.

Etymology: From “*oros*” Greek name of “mountain” and “*Samius*” Latin adjective of Samos (Aegean Island).

Distribution: This species is localized in the top of Mt. Kerkis (Samos island), where it grows in the carbonatic rocky stands within the community characterized by *Astragalus creticus* subsp. *samius*.

(7) *Allium karvounis* Brullo, Giusso & Musarella, sp. nov.

Greece, Island of Samos, Mt. Ambelos, near the top at 1100 m a.s.l., 11/06/2005, *S. Brullo & C.M. Musarella s.n.* (CAT).

Diagnosis: *Allio stamineo simili sed bulbis maioribus, tunicis fibroso-coriaceis, scapis minoribus e vaginis foliorum per 1/2 longitudinis tectis, lamina foliorum rigida, spathis brevioribus, inflorescentia usque ad 80 floribus, pedicellis usque ad 40 mm longis, tepalis minoribus 3.8–4 × 1.6–1.8 mm, staminum filamentibus brevioribus, ovario obovoideo, laeviter papilloso, maiore, stilo 2–6 mm longo, capsula maiore.*

Description: Bulb ovoid, 10–15 × 8–12 mm, with outer tunics fibrous-coriaceous, dark brown, the inner ones membranous, whitish. Scape glabrous, erect, 18–24 cm high, covered by leaf sheaths for 1/2 of its length. Leaves 3–4, green, semicylindrical, costate, with blade rigid, 8–20 cm long. Spathe persistent, with 2 unequal valves, longer than umbel, the larger 7-nerved, 4–7 cm long, the smaller 5–7-nerved, 2–4 cm long. Inflorescence fastigiate, compact, 25–80-flowered; pedicels unequal, flexuous, 8–40 mm long. Perigon conic-campanulate, with tepals equal, greenish yellow tinged with purplish, oblong, rounded at apex, 3.8–4 × 1.6–1.8 mm. Stamens simple, exserted, with filaments subulate, 3.5–5 mm long, white below, purplish above, connate at base into an annulus 0.4–0.5 mm high; anthers oblong, yellow, rounded at apex, 1.2–1.3 × 0.8–0.9 mm. Ovary obovoid slightly throttled, green, slightly papillose above, 1.8–2 × 1.8–2 mm. Style white, 2–6 mm long. Capsule obovoid, green, 4.5–5 × 4.5–5 mm.

Etymology: From “*Karvounis*” old name of Ambelos mount from Samos (Aegean Island).

Distribution: This species is localized in the top of Mt. Ambelos from Samos island in the Aegean area, where it grows into the orophilous cushion-like vegetation.

(8) *Allium lefkadensis* Brullo, Giusso & Musarella, sp. nov.

Holotype: Greece, Lefkàda, Ionian Islands, Mt. Elati, near the top at 1000 m a.s.l., 16/07/2011, *S. Brullo & G. Giacalone s.n.* (CAT).

Diagnosis: *Allio stamineo simili sed tunicis interioribus bulborum brunneo-purpurescentibus, scapis minoribus e vaginis foliorum per 1/4–1/3 longitudinis tectis, lamina foliorum 8–16 mm, spathis brevioribus, tepalis minoribus, pruinosis, staminum filamentibus supra roseam suffusis, ovario maiore, stilo longiore.*

Description: Bulb ovoid, 15 × 10 mm, with outer tunics coriaceous, dark brown, the inner ones membranous, reddish-brown. Scape glabrous, erect, 10–16 cm high, covered by leaf sheaths for 1/4–1/3 of its length. Leaves 4, green, semicylindrical, costate, 8–16 cm long. Spathe persistent, with 2 unequal valves, longer than umbel, the larger 7-nerved, 3–4 cm long, the smaller 5-nerved, 1.5–2 cm long. Inflorescence lax, diffuse, 20–25-flowered; pedicels unequal, flexuous, 10–25 mm long. Perigon conical-campanulate, with tepals equal, greenish yellow pruinose, oblong, rounded at apex, 4.5 × 2 mm. Stamens simple, exserted, with filaments subulate, 6–7 mm long, white below and slightly tinged with pink above, connate at base into an annulus 0.5–0.6 mm high; anthers oblong, straw, apiculate at apex, 1.2–1.4 × 0.6–0.7 mm. Ovary subglobose, yellow-greenish, slightly rugose-papillose above, 2 × 2–2.1 mm. Style white, 2.5–6 mm long. Capsule not observed.

Etymology: From “Lefkàda”, the Greek Ionian island where this species is confined.

Distribution: The species was observed only on the top of Mt. Elati at Lefkàda Ionian Island.

### 2.2. Phytogeographical Analisys

Regarding the life forms (Table 1), this florula is characterized mainly by hemicryptophytes (H) (43.06%), followed by chamaephytes (Ch) (34.86%), while geophytes (G) (9.78%) and therophytes (T) (9.15%) are clearly inferior. Finally, nanopharenophytes (NP) (2.68%) and phanerophytes (P) (0.47%) are negligible. In fact, due to the extremely harsh conditions of these high mountain habitats, only plants with particular structural adaptations can aggregate in plant communities able to express their potential to the fullest. In this respect, the hemicryptophytes and chamephytes, being perennial plants characterized by a habit slightly raised from the soil, are those that are best suited to these environments. They are affected by a climate with very cold winter, characterized by long periods of snow cover, strong winds blowing on the mountain tops, the marked daily temperature ranges, hot and dry summers. In particular, these habitats are characterized by the dominance of dwarf shrub chamaephytes, showing often a pulvinate habit that tolerates better these extreme environmental conditions. Instead, nanopharenophytes and phanerophytes do not go beyond the timberline, while geophytes and therophytes, having no adaptions, are very rare and grow usually into the shrubs.

From the chorological viewpoint, being Mediterranean mountains, the floristic set featuring these habitats, shows a clear predominance of Mediterranean species (Table 2). In particular, the Mediterranean element shows the highest percentage (42.43%), within which the more representative are the East-Mediterranean taxa (29.65%), while the circum-mediterranean ones present lower percentages (9.62%). As concerns the other mediterranean elements, they are scarcely represented. Apart to the Mediterranean element, the endemic one is very high represented (40.38%).

Within the endemic set, different endemisms can be distinguished, such as: Balkan one which is the more frequent (22.66%), CS Greece one (18.75%), Peloponnese one (17.58%), Greece one (15.23%), Sterea Ellas one (6.25%), while the other endemic species occuring in the Greek islands show a lower percentage, such as those ones of E-Aegean islands (5.86%), Ionian islands (2.73%), N-Aegean (2.73%), and Euboea (2.34%) (Table 3). Other elements are less significant, such as the European one (11.67%) and the wide distribution one (5.52%), the latter including circumboreal, cosmopolite, and paleotemperate species (Table 2).

This diversity of endemic species in cacuminal stations of the investigated mountain ranges of Greece is clearly to be connected to the paleogeographic vicissitudes that these territories have had in the last million years. Most probably the geographical isolation of these mountain massifs has clearly increased the speciation processes in the orophilous populations confined in the cacuminal stands, mainly in those ones having a relic character.

### 2.3. Phytosociological Investigation

Previously, the orophilous pulvinate vegetation of central-southern and insular Greece hitherto known in literature were included in *Daphneeto-Festucetea* class as described by Quézel [35]. Within this class, the associations were arranged according to the syntaxonomical scheme proposed by that author, afterwards modified by Quézel et al. [80]:

*DAPHNO OLEOIDIS-FESTUCETEA VARIAE* Quézel 1964, corr. Quézel et al. 1992

Syn.: *Daphneeto-Festucetea* Quézel 1964, Vegetatio, 12:325

Lectotypus: *Daphno oleoidis-Festucetalia variae* Quézel 1964

  *DAPHNO OLEOIDIS-FESTUCETALIA VARIAE* Quézel 1964, corr. Quézel et al. 1992

  Syn.: *Daphneeto-Festucetalia* Quézel 1964, Vegetatio, 12:325

  Lectotypus: *Eryngio multifidi-Bromion fibrosi* Quézel 1964

    *STIPO PULCHERRIMAE-MORINION PERSICAE* Quézel 1964, corr. Quézel et al. 1992

    Syn.: *Stipeto-Morinion* Quézel 1964, Vegetatio, 12: 326

    Lectotypus: *Scabioso taygeteae-Onosmetum leptanthae* Quézel 1964

      *Scabioso taygeteae-Onosmetum leptanthae* Quézel 1964 Vegetatio, 12:327

      Syn.: Ass. à *Scabiosa taygetea* et *Onosma leptanthum* Quézel 1964

      *Galio lucidi-Ribetum uvae-crispae* Quézel 1964, Vegetatio, 12:329

      Syn.: ass. à *Galium lucidum* et *Ribes uva-crispa* Quézel 1964

      *Onobrychido minoris-Juniperetum foetidissimae* Quézel 1973, Biol. Gallo-Hell. 5(1):147

      Syn.: ass. à *Juniperus foetidissima* et *Onobrychis ebenoides* var. *minor* Quézel 1973

      *Juniperetum foetidissimae* Georgiadis & Dimopoulos 1993, Bot. Helv. 103:152 (nom. inval.)

      *scabiosetosum ochroleucae* Maroulis & Georgiadis 2005, Fitosociologia 42(1):37

      *Acer monspessulano-Prunetum mahaleb* Georgiadis & Dimopoulos 1993, Bot. Helv. 103:153 (nom. inval.)

      *Astracantho thracicae-Marrubietum cyllenei* Georgiadis & Dimopoulos 1993, Bot. Helv. 103:153 (nom. inval.)

      *galietosum taygetei* Georgiadis & Dimopoulos 1993, Bot. Helv. 103:153 (nom. inval.)

      *Stipa pennata* subsp. *pulcherrima*-*Sesleria vaginalis* comm. Maroulis & Georgiadis 2005, Fitosociologia 42(1):42

      *Hippocrepis comosa-Stipa pennata* subsp. *pulcherrima* comm. Maroulis & Georgiadis 2005, Fitosociologia 42(1):43

    *ERYNGIO MULTIFIDI-BROMION FIBROSI* Quézel 1964, corr. Quézel et al. 1992

    Syn.: *Eryngieto-Bromion* Quézel 1964, Vegetatio, 12:326

    Lectotypus: ass. à *Astragalus cylleneus* et *Cirsium cylleneum*, Quézel 1964

      *Sideritetum theezantis* Quézel 1964, Vegetatio, 12:331

      Syn.: ass. à *Sideritis theezans* Quézel 1964

      *Cirsio cyllenei-Astragaletum cyllenei* Quézel 1964, Vegetatio, 12:332

      Syn.: ass. à *Astragalus cylleneus* et *Cirsium cylleneum* Quézel 1964

      *Marrubio velutini-Astragaletum cretici* Quézel 1964, Vegetatio, 12:334

      Syn.: ass. à *Astragalus creticus* ssp. *rumelicus* et *Marrubium velutinum* Quézel 1964

      *Astracantho thracicae-Marrubietum cyllenei* Georgiadis & Dimopoulos 1993, Bot. Helv. 103:153, nom. inval.

      *typicum* Georgiadis & Dimopoulos 1993, Bot. Helv. 103: 158 (nom. inval.)

      *festucetosum cyllenecae* Georgiadis & Dimopoulos 1993, Bot. Helv. 103:158 (nom. inval.)

      *Marrubio cyllenei-Astragaletum rumelici* Maroulis & Georgiadis 2005, Fitosociologia 42(1):43

      *Festuco politae-Festucetum cyllenicae* Maroulis & Georgiadis 2005, Fitosociologia 42(1):44

  *ASTRAGALO ANGUSTIFOLII-SESLERION COERULANTIS* Quézel 1964, corr. Quézel et al. 1992.

  Syn.: *Astragaleto-Seslerion* Quézel 1964, Vegetatio, 12:326

  Lectotypus: ass. à *Minuartia stellata* et *Erysimum parnassi* Quézel 1964, Vegetatio, 12:326

      *Rindero graecae-Acantholimetum graeci* Quézel 1964, Vegetatio, 12:336

      Syn.: ass. à *Acantholimon echinus* et *Rindera graeca* Quézel 1964

      *Asteri cyllenei-Globularietum stygiae* Quézel 1964, Vegetatio, 12:337

      Syn.: ass. à *Aster cylleneus* et *Globularia stygia* Quézel 1964

      *Convolvulo cochlearis-Astragaletum lactei* Quézel 1964, Vegetatio, 12:339

      Syn.: ass. à *Convolvulus cochlearis* et *Astragalus lacteus* Quézel 1964

      *Erysimo parnassi-Minuartietum stellatae* Quézel 1964, Vegetatio, 12:340

      Syn.: ass. à *Minuartia stellata* et *Erysimum pusillum* ssp. *parnassi* Quézel 1964

      *Paronychio chionaeae-Thymetum ciliato-pubescentis* Quézel 1964, Vegetatio, 12:341

      Syn.: ass. à *Paronychia chionaea* et *Thymus hirsutus* ssp. *ciliato-pubescens* Quézel 1964

      *Violo-Seslerietum vaginalis* Quézel 1973, Biol. Gallo-Hell. 5(1):152

      Syn.: ass. à *Sesleria coerulans* et *Viola stojanowii* Quézel 1973

      *Euphrasio salisburgensis-Asperuletum nitidae* Quézel 1974, Rev. Biol. Ecol. Medit. 1(1):19

      Syn.: ass. à *Asperula nitida* et *Euphrasia salisburgensis* Quézel 1974

      *Festuco cyllenicae-Asperuletum boissieri* Georgiadis & Dimopoulos 1993, Bot. Helv. 103: 158, nom. inval.

Within this hierarchical arrangement proposed by Quézel [35], the most relevant aspect that emerges from this classification was to use only the altitudinal distribution of plant communities as a discriminating criterion for alliance identification. In fact, according to this author, the order *Daphno-Festucetalia* includes three alliances which are widespread in all the mountains of Greece and are distributed exclusively at different altitudinal ranges. They are: (a) *Stipeto-Morinion* occurring between 1500 and 1700 m; (b) *Eryngieto-Bromion* between 1700 and 2200 m; (c) *Astragaleto-Seslerion* above 2200 m, sloping down sometimes up to 1700 m. Another important factor to note is that these alliances do not provide any information on the real phytogeographic role of the rich floristic contingent featuring this type of orophilous vegetation. Indeed, Quézel [35] considered as characteristics of these alliances mainly species having a wide East Mediterranean or even circum-Mediterranean distribution, showing also a wide altitudinal range and not limited to a narrow belt as stated by the author. In particular, the author proposed *Stipa endotricha* (=*S. pennata* var. *pulcherima*), *Melica ciliata, Asphodeline lutea, Ononis pusilla, Morinia persica, Scutellaria rupestris* (=*S. peregrina* subsp. *rupestris*), *Pterocephalus perennis* and *Anthemis spruneri* (=*A. montana* var. *incana*) as characteristics of *Stipeto-Morinion*; while *Bromus riparius* (=*B. fibrosus*), *Helictotrichon aetolicum* (=*Avena australis*), *Eryngium multifidum, Thymus sibthorpii, Galium thymifolium, Campanula spathulata, Podosmermum canum* var. *alpinum*, and *Carduus tmoleus* (=*C. armatus*), as characteristics of *Eryngieto-Bromion*; finally, *Sesleria tenerrima* (=*S. coerulans*), *Iberis sempervirens, Astragalus angustifolius, Draba lasiocarpa* (=*D. affinis*), *Viola graeca* (=*Viola heterophylla* subsp. *graeca*), *Trinia frigida* (*Apinella frigida*)*, Trinia guicciardii* (*Apinella guicciardii*)*, Acantholimon graecum (= A. echinus), Lactuca intricata* (=*L. graeca*)*, Veronica orsiniana* subsp. *teucrioides* (=*V. austriaca* var. *teucrioides*)*, V. thessalica, V. thymifolia, Asperula boissieri*, and *Tragopogon crocifolius* subsp. *samaritani* as characteristics of *Astragaleto-Seslerion*. On the basis of literature and personal observations, these taxa can not be used to characterize alliances, at most, some of them may be included among the characteristics of order or class, while others are simply accidentals or ubiquitous species. Even, the same author [35,36] underlined often some perplexity in the inclusion of a given association in one of the three alliances identified by him, due to the contemporaneous occurrence in the relevés of characteristic species belonging to all three alliances. Therefore, the alliances identified by Quézel [35] are not being characterized by exclusive species, since they include ubiquitous or species of wider ecological requirements, that are not strictly related to those specific habitats; in this way they do not provide clear information from an ecological and phytogeographical point of view. Based on the above, these alliances do not satisfy the prerequisites required by the sigmatist phytosociological method. They only create a lot of confusion and ambiguity in the syntaxonomical arrangement of this very peculiar kind of orophilous vegetation. In conclusion, these alliances are really ambiguous names that must be rejected (art. 36). Therefore, a new phytosociological framework is necessary to propose. The designation of new alliances must be essentially based on the phytogeographic criteria and such characteristics must include steno-endemic species in order to define unequivocally the geographical boundaries of each syntaxon as well as its syntaxonomical role.

In order to emphasize the distribution of characteristic species within the three alliances and syntaxa of higher rank according to the hierarchic arrangement proposed by Quézel [35], a synoptic table (Appendix B, Table A2) was processed including all the phytosociological relevés published until now on this type of orophilous vegetation in central-southern Greece by Quézel [35,38] and Quézel and Katrabassa [40], as well as other later authors as Georgiadis and Dimopoulos [42] and Maroulis and Georgiadis [44]. From the analysis of this table, the floristic comparison among the hitherto recognized associations, which are well differentiated from the phytosociological viewpoint, shows clearly that the species proposed as characteristics of the alliances are distributed indifferently in all three syntaxa, often with high frequency values. Therefore, it can be easily deduced that a single association cannot be clearly and unambiguously attributed to a specific alliance. Quézel [35] in order to attribute an association to a given alliance, he relied mainly on its altitudinal distribution, rather than considering the information relating to its floristic cortege. Unfortunately, the species selected by the author to define these alliances are not strictly linked to well-defined altitudinal bands, but are widespread almost at all altitudes. From this, it can easily be deduced that, in the case of the orophilous pulvinate vegetation of the Greek mountains, as well as of other geographic territories, this criterion can not be followed. Instead, a purely phytogeographical method must be selected, mainly based on endemic flora, that gives more significant information under phytosociological feature.

On the basis of several unpublished phytosociological relevés carried out by us in the summit stands of most of central and southern Greek mountains as well as in some islands (Figure 1), it was possible to verify that only a strictly phytogeographic policy can allow for a correct syntaxonomic arrangement of these communities, similar to what has been achieved for other Mediterranean territories [22,23,24,25,26,27,28,29,30,31,32,33,34,35,36,37,38,39,40,41,42,43,44,45,46,47,48]. In fact, it is much more realistic and meaningful to identify alliances based on floristic elements that give clear information on phytogeographic correlations of the various associations, rather than on their altitudinal distribution. In particular, the flora characterizing the orophilous community usually shows a significant richness in relict species, often very isolated, or represented by geographical vicariants of remarkable phytosociological significance. Therefore, for a syntaxonomic arrangement that can best express the floristic and structural organization of the pulvinate-orophilous plant communities currently occurring in the Greek mountains, it has to be based on the choice of species suitable for providing more precise information on their phytogeographical role. Following this viewpoint, this study presents a clearer and more comprehensive syntaxonomical overview of these plant communities, reflecting their origin and diversification. Therefore, for a correct floristic characterization of higher syntaxa (alliances, orders and classes) allowing differentiation of specific alliances, the choice should fall on endemics with restricted distribution, such as those confined to one or few neighbouring or close mountain ranges, and it should gradually move on to those endemics with wider ranges and the other more widespread taxa which should be used for the designation of orders and classes. In addition, the floristic contingent that differentiates the higher syntaxa, and particularly in the case of orophilous vegetation featuring the Mediterranean mountains, provides clearer information about the relationships that the plant communities show among them, since they are the result of paleogeographic vicissitudes of the territories that host them.

Furthermore, it must be emphasized that Quézel et al. [80] when lectotypified the class *Daphno-Festucetea* and the corresponding order *Daphno-Festucetalia*, corrected respectively the two names in *Daphno oleoidis-Festucetea variae* and *Daphno oleoidis-Festucetalia variae*. The use of *Daphne oleoides* and *Festuca varia* for giving the name to the two syntaxa brings further confusion and ambiguity, since both species are not pertinent to this type of vegetation. In fact, *Daphne oleoides* is widespread in all Mediterranean mountains and is considered a typical characteristic species of the class *Junipero-Pinetea sylvestris* Rivas-Martínez 1964, as emphasized by Rivas-Martínez [52], Rivas-Martínez et al. [53,54,55,80,81,82], Stanisci [56] and Brullo et al. [57], while in the pulvinate dwarf shrub vegetation it is rather rare and occasional. As concerns *Festuca varia*, this species has a properly alpine distribution and is totally absent in Greece [83], where it is replaced by various other species of this genus. Moreover, it is not possible to identify in a univocal and correct way what is the species of *Festuca* to which Quézel [35] refers in naming these syntaxa.

Besides, among the species proposed by Quézel [35] as characteristic of the class and order is to be noted that some of them, such as *Juniperus communis* var. *hemisphaerica*, *Berberis cretica, Prunus prostrata* and mainly *Daphne oleoides*, are linked to the orophilous communities characterized by phanerophytes and nanophanerophytes belonging to the class *Junipero-Pinetea sylvestris* Rivas-Martínez 1965 nom. invers. propos. (=*Pino-Juniperetea* Rivas-Martínez 1965). This is in agreement with the literature data concerning this type of orophilous forest vegetation [55,56,57,63,84].

In particular as emphasized by Brullo et al. [57] and Mucina et al. [84], the woody communities characterized by the dominance of erect or prostrate conifers occurring in Greece and other central-eastern Mediterranenan territories, must be ascribed to syntaxa exclusive to these mountaints, representated by the order *Berberido creticae-Juniperetalia excelsae* Mucina in Mucina et al. 2016 and some alliances, such as *Berberido aetnensis-Pinion laricionis* (Brullo et al. 2001) Mucina & Theurillat in Mucina 2016, *Juniperion excelso-foetidissimae* Em ex Matevski et al. 2010, *Berberido creticae-Juniperion foetidissimae* Brullo et al. 2001, etc. These forest communities are relegated to the supra- and oro-Mediterranean belts, as well as supra-temperate belt, where they show a fragmentary distribution, which confirms their relict origin. Usually, they occupy an intermediate position between the typical mountain forests of *Querco-Fagetea* and pulvinate orophilous dwarf shrubs linked to cacuminal stands.

Besides as emphasized by Brullo et al. [57], some associations of *Daphno-Festucetea* described by the previous authors must be rather clearly attributed to the class *Junipero-Pinetea sylvestris,* since they show a floristic, structural and ecological feature of the last syntaxon. In particular, this is the case of the “ass. à *Galium lucidum* et *Ribes uva-crispa* Quézel 1964”, “ass. à *Juniperus foetidissima* et *Onobrychis ebenoides* var. *minor* Quézel 1973”, “*Juniperetum foetidissimae* Georgiadis & Dimopoulos 1993”, “*Acer monspessulano-Prunetum mahaleb* Georgiadis & Dimopoulos 1993”, contributing further to confer a marked ambiguity to the class *Daphno-Festucetea*.

For the reasons above mentioned, the names *Daphno-Festucetea* Quézel 1964 and *Daphno-Festucetalia* Quézel 1964 must be proposed as nomina ambigua rejicienda (Art. 36), since they are based on very ambiguous alliances, are sources of continuous errors in the univocal and unambiguous designation of the relative associations. The new names proposed here in order to replace those of the two aforesaid syntaxa are *Cerastio candidissimi-Astragaletea rumelici* and *Eryngio multifidi-Armerietalia orphanidis*, both having a large distribution in the high mountains of southern Balkans and Aegean area.

The floristic analysis of the investigated plant communities occurring mainly in the high mountains of the Peloponnese and Sterea Ellas, as well as in some Ionian Islands and Euboea, showed the existence of significant sets of endemic species, which have a well-defined geographical distribution that allows the identification of alliances based on a clear phytogeographical role, emphasizing especially the palaeogeographical isolation of the various mountain areas among them.

Based on these criteria, it was possible to distinguish in the aforesaid territories some new alliances, which are well circumscribed from the phytogeographical point of view and allow a very realistic arrangement of the orophilous dwarf shrubby vegetation occurring in these Greek high mountains, emphasizing their floristic affinities. These are: *Marrubio velutini-Thymion parnassici,* distributed in the mountains of Sterea Hellas and Attica; *Festuco achaicae-Marrubion cyllenei*, from the North Peloponnese mountains; *Sideritido clandestinae-Asperulion mungieri*, from South Peloponnese mountains. Moreover, *Astragalion cephalonici,* from the Ionian islands of Cephalonia and Lefkada, as well as *Astragalion euboici* from the island of Euboea, must be added to these alliances.

In order to highlight that these alliances have a clear phytosociological role with a well-defined phytogeographic boundary than those proposed by Quézel [35], the associations examined in Appendix B, Table A2 were processed according to this new syntaxonomic scheme. As can be clearly observed in the new Table A3 (Appendix B), the associations fall within floristically well-differentiated alliances, since they are characterized by endemics exclusive of geographically distinct areas, which are characterized by very similar paleogeographic vicissitudes.

In addition, further phytosociological investigations were carried out in the high mountains of some islands of North Aegean area (Thassos, Lesbos, Chios, and Samos) peaking over 1000 m. a.s.l and hosting this kind of vegetation. Within the orophilous pulvinate dwarf shrubs communities occurring in these islands, some characteristic species of *Cerastio candidissimi-Astragaletea rumelici* class are still present (although numerically reduced), while species belonging to the *Eryngio multifidi-Armerietalia orphanidis* order and related alliances are fully missing.

In these insular high-mountain areas, there is a rich set of endemics or eastern Aegean taxa, which allow to differentiate a new vicariant order, namely *Noaeo mucronate-Silenetalia urvillei.* On essential phytogeographical basis, it is possible to distinguish three floristically well-differentiated alliances, represented by *Asperulion samiae*, circumscribed to Samos, *Festuco pseudosupinae*-*Astragalion aegeici*, distributed to Chios and Lesbos, and *Seslerio achtarovii-Anthemidion tenuilobae,* from Thassos. Based on the observations above emphasized, a new syntaxonomic scheme is proposed:

*CERASTIO CANDIDISSIMI-ASTRAGALETEA RUMELICI* Musarella, Brullo & Giusso cl. nov.

  *ERYNGIO MULTIFIDI-ARMERIETALIA ORPHANIDIS* Musarella, Brullo & Giusso ord. nov.

    *MARRUBIO VELUTINI-THYMION PARNASSICI* Musarella, Brullo & Giusso all. nov.

      *Marrubio velutini-Astragaletum rumelici* Quézel 1964

        *typicum*

        *achilleetosum nobilis* Quézel 1964

      *Astragalo lactei-Convolvuletum cochlearis* Quézel 1964

      *Nepeto epiroticae-Astragaletum corynthiaci* (Quézel 1964) Musarella, Brullo & Giusso nom. nov.

      *Nepeto spruneri-Astragaletum corynthiaci* Musarella, Brullo & Giusso ass. nov.

      *Thymo parnassici-Paronychietum polygonifoliae* Quézel 1964

        *typicum*

        *linetosum angustifolii* Quézel 1964

      *Nepeto sprunerii-Astragaletum tymphrestei* Musarella, Brullo & Giusso ass. nov.

      *Violo stojanowii-Seslerietum vaginalis* Quézel 1973

      *Erysimo parnassi-Minuartietum stellatae* Quézel 1964

      *Aurinio gionae-Minuartietum stellatae* Musarella, Brullo & Giusso ass. nov.

      *Achilleo fraisii-Dianthetum tymphrestei* Musarella, Brullo & Giusso ass. nov.

      *Asperulo luteae-Achilleetum umbellatae* Musarella, Brullo & Giusso ass. nov.

      *Astragalo lactei-Asperuletum apiculatae* Musarella, Brullo & Giusso ass. nov.

      *Diantho minutiflori-Festucetum cyllenicae* Musarella, Brullo & Giusso ass. nov.

      *Scabioso ochroleucae-Sideridetum raeseri* Musarella, Brullo & Giusso ass. nov.

      *Ranunculo psilostachydis-Festucetum cyllenicae* Musarella, Brullo & Giusso ass. nov.

      *Edraiantho parnassici-Globularietum cordifoliae* Musarella, Brullo & Giusso ass. nov.

      *Thymo parnassici-Astragaletum parnassi* Musarella, Brullo & Giusso ass. nov.

      *Chamaecytiso hirsuti-Astragaletum parnassi* Musarella, Brullo & Giusso ass. nov.

      *Onobrychido pentelicae-Genistetum parnassicae* Musarella, Brullo & Giusso ass. nov.

      *Allio cithaeronis-Dianthetum serratifolii* Musarella, Brullo & Giusso ass. nov.

      *Inulo methaneae-Sideritetum atticae* Musarella, Brullo & Giusso ass. nov.

    *ASTRAGALION CEPHALONICI* Musarella, Brullo & Giusso all. nov.

      *Helictotricho convoluti-Thymetum holosericei* Musarella, Brullo & Giusso ass. nov.

      *Saturejo cuneifoliae-Thymetum holosericei* Musarella, Brullo & Giusso ass. nov.

      *Scutellario cephalonicae-Astragaletum cephalonici* Musarella, Brullo & Giusso ass. nov.

      *Paronychio graecae-Astragaletum erinacei* Musarella, Brullo & Giusso ass. nov.

    *ASTRAGALION EUBOICI* Musarella, Brullo & Giusso all. nov.

      *Sideritido euboeae-Astragaletum euboici* Musarella, Brullo & Giusso ass. nov.

      *Scabioso webbianae-Phlomidetum samiae* Musarella, Brullo & Giusso ass. nov.

      *Sideritido euboeae-Festucetum cyllenicae* Musarella, Brullo & Giusso ass. nov.

      *Inulo limonellae-Seslerietum krajinae* Musarella, Brullo & Giusso ass. nov.

    *FESTUCO ACHAICAE-MARRUBION CYLLENEI* Musarella, Brullo & Giusso all. nov.

      *Cirsio hypopsilii-Astragaletum taygetici* Quézel 1964 corr.

      *Astero cyllenei-Globularietum stygiae* Quézel 1964

      *Euphrasio salisburgensis-Asperuletum oetaeae* Quézel & Katrabassa 1974 corr.

      *Marrubio cyllenei-Astragaletum calavrytensis* Musarella, Brullo & Giusso ass. nov.

        *elytrigietosum intermediae* Musarella, Brullo & Giusso subass. nov.

        *hippocrepidetum comosae* Musarella, Brullo & Giusso subass. nov.

        *tulipetosum australis* Musarella, Brullo & Giusso subass. nov.

      *Plantagini graecae-Astragaletum cyllenei* Musarella, Brullo & Giusso ass. nov.

      *Festuco achaicae-Minuartietum stellatae* Musarella, Brullo & Giusso ass. nov.

      *Alysso taygetei-Plantaginetum alpestris* Musarella, Brullo & Giusso ass. nov.

      *Hieracio sartoriani-Seslerietum tenerrimae* Musarella, Brullo & Giusso ass. nov.

      *Asperulo boissieri-Festucetum cyllenicae* Georgiadis & Dimopoulos ass. nov.

      *Ranunculo brevifolii-Seslerietum tenerrimae* Musarella, Brullo & Giusso ass. nov.

      *Astragaletum hellenico-erinacei* Musarella, Brullo & Giusso ass. nov.

      *Festucetum polito-cyllenicae* Maroulis & Georgiadis 2005

      *Arenario filicaulis-Festucetum cyllenicae* Musarella, Brullo & Giusso ass. nov.

      *Aurinio moreanae-Lomelosietum crenatae* Musarella, Brullo & Giusso ass. nov.

      *Onosmo malickyi-Astragaletum hellenici* Musarella, Brullo & Giusso ass. nov.

      *Violo graecae-Festucetum cyllenicae* Musarella, Brullo & Giusso ass. nov.

      *Tripodio graeci-Helictotrichetum heldreichii* Musarella, Brullo & Giusso ass. nov.

    SIDERITIDO CLANDESTINAE-ASPERULION MUNGIERI Musarella, Brullo & Giusso all. nov.

      *Scabioso taygeteae-Onosmetum leptanthae* Quézel 1964

      *Danthoniastro compacti-Fumanetum alpinae* Musarella, Brullo & Giusso ass. nov.

      *Sideritido clandestinae-Astragaletum taygetici* Musarella, Brullo & Giusso ass. nov.

      *Rindero graecae-Acantholimetum graeci* Quézel 1964

      *Onosmo heterophyllae-Astragaletum erinacei* Musarella, Brullo & Giusso ass. nov.

      *Astragaletum lacteo-taygetici* Musarella, Brullo & Giusso ass. nov.

      *Violo parnoniae-Astragaletum erinacei* Musarella, Brullo & Giusso ass. nov.

      *astragaletosum erinacei* Musarella, Brullo & Giusso subass. nov.

      *asperuletosum malevonensis* Musarella, Brullo & Giusso subass. nov.

  NOAEO MUCRONATAE-SILENETALIA URVILLEI Musarella, Brullo & Giusso ord. nov.

    ASPERULION SAMIAE Musarella, Brullo & Giusso all. nov.

      *Astragaletum samii* Musarella, Brullo & Giusso ass. nov.

      *Thymo samii-Astragaletum condensati* Musarella, Brullo & Giusso ass. nov.

      *Campanulo lyratae-Genistetum parnassicae* Musarella, Brullo & Giusso ass. nov.

      *Arenario guicciardii-Seslerietum anatolicae* Musarella, Brullo & Giusso ass. nov.

    FESTUCO PSEUDOSUPINAE-ASTRAGALION AEGEICI Musarella, Brullo & Giusso all. nov.

      *Anthemido discoideae-Astragaletum aegeici* Musarella, Brullo & Giusso ass. nov.

      *Diantho zonati-Astragaletum lesbiaci* Musarella, Brullo & Giusso ass. nov.

      *Galio insularis-Thymetum sypilei* Musarella, Brullo & Giusso ass. nov.

      *Acantholimo aegaei-Astragaletum lesbiaci* Musarella, Brullo & Giusso ass.nov.

    SESLERIO ACHTAROVII-ANTHEMIDION TENUILOBAE Musarella, Brullo & Giusso all.nov.

      *Paronychio bornmuelleri-Astragaletum odoniani* Musarella, Brullo & Giusso ass. nov.

Finally, in order to highlight the phytosociological relantionships among the investigated associations belonging to *Cerastio candidissimi-Astragaletea rumelici*, two synoptical tables regarding the orders *Eryngio multifidi-Armerietalia orphanidis* (Appendix B, Table A4) and *Noaeo mucronatae-Silenetalia urvillei* (Appendix B, Table A5) are provided.

### 2.4. Description of the Vegetation

*CERASTIO CANDIDISSIMI-ASTRAGALETEA RUMELICI* Musarella, Brullo & Giusso cl. nov. *hoc loco*

Syn.: *Daphneeto-Festucetea* Quézel 1964, Vegetatio 12:325, p.p., nom. amb. rejic. propos. (art. 36)

*Daphno oleoidis-Festucetea variae* Quézel 1964, corr. Quézel, Barbero & Akman 1992, Ecol. Medit. 18: 82, p.p., nom. amb. rejic. propos. (art. 36)

Holotypus: *Eryngio multifidi-Armerietalia orphanidis* Musarella, Brullo & Giusso ord. nov. *hoc loco.*

Characteristic species: *Achillea umbellata*, *Alyssum montanum* subsp. *graecum Arenaria guicciardii*, *Asperula boissieri*, *Asperula lutea*, *Asperula thessala*, *Astragalus rumelicus* subsp. *rumelicus*, *Beta nana*, *Campanula radicosa*, *Centaurea pichleri*, *Centaurea raphanina* subsp. *mixta*, *Cerastium candidissimum*, *Crepis fraasii* subsp. *fraasii*, *Dianthus tymphristeus*, *Draba lacaitae*, *Erysimum cephalonicum*, *Erysimum microstylum*, *Erysimum pectinatum*, *Festuca cyllenica* subsp. *cyllenica*, *Festuca polita*, *Fritillaria graeca*, *Fritillaria guicciardii*, *Galium citraceum*, *Galium thymifolium*, *Helianthemum hymettium*, *Herniaria parnassica* subsp. *parnassica*, *Hieracium lazistanum* subsp. *Leithneri*, *Lamium pictum*, *Leontodon graecus*, *Lysimachia serpyllifolia*, *Minuartia confusa*, *Minuartia attica* subsp. *attica*, *Nepeta argolica* subsp. *argolica*, *Paronychia albanica* subsp. *graeca*, *Poa thessala*, *Podospermum canum* var. *alpinum*, *Pterocephalus perennis* subsp. *perennis*, *Scutellaria rupestris* subsp. *parnassica*, *Silene radicosa* subsp. *radicosa*, *Stipa endotricha*, *Teucrium montanum* var. *parnassicum*, *Trinia frigida*, *Trinia guicciardi*, *Trisetum tenuiforme*, *Verbascum epixanthinum* var. *epixanthinum*, *Veronica erinoides*, *V. thymifolia*, *Viola chelmea*, *V. greca*.

Differential species: *Achillea fraasii*, *Achillea holosericea*, *Acinos alpinus* subsp. *meridionalis*, *Aethionema saxatile* subsp. *graecum*, *Anthemis cretica* subsp. *cretica*, *Asyneuma limonifolium*, *Aubrieta deltoidea* var. *deltoidea*, *Aubrieta deltoidea* subsp. *intermedia*, *Bromopsis lacmonica*, *Bromus riparius*, *Campanula spathulata* subsp. *spathulata*, *Carduus tmoleus*, *Carlina frigida*, *Carum graecum* subsp. *graecum*, *Carum meoides*, *Dianthus integer* subsp. *minutiflorus*, *Dianthus viscidus* var. *viscidus*, *Draba lasiocarpa*, *Euphorbia herniariifolia*, *Festuca callieri* subsp. *callieri*, *Festuca jeanpertii* subsp. *jeanpertii*, *Galium incanum* subsp. *incanum*, *Geranium macrostylum*, *Geranium subcaulescens*, *Helictotrichon aetolicum*, *Koeleria mitrushii*, *Linaria peloponnesiaca*, *Linum elegans*, *Minuartia juniperina*, *Minuartia stellata*, *Morina persica*, *Myosotis suaveolens*, *Myosotis sylvatica* subsp. *canea*, *Onobrychis alba* subsp. *pentelica*, *Pimpinella tragium* subsp. *polyclada*, *Pimpinella tragium* subsp. *tragium*, *Ranunculus sartorianus*, *Sedum laconicum*, *Sempervivum marmoreum*, *Sesleria tenerrima*, *Sesleria vaginalis*, *Silene bupleuroides* subsp. *staticifolia*, *Stachys heldreichii*, *Telephium orientale*, *Thymus chaubardii*, *Thymus leucotrichus*, *Tragopogon crocifolius* subsp. *samaritanii*.

Structure and ecology: The class groups pulvinate orophilous plant communities characterized by dominance of dwarf shrubs, often with tragacanthoid habit, sometimes mixed with caespitose hemicryptophytes, which constitute quite spaced grasslands, where numerous geophytes or rosulate hemicryptophytes play a relevant physiognomic role. The stands colonized by these communities are usually represented by more or less rocky windy ridges and cacuminal surfaces usually with undeveloped soils, as well as more o less stabilized screes. These habitats are distributed mainly in the mountains at 1500–3000 m of altitude, with stands characterized by quite rigid environmental conditions. Sometimes, especially in situations of insularity these plant communities occur also at lower altitudes, sometimes up to 1000 m. From the bioclimatic point of view, these communities are distributed prevalently within the supra- and oro-Mediterranean belts, as well as in supra- and oro-temperate belts, often of sub-Mediterranean type. Downwards, they tend to penetrate into meso-Mediterranean belt, especially due to the degradation processes of the woodlands or when the edaphic conditions are particularly critical, as in the case of blocking of the pedogenetic processes. Dynamically, it is a typically orophilous vegetation showing usually a climatophilous role, even if often it is represented by edaphophilous communities. When these communities are localized within the forest belt, they assume a secondary role, being linked usually to processes of woodland degradation. As concerns its floristic arrangement, this vegetation is characterized by a rich set of endemics, often having a relevant taxonomic and phytogeographic significance. Many of them are relict species belonging to Tertiary elements, often represented by groups taxonomically isolated, segregated in a lot of geographical vicariants. Apart from a contingent of endemic taxa, which are proposed as characteristics of this class, other non-strictly endemic species with a wider distribution are considered as “differential species”, since in Greece they are usually localized in this type of orophilous vegetation.

Distribution: According to literature and unpublished personal data, this class has its greater spread on the mountains of mainland Greece, extending northwards to Albania and Macedonia and eastwards in the north-western and western Anatolia, as well as in Euboea and some Ionian Islands. Moreover, altough floristically rather impoverished, it is represented also in some islands of north-eastern and northern Aegean, such as Samos, Chios, Lesbos, Samothraki and Thassos, where high mountains occur.

*ERYNGIO MULTIFIDI-ARMERIETALIA ORPHANIDIS* Musarella, Brullo & Giusso ord. nov. *hoc loco.*

Syn.: *Daphneeto-Festucetalia* Quézel 1964, Vegetatio, 12:325 p.p., nom. amb. rejic. propos. (art. 36).

*Daphno oleoidis-Festucetalia variae* Quézel 1964, Vegetatio, 12:325, corr. Quézel, Barbero & Akman 1992, Ecol. Medit. 18:82, p.p., nom. amb. rejic. propos. (art. 36).

*Acantholimo-Astragaletalia* Voliotis 1973, Sci. Ann. Fac. Phys. Math Univ. Thess. 13:237, p.p., *nom. nud.*

Holotypus: *Sideritido raeseri-Thymion parnassici* Musarella, Brullo & Giusso all. nov. *hoc loco.*

Characteristic species: *Acantholimon graecum*, *Alkanna graeca* subsp. *boetica*, *Allium achaium*, *Allium frigidum*, *Alyssum repens* var. *brachyphyllum*, *Armeria orphanidis*, *Asperula rigidula*, *Astragalus angustifolius* subsp. *erinaceus*, *Astragalus rumelicus* subsp. *taygeticus*, *Avenochloa agropyroides*, *Centaurea affinis* subsp. *laconiae*, *Cirsium hypopsilium*, *Crepis incana*, *Dasypyrum hordeaceum*, *Dianthus androsaceus*, *Dianthus biflorus*, *Draba parnassica*, *Echinops taygeteus*, *Erodium chrysanthum*, *Eryngium multifidum*, *Erysimum asperulum*, *Erysimum pusillum*, *Euphorbia deflexa*, *Festuca janpertii* subsp. *achaica*, *Galium taygeteum*, *Geocaryum parnassicum*, *Geocaryum peloponnesiacum*, *Inula candida* subsp. *limonella*, *Noccaea graeca*, *Paronychia albanica* subsp. *graeca*, *Rindera graeca*, *Scutellaria rupestris* subsp. *rupestris*, *Verbascum acaule*.

Structure and ecology: This order groups the orophilous plant communities, as highlighted in the class, linked mainly to the supra-and oro-temperate belts of sub-Mediterranean type, occurring mainly at above 1700–1800 m of altitude. These plant communities show a climatophilous, or sometimes edaphophilous character, usually are localized in the cacuminal stands of the mountains above the timberline. Within this syntaxon the plant communities distributed also at lower altitudes (1000–1700 m) falling in the meso-and oro-Mediterranean belt can be included. In this case, the vegetation is largely represented by secondary communities, often of edaphophilous type, since linked to degradation processes of the woodlands.

Distribution: On the basis of current knowledge, the order seems to be circumscribed to the mountains of Greece, Peloponnese included, as well as the Ionian Islands (Cephalonia and Lefkas) and Euboea.

*MARRUBIO VELUTINI-THYMION PARNASSICI* Musarella, Brullo & Giusso all. nov. *hoc loco.*

Syn.: *Eryngieto-Bromion* Quézel 1964, Vegetatio, 12:326, p.min.p., nom. amb. rejic. propos. (art. 36).

*Eryngio multifidi-Bromion fibrosi* Quézel 1964, corr. Quézel, Barbero & Akman 1992, Ecol. Medit. 18:82 p.min.p., nom. amb. rejic. propos. (art. 36).

*Astragaleto-Seslerion* Quézel 1964, Vegetatio, 12:326, p.min.p., nom. amb. rejic. propos. (art. 36).

*Astragalo angustifolii-Seslerion coerulantis* Quézel 1964, corr. Quézel, Barbero & Akman 1992, Ecol. Medit. 18:82, p.min.p., nom. amb. rejic. propos. (art. 36).

*Stipeto-Morinion* Quézel 1964, Vegetatio, 12:326, p.min.p, nom. amb. rejic. propos. (art. 36).

*Stipo pulcherrimae-Morinion persicae* Quézel 1964, corr. Quézel, Barbero & Akman 1992, Ecol. Medit. 18:82 p.min.p., nom. amb. rejic. propos. (art. 36).

Holotypus: *Astragalo lactei-Convolvuletum cochlearis* Quézel 1964, *hoc loco.*

Characteristic species: *Alyssum montanum* subsp. *hymettium*, *Centaurea affinis* subsp. *affinis*, *Centaurea affinis* subsp. *pallidior Dianthus viscidus* var. *parnassicus*, *Erigeron glabratus* subsp. *graecus*, *Erysimum parnassi*, *Festuca graeca* subsp. *graeca*, *Galium circae*, *Geocaryum parnassicum*, *Lactuca intricata*, *Linaria parnassica*, *Marrubium velutinum*, *Nepeta parnassica*, *Nepeta spruneri*, *Satureja parnassica*, *Sideritis raeseri* subsp. *raeseri*, *Thymus leucospermus*, *Thymus parnassicus*, *Thymus teucrioides* subsp. *teucrioides*, *Verbascum parnassicum*.

Structure and ecology: Within the order *Eryngio multifidi-Armerietalia orphanidis*, this alliance is that one showing more marked characters of continentality. The associations belonging to this syntaxon seem to have greater floristic structural and ecological correlations with those ones occurring in the northern Greece. Clearly, towards to the north of Greece, the bioclimate becomes markedly more mesic with a progressive decrease of its Mediterranean character. This is reflected quite well in the orophilic pulvinate vegetation, which shows a more marked thermophily in the mountains of southern Greece. Therefore, this syntaxon can be considered as the transition term between the southernmost alliances occurring in the Peloponnese and probably the northernmost ones regarding the mountain ranges of Pindus and Mt. Olympus, which is still to be defined under the phytosociological profile including several associations already defined by Quézel [36]. In particular, the associations falling in the *Marrubio velutini-Thymion parnassici*, while maintaining structurally their prerogatives of shrub-pulvinate community, tend to show a certain increase of the hemicriptophytic component. Further, their floristic settlement increases with elements having more relantionships with taxonomic groups having a more northernmost distribution.

Distribution: The alliance is distributed mainly in the massifs of Sterea Ellas, such as Mt. Parnassus, Mt. Giona, Mt. Vardoussia and Mt. Timfristos, as well as of Attica. Probably, plant communities belonging to this syntaxon occur also in other mountains of this continental area of Greece.

Notes: The *Marrubio velutini-Thymion parnassici* does not show any clear floristic, ecological and chorological correlation with the three alliances described by Quézel [35]. In particular, this new syntaxon is floristically differentiated by endemics distributed in the high-mountain belt of the massifs located exclusively in Sterea Ellas and Attica. In addition, this alliance groups associations that are not linked to a well-defined altitudinal belt, but they are distributed from the lower mountain zones (1200–1300 m) up to the high-mountain ones reaching the altitude of 2500 m.

*Marrubio velutini-Astragaletum rumelici* Quézel 1964, Vegetatio 12:334 (Appendix C, Table A6).

Syn.: Association à *Astragalus creticus* subsp. *rumelicus* et *Marrubium velutinum*, Quézel 1964.

Lectotypus: Table 18, rel. 3, Quézel [35], *hoc loco.*

Characteristic species: *Astragalus rumelicus* subsp. *rumelicus*, *A. hellenicus*, *Nepeta parnassica*.

Structure and ecology: The association is located on calcareous and dolomitic substrata, of more or less rocky steep slopes (30°–40°), characterized by eroded or not very deep soils, rich in coarse skeletal component. It assumes a clear climatophilous role in the supra-temperate sub-Mediterranean belt at an elevation of 1800 and 2100 m, while at lower altitudes (examples were found up to 1500 m) shows a clearly secondary pattern, because its spread is linked to the processes of forest degradation, here represented mainly by *Abies cephalonica* woods. Physiognomically, this association is dominated by thorny cushion-like of *Astragalus rumelicus* subsp. *rumelicus*, which often constitues dense populations. Quite significant it is the occurrence, although scattered, in this vegetation of two interesting endemic species, such as *Nepeta parnassica*, distributed in Mt. Parnassus and Mt. Chelmos (on the latter, however, is quite rare), and *Astragalus hellenicus*, widespread on the mountains of Sterea Ellas. Within this association, as emphatized by Quézel [35], two subassociations linked to different soil conditions can be distinguished. They are cited by that author as subass. *typicum*, localized on carbonatic substrates with no floristic differentiation, and subass. *achilleetosum nobilis* Quézel 1964 (lectotypus rel. 12, Table 18, Quézel [35], *hoc loco*) restricted to sandstone or sometimes schist outcrops, differentiated by *Achillea nobilis* and *Salvia argentea* var. *alpina*.

Distribution: This association is well represented on the southernmost massifs of Sterea Ellas, as Mt. Parnassus, Mt. Giona and Mt. Vardoussia. However, its occurrence also in other mountain massifs of this area can not be excluded.

*Astragalo lactei-Convolvuletum cochlearis* Quézel 1964, Vegetatio 12:339 (Appendix C, Table A7).

Syn.: Association à *Convolvulus cochlearis* et *Astragalus lacteus* Quézel 1964.

Lectotypus: Table 21, rel. 4, Quézel [35], *hoc loco.*

Characteristic species: *Astragalus lacteus*, *Convolvulus cochlearis*, *Koeleria carniolica*.

Structure and ecology: The association is confined to the dolomitic substrates of the ridges that bordered some deep dolines. The surfaces occupied by this association are usually almost flat and are distributed at an altitude of 1650–1800 m, within the supratemperate sub-Mediterranean bioclimatic belt. This vegetation is dominated by small prostrate chamaephytes, among them have a quite significant role *Convolvulus cochlearis* (=*C. parnassicus* Boiss. & Orph.), rather rare Balkan endemic. In this association it occurs also *Astragalus lacteus*, which shows a quite constant frequence, as well as *Asperula rigidula* and *Koeleria carniolica*, which are less frequent.

Distribution: Currently it is known only to the Mt. Parnassus, where it is observed near the refuge of the EOS Gherondovrachos.

Notes: As concerns this association, Quézel [35] highlight that it occupies an intermediate position between the *Astragalo-Seslerion* and *Stipo-Morinion* alliances, because in its floristic settlement are present characteristic species of both syntaxa. However, the author considers more properly to include it in the *Astragalo-Seslerion*, mainly for the occurrence of *Astragalus angustifolius*. That is further evidence of the lack of phytosociological value of the alliances proposed by the author.

*Nepeto epiroticae-Astragaletum corynthiaci* (Quézel 1964) Musarella, Brullo & Giusso nom. nov. (Appendix C, Table A8).

Syn.: Association à *Astragalus cephalonicus* et *Nepeta nuda* Quézel 1964, Vegetatio 12:357.

Lectotypus: Table 30, rel. 2, Quézel [35], *hoc loco.*

Characteristic species: *Astragalus corynthiacus*, *Nepeta nuda* var. *epirotica*.

Structure and ecology: The association is localized on the bottom of dolines and also on slightly inclined surfaces characterized by rather deep silt-clay soils, deposited on carbonate substrata. It is distributed between 1600 and 1900 m of altitude, sometimes reaching 2100 m, having its optimum in the supratemperate sub-Mediterranean belt. Physiognomically, this vegetation is differentiated by the dominance of *Astragalus corynthiacus*, a new species closely related to *A. cephalonicus*, which tends to constitute dense and homogeneous populations. Another quite significant species is *Nepeta nuda* var. *epirotica*, which seems to have its optimum in these stands. Potentially, this association is linked to the erosion processes and washing away of calcareous rocks that accumulate fine particles into the lower parts of dolines and depressions. These surfaces, in extreme conditions, with very deep soils, are ususally colonized by hemicryptophytic communities of *Trifolion parnassi*. In fact, in this association, some elements belonging to the latter alliance and related order, *Trifolietalia parnassi*, are present which clearly have the meaning of transgressiion. In conditions of marked edaphic xericity, such as in the stands with rocky outcrops and superficial soils, the vegetation at issue is replaced by the climatophylous communities of *Marrubio velutini-Astragaletum rumelici*.

Distribution: The association was currently observed only on Mt. Parnassus, where it is represented mainly in the dolines.

Notes: As regards its phytosociological arrangement, this association was described by Quézel [35] as Association à *Astragalus cephalonicus* et *Nepeta nuda* and included into the alliance *Trifolion parnassi*, since the author based on its ecological requirements, being linked to deep soils and on the presence of a fair number of species characteristic of this syntaxon. However, it should be noted that the author considered this association structurally very similar to the communities of *Daphno-Festucetalia*, especially for the dominance of torny cushion-like shrubs, completely absent in the typical grasslands of *Trifolion parnassi*. Moreover, for the presence of a significant settlement of *Daphno-Festucetalia*, he considered this association as intermediate between this order and that of *Trifolietalia parnassi*. In fact, this perplexity of Quézel [35] is here shared by us too, but basing on its floristic and structural characterics, it seems to exclude its possible attribution to *Trifolion parnassi*. It is to underline that on the whole in this association are well represented many species of *Marrubio velutini-Thymion parnassici* and related higher syntaxa. The dominant species was previously identified by Quézel [35] as *Astragalus cephalonicus,* but this attribution was wrong, since it clearly differs from the latter in numerous morphological features and should be treated as a distinct new species named *A. corinthiacus.*

*Nepeto spruneri-Astragaletum corynthiaci* Musarella, Brullo & Giusso ass. nov. *hoc loco* (Appendix C, Table A9).

Holotypus: Appendix C, Table A9, rel. 3, *hoc loco.*

Characteristic species: *Astragalus corynthiacus*, *Nepeta spruneri*.

Structure and ecology: This association can be considered as a geographical vicariant of *Nepeto epiroticae-Astragaletum corynthiaci* previously described from Mt Parnassus. It is also caracterized by the dominance of *Astragalus corynthiacus,* while *Nepeta nuda* var. *epirotica* is replaced by *N. spruneri.* This vegetation shows the same ecology of the above-mentioned association, since it always occurs in the dolines characterized by quite deep soils, usually localized between 1700–1800 m of elevation, sometimes reaching 2000 m. Floristically, it is well differentiated by several species of the alliance and higher ranks, while that ones of *Trifolion parnassi* are very rare.

Distribution: The association was surveyed in some stands of Mt. Giona.

*Thymo parnassici-Paronychietum polygonifoliae* Quézel 1964, Vegetatio 12:341 corr. (Appendix C, Table A10).

Syn.: Association à *Paronychia chionaea* et *Thymus hirsutus* subsp. *ciliato-pubescens* Quézel 1964.

Lectotypus: Table 23, rel. 3, Quézel [35], *hoc loco.*

Characteristic species: *Paronychia polygonifolia* (=*P. chionaea*), *Edraianthus graminifolius* f. *minor*, *Dianthus ventricosus*.

Structure and ecology: This association, characterized by dominance of small chamaephytes showing a prostrate or creeping habit, is localized in correspondence to the very windy ridges, usually over 2000 m of altitude. It is possible to observe this vegetation also at lower altitudes (ca. 1800 m), always in cacuminal stands. From the bioclimatic point of view, this association is well represented in the oro-temperate sub-Mediterranean belt extending downward in the supra-temperate sub-Mediterranean one. The surfaces are rather flat with superficial soils rich in minute skeleton, where, due to the action of the winds, the soil evolution is very slow, and the vegetation always keeps a prostrate habit. According to Quézel [35], this vegetation is dominated by plants showing a small size, such as *Paronychia polygonifolia* (as *P. chionaea*), *Thymus parnassicus* (as *T. hirsutus* subsp. *ciliato-pubescens*), *Edraianthus graminifolius* f. *minor, Dianthus ventricosus.* The pulvinated camaephytes and the cespitose grasses are totally absent. The author distinguished two subassociations linked to altitudinal factors, represented at over 2100 m of altitude by the subass. *typicum*, which is replaced at lower altitudes from subass. *linetosum angustifolii* Quézel 1964 (lectotypus: Table 23 rel. 5, *hoc loco*). Floristically, the first subassociation is differentiated by *Euphrasia salisburgensis*, *Minuartia condensata, Festuca halleri* subsp. *riloensis, Carex kitaibeliana*, and *Galium plebeium,* while the second one has as differential species *Linum tenuifolium* and *Ptilotrichum rupestre.*

Distribution: The association seems to be exclusive of Mt. Giona, where it is very frequent.

*Nepeto sprunerii-Astragaletum tymphrestei* Musarella, Brullo & Giusso ass. nov. *hoc loco* (Appendix C, Table A11).

Holotypus: Appendix C, Table A11, rel. 1, *hoc loco.*

Characteristic species: *Astragalus tymphresteus*.

Structure and ecology: The association was observed in stands at altitudes between 1200 and 1400 m, on slightly inclinated slopes characterized by carbonate rocks within the meso-Mediterranean bioclimatic belt. The soils are poorly developed with many minute skeletons. This vegetation is dominated by *Astragalus thymphresteus*, thorny dwarf shrub growing with other small chamaephytes, such as *Nepeta spruneri*, *Thymus chaubardii*, *Chamaecytisus hirsutus*, and some cespitose hemicryptophytes.

Distribution: The association was found only on Mt. Giona, where it is circumscribed to stands of lower altitudes, but it probably occurs also in other mountains.

*Violo stojanowii-Seslerietum vaginalis* Quézel 1973, Biol. Gallo-Hellen. 5(1):152, corr. (Appendix C, Table A12).

Syn.: Association à *Sesleria coerulans* et *Viola stojanowii* Quézel 1973.

Lectotypus: Table 3, rel. 11, Quézel [38], *hoc loco.*

Characteristic species: *Viola stojanowii*, *Thymus teucrioides* subsp. *teucrioides*, *Thymus striatus*.

Structure and ecology: The association occurs over 2200 m of altitude, where is localized in the small depressions among the cacuminal rocky peaks, where very minute clasts are accumulated and covered by soils rich in clay subject to solifluction. In these stands characterized by an acclivity of 20–30%, the vegetation shows a rather sparse coverage in which *Sesleria vaginalis* (=*S. coerulans*) plays an important role. On the whole, it is a floristically quite poor herbaceous vegetation, where *Viola stojanowii* is physiognomically significant. Usually, this association takes catenal contacts with the scree vegetation belonging to *Drypetalia spinosae*.

Distribution: This vegetation was described by Quézel [38] for Mt. Vardoussia, but probably it occurs also in other mountains of Sterea Ellas.

*Erysimo parnassi-Minuartietum stellatae* Quézel 1964, Vegetatio 12:340 (Appendix C, Table A13).

Syn.: Association à *Minuartia stellata* et *Erysimum pusillum* subsp. *parnassi* Quézel 1964.

Lectotypus: Table 22, rel. 3, Quézel [35], *hoc loco.*

Characteristic species: *Minuartia stellata*, *Astragalus apollineus*, *Anthemis spruneri*, *Allium parnassicum*, *Anthemis tinctoria* var. *parnassica*, *Erigeron alpinus*.

Structure and ecology: The association colonizes the rocky outcrops and the stabilized screes at altitudes over 2100 m, within the oro-temperate sub-Mediterranean bioclimatic belt. It is frequent on the prevalently rocky surfaces that, due to the considerable acclivity, the soils are very superficial, accumulating mainly among the rocky crevices and into the bushes. Physiognomically, it is distinguished by the dominance of compact and often voluminous cushion-like shrubs of *Minuartia stellata*, that usually grows togheter with *Sesleria vaginalis* and several species with prostrate habit. The characteristic species of the alliance *Marrubio velutini-Thymion parnassici are well represented*, among them *Erysimum parnassi*, *Marrubium velutinum*, *Satureja parnassica*, which show high coverage value. Within this association Quézel [35] distinguished two subassociations on phytogeographical base, represented by *saturejetosum parnassicae* (=subass. *teucrioides* à *Thymus*), restricted to Mt. Parnassus, and by *aurinietosum giónae* (=subass. *kionae* à *Alyssum*) for Mt. Giona. The first one corresponds clearly to the type, while the second one must be treated as a distinct association, well differentiated from floristically, also from chorological point of view, named as *Aurinio gionae-Minuartietum stellatae*.

Distribution: Actually, this vegetation is distributed only on Mt. Parnassus.

*Aurinio gionae-Minuartietum stellatae* Musarella, Brullo & Giusso ass. nov. *hoc loco* (Appendix C, Table A14).

Syn.: Association à Minuartia stellata et Erysimum pusillum subsp. parnassi subass. à Alyssum kionae Quézel 1964.

Holotypus: Table 22, rel. 3, Quézel [35], *hoc loco.*

Characteristic species: *Minuartia stellata*, *Aurinia gionae*.

Structure and ecology: From the ecological point of view, the association is very similar to *Erysimo parnassi-Minuartietum stellatae*. In fact, it occurs at altitudes between 2100 and 2450 m, on calcareous substrata, more or less acclive, showing a coverage which not exceeding 70%. Floristically the vegetation differs markedly from the *Erysimo parnassi-Minuartietum stellatae*, for the almost total absence of *Erysimum parnassi*, *Satureja parnassica*, *Sesleria vaginalis*, all species that in the latter association are fairly common and often dominant. In addition to the absence of all characteristic species, the association at issue differs from the previous one also for the occurrence of the endemic *Aurinia gionae*. The only common element between the two communities is the dominance of *Minuartia stellata*.

Distribution: The association is esclusive of some places of Mt. Giona, where it is quite frequent.

*Achilleo fraisii-Dianthetum tymphrestei* Musarella, Brullo & Giusso ass. nov. *hoc loco* (Appendix C, Table A15).

Holotypus: Appendix C, Table A15, rel. 5, *hoc loco.*

Characteristic species: *Dianthus tymphresteus*, *Valeriana bertiscea*.

Structure and ecology: The association is localized on small rocky summits, in the more or less flat places characterized by minute crumbly limestone mixed with a little soil. It has been observed at altitudes of 1700–1800 m of very windy stands, within the supra-temperate sub-Mediterranean belt. Floristically, it is differentiated by the dominance of small pulvinate shrubs of *Dianthus tymphresteus*, which grows together other cespitose hemicryptophytes and small prostrate chamaephytes, such as *Centaurea affinis* subsp. *affinis*, *Achillea fraisii*, *Koeleria mitrushi*, *Festuca jeanpertii* subsp. *jeanpertii*, *Astragalus lacteus*, etc.

Distribution: This association was surveyed on Mt. Giona at Liritsa, but it probably occurs also in other neighbouring massifs, such as Vardoussia and Timphristos.

*Asperulo luteae-Achilleetum umbellatae* Musarella, Brullo & Giusso ass. nov. *hoc loco* (Appendix C, Table A16).

Holotypus: Appendix C, Table A16, rel. 3, *hoc loco.*

Characteristic species: *Achillea umbellata*, *Carex caryophyllea*.

Structure and ecology: The association colonizes the slopes often rather inclinated with fresh solis, mixed to big size clasts, at altitudes of 1700–1800 m, within the suprat-emperate sub-Mediterranean belt. The surfaces occupied by this vegetation are usually South-facing and are frequent at the base of small rocky ridges. In such habitats, several hemicryptophytes such as *Achillea umbellata*, *Carex caryophyllea*, *Asperula lutea*, *Festuca cyllenica* subsp. *cyllenica, Stipa endotricha*, *Koeleria mitrushi* and *Festuca jeanpertii* subsp. *jeanpertii* occur and thrive.

Distribution: This vegetation was surveyed only on Mt. Giona, near Liritsa, where it is very circumscribed.

*Astragalo lactei-Asperuletum apiculatae* Musarella, Brullo & Giusso ass. nov. *hoc hoco* (Appendix C, Table A17).

Holotypus: Appendix C, Table A17, rel. 2, *hoc loco.*

Characteristic species: *Asperula purpurea* subsp. *apiculata*, *Astragalus lacteus*.

Structure and ecology: The association seems exclusive of the calcareous rocky ridges at altitudes between 1500 and 1600 m, where it is linked to slopes with very variable inclination (30–80°), with S-SO exposure. From the biolimatic point of view, it falls between the meso-Mediterranean and supra temperate sub-Mediterranean belts. The vegetation is localized along the large cracks of the rock and is characterized by small chamaephytes and hemicryptophytes. Among them, *Asperula purpurea* subsp. *apiculata, Astragalus lacteus*, *Achillea holosericea* and *Thymus chaubardii* are dominant togheter with various grasses.

Distribution: The association was observed on Mt. Giona at Mavrikorfi, near Proni, where seems quite localized.

*Diantho minutiflori-Festucetum cyllenicae* Musarella, Brullo & Giusso ass. nov. *hoc loco* (Appendix C, Table A18).

Holotypus: Appendix C, Table A18, rel. 5, *hoc loco.*

Characteristic species: *Dianthus integer* subsp. *minutiflorus*, *Festuca cyllenica* subsp. *cyllenica*, *Silene roemeri* subsp. *macrocarpa*.

Structure and ecology: The association colonizes the more or less stabilized screes with an inclination of 20–30°, at an altitude of around 2000 m. It is found in the orotemperate sub-Mediterranean belt, penetrating downward in the sub-Mediterranean supra.temperate one. Physiognomically, it is differentiated by the dominance of large tuffs of *Festuca cyllenica* subsp. *cyllenica*, often associated with *Sesleria vaginalis*. In particular, this community is characterized by *Dianthus integer* subsp. *minutiflorus* and *Silene roemeri* subsp. *macrocarpa*. Moreover, *Satureja parnassica, Nepeta spruneri, Galium thymifolium, Campanula spathulata* subsp. *spathulata*, and *Ranunculus brevifolius* are very frequent.

Distribution: The association was surveyed on Mt. Giona at Amfissa, near Pirghakia.

*Scabioso ochroleucae-Sideridetum raeseri* Musarella, Brullo & Giusso ass. nov. *hoc loco* (Appendix C, Table A19).

Holotypus: Appendix C, Table A19, rel. 4, *hoc loco.*

Characteristic species: *Scabiosa ochroleuca*, *Sideritis raeseri* subsp. *raeseri*, *Vincetoxicum hirundinaria* subsp. *nivale*.

Structure and ecology: This association replaces the *Diantho minutiflori-Festucetum cyllenicae* in the stabilzed screes or, anyway, on the surfaces more compact and richer in soil. Physiognomically, it is differentiated by the dominance of suffruticous shrubs, mainly chamaephytes, such as *Scabiosa ochroleuca, Sideritis raeseri* subsp. *raeseri, Vincetoxicum hirundinaria* subsp. *nivale*, *Satureja parnassica*, *Marrubium velutinum*, *Asperula lutea*, *Centaurea affinis* subsp. *affinis*, *Nepeta spruneri*, etc., while decrease the coverage of the caespitose hemicryptophytes.

Distribution: The association was surveyed on Mt. Giona, in the same place where the *Diantho minutiflori-Festucetum cyllenicae* occurs.

*Ranunculo psilostachydis-Festucetum cyllenicae* Musarella, Brullo & Giusso ass. nov. *hoc loco* (Appendix C, Table A20).

Holotypus: Appendix C, Table A20, rel. 3, *hoc loco.*

Characteristic species: *Festuca cyllenica* subsp. *cyllenica*, *Laserpitium pseudomeum*, *Ranunculus psilostachys*.

Structure and ecology: This association replaces the *Diantho minutiflori-Festucetum cyllenicae* on the more or less stabilized screes localized at lower altitudes (1700–1750 m) in quite fresh and sheltered stands. Particularly, it is frequent in the supra-temperate sub-Mediterranean belt, on surfaces having an inclination of 25–35°. Physiognomically, this vegetation is dominated by *Festuca cyllenica* subsp. *cyllenica*, but in comparison with the prevoius association, in its floristic settlement, a marked decrease of the more orophilous species is observable. Nevertheless, it is well differentiated due to the occurrence of *Ranunculus psilostachys*, *Laserpitium pseudomeum*, *Galium circae*, *Avenochloa agropyroides*, *Trisetum tenuiforme*, etc., species linked to stands of lower altitudes.

Distribution: As the two previous associations, this vegetation was surveyed in the same area of Mt. Giona, but at lower altitudes.

*Edraiantho parnassici-Globularietum cordifoliae* Musarella, Brullo & Giusso ass. nov. *hoc loco* (Appendix C, Table A21).

Holotypus: Appendix C, Table A21, rel. 2, *hoc loco.*

Characteristic species: *Globularia cordifolia*, *Anthyllis montana* subsp. *jacquinii*, *Edraianthus parnassicus*, *Silene auricolata*.

Structure and ecology: The association is localized in rocky places, generally more or less flat or, however, a little sloped. It shows a wide altimetric range, ranging at altitudes from 1700 to 2150 m, thus affecting the supratemperate and orotemperate sub-Mediterranean bioclimatic belts. It is can be considered as a semi-rupestrian community characterized by prostrate or creeping chamaephytes, such as *Globularia cordifolia, Anthyllis montana* subsp. *jacquinii, Edraianthus parnassicus, Silene auricolata*, which grow togheter with other small pulvinate shrubs, among them *Paronychia polygonifolia, Satureja parnassica, Thymus leucotrichus,* etc.

Distribution: The association is spread on some mountain places of Mt. Giona where, usually, it is localized on small surfaces.

*Thymo parnassici-Astragaletum parnassi* Musarella, Brullo & Giusso ass. nov. *hoc loco* (Appendix C, Table A22).

Holotypus: Appendix C, Table A22, rel. 4, *hoc loco.*

Characteristic species: *Astragalus parnassi*.

Structure and ecology: This association is characterized by the dominance of thorny cushion-like shrubs of *Astragalus parnassi*. This species is linked to very lower altitudes (1000–1300 m) within the meso-Mediterranean bioclimatic belt, characterizing one of the most termophilous communities of the *Marrubio velutini-Thymion parnassici*. However, the characteristic species of this alliance and the higher syntaxa, are here well represented, among them *Thymus parnassicus*, *Erysimum parnassi*, *Festuca graeca* subsp. *graeca*, *Astragalus angustifolius* subsp. *erinaceus*, *Asperula lutea*, etc. The vegetation is usually localized in quite fresh and sheltered stands, represented mainly by clearing within the *Abies cephalonica* woodlands. It colonizes the more or less flat surfaces, showing high coverage values.

Distribution: The association is spread in the southern slopes of Mt. Parnassus.

*Chamaecytiso hirsuti-Astragaletum parnassi* Musarella, Brullo & Giusso ass. nov. *hoc loco* (Appendix C, Table A23).

Holotypus: Appendix C, Table A23, rel. 6, *hoc loco.*

Characteristic species: *Astragalus parnassi*, *Chamaecytisus hirsutus*.

Structure and ecology: This association must be considered as a geographic vicariant of the *Thymo parnassici-Astragaletum parnassi***.** In fact, it occurs on Mt. Giona, where it is localized in habitats very similar to that one occupied by the aforesaid association. This vegetation is always characterized by the dominance of *Astragalus parnassi* and is ditributed at an altitude of 1250–1500 m, in little inclinate stands localized within the *Abies cephalonica* woodlands. Floristically, it is characterized by the occurrence of *Chamaecytisus hirsutus* that forms large creeping cushion-like shrubs, while totally absent are several species of the related alliance, frequent though in the previous association.

Distribution: The association occurs in various localities of Mt. Giona.

*Onobrychido pentelicae-Genistetum parnassicae* Musarella, Brullo & Giusso ass. nov. *hoc loco* (Appendix C, Table A24).

Holotypus: Appendix C, Table A24, rel. 5, *hoc loco.*

Characteristic species: *Genista parnassica*, *Onobrychis alba* subsp. *pentelica*.

Structure and ecology: The association replaces the *Thymo parnassici-Astragaletum parnassici* on the slopes with northern exposure of the southern part of Mt. Parnassus. This vegetation is very circumscribed and linked to a little inclinated escarpments with very deep and fresh soils at an altitude of 1100–1200 m. It is localized wihin the meso-temperate bioclimatic belt and is characterized by the dominance of the rare *Genista parnassica* that usually forms large thorny cushion-like shrubs, aften growing with *Astragalus angustifolius* subsp. *erinaceus* and, occasionally, with *Astragalus rumelicus* subsp. *rumelicus* and *A. parnassi*. Differential species of this association is *Onobrychis alba* subsp. *pentelica*, while as concerns the species of the higher syntaxa are well represented.

Distribution: The association seems circumscribed to a very narrow area of the southern part of Mt. Parnassus.

*Allio cithaeronis-Dianthetum serratifolii* Musarella, Brullo & Giusso ass. nov. *hoc loco* (Appendix C, Table A25).

Holotypus: Appendix C, Table A25, rel. 1, *hoc loco.*

Characteristic species: *Allium cithaeronis*, *Dianthus serratifolius* subsp. *serratifolius*, *Petrorhagia armerioides*, *Paronychia macedonica*, *Scabiosa ochroleuca*.

Structure and ecology: The association is circumscribed at the cacuminal calcareous plateau of Mt. Kitheronas, at an altitude of 1350–1400 m. It is a very windy place, subjected, unfortunately, to overgrazing, falling in the meso-Mediterranean bioclimatic belt. Physiognomically, it is differentiated by the occurrence of some small chamaephytes as *Dianthus serratifolius* subsp. *serratifolius, Petrorhagia armerioides, Paronychia macedonica,* and *Scabiosa ochroleuca*, growing togheter with the endemic *Allium cithaeronis*.

Distribution: The association is exclusive of Mt. Kitheronas (Sterea Hellas).

*Inulo methaneae-Sideritetum atticae* Musarella, Brullo & Giusso ass. nov. *hoc loco* (Appendix C, Table A26).

Holotypus: Appendix C, Table A26, rel. 5, *hoc loco.*

Characteristic species: *Inula verbascifolia* subsp. *methanea*, *Sideritis raeseri* subsp. *attica*, *Aethionema saxatile* subsp. *graecum*.

Structure and ecology: The association occurs on the calcareous slopes of Mt. Parnis at an altitude of 1150–1300 m, within an area characterized by a meso-Mediterranean bioclimate and, particularly, affected by a regime of dense fog. It is localized on flat or a little inclinated surfaces with variable exposure. Physiognomically, it is differentiated by the occurrence and often dominace of small shrubs, as *Inula verbascifolia* subsp. *methanea, Sideritis raeseri* subsp. *attica, Aethionema saxatile* subsp. *graecum*, *Alyssum montanum* subsp. *hymettium*, *Achillea holosericea*, etc.

Distribution: The association was surveyed only in cacuminal stands of Mt. Parnis near Athens.

*ASTRAGALION CEPHALONICI* Musarella, Brullo & Giusso all. nov. *hoc loco.*

Holotypus: *Scutellario cephalonicae-Astragaletum cephalonici Musarella*, Brullo & Giusso ass. nov. *hoc loco.*

Characteristic species: *Astragalus cephalonicus*, *Centaurea subciliaris* subsp. *subciliaris*, *Thymus holosericeus*, *Petrorhagia fasciculata* var. *cephallenica*, *Scutellaria rupestris* subsp. *cephalonica*.

Structure and ecology: The alliance replaces in the Ionian islands of Cephalonia and Lefkada the *Marrubio velutini-Thymion parnassici* distributed in Sterea Ellas and Attica. The syntaxon at issue is well differentiated from the previous alliance for some floristic and ecological peculiarity due to its geographical isolation. Floristically, it is also differentiated by some insular endemics exclusive of Cephalonia and Lefkada, taxonomically quite significant, such as *Astragalus cephalonicus*, *Centaurea subciliaris* subsp. *subciliaris*, *Thymus holosericeus*, *Scutellaria rupestris* subsp. *cephalonica*, and *Petrorhagia fasciculata* var. *cephallenica*. The communities belonging to this alliance are localized on the top of isolated mountain summits at altitudes between 800 and 1400 m, which are markedly affected by moist marine winds.

Distribution: The alliance seems circumscribed to the Ionian Islands of Cephalonia and Lefkada.

*Helictotricho convoluti-Thymetum holosericei* Musarella, Brullo & Giusso ass. nov. *hoc loco* (Appendix C, Table A27, rel. 1–5).

Holotypus: Appendix C, Table A27, rel. 3, *hoc loco.*

Characteristic species: *Helictotrichon convolutum* subsp. *convolutum*, *Ononis pusilla*, *Allium lefkadensis*, *Aurinia saxatilis* subsp. *saxatilis*, *Erysimum linearifolium*.

Structure and ecology: The association is localized on the cacuminal plateau more or less windy, characterized by very rocky calcareous substrata with immature soils. This vegetation has its optimum at 800–1000 m of altitude, within the upper meso-Mediterranean belt. Floristically, it is differentiated by the dominance of the endemic *Thymus holosericeus* which grows togheter with the tuffs of *Helictotrichon convolutum* subsp. *convolutum,* an Est-Mediterranean species, and the endemic *Allium lefkadensis*. In this association occurs also *Astragalus cephalonicus* which was already recorded in this mountain by Hofmann [85].

Distribution: This association is localized in the Lefkas Island in small places on Mt. Elati (Stravoti).

*Saturejo cuneifoliae-Thymetum holosericei* Musarella, Brullo & Giusso ass. nov. *hoc loco* (Appendix C, Table A27, rel. 6–9).

Holotypus: Appendix C, Table A27, rel. 6, *hoc loco.*

Characteristic species: *Allium cephalonicum*, *Centaurea spruneri* subsp. *guicciardi*, *Satureja cuneifolia*.

Structure and ecology: The association, ecologically very similar to the previous one, occurs on calcareous rocky outcrops at 800–1000 m of altitude in the Cephalonia Island. Floristically, it is differentiated from the previous one for the lack of *Helictotrichon convolutum* subsp. *convolutum*, while *Satureja cuneifolia* is frequent, which togheter with *Thymus holosericeus* and *Astragalus cephalonicus,* characterizes this cushion-like prostrate vegetation. Moreiover, the occurrence of *Allium cephalonicum* in this vegetation is significant, as a very rare and isolated endemic species, closely related to *A. callidictyon* C. A. Meyer ex Kunth [86].

Distribution: It is a geographical vicariant of the previous association in Cephalonia Island where it is localized on Mt. Ainos and Mt. Roudhi in open and windy places.

*Scutellario cephalonicae-Astragaletum cephalonici* Musarella, Brullo & Giusso ass. nov. *hoc loco* (Appendix C, Table A27, rel. 10–12).

Holotypus: Appendix C, Table A27, rel. 12, *hoc loco.*

Characteristic species: *Astragalus cephalonicus*, *Galium ionicum*, *Erysimum cephalonicum*.

Structure and ecology: This association replaces the previous one in the higher stands at altitudes between 1200 and 1400 m, where it is localized in more or less sloping stands characterized by calcareous rocky substrata. Floristically, it is differentiated from the previous association for the dominace of *Astragalus cephalonicus* which grows togheter with other endemisms as *Erysimum cephalonicum* and *Scutellaria rupestris* subsp. *cephalonica*. This vegetation is localized within supra-Mediterranean bioclimatic belt in the clearing of the *Abies cephalonica* woodlands that occur in the surfaces with more deep and mature soils.

Distribution: The association occuring in the Cephalonia Island, replaces at higher altitudes the *Saturejo cuneifoliae-Thymetum holosericei*.

*Paronychio graecae-Astragaletum erinacei* Musarella, Brullo & Giusso ass. nov. *hoc loco* (Appendix C, Table A27, rel. 13–19).

Holotypus: Appendix C, Table A27, rel. 15, *hoc loco.*

Characteristic species: *Astragalus angustifolius* subsp. *erinaceus*, *Paronychia albanica* subsp. *graeca*, *Galium circae*, *Trinia glauca* subsp. *pindica*, *Aubrieta deltoidea*, *Viola cephalonica*, *Astragalus depressus* subsp. *depressus*, *Verbascum guicciardii*.

Structure and ecology: This association is localized in cacuminal open stands at an altitude of 1600 m, colonizing the calcareous rocks of southern slopes usually quite inclined. These surfaces are strongly affected by winds and daily thermic changes, also subject to long periods of snow cover, with very superficial and eroded soils. Physiognomically it is characterized by small and flattened pulvines of *Astragalus angustifolius* subsp. *erinaceus,* growing together with other dwarf orophytes with chamaephytic or hemicryptophytic habit, some of them endemic, such as *Paronychia albanica* subsp. *graeca, Galium circae, Viola cephalonica, Scutellaria rupestris* subsp. *cephalonica,* etc. This vegetation occurs within supra-Mediterranean bioclimatic belt, which is replaced in the northern slopes with not eroded and mature soils by *Abies cephalonica* woodlands.

Distribution: The association is exclusive of Cephalonia Island it only occurs in the top of Mount Ainos.

*ASTRAGALION EUBOICI* Musarella, Brullo & Giusso all. nov. *hoc loco.*

Holotypus: *Sideritido euboeae-Astragaletum euboici* Musarella, Brullo & Giusso ass. nov., *hoc loco.*

Characteristic species: *Astragalus rumelicus* subsp. *euboicus*, *Asperula suffruticosa*, *Hieracium pannosum* subsp. *euboeum*, *Nepeta dirphya*, *Paronychia euboaea*, *Sideritis euboea*, *Verbascum delphicum*.

Structure and ecology: The alliance can be considered a geographical vicariant on the Euboea mountains of the *Marrubio velutini-Thymion parnassici* distributed in the continental Central Greece. It is differentiated from the latter alliance for its floristic peculiarities (represented by several endemics), linked to geographical isolation due to its insularity. The plant communities belonging to this syntaxon are surveyed at altitudes between 1000 and 1700 m on prevalently carbonatic substrata.

Distribution: The alliance is circumscribed to the Euboea Island in the Central Egean Sea.

*Sideritido euboeae-Astragaletum euboici* Musarella, Brullo & Giusso ass. nov. *hoc loco* (Appendix C, Table A28).

Holotypus: Appendix C, Table A28, rel. 4, *hoc loco.*

Characteristic species: *Astragalus rumelicus* subsp. *euboicus*, *Cytisus supinus*.

Structure and ecology: The association is localized on the carbonatic rocky outcrops at 1100–1200 m of altitude, occasionally reaching 1350 m. The surfaces colonized by this vegetation are more or less inclinate and represented by sunny slopes. The vegetation is dominated by pulvinate shrubs of *Astragalus rumelicus* subsp. *euboicus*, which covers also very large surfaces. Other shrubs are also very frequent, such as *Cytisus supinus*, *Sideritis euboea*, *Inula candida* subsp. *limonella* and *Nepeta dirphya*, species quite important from the physiognomical point of view.

Distribution. The association was surveyed on Mt. Dirfis in the Euboea Island.

*Scabioso webbianae-Phlomidetum samiae* Musarella, Brullo & Giusso ass. nov. *hoc loco* (Appendix C, Table A29).

Holotypus: Appendix C, Table A29, rel. 3, *hoc loco.*

Characteristic species: *Phlomis samia*, *Scabiosa webbiana*, *Viola euboaea*, *Helleborus cyclophyllus*.

Structure and ecology: The association is circumscribed to the fresh depressions with more deep soils and rich in humus, localized at 1000–1100 m of altitude. Quite significant it is here the occurrence of some mesophilous species with herbaceous habit, such as: *Phlomis samia*, *Scabiosa webbiana, Viola euboaea* and *Helleborus cyclophyllus*.

Distribution: The association was surveyed on Mt. Dirfis in the Euboea Island.

*Sideritido euboeae-Festucetum cyllenicae* Musarella, Brullo & Giusso ass. nov. *hoc loco* (Appendix C, Table A30).

Holotypus: Appendix C, Table A30, rel. 1, *hoc loco.*

Characteristic species: *Festuca cyllenica* subsp. *cyllenica*, *Sideritis euboea*, *Bolanthus graecus*, *Carum graecum* subsp. *graecum*, *Arenaria filicaulis* subsp. *euboica*.

Structure and ecology: The cacuminal stands at altitudes over 1550 m are colonized by a herbaceous perennial vegetation dominated by *Festuca cyllenica* subsp. *cyllenica*. Usually, this species colonizes the stony soils and the consolidated screes, adapting well to long periods of snow cover. The association is well differentiated from the other communities characterized by the dominace of *Festuca cyllenica* subsp. *cyllenica*, distributed in the mountains of continental Greece, due to the occurrence of rare orophytes, some endemic of Euboea, such as *Sideritis euboea*.

Distribution: The association was surveyed on Mt. Dirfis in the Euboea Island.

*Inulo limonellae-Seslerietum vaginalis* Musarella, Brullo & Giusso ass. nov. *hoc loco* (Appendix C, Table A31).

Holotypus: Appendix C, Table A31, rel. 8, *hoc loco.*

Characteristic species: *Sesleria vaginalis*, *Inula candida* subsp. *limonella*.

Structure and ecology: The association covers the very inclinate southern rocky slopes of the calcareous summits at 1150–1500 m of altitude. Physiognomically, it is characterized by the dominace, with high coverage values, of *Sesleria vaginalis*, which grows with small shrubs of *Inula candida* subsp. *limonella*, *Sideritis euboea* and *Astragalus rumelicus* subsp. *euboicus*. The association replaces the *Sideritido euboeae-Astragaletum euboici* in the stands at altitudes over 1150 m of very opened and windy slopes.

Distribution: The association was surveyed on Mt. Dirfis in the Euboea Island.

*FESTUCO ACHAICAE-MARRUBION CYLLENEI* Musarella, Brullo & Giusso all. nov. *hoc loco*

Syn.: *Eryngieto-Bromion* Quézel 1964, Vegetatio, 12:326, p.min.p., nom. ambig. rejic. propos. (art. 36).

*Eryngio multifidi-Bromion fibrosi* Quézel 1964, corr. Quézel, Barbero & Akman 1992, Ecol. Medit. 18:82 p.min.p.nom. ambig. rejic. propos. (art. 36).

*Astragaleto-Seslerion* Quézel 1964, Vegetatio, 12:326, p.min.p., nom. ambig. rejic. propos. (art. 36).

*Astragalo angustifolii-Seslerion coerulantis* Quézel 1964, corr. Quézel, Barbero & Akman 1992, Ecol. Medit. 18:82, p.min.p., nom. ambig. rejic. propos. (art. 36).

*Stipeto-Morinion* Quézel 1964, Vegetatio, 12:326, p.min.p., nom ambig. rejic. propos. (art. 36).

*Stipo pulcherrimae-Morinion persicae* Quézel 1964, corr. Quézel, Barbero & Akman 1992, Ecol. Medit. 18:82 p.min.p., nom. ambig. rejic. propos. (art. 36).

Holotypus: *Festuco achaicae-Minuartietum stellatae* Musarella, Brullo & Giusso ass. nov. *hoc loco.*

Characteristic species: *Aster cylleneus*, *Astragalus calavrytensis*, *A. cylleneus*, *Festuca jeanpertii* subsp. *achaica*, *Globularia stygia*, *Marrubium cylleneum*, *Onobrychis montana* subsp. *macrocarpa*, *Sideritis clandestina* subsp. *peloponnesiaca*, *Taraxacum cylleneum*, *Verbascum cylleneum*.

Structure and ecology: This alliance represents the southern geographical vicarious of *Marrubio velutini-Thymion parnassici*, grouping, similarly to the latter, orophilous plant communities structurally characterized by the dominance of chamaephytes and pulvinate nanophanerophytes, sometimes mixed with caespitose hemicryptophytes. Particularly, they differ from those ones occurring in the mountains of Sterea Ellas, apart from the occurrence of a rich set of endemics, also for their ecological requirements. In fact, these communities are subject to climatic conditions characterized by a a more marked thermophily, with higher average annual temperatures and drier rainfall regime, especially in summer. This area falls mainly in the supra-and oro-temperate of sub-Mediterranean type. Moreover, from the phytogeographical point of view, it is possible observe a strong increase of species belonging to taxonomic groups showing a more southern origin.

Distribution: The alliance is distributed in the mountains of northern Peloponnese (Mt. Erimanthos, Mt. Panachaiko, Mt. Chelmos, Mt. Klokos, Mt. Killini and Mt. Menalon).

Notes: The *Festuco achaicae-Marrubion cyllenei* has a strictly phytogeographical characterization, since it is floristically differentiated by species confined to the mountains of Achaia, Corinthia and North Arcadia. It groups plant communities occurring in high mountain stands at altitudes from 1200 to 2400 m. This alliance groups, in addition to several new associations, also other ones described by Quézel [35], Quézel and Katrabassa [40], Georgiadis and Dimopoulos [42], Maroulis and Georgiadis [44], which previously were attributed by these authors in the alliances *Stipo-Morinion*, *Eryngio-Bromion* and *Astragalus-Seslerion*.

Cirsio hypopsilii-Astragaletum taygetici Quézel 1964 corr. (Table A32)

Syn.: Association à *Astragalus cylleneus* et *Cirsium cylleneum* Quézel 1964, Vegetatio 12:332.

*Astracantho thracicae-Marrubietum cyllenei* Georgiadis & Dimopoulos 1993 Bot. Helv. 103:153, nom. inval. (art. 3 c, 5)

*Marrubio cyllenei-Astragaletum rumelici* Maroulis & Georgiadis 2005, Fitosociologia 42(1): 43, nom. illeg. (art. 22,23); Holotypus: Table 2, rel. 460, Maroulis & Georgiadis [44].

Lectotypus: Table 17, rel. 1, Quézel [35], *hoc loco.*

Characteristic species: *Astragalus rumelicus* subsp. *taygeticus*, *Cirsium hypopsilium*.

Structure and ecology: The association is localized on the slopes more or less inclined with variable exposure, characterized by carbonatic stony substrata with rocky outcrops. The soils are enough evolved, but with a significant component of coarse skeleton. It is widespread at altitudes from 1400 to 2000 m, within the supra-temperate sub-Mediterranean bioclimatic belt, with penetrations upwoard in the oro-temperate submediterranean belt and downward in the meso.Mediterranean one. In fact, examples of this vegetation can be observed up to 2150 m of altitude in places well exposed and sunny, as well as at relative low altitudes (1150 m), limited to cacuminal and very rocky windy stands. Physiognomically, this association is differentiated by large thorny pulvinate individuals, often quite raised from the ground, of *Astragalus rumelicus* subsp. *taygeticus,* that in the Peloponnese replaces the subsp. *rumelicus*, distributed in the central and northern Greece [87]. Previously, the populations of this *Astragalus* occurring in the M. Killini were identified by Quézel [35] and Georgiadis and Dimopoulos [42] as *Astragalus cylleneus*, quite rare species on this massif, where it is localized in habitat totally different from those ones normally occupied by the association in question. As regards the floristic composition of this pulvinate vegetation, it is observed a rich contingent of characteristic species of the alliance, as well as of higher syntaxa. It assumes usually a climatophilous role especially at altitudes over 1700–1800 m, while at lower altitudes can be considered as an edaphophilous vegetation, limitedly to cacuminal more rocky stands. Within the climatophilous belt relative to *Abies cephalonica* woodlands, the association represents usually a substitution aspect, due to degradation of this forest.

Distribution: The association is widespread and well represented in the various mountains of the northern Peloponnese, as Mt. Erimanthos, Mt. Panachaiko, Mt. Klokos and Mt. Killini, where it tends to occupy large surfaces.

Notes: This association was originally described by Quézel [35] for Mt. Killini as ass. à *Astragalus cylleneus* et *Cirsium cylleneum* and successively redescribed by Georgiadis and Dimopoulos [42], but changing its name in *Astracantho thracicae-Marrubietum cyllenei* comb. nova, not indicating the holotypus. Therefore, the last syntaxon is an invalid name, according to articles 3 c and 5. In both cases, the authors indicate as characteristic species, physiognomically dominant, *Astragalus cylleneus* (=*Astracantha thracica* subsp. *cyllenea*). Unfortunately, this species was misidentified by these authors, since on the Mt. Killini in the stands where they have carried out the relevés there is exclusively *Astragalus rumelicus* subsp. *taygeticus*, while the true *A. cylleneus* is very rare and confined in depressed areas, such as dolines, characterized by very deep soils rich in silt-clay component, not occurring never on rocky substrata. The association occurs with the same ecological characteristics and floristic composition also on Mt. Erimanthos, where it was correctly described by Maroulis and Georgiadis [44] as *Marrubio cyllenei-Astragaletum rumelici*. However, this syntaxon is an illegitimate name being a synonym of the association described by Quézel [35], whose name must be corrected in *Cirsio hypopsilii-Astragaletum taygetici*. In this association are well represented the characteristic species of the three alliances proposed by Quézel [35], particularity already evidenced by Georgiadis and Dimopoulos [42] and also by Maroulis and Georgiadis [44].

*Asteri cyllenei-Globularietum stygiae* Quézel 1964, Vegetatio 12: 337 (Appendix C, Table A33).

Syn.: Association à *Aster cylleneus* et *Globularia stygia*. Quézel 1964, Vegetatio, 12:337.

Lectotypus: Table 20, rel. 5, Quézel [35], *hoc loco.*

Characteristic species: *Aster cylleneus*, *Globularia stygia*, *Macrotoma cephalotes*, *Taraxacum bythinicum*.

Structure and ecology: The association has its best expression between 2000 and 2330 m of altitude, within the orotemperate sub-Mediterranean bioclimatic belt. It can be observed sometimes up to 1800 m in stands representated by rocky ridge. Usually, it is localized on markedly rocky surfaces, constitute by carbonate substrata, as the ridges, saddles and stabilized screes, stends generally very windy with very shallow and undeveloped soils. It is a vegetation dominated by small prostrate dwarf shrubs mixed with several hemicryptophytes. The most important species are *Globularia stygia* and *Aster cylleneus*, rare endemics known for Mt. Chelmos and Mt. Killini. Floristically, the association is rather poor with low values of coverage. Dynamically, it can be considered an essentially edaphophilous vegetation.

Distribution: The association is currently known only for Mt. Chelmos and Mt. Killini in the northern Peloponnese.

Notes: This association was previously included by Quézel [35] within *Astragalo-Seslerion*, even though, as evidenced by the same author, the species of this alliance were not well represented in the relevés.

*Euphrasio salisburgensis-Asperuletum oetaeae* Quézel & Katrabassa 1974, Rev. Biol. Ecol. Medit. 1(1):19, corr. (Appendix C, Table A34).

Syn.: Association à *Asperula nitida* et *Euphrasia salisburgensis* Quézel & Katrabassa 1974.

Lectotypus: Table 4, rel. 3, Quézel and Katrabassa [40], *hoc loco.*

Characteristic species: *Asperula oetaea*, *Euphrasia salisburgensis*, *Iberis saxatilis* subsp. *saxatilis*.

Structure and ecology: The association is localized at 2000 and 2200 m of altitude in the windy crests, with flat surfaces formed by eroded limestone cracked and free of soil. It is linked to the oro-temperate sub-Mediterranean bioclimatic belt, where it assumes a role clearly edaphophilous. Physiognomically, it is dominated by small prostrate chamaephytes mixed to rosulate hemicryptophytes with coverage values not too high. It is significant the occurrence of some orophytes that find in this vegetation type their optimal growth conditions, such as *Asperula oetaea* (by Quèzel and Katrabassa [40] quoted as *A. nitida*), *Paronychia albanica* subsp. *graeca* (as *P. chionaea*), *Euphrasia salisburgensis* and *Iberis saxatilis* subsp. *saxatilis*. These authors distinguish within this association two sub-associations proposed as *erodietosum chrysanthi*, located on compact limestone, and *minuartietosum confusae*, occurring on calcareous substrata that flake on plakes.

Distribution: The association occurs only on Mt. Chelmos in northern Peloponnese.

*Marrubio cyllenei-Astragaletum calavrytensis* Musarella, Brullo & Giusso ass. nov. *hoc loco* (Appendix C, Table A35).

Syn.: Association à *Astragalus cylleneus* et *Cirsium cylleneum* subass. à *Astragalus cylleneus* Quézel & Katrabassa 1974, Rev. Biol. Ecol. Medit. 1(1):16, non Quézel 1964.

Holotypus: Appendix C, Table A35, rel. 5, *hoc loco.*

Characteristic species: *Astragalus calavrytensis*.

Structure and ecology: The association is localized on the little inclined slopes with rocky outcrops and more or less developed soils, rich in coarse skeletal component. It is developped in the bioclimatic belts between the supra-temperate sub-Mediterranean and the oro-temperate sub-Mediterranean, at altitudes of 1800 and 2200 m. Sometimes examples of this vegetation are found up to 1500 m of altitude in the markedly rocky stands. It is a plant community dominated by thorny pulvini of *Astragalus calavrytensis*, by Quézel and Katrabassa [40] mistakenly attributed to *A. cylleneus*. This species showing often high values of coverage, it is usually associated with *Marrubium cylleneum*, which assumes also a significant physiognomical role. The association has usually a clear climatophilous role, although sometimes, especially at lower altitudes, it represents an aspect of substitution, or at most edaphophilous. It can be considered as a geographical vicariant of the *Cirsio hypopsilii-Astragaletum taygetici* occurring in other mountains of the Peloponnese. Whitin this association, three subassociations linked to altitudinal ranges can be distinguished: (a) *elytrigietosum intermediae* subass. nov. (holotypus: rel. 6, *hoc loco*), distributed at lower altitudes (1250–1650 m), differentiated by *Elytrigia intermedia* and *Silene italica* subsp. *peloponnesiaca*; (b) *hippocrepidetum comosae* subass. nov. (holotypus: rel. 10, *hoc loco*), distributed between 1650 and 2000 m of altitude, which is differentiated by *Hippocrepis comosa*; (c) *tulipetosum australis* subass. nov. (holotypus: rel. 21, *hoc loco*), localized at 2000–2200 m of altitude, characterized by *Tulipa australis*, *Ornithogalum oligophyllum* and *Gagea villosa*.

Distribution: On the basis of current knowledge, this association seems to be exclusive of Mt. Chelmos in the northern Peloponnese.

Notes: Previously Quézel and Katrabassa [40] attributed this vegetation to the Ass. à *Astragalus cylleneus* et *Cirsium cylleneum* described by Quézel [35] for Mt. Killini. Effectively as previously emphasized, the aforesaid authors mistakenly attributed these populations of *A. calavrytensis* to *A. cylleneus*. As clearly can be observed from floristic composition and ecology, the vegetation of Mt Chelmos is well differentiated from that one of Mt. Killini and therefore they must be treated as two distinct associations.

*Plantagini graecae-Astragaletum cyllenei* Musarella, Brullo & Giusso ass. nov. *hoc loco* (Appendix C, Table A36).

Holotypus: Appendix C, Table A36, rel. 4, *hoc loco.*

Characteristic species: *Astragalus cylleneus*, *Alopecurus gerardii*, *Plantago atrata* subsp. *graeca*, *Potentilla recta*.

Structure and ecology: The association is localized in small depressions, similar to dolines, in the middle of the carbonatic rock outcrops, where there is a fairly deep soil rich in silt and clay, accumulated as a result of processes of washing away of the surrounding surfaces more or less sloping. It was surveyed in the supra-temperate sub-Mediterranean bioclimatic belt at 1800–2000 m of altitude. Physiognomically, it is dominated by *Astragalus cylleneus*, usually associated with numerous other orophytes of the alliance and higher syntaxa. The deep and compact soil justifies the occurrence of mesic species of the *Trifolion parnassi*, such as *Alopecurus gerardii*, *Plantago atrata* subsp. *graeca* and *Potentilla recta*. The arrangment of this association in the *Festuco achaicae-Marrubion cyllenei* rather than in the *Trifolion parnassi* is justified by the fact that from the structural point of view it is a shrub vegetation of tragacanthoid type, as most of the community of the alliance in question and not of a meadow with prevalence of small herbaceous hemicryptophytes. In addition, the floristic contingent of species of the *Cerastio candidissimi-Astragaletea rumelici* as well as the related alliance is clearly prevalent respect to that one of *Trifolietalia* and *Trifolion parnassi*.

Distribution: This association was observed on Mt. Killini, where is localized exclusively on Mt. Simios.

Notes: The *Plantagini graecae-Astragaletum cyllenei* is floristically and ecologically quite related to the *Nepeto epiroticae-Astragaletum corynthiaci* occurring on M. Parnassuss. In fact, both associations are characterized by the dominance of vicariant tragacantoidi species of *Astragalus* and by the occurrence of species of *Trifolion parnassi*. In addition, they are localized exclusively in stands more or less depressed with very thick and compact soils, poor in skeleton.

*Festuco achaicae-Minuartietum stellatae* Musarella, Brullo & Giusso ass. nov. *hoc loco* (Appendix C, Table A37).

Syn.: Aggr. à *Minuartia stellata* Quézel & Katrabassa 1974, Rev. Biol. Ecol. Medit. 1(1):18.

Comm. à *Minuartia stellata* Georgiadis & Dimopoulos 1993, Bot. Helv. 103:160.

Holotypus: Appendix C, Table A37, rel. 7, *hoc loco.*

Characteristic species: *Minuartia stellata*, *Festuca jeanpertii* subsp. *achaica*, *Allium cylleneum*.

Structure and ecology: The association is linked to rocky stands with calcareous outcrops or to compact rocky surfaces, more or less sloping at the foot of vertical walls. It is a habitat of semirupestrian type, with soils present only in rocky crevices or in small ledges. This vegetation seems to have its optimum in the oro-temperate sub-Mediterranean bioclimatic belt, at 2000–2250 m of altitude; examples can be observed also at lower altitudes (up to 1800 m) within the supra-temperate sub-Mediterranean belt. Physiognomically, the association is differentiated by the occurrence of compact and large pulvini of *Minuartia stellata*, sometimes mixed with smaller other ones of *Asperula boissieri*. The characteristics of the alliance of higher syntaxa are here well represented; among these show a greater diffusion and coverage *Astragalus angustifolius* subsp. *erinaceus, Festuca janpertii* subsp. *achaica* and *Festuca cyllenica* subsp. *cyllenica*. The association is a typical edaphophilous aspect, colonizing small areas scattered in midst of the tragacanthoid community of *Cirsio hypopsilii-Astragaletum taygetici* or *Plantagini graecae-Astragaletum cyllenei*. Previously, it was described by Quézel and Katrabassa [40] as. aggr. à *Minuartia stellata* and by Georgiadis and Dimopoulos (ref. [42] as comm. à *Minuartia stellata*).

Distribution: This association is well represented on Mt. Chelmos, Mt. Killini and Mt Klokos in the northern Peloponnese.

Notes: The *Festuco achaicae-Minuartietum stellatae* can be considered as a southern vicariant of the *Erysimo parnassi-Minuartietum stellatae*, association described by Quézel [35] for Mt. Parnassus.

*Alysso taygetei-Plantaginetum alpestris* Musarella, Brullo & Giusso ass. nov. *hoc loco* (Appendix C, Table A38).

Holotypus: Appendix C, Table A38, rel. 1, *hoc loco.*

Characteristic species: *Alyssum taygeteum*, *Plantago holosteum* var. *alpestris*, *Scorzonera mollis*.

Structure and ecology: The association is linked to cacuminal stations and very windy stands, localizing on carbonate substrata flaking in platelets, with primitive or very immature soils. It is distributed within the oro-temperate sub-Mediterranean bioclimatic belt, at 2000–2100, where it has its optimum on surfaces strongly eroded and subject to gelifluxion. It is a low pulvinar vegetation characterized by small, often prostrate, chamaephytes, in which play a significant role *Alyssum taygeteum* and *Plantago holosteum* var. *alpestris*, growing usually together with *Astragalus angustifolius* subsp. *erinaceus* and *Astragalus rumelicus* subsp. *taygeticus*.

Distribution: The association was observed only on Mt. Chelmos in the northern Peloponnese.

*Hieracio sartoriani-Seslerietum tenerrimae* Musarella, Brullo & Giusso ass. nov. *hoc loco* (Appendix C, Table A39).

Syn.: Ass. à *Astragalus cylleneus* et *Cirsium cylleneum* subass. à *Festuca varia* facies à *Sesleria coerulans* Quézel & Katrabassa 1974, Rev. Biol. Ecol. Medit. 1(1):18.

Holotypus: Appendix C, Table A39, rel. 7, *hoc loco.*

Characteristic species: *Sesleria tenerrima*, *Hieracium sartorianum*, *Arenaria cretica* var. *stygia*, *Galium incanum* subsp. *incanum*, *Silene auriculata*.

Structure and ecology: The association is localized on the rocky ridges, sometimes cacuminal, on substrates consisting of compact craked limestone, with soil present only in the rocky ravines and ledges. It was observed at 1900–2350 m of altitude, within the oro-temperate sub-Mediterranean bioclimatic belt, in ecologically very specialized contexts. In fact, in these stands there are very rigid environmental conditions, as strong winds, soil erosion, marked acclivity, gelifluction, etc. This vegetation represents a typical orophilous thinned out grassland, dominated by *Sesleria tenerrima*. In the middle of the tuffs of this grass grow several hemicryptophytes and chasmophyte, that highlight the semirupestrian characteristics of the habitat. This association clearly constitutes an edaphophilous aspect, replaced in typically rocky habitats by casmophilous comminities of *Asplenietea trichomanis*.

Distribution: Based on current knowledge, the association is known only for Mt. Chelmos in northern Peloponnese.

Notes: Within this association some relevés carried out by Quézel and Katrabassa [40] and considered by them as a facies à *Sesleria caerulans* of the subass. à *Festuca varia* of the ass. à *Astragalus cylleneus* et *Cirsium cyllenneum* can be included.

*Asperulo boissieri-Festucetum cyllenicae* Georgiadis & Dimopoulos ex Musarella, Brullo & Giusso ass. nov. *hoc loco* (Appendix C, Table A40).

Syn.: *Festuco cyllenicae-Asperuletum boissieri* Georgiadis & Dimopoulos 1993, Bot. Helv. 103(2):158, nom. inval. (art. 5).

Holotypus: Table 3, rel. 1, Georgiadis and Dimopoulos [42], *hoc loco.*

Characteristic species: *Festuca cyllenica* subsp. *cyllenica*, *Dianthus integer* subsp. *minutiflorus*.

Structure and ecology: The association colonizes the rocky calcareous substrata and stabilized screens, more or less sloping with shallow undeveloped and heavily skeletal soils. It is usually distributed at 2000 and 2200 m of altitude, coming down sometimes up to 1800 m, within the oro-temperate sub-Mediterranean bioclimatic belt, penetrating marginally also in that one supra temperate sub-Mediterranean belt. Physiognomically, it is differentiated by the dominance of large tufts of *Festuca cyllenica* subsp. *cyllenica,* that, sometimes, are mixed with those ones of *Sesleria vaginalis*. Scattered with these grasses there are some low prostrate pulvini of *Asperula boissieri* and *Astragalus angustifolius* subsp. *erinaceus*. It usually assumes a climatophilous role in the higher cacuminal places of the mountains.

Distribution: The association is known only for Mt. Killini in the northern Peloponnese.

Notes: This association was described by Georgiadis and Dimopoulos [42] from various stands of Mt. Killini and included by them with some perplexity within the *Astragalo-Seslerion*, due to the occurrence of a relevant number of characteristics of the *Eryngio-Bromion*. However, this syntaxon is an invalid name, because the authors do not indicate the relevé type of the association.

*Ranunculo brevifolii-Seslerietum tenerrimae* Musarella, Brullo & Giusso ass. nov. *hoc loco* (Appendix C, Table A41).

Holotypus: Appendix C, Table A41, rel. 3, *hoc loco.*

Characteristic species: *Sesleria tenerrima*, *Ranunculus brevifolius*, *Ranunculus sartorianus*, *Dianthus serratifolius* subsp. *abbreviatus*.

Structure and ecology: The association is linked to stabilized screes characterized by a marked acclivity and occurrence of undeveloped soils with a high percentage of skeleton. It is located at 2000–2100 m of altitude, within the oro-temperate sub-Mediterranean bioclimatic belt. It is a typical grassland characterized by the dominance of *Sesleria tenerrima*, showing a very scattered coverage, interspersed with small bare sufaces. Mixed with this grass there are tuffs of *Festuca cyllenica* subsp. *cyllenica,* which often show a high coverage, and several quite significant rosulate hemicryptophytes, such as *Ranunculus brevifolius, Ranunculus sartorianus*, *Dianthus serratifolius* subsp. *abbreviatus*. For its peculiar ecology, the association must to be considered as an edaphophilous aspect, which tends due to the natural evolution of the soil, towards pulvinate communities, structurally more evolved.

Distribution: The association occurs only in Killini massif on Mt. Simios (northern Peloponnese).

*Astragaletum hellenico-erinacei* Musarella, Brullo & Giusso ass. nov. *hoc loco* (Appendix C, Table A42).

Holotypus: Appendix C, Table A42, rel. 6, *hoc loco.*

Characteristic species: *Astragalus angustifolius* subsp. *erinaceus*, *A. hellenicus*.

Structure and Ecology: The association is localized on fairly inclined rocky slopes, with soils more or less deep and rich in coarse skeleton. From the structural point of view, the vegetation is differentiated by the dominance of tragacanthoid pulvini of *Astragalus angustifolius* subsp. *erinaceus*, which grows togheter with several chamaephytes and hemicryptophytes, among them *Astragalus hellenicus*, endemic species, rare in the Peloponnese. This association, showing usually a climatophilous character, at least in the rocky cacuminal stands, represents often a substitution aspect, replacing the forests of *Abies cephalonica* as a result of soil degradation processes.

Distribution: The association was suveyed only on Mt. Menalon in the Central Peloponnese.

Notes: On the whole, it can be considered as a thermophilous vicariant of the community with *Astragalus rumelicus* subsp. *taygeticus* occurring in the other massifs of the northern Peloponnese.

*Festucetum polito-cyllenicae* Maroulis & Georgiadis 2005, Fitosociologia 42(1):44, corr. (Appendix C, Table A43).

Syn.: *Festuco politae-Festucetum cyllenicae* Maroulis & Georgiadis 2005, Fitosociologia 42(1):44.

Holotypus: Table 2, rel. 509, Maroulis and Georgiadis [44].

Characteristic species: *Festuca cyllenica* subsp. *cyllenica*, *Festuca polita*, *Campanula albanica* subsp. *albanica* and *Taraxacum delphicum*.

Structure and ecology: The association is localized along the very sloped surfaces on stabilized screes or rocky stands with undevelopped calcareous soils rich in scheleton. It is widespread at 1750–2200 m of altitude, within the supra-temperate and oro-temperate sub-Mediterranean bioclimatic belts, where it plays a climatophilous role. This vegetation constitutes dense orophilous grasslands dominated by *Festuca cyllenica* subsp. *cyllenica*, *Festuca polita* and *Sesleria vaginalis*, where are frequent several other hemicryptophytes and small chamaephytes.

Distribution: According to literature, it is widespread on various mountains of the Erimanthos massif in the Northern Peloponnese.

Notes: The association described by Maroulis and Georgiadis [44] as *Festuco politae-Festucetum cyllenicae*, was included by the authors in the *Eryngio-Bromion* although there is a significant contingent of characteristics of *Astragalo-Seslerion*. For its structure and ecology, as well as for its floristic composition, this association is quite related to *Asperulo boissieri-Festucetum cyllenicae* from Mt. Killini, from which differs mainly for its floristic set.

*Arenario filicaulis-Festucetum cyllenicae* Musarella, Brullo & Giusso ass. nov. *hoc loco* (Appendix C, Table A44).

Holotypus: Appendix C, Table A44, rel. 1, *hoc loco.*

Characteristic species: *Festuca cyllenica* subsp. *cyllenica*, *Arenaria filicaulis* subsp. *filicaulis*, *Ranunculus psilostachys*.

Structure and ecology: The association occurs mainly on stabilized screes or on quite inclined slopes covered by calcareous stones, mixed with scarce humus. It is surveyed at 1500 and 1600 m of altitude on northern slopes, it colonizes large surfaces. Physiognomically, it is characterized by the dominance of large tufts of *Festuca cyllenica* subsp. *cyllenica*, which grows very well on inclined slopes subject to long periods of snow coverage. This vegetation, where it is frequent also *Festuca jeanpertii* subsp. *achaica,* results well differentiated from the other associations with *Festuca cyllenica* subsp. *cyllenica* for the occurrence of *Arenaria filicaulis* subsp. *filicaulis* and *Ranunculus psilostachys*.

Distribution: The association is frequent on Mt. Panachaiko in the northern Peloponnese.

*Aurinio moreanae-Lomelosietum crenatae* Musarella, Brullo & Giusso ass. nov. *hoc loco* (Appendix C, Table A45).

Holotypus: Appendix C, Table A45, rel. 6, *hoc loco.*

Characteristic species: *Aurinia moreana*, *Lomelosia crenata* subsp. *crenata*.

Structure and ecology: The association is localized in semirupestrian habits on very sloped (70–80°) limestone outcrops, usually showing a northern exposure. This vegetation dominated by *Lomelosia crenata* subsp. *crenata* and *Aurinia moreana*, is distributed at 1600 and 1700 m of altitude. It is an edaphophilous community, replacing in this rocky stand the *Cirsio hypopsilii-Astragaletum taygetici*. On the whole, the species of the alliance and higher syntaxa are here well represented, among them there are *Festuca jeanpertii* subsp. *achaica*, *Astragalus angustifolius* subsp. *erinaceus*, *Astragalus rumelicus* subsp. *taygeticus*, *Achillea umbellata*, etc.

Distribution: The association was surveyed only on Mt. Klokos in northern Peloponnese.

*Onosmo malickyi-Astragaletum hellenici* Musarella, Brullo & Giusso ass. nov. *hoc loco* (Appendix C, Table A46).

Holotypus: Appendix C, Table A46, rel. 2, *hoc loco.*

Characteristic species: *Onosma erectum* subsp. *malickyi*, *Astragalus hellenicus*, *Alyssum murale*.

Structure and ecology: The association occurs at 1300–1400 m of altitude, in the clearing within the *Abies cephalonica* woodlands. The surfaces are slightly inclined, and the soils are covered with a bed of fir needles. Floristically, it is characterized by hemicryptophytes and small chamaephytes, among them *Onosma erectum* subsp. *malickyi*, *Astragalus hellenicus*, *Alyssum murale*, *Helianthemum hymettium* and *Festuca jeanpertii* subsp. *achaica*.

Distribution: This community was surveyed only on Mt. Chelmos near Mavros Logos.

*Violo graecae-Festucetum cyllenicae* Musarella, Brullo & Giusso ass. nov. *hoc loco* (Appendix C, Table A47).

Holotypus: Appendix C, Table A47, rel. 7, *hoc loco.*

Characteristic species: *Festuca cyllenica* subsp. *cyllenica*, *Viola graeca*, *Ornithogalum oligophyllum*.

Structure and ecology: The association occurs on calcareous stabilized screes and stony slopes at 2000 and 2500 m of altitude. It is a pioneer vegetation linked to slightly inclined surfaces and poor in soil. Physiognomically, it is characterized by the dominance of *Festuca cyllenica* subsp. *cyllenica*, which constitute wide grasslands where occur several orophytes of higher syntaxa. Small hemicryptophytes and geophytes found often refuge among the tuffs of this plant, among them *Viola graeca*, *Ornithogalum oligophyllum, Allium frigidum*, *Galium taygeteum*, *Geocaryum peloponnesiacum*, etc.

Distribution: This association is widespread on Mt. Chelmos.

*Tripodio graeci-Helictotrichetum heldreichii* Musarella, Brullo & Giusso ass. nov. *hoc loco* (Appendix C, Table A48).

Holotypus: Appendix C, Table A48, rel. 1, *hoc loco.*

Characteristic species: *Helictotrichon convolutum* subsp. *heldreichii*, *Tripodion graecum*.

Structure and ecology: The association occurs at 1400 and 1600 m of altitude, in the large rock clearing within the *Abies cephalonica* woodlands. Normally it is frequent along the more or less inclined slopes characterized by rocky outcrops with very shallow and immature soils. The occurrence of *Tripodion graecum* is significant, i.e., since it is a species known only from a few places in the Peloponnese and Anatolia (mainly in the Taurus region). It is usually associated with *Helictotrichon convolutum* subsp. *heldreichii*, generally with high values of coverage, and *Festuca jeanpertii* subsp. *achaica*. In this vegetation the species of higher syntaxa are overall well represented.

Distribution: The association is widespread in the lower montane belt of Mt. Menalon, Central Peloponnese.

*SIDERITIDO CLANDESTINAE-ASPERULION MUNGIERI* Musarella, Brullo & Giusso all. nov. *hoc loco.*

Syn.: *Eryngieto-Bromion* Quézel 1964, Vegetatio, 12:326, p.min.p., nom. ambig. rejic. propos. (art. 36).

*Eryngio multifidi-Bromion fibrosi* Quézel 1964, corr. Quézel, Barbero & Akman 1992, Ecol. Medit. 18:82 p.min.p., nom. ambig. rejic. propos. (art. 36).

*Astragaleto-Seslerion* Quézel 1964, Vegetatio, 12:326, p.min.p., nom. ambig. rejic. propos. (art. 36).

*Astragalo angustifolii-Seslerion coerulantis* Quézel 1964, corr. Quézel, Barbero & Akman 1992, Ecol. Medit. 18:82, p.min.p., nom. ambig. rejic. propos. (art. 36).

*Stipeto-Morinion* Quézel 1964, Vegetatio, 12: 26, p.min.p., nom. ambig. rejic. propos. (art. 36).

*Stipo pulcherrimae-Morinion persicae* Quézel 1964, corr. Quézel, Barbero & Akman 1992, Ecol. Medit. 18:82, p.min.p., nom. ambig. rejic. propos. (art. 36).

Holotypus: *Sideritido clandestinae-Astragaletum taygetici* Musarella, Brullo & Giusso ass. nov.

Characteristic species: *Achillea setacea*, *Achillea taygetea*, *Allium pycnotrichum*, *Anthemis laconica*, *Asperula boryana*, *Asperula mungieri*, *Astragalus taygeteus*, *Asyneuma psaridis*, *Crepis heldreichiana*, *Nepeta camphorata*, *Phitosia crocifolia*, *Sideritis clandestina* subsp. *clandestina*, *Viola sfikasiana*.

Structure and ecology: It gathers, likewise to the previous alliances included in order *Eryngio multifidi-Armerietalia orphanidis*, the orophilous plant communities rich in chamaephytes and nanophanerophytes, often with pulvinate habit, as well as in hemicryptophytes, while rarer are the geophytes. On the whole, the associations belonging to this alliance show a more marked thermophily than those ones of the other two alliances. In addition, the considerable contingent of endemics that characterizes this syntaxon is represented mainly by species taxonomically quite isolated or otherwise of remarkable phytogeographical significance. From the bioclimatic point of view, this alliance falls in an area affected by termotypes referring to supra-and oro-Mediterranean, since one detects a long period of high summer dryness enough, although there is a certain tendency towards the supra- and oro-temperate sub-Mediterranean type, with ombrotypes characterized by scarce rainfall, especially during the summertime.

Distribution: The alliance is confined to the southern Peloponnese including the massifs of the Taygetos and Parnon.

Notes: Into this alliance, analogously to the other two previously described, fall within part of the alliances described by Quézel [35], namely *Eryngio-Bromion*, *Stipo-Morinion*, and *Astragalo-Seslerion*.

*Scabioso taygeteae-Onosmetum leptanthae* Quézel 1964, Vegetatio, 12:327 (Appendix C, Table A49).

Syn.: Association à *Scabiosa taygetea* et *Onosma leptanthum* Quézel 1964, Vegetatio, 12: 327.

Lectotypus: Table 15, rel. 2, Quézel [35], *hoc loco.*

Characteristic species: *Onosma leptantha*, *Scabiosa taygetea* subsp. *taygetea*, *Calamintha suaveolens*, *Tripodion graecum*.

Structure and ecology: The association is located on rocky outcrops or however more or less rocky surfaces consisting of compact limestone subject to heavy erosion and washing away. The soils are very superficial and localized in crevices and ledges. It is widespread within the meso-mediterranean and supra-Mediterranean bioclimatic belt, at 1250–1800 m of altitude. This vegetation is dominated by chamaephytes and nanophanerophytes of small and medium size, mixed to several caespitose hemicryptophytesche, and among them there are *Onosma leptantha, Scabiosa taygetea* subsp. *taygetea*, *Pterocephalus perennis* spp. *perennis, Stipa endotricha, Dasypyrum hordeaceum, Koeleria mitrushii, Bromus riparius, Festuca jeanpertii* subsp. *jeanpertii.* The association has a purely edaphophilous role, although it represents a secondary aspect too, as a result of degradation processes of *Abies cephalonica* woodlands.

Distribution: The association is distributed on Mt. Taygetos in the southern Peloponnese.

Notes: This association was considered by Quézel et al. [80] as the nomenclatural type of the alliance *Stipo pulcherrimae-Morinion persicae*, although in the relative phytosociological table there are several characteristic species of the other two alliances described by Quézel [3].

*Danthoniastro compacti-Fumanetum alpinae* Musarella, Brullo & Giusso ass. nov. *hoc loco* (Appendix C, Table A50).

Holotypus: Appendix C, Table A50, rel. 4, *hoc loco.*

Characteristic species: *Fumana paphlagonica* subsp. *alpina*, *Danthoniastrum compactum*.

Structure and ecology: The association is localized on the slightly sloping limestone slabs, especially with an eastern exposure. It is developped within the supra-Mediterranean bioclimatic belt at an altitude of about a 1700 m. The surfaces are free of soil except in the cracks and small depressions, that allow the establishment of a rather sparse vegetation. It is a vegetation rich in small prostrate chamaephytes, sometimes creeping, among them particularly significant are *Fumana paphlagonica* subsp. *alpina*, *Helianthemum hymettium*, *Teucrium montanum* var. *parnassicum* and *Asperula mungieri.* Several hemicryptophytes, such as *Danthoniastrum compactum, Festuca jeanpertii* subsp. *jeanpertii, Koeleria mitrushii* and *Stipa endotricha* are also well represented. This association plays a clearly edaphophilous role replacing the *Scabioso taygeteae-Onosmetum leptanthae* in the above-mentioned habitats.

Distribution: Basing on the current knowledge, the association is confined to a small area of Mt. Taygetos in the southern Peloponnese.

*Sideritido clandestinae-Astragaletum taygetici* Musarella, Brullo & Giusso ass. nov. *hoc loco* (Appendix C, Table A51).

Syn.: Association à *Sideritis theezans*, Quézel 1964, Vegetatio, 12:331, nom. illeg. (art. 29).

Holotypus: Appendix C, Table A51, rel. 14, *hoc loco.*

Characteristic species: *Astragalus rumelicus* subsp. *taygeticus*, *A. taygeteus*, *Plantago holosteum* var. *alpestris*, *Hypericum olympicum* and *Arabis subflava*.

Structure and ecology: The association is widely distributed on flat and sometimes more or less sloped surfaces, characterized by not very deep soils, rich in minute skeletal component of carbonatic nature. It grows at 1700 and 2100 m of altitude, within the supra-Mediterranean bioclimatic belt with penetrations in oro-Mediterranean that one. This vegetation is physiognomically differentiated by the dominance of flashy tragacanthoid pulvini of *Astragalus rumelicus* subsp. *taygeticus*, *A. taygeteus* and, more rarely, by *A. angustifolius* subsp. *erinaceus.* Many other small shrubs are also quite frequent, such as *Plantago holosteum* var. *alpestris*, *Sideritis clandestina* subsp. *clandestina*, *Cerastium candidissimum* and several hemicryptophytes. Overall, the association, which usually shows a high value of coverage, must be considered as a climatophilous aspect, spread on all slopes regardless of exposure. From the physiognomic-structural point of view, it is quite related with the other pulvinate tragacanthoid associations dominated by *Astragalus rumelicus s.l.*, such as *Cirsio hypopsilii-Astragaletum taygetici*, *Astragaletum lacteo-taygetici* and *Marrubio velutini-Astragaletum rumelici*.

Distribution: The association is exclusive of Mt Taygetos, in the southern Peloponnese.

Notes: This vegetation was described by Quézel [35] with the name “Association à *Sideritis theezans*”, which actually is not very significant from the floristic-structural viewpoint. In fact, *Sideritis theezans* (whose correct name is *S. clandestina* subsp. *clandestina*) is a small camaephyte with a secondary physiognomically role in the context of this shrubby association, dominated by some tragacanthoid nanophanerophytes, such as *Astragalus rumelicus* subsp. *taygeticus* and *A. taygeteus*. According to art. 29, the syntaxon is an illegitimate name and therefore should be replaced by a new name that expresses in clear way its floristic and physiognomic-structural peculiarities. It is therefore proposed the new name *Sideritido clandestinae-Astragaletum taygetici* with a better floristic characterization. In this regard it should be noted that *S. clandestina* subsp. *clandestina* is not exclusive to this association, but it is an endemic chamaephyte widespread in various orophilous communities of southern Peloponnese and therefore it has been proposed as characteristics of the alliance *Sideritido clandestinae-Asperulion mungieri*.

*Rindero graecae-Acantholimetum graeci* Quézel 1964, Vegetatio, 12:336 (Appendix C, Table A52), corr.

Syn.: Association à *Acantholimon echinus* et *Rindera graeca* Quézel 1964, Vegetatio, 12:336.

Lectotypus: Table 19, rel. 2, Quézel [35], *hoc loco.*

Characteristic species: *Sesleria vaginalis*, *Jurinea taygetea*, *Minuartia condensata*, *Campanula papillosa*, *Erigeron epiroticus*, *Aethionema carlsbergii*, *Alyssum taygeteum*, *Bupleurum sibthorpianum*.

Structure and ecology: The association is localized in cacuminal stands of high altitude, about 2200–2400 m, within the oro-Mediterranean bioclimatic belt. It prefers quite acclive surfaces, where it shows a coverage of 40–70%, which decreases significantly at higher altitudes. The substrata are represented by carbonatic rocks that break up into plaquettes or sometimes by semi-stabilized screes. Physiognomically the vegetation is characterized by thorny pulvini of *Astragalus angustifolius* subsp. *erinaceus* and *Acantholimon graecum*, mixed to several caespitose hemicryptophytes, such as *Sesleria vaginalis*. In addition, it is very significant the occurrence of some rare endemics exclusive of this vegetation, as *Jurinea taygetea* and *Aethionema carlsbergii*. This community, showing a clear climatophilous role, is linked to winterproof environmental features, such as the prolonged snow cover, the accentuated phenomena of gelifluction, exposure to cold winds and the occurrence of rocky substrata with shallow and immature soils. The name of this association must be corrected in *Rindero graecae-Acantholimetum graeci* since *Acantholimon echinus* subsp. *echinus* used by Quézel [35] is taxonomically incorrect and should be attributed to *Acantholimon graecum* (see Dimopoulos et al. [71].

Distribution: The association is confined to the cacuminal higher part of Mt. Taygetos, in the southern Peloponnese.

Notes: This association was included by Quézel [35] in the *Astragalo-Seslerion* and afterwards indicated by Quézel et al. [80] as the lectotype of this alliance.

*Onosmo heterophyllae-Astragaletum erinacei* Musarella, Brullo & Giusso ass. nov. *hoc loco* (Appendix C, Table A53).

Holotypus: Appendix C, Table A53, rel. 3, *hoc loco.*

Characteristic species: *Onosma heterophylla.*

Structure and ecology: The association was surveyed on carbonatic rocky slopes more or less inclined occurring at relatively low altitudes (1300–1500 m), characterized by coarse material mixed with immature soils. It is localized within the meso-Mediterranean bioclimatic belt, usually occupied by *Abies cephalonica* woodlands. From the physiognomic-structural point of view, this vegetation is differentiated by thorny pulvini of *Astragalus angustifolius* subsp. *erinaceus*, that grow together with other shrubs and several caespitose hemicryptophytes; among the latter there are *Onosma heterophylla*, *Stipa endotricha, Festuca jeanpertii* subsp. *jeanpertii, Koeleria mitrushii, Bromus riparius, Stipa holosericea*. It is a substitution community linked to the degradation processes of the woody vegetation, although in strictly rocky conditions it tends to have an edaphophilous role.

Distribution: The association has been surveyed on M. Parnon, exclusively at Prophitis M. Ilias, near Agriani in the Southern Peloponnese.

Notes: This association is closely related to *Scabioso taygeteae-Onosmetum leptanthi* from Mt. Taygetos, of which it can be considered a geograpical vicariant.

*Astragaletum lacteo-taygetici* Musarella, Brullo & Giusso ass. nov. *hoc loco* (Appendix C, Table A54).

Holotypus: Appendix C, Table A54, rel. 7, *hoc loco.*

Characteristic species: *Astragalus rumelicus* subsp. *taygeticus*, *A. lacteus*, *Cynoglossum pustulatum* subsp. *parviflorum*.

Structure and ecology: The association is linked to rocky slopes characterized by very compact limestone with soil accumulating only in crevices and depressions of the rocks. It is well developed between 1400 and 1800 m of altitude, within the meso-Mediterranean and supra-Mediterranean bioclimatic belts, constituting usually a climatophilous vegetation which tends to expand towards lower elevations as a result of the degration processes of *Abies cephalonica* forest. This vegetation is physiognomically characterized by tragacanthoid pulvini of *Astragalus rumelicus* subsp. *taygeticus* and *A. angustifolius* subsp. *erinaceus*, in the midst of which grow several small chamaephytes and caespitose or rosulate hemicryptophytes.

Distribution: The association occurs only on the massif of Parnon, in the southern Peloponnese, where it is common in several mountains.

Notes: From the physiognomic-structural and partially floristic viewpoint, this association is quite similar to *Sideritido clandestinae-Astragaletum taygetici* from Mt. Taygetos, differing, however especially for the dynamic role, since the latter association is distributed in a higher altitudinal belt.

*Violo parnoniae-Astragaletum erinacei* Musarella, Brullo & Giusso ass. nov. *hoc loco* (Appendix C, Table A55).

Holotypus: Appendix C, Table A55, rel. 2, *hoc loco.*

Characteristic species: *Viola parnonia*, *Astragalus agraniotii*, *Centaurea parnonia*.

Structure and ecology: The association covers the rather inclined rocky slopes characterized by generally undeveloped calcareous soils, sometimes represented by lithosols. It is widespread within the bioclimatic supra-Mediterranean bioclimatic belt at 1700–1900 m of altitude, regardless of exposure. In this community plays a significant physiognomic role *Astragalus angustifolius* subsp. *erinaceus*, which tends to constitute with its characteristic compact thorny pulvini extensive populations. Furthermore, the occurrence of several chamaephytes and hemicryptophytes, including in particular some rare endemics such as *Viola parnonia*, *Astragalus agraniotii* and *Centaurea parnonia,* differentiate vey well this vegetation from other ones of this alliance. Based on the edaphic characteristics, it is possible to distinguish two subassociations, indicated as *astragaletosum erinacei* and *asperuletosum malevonensis*, which will be examined below.

Distribution: The association was surveyd exclusively on Megali Tourla, which is the highest mountain of the Parnon Massif in the southern Peloponnese.

*(a)*  astragaletosum erinacei Musarella, Brullo & Giusso subass. nov. *hoc loco* (Appendix C, Table A55, rel. 1–3).

Holotypus: Appendix C, Table A55, rel. 2, *hoc loco.*

Characteristic species: *Astragalus angustifolius* subsp. *erinaceus* (dominant).

Structure and ecology: It represents the typical aspect of the association and is localized on the inclined slopes of the summit, where it colonizes surfaces rich in clastic stabilized material with more or less deep and rich in coarse skeleton soils. Physiognomically, it is differentiated by the dominance of *Astragalus angustifolius* subsp. *erinaceus*, which tends to cover most of the surface occupied by the association. This subassociation plays a prevalently climatophylous role, although currently it is also widespread in stands in the past occupied by *Abies cephalonica* forests, where has a secondary meaning as a result of degradation processes of soil.

Distribution: See association.

*(b)*  *asperuletosum malevonensis* Musarella, Brullo & Giusso subass. nov. *hoc loco* (Appendix C, Table A55, rel. 4–11).

Holotypus: Appendix C, Table A55, rel. 10, *hoc loco.*

Characteristic species: *Achillea umbellata*, *Asperula malevonensis*, *Helianthemum canum* subsp. *canum*.

Structure and ecology: It replaces the typical aspect in correspondence of compact outcrops, consisting of more or less cracked limestones. In these stands, some species which show markedly chasmophytic habit often occur, such as *Achillea umbellata*, *Helianthemum canum* subsp. *canum* and the local endemic *Asperula malevonensis*, thus providing a distinct physiognomy. It is an edaphophilous aspect showing a scarce coverage, forming small patches in the middle of the previous subassociation.

Distribution: See association.

*NOAEO MUCRONATAE-SILENETALIA URVILLEI* Musarella, Brullo & Giusso ord. nov *hoc loco.*

Holotypus: *Asperulion samiae* Musarella, Brullo & Giusso all. nov. *hoc loco.*

Characteristic species: *Acantholimon aegaeum*, *Aethionema saxatile* subsp. *creticum*, *Alopecurus davisii*, *Astragalus angustifolius* subsp. *aegeicus*, *Atraphaxis billardierei*, *Bunium microcarpum* subsp. *microcarpum*, *Centaurea urvillei* subsp. *urvillei*, *Dianthus zonatus*, *Draba heterocoma* subsp. *archipelagi*, *Erysimum hayekii*, *Galium heldreichii*, *Inula heterolepis*, *Jurinea cadmea*, *Koeleria lobata*, *Minuartia anatolica* var. *polymorpha*, *Noaea mucronata*, *Paracaryum aucheri*, *Paronychia chionaea*, *Pterocephalus pinardii*, *Sesleria anatolica*, *Sideritis sipylea*, *Silene urvillei*, *Stachys cretica* subsp. *smyrnaea*, *Verbascum pycnostachyum*.

Structure and ecology: This order groups the orophilous pulvinate plant communities dominated by chamaephytes and nanophanerophytes with tragacanthoid habit linked to cacuminal very sunny and windy stands at altitudes higher than 900–1000 m. The substrates are prevalently carbonatic with immature soils rich in coarse skeleton occurring mainly in the rocky crevices. The vegetation belonging to this syntaxon colonize mainly rocky surfaces more or less denuded, often quite sloping, affected by moist marine winds or a regime of mists. During the winter period these stations are usually covered for quite short periods by snow. On the basis of investigations carried out in the Aegean area, the habitats colonized by this type of vegetation are represented mainly by the summit areas of island mountains, where the peculiar environmental conditions above emphasized can be found. From the bioclimatic point of view, the order is linked to mountain or high mountain habitats falling into meso- and supra-Mediterranean belts, extending marginally also in the oro-Mediterranean one. Floristically, the order is characterized by a rich contingent of species having mainly an East Aegean-Anatolian distribution, including also several rare endemics.

Distribution: Basing on the current knowledge, the order seems distributed on the mountains of some north-eastern Aegean islands, such as Samos, Chios, Lesvos and Thassos. It is likely that plant communities related to this syntaxon are also present on the island of Samothraki, Mt. Athos, and some coastal mountains of western Anatolia.

Notes: The *Noaeo mucronatae-Silenetalia urvillei* must be considered as the eastern vicariant of *Eryngio multifidi-Armerietalia orphanidis,* distributed in the mainland of central-southern Greece, as well as in some Ionian Islands and Euboea.

*ASPERULION SAMIAE* Musarella, Brullo & Giusso all. nov. *hoc loco.*

Holotypus: *Astragaletum samii* Musarella, Brullo & Giusso ass. nov. *hoc loco.*

Characteristic species: *Allium hirtovaginatum* subsp. *samium*, *A. orosamium*, *Alyssum samium*, *Anthemis samia*, *Asperula samia*, *Erodium sibthorpianum.* subsp. *vetteri*, *Satureja spinosa* var. *glabra*, *Thymus samius*.

Structure and ecology: This alliance gathers the plant communities occurring in cacuminal stands of island mountains, localized at 900–1400 m of altitute. It is an essentially calcicolous syntaxon linked to a meso-Mediterranean bioclimatic belt, extending towards the supra-Mediterranean one. It has its best expression in very peculiar ecological conditions, where some environmental factors play an important role, such as the marine moist winds, rather cold during the autumn and winter, the erosive action of weathering on rocky surfaces, the mists, and the marked summer dryness. Floristically, it is differentiated by a rich endemic and rare species contingent, having a considerable taxonomical and phytogeographical significance.

Distribution: The alliance is confined to the mountains of the island of Samos in the eastern Aegean.

*Astragaletum samii* Musarella, Brullo & Giusso ass. nov. *hoc loco* (Appendix C, Table A56).

Holotypus: Appendix C, Table A56, rel. 3, *hoc loco.*

Characteristic species: *Astragalus creticus* subsp. *samius* and *Allium orosamium*.

Structure and ecology: The association is localized on calcareous slopes of cacuminal areas at 1000–1400 m of altitude, where it tends to cover wide surfaces. It has its best expression on compact rocky substrates, often very sloping, represented by calcareous outcrops and buttresses, with soils present almost exclusively in the crevices and ledges. From the bioclimatic viewpoint, this vegetation grows within the meso-Mediterranean belt, extending marginally also in the oro-Mediterranean one, showing a role, not strictly climatophilous, but rather of edaphophilous vegetation. However, it is spread in an area located above the limit of the forests, consisting mainly of *Pinus brutia* and *Quercus calliprinos* woodlands. In the tracts with deeper and mature soils, this pulvinate vegetation is mixed to relict of orophilous conifer forest of *Junipero-Pinetea sylvestris*, where *Juniperus foetidissima* and *J. oxycedrus* play an important role. Floristically, the association is characterized by the dominance of thorny pulvini of *Astragalus creticus* subsp. *samius*, punctiform endemic of considerable phytogeographical interest. Several relevant endemic orophytes, such as *Allium orosamium, Alyssum samium, Anthemis samia, Asperula samia, Erodium sibthorpianum*. subsp. *vetteri*, and *Thymus samius* occur in this association and probably also *Centaurea xylobasis,* a rare endemic exclusive of these cacuminal stands.

Distribution: The association is exclusive of the cacuminal area of Mt. Kerkis in the island of Samos (East Aegean).

*Thymo samii-Astragaletum condensati* Musarella, Brullo & Giusso ass. nov. *hoc loco* (Appendix C, Table A57).

Holotypus: Appendix C, Table A57, rel. 10, *hoc loco.*

Characteristic species: *Astragalus condensatus* (=*A. ptilodes*), *Valeriana dioscoridis*, *Phlomis grandiflora Fritillaria carica*, *Centaurea cariensis* subsp. *maculiceps*, *Petrorhagia armeriodes*, *Vincetoxicum canescens* subsp. *peduncolatum*, *Allium karvounis*, *Lomelosia polykratis*, *Ranunculus rumelicus*, *Crocus oliveri* subsp. *balansae*.

Structure and ecology: The association is localized in a cacuminal area characterized by outcrops of compact crystalline limestone (marble), with surfaces flat or slightly sloping. The soils are very shallow and fill the depressions and cracks of the rock. The area in which it is developed, localized at 1100–1200 m of altitude, falls within the meso-Mediterranean bioclimatic belt. From the structural point of view, it is observed the dominance of low pulvinate or creeping shrubs, among them *Astragalus condensatus*, tragacanthoid species, playing a relevant role, and various other small shrubs, such as *Asperula samia, Noaea mucronata, Satureja spinosa* var. *glabra, Thymus samius,* etc. In this association are found numerous endemic species rather rare exclusive of these cacuminal stands. Outside of the limestone outcrops, in correspondence of the schistose substrata, this pulvinate vegetation is replaced by *Pinus pallasiana* woodlands, adaphically much more exigent. On the whole, this vegetation has a clear edaphophilous role.

Distribution: The association is exclusive of cacuminal area of Mt. Ambelos in the island of Samos (East Aegean).

Notes: The *Thymo samii-Astragaletum condensatis* can be considered a vicariant of the *Astragaletum samii*, occurring on different substrata in another mountain of Samos.

*Campanulo lyratae-Genistetum parnassicae* Musarella, Brullo & Giusso ass. nov. *hoc loco* (Appendix C, Table A58).

Holotypus: Table 11, rel. 5, Christodoulakis and Georgiadis [41], *hoc loco.*

Characteristic species: *Genista parnassica*, *Campanula lyrata* subsp. *lyrata*.

Structure and ecology: Based on the relevés published by Christodoulakis and Georgiadis [41], at altitude lower than 1000 m always in calcareous rocky stands, more or less sloping, the *Astragaletum samii* is replaced by another type of shrub pulvinate vegetation, differentiated by the dominance of *Genista parnassica*. In the places occupied by this vegetation, *Astragalus creticus* subsp. *samius* is wholly absent, as well as the species most significant of the alliance and order decrease and become quite rare. This community, which is proposed as *Campanulo lyratae-Genistetum parnassicae*, therefore, can be considered as a vicariant of low altitude of the *Astragaletum samii*. From the bioclimatic point of view, the association is distributed in the meso-Mediterranean belt.

Distribution: The association is known only to Mt. Kerkis in the island of Samos (East Aegean).

*Arenario guicciardii-Seslerietum anatolicae* Musarella, Brullo & Giusso ass. nov. *hoc loco* (Appendix C, Table A59).

Holotypus: Appendix C, Table A59, rel. 3, *hoc loco.*

Characteristic species: *Sesleria anatolica*, *Arenaria guicciardii*, *Pimpinella peregrina*.

Structure and ecology: The association is localized in calcareous markedly sloping rocky places with soils occurring only in the cracks and the crevices. It seems to have its optimum at 900–1000 m of altitude on a little sunshine surface especially with northern exposure, within the meso-Mediterranean bioclimatic belt. This vegetation is dominated by *Sesleria anatolica* which grows togheter with a rich contingent of small pulvini or creeping shrubs, such as *Anthemis samia, Inula heterolepis, Noaea mucronata, Satureja spinosa* var. *glabra, Sideritis sipylea,* etc. As concerns its dynamic role, it is a community prevalently edaphophilous, occurring within the climatophilous *Pinus brutia* forests, which is linked to surfaces with mature and more or less deep soils.

Distribution: The association was surveyed on Mt. Kerkis in the island of Samos (East Aegean).

Notes: The *Arenario guicciardii-Seslerietum anatolicae* tends to replace the *Astragaletum samii* at altitudes lower than 1000 m, limited to shady and fresh stands.

*FESTUCO PSEUDOSUPINAE-ASTRAGALION AEGEICI* Musarella, Brullo & Giusso all. nov. *hoc loco.*

Holotypus: *Anthemido discoideae-Astragaletum aegeici* Musarella, Brullo & Giusso ass. nov. *hoc loco.*

Characteristic species: *Anthemis cretica* subsp. *leucanthemoides*, *Astragalus lesbiacus*, *Crepis sancta* subsp. *nemausensis*, *Erysimum hayekii*, *Festuca pseudosupina*.

Structure and ecology: The alliance gathers pulvinate plant communities dominated by small tragacanthoid shrubs occurring in the mountain cacuminal stands of insular mountains. They are localized at 900–1300 m of altitude, mainly within the meso-Mediterranean bioclimatic belt. The associations falling in this sintaxon are very specialized and linked to very peculiar edaphic and bioclimatic conditions. They are circumscribed to carbonatic substrata represented by ridges and rocky outcrops, with soils present only in the cracks and crevices. The alliance is floristically differentiated by endemic species exclusive to these summit stands, that emphasized their marked geographical isolation.

Distribution: The alliance is circumscribed to the East Aegean islands of Lesvos and Chios.

Notes: This syntaxon can be considered as a geographical vicariant of the *Asperulion samiae.*

*Anthemido discoideae-Astragaletum aegeici* Musarella, Brullo & Giusso ass. nov. *hoc loco* (Appendix C, Table A60, rel. 1–6).

Holotypus: Appendix C, Table A60, rel. 1, *hoc loco.*

Characteristic species: *Astragalus angustifolius* subsp. *aegeicus*, *Anthemis aciphylla* subsp. *discoidea*, *Allium stamineum s.l.*, *Silene lesbiaca*, *Paronychia macrosepala*.

Structure and ecology: The association is restricted to cacuminal stands at 900–967 m of altitude, on compact limestone with very superficial soils confined to the crevices of the rock. It is developed within the meso-Mediterranean bioclimatic belt on rocky surfaces usually well exposed and windy. In this vegetation small often thorny pulvini occur, among which a relevant physiognomic role is played by *Astragalus angustifolius* subsp. *aegeicus, Inula heterolepis, Noaea mucronata, Silene urvillei, Sideritis sipylea, Anthemis aciphylla* subsp. *discoidea,* mixed to which there are some caespitose grasses, such as *Festuca pseudosupina* and *Koeleria lobata*. It is clearly an edaphophilous vegetation closely related to very peculiar environmental conditions, such as eroded soils, marked winds and mist regime. These factors taken together do not allow a natural evolution of the vegetation towards more mature forms, such as *Pinus brutia* pine forest widespread in the surrounding areas.

Distribution: The association is exclusive of Mt. Olymbos in the island of Lesbos (Eastern Aegean).

*Diantho zonati-Astragaletum lesbiaci* Musarella, Brullo & Giusso ass. nov. *hoc loco* (Appendix C, Table A60, rel. 7–11).

Holotypus: Appendix C, Table A60, rel. 7, *hoc loco.*

Characteristic species: *Astragalus lesbiacus*, *Dianthus zonatus*, *Petrorhagia armerioides*.

Structure and ecology: The association is localized on outcrops of calcareous rocks characterized by very shallow and immature soils. Usually it grows on fairly flat surfaces at altitudes between 700 and 800 m. Physiognomically, it shows a coverage rather scattered characterized by small prostrate shrubs, representated mainly by *Astragalus lesbiacus*, *A. angustifolius* subsp. *aegeicus* and *Dianthus zonatus*. The association usually covers small surfaces interspersed with uncultivated or reforested areas.

Distribution: The association was observed only on Mt. Marathovounos in Chios island.

*Galio insularis-Thymetum sypilei* Musarella, Brullo & Giusso ass. nov. *hoc loco* (Appendix C, Table A60, rel. 12–16).

Holotypus: Appendix C, Table A60, rel. 15, *hoc loco.*

Characteristic species: *Thymus sipyleus*, *Minuartia attica* subsp. *idaea*, *Galium brevifolium* subsp. *insulare*, *Minuartia mesogitana* subsp. *kotschyana*, *Asyneuma virgatum* subsp. *cichoriforme*.

Structure and ecology: The association colonizes the rocky ridges very windy and washed away at altitudes above 1100 m. The vegetation is rather thinned out with small dwarf shrubs that grow in the cracks of rocks. The more frequent species in this vegetation are *Thymus sipyleus*, *Minuartia attica* subsp. *idaea*, *Galium brevifolium* subsp. *insulare, Minuartia mesogitana* subsp. *kotschyana*, *Festuca pseudosupina*, *Centaurea urvillei* subsp. *urvillei*, *Pterocephalus pinardii* and *Euphorbia herniariifolia*. This vegetation is very degraded and floristically impoverished mainly due to heavy grazing

Distribution: The association occurs on Mt. Pelineon in Chios.

*Acantholimo aegaei-Astragaletum lesbiaci* Musarella, Brullo & Giusso ass. nov. *hoc loco* (Appendix C, Table A60, rel. 17–21).

Holotypus: Appendix C, Table A60, rel. 17, *hoc loco.*

Characterstics species: Astragalus lesbiacum, Acantholimon aegaeum, Thymus zygioides.

Structure and ecology: The association occurs on the cacuminal limestone plateaux, where it colonizes washed rocky surfaces at altitudes between 800 and 900 m. It is characterized by the dominance of pulvinate tragacanthoid shrubs, such as *Astragalus lesbiacus*, *A. angustifolius* subsp. *aegeicus*, *Acantholimon aegaeum*, and *Silene urvillei*.

Distribution: The association was surveyed exclusively on Mt. Plakes in Chios island.

*SESLERIO ACHTAROVII-ANTHEMIDION TENUILOBAE* Musarella, Brullo & Giusso all. nov. *hoc loco.*

Holotypus: *Paronychio bornmuelleri-Astragaletum odoniani* Musarella, Brullo & Giusso ass. nov. *hoc loco.*

Characteristic species: *Anthemis tenuiloba*, *Festuca hirtovaginata*, *Galium insulare*, *Inula aschersoniana* var. *athoa*, *Satureja montana* subsp. *macedonica*, *Sesleria achtarovii*.

Structure and ecology: This alliance brings plant communities linked to carbonatic substrates of mountain and high-mountain stands, dominated by thorny pulvini. This vegetation is distributed at altitudes above 900 m, where it is localized in places usually represented by summit rocky plateaux, ridges and cacuminal areas with very superficial and immature soils, present mainly in small depressions and crevices. From the bioclimatic viewpoint, this alliance is distributed within the meso-Mediterranean belt, with ombrotype more or less humid, even during the summer, penetrating probably in that supra-Mediterranean one. Floristically, it is differentiated by a set of endemic species with North Aegean distibution.

Distribution: The alliance is currently known only for the island of Thassos in the northen Aegean, but based on the geographic distribution of characteristic species, problably it occurs also in the coastal mountains of North Greece.

Notes: This syntaxon can be considered as a northern vicariant of the other two alliances included in the *Noaeo mucronatae-Silenetalia urvillei* previously described.

*Paronychio bornmuelleri-Astragaletum odoniani* Musarella, Brullo & Giusso ass. nov. *hoc loco* (Appendix C, Table A61).

Holotypus: Appendix C, Table A61, rel. 1, *hoc loco.*

Characteristic species: *Astragalus angustifolius* subsp. *odonianus*, *Paronychia bornmuelleri*, *Cerastium moesiacum* subsp. *glutinosum*, *Allium cremnophilum*, *Dianthus gracilis* subsp. *xanthianus*, *Minuartia verna* var. *thasia*.

Structure and ecology: The association is confined to the summit very windy and sunny plateaux, consisting of crystalline limestones, located at altitudes between 900 and 1000 m, falling within the bioclimatic meso-Mediterranean belt. It occurs on rocky substrates with very superficial and immature soils, reaching its maximum expression in situations of ridge. The vegetation is dominated by thorny pulvini of *Astragalus angustifolius* subsp. *odonianus,* which forms large populations mixed with small prostrate chamaephytes (*Dianthus gracilis* subsp. *xanthianus, Minuartia verna* var. *thasia, Paronychia bornmuelleri, Cerastium moesiacum* subsp. *glutinosum*) and several hemicriptophytes represented mainly by caespitose grasses (*Festuca hirtovaginata, Sesleria achtarovii, Koeleria lobata, Stipa endotricha*). This vegetation is typically edaphophile, since it is linked to peculiar environmental conditions that do not allow the normal development of the soil. In edaphic more mature situations, the association is usually replaced by *Juniperus excelsa* woodlands.

Distribution: The association is confined to rocky outcrops of the cacuminal stands of Mt. Ipsario in the island of Thassos (northern Aegean).

## 3. Materials and Methods

The methodology used for the study regarding this kind of orophilous vegetation was based on a careful analysis of the diagnostic components that characterize the biotic and abiotic landscape of the investigated area.

The 680 phytosociological relevés (460 unpublished and 220 from literature), carried out during the spring-summer of the several years (2003, 2004, 2005, 2006, 2007, 2008, 2011, 2019) according to the sigmatist method of Zürich-Montpellier school [88,89], allowed for the definition of the main vegetation typologies with the identification of many different plant communities, for whose correct syntaxonomic arrangement and the phytosociological nomenclature code was followed [90]. The literature data refer to the contributions of several authors who carried out phytosociological investigations on the mountain ranges included in this study [35,38,40,41,42,44]. All the identified syntaxa were analyzed from nomenclatural, floristic, structural, ecological, chorological and syndynamic point of view. With regard to single associations, these are provided with a phytosociological table in which the unpublished relevés are complemented by literature data after floristic update. For the identification of the plants listed in phytosociological relevés, several Balkan, European, and Mediterranean floras were used, while for the floristic nomenclature we were based on the most significant floras, checklist and taxonomic monographs regarding genera and critical groups. The main works consulted were: Boissier [91], Halácsy [92], Hayek [93], Tutin et al. [94,95], Davis [96], Cristodoulakis and Georgiadis [97], Greuter et al. [98], Scholz [99], Strid [73,74], Strid and Tan [75,76,77], Strasser [100], Tan et al. [70], Greuter [101], Krendl [102], Podlech and Zarre [103], Dimopoulos et al. [71], “Flora of Greece web” [103], and other regarding particular species [104,105]. The floristic list obtained from phytosociological relevés is reported in Appendix A (Table A1) and were used for the phytogeografic processing.

As regards the bioclimatic investigations, the classification of Rivas-Martínez [64] was followed, based on the thermopluviometric data by this author. In particular, the charts built according to the criteria proposed by Walter and Leith [67] are provided, using the extrapolation data according to the method of Hijmans et al. [68,69], which are listed in the “Global climate surfaces” and relate to the period 1950–2000. These data have been taken from a map grid of 10 km^2^, in which the toponym is not given but only the geographical coordinates of the centroid of the square.

For the taxonomic treatment of the new species and subspecies described in this work, the study is based on floristic collections carried out in the investigated territories, integrated by herbarium and literature data in order to clarify their morphological relationships. As regards the taxonomic approach, the international code of botanical nomenclature [106] was followed.

## 4. Conclusions

This study allowed to improve the knowledge on the orophilous pulvinate vegetation occurring in the high-mountains of continental and insular Greece. These plant communities probably dating back to the Messinian (late Miocene) following the desiccation of the Mediterranean basin, since they are featured by steppic species, that currently have their greatest diffusion in the Irano-Turanian region. In particular, these species having usually a cushion-like habit, often thorny, seem to have penetrated in the Mediterranean after the drying up of the climate, which led to climatically challenging and very harsh environmental conditions unfit for the pre-existing flora.

It is a very peculiar and phisiognomically well characterized vegetation, very rich in endemics represented mainly by pulvinate chamaephytes and nanophanerophytes as well as often by dominance of hemicryptophytes. Most of the endemic species have a relict distribution and belong to the ancient tertiary flora, which gives a remarkable phytogeographic significance to this kind of vegetation.

Compared to the previous syntaxonomic scheme proposed by Quézel [35], nomenclaturally updated by Quézel et al. [80] and more recently taken up by Mucina et al. [84], which did not provide clear information on the classification of the plant communities present in the cacuminal stations of the Greek mountains, a new treatment is proposed in this study, based above all on the phytogeographic role of endemic species and not on the altimetric ranges, at least as regards the alliances. On the whole, this new class, namely *Cerastio candidissimi-Astragaletea rumelici*, replacing the previous *Daphno-Festucetea* which must be considered as an ambiguous name, represent a geographical vicariant in Greece and Aegean area of other syntaxa already kwown in literature [2,22,31,45,48,51]. Such cases are the following: *Carici-Genistetea lobelii* Klein 1972 in Sardinia and Corsica; *Rumici-Astragaletea siculi* Pignatti & Nimis in Pignatti et al. 1980 in Sicily and Calabria; *Saturejetea spinosae* Zaffran 1990 in Crete; *Diantho troodi-Teucrietea cyprii* Brullo, Giusso & Guarino 2005 in Cyprus; and *Astragalo-Brometea* Quézel 1973 in Anatolia and Lebanon.

There are numerous problems related to the conservation of these high mountain vegetation aspects. The most important are the anthropogenic pressure, due to grazing, especially goats, and the landslide of some areas which makes them particularly inconsistent, and this causes continuous erosions of various strips of vegetation. If on the one hand, thorny pulvins are not eaten by grazing animals, it is also true, however, that their presence leads continuous trampling and excessive eutrophication.

Furthermore, the ongoing climate change will certainly have a further negative influence on these peculiar high mountain plant communities and can promote a change in species strategies and growth form [107]. In fact, increasing temperatures will result in less water availability at ever higher altitudes, resulting in the impossibility for the plant communities to be able to survive using their environmental adaptations, such as spinescence, pulvinate habit, etc. All this could involve changes in the vegetation typology, with a progressive replacement of the hitherto predominant angiosperms with dwarf gymnosperms, as species of *Juniperus* [108,109].

For all these reasons, a strictly ecological approach could provide more detailed information on the role that these plant communities have within the entire ecosystem of the high mountains involved in this study. This research related to conservation biology could be used mainly for protection policy.

## Figures and Tables

**Table 1:** Life forms of the investigated orophilous flora (from [70,71,72,73,74,75,76,77]).

Life Form	n.	%
**Ch total**	**221**	**34.86**
Ch caesp	7	1.10
Ch frut	28	4.42
Ch pulv	45	7.10
Ch rept	9	1.42
Ch succ	9	1.42
Ch suffr	123	19.40
**G total**	**62**	**9.78**
G bulb	42	6.62
G rhiz	20	3.15
**H total**	**273**	**43.06**
H bienn	13	2.05
H caesp	105	16.56
H rept	13	2.05
H rhiz	3	0.47
H ros	30	4.73
H scap	109	17.19
**T total**	**58**	**9.15**
T scap	58	9.15
**NP**	**17**	**2.68**
**P**	**3**	**0.47**
**TOTAL**	**634**	**100**

**Table 2:** Chorotypes of the investigated orophilous flora (from [70,71,72,73,74,75,76,77]).

Chorotypes	N.	%
**Wide distribution**		
**total**	**35**	**5.52**
cosmop	9	1.42
circumboreal	3	0.47
paleotemp	23	3.63
**Europeans**		
**total**	**74**	**11.67**
european	10	1.58
eurasian	20	3.15
euro-siberian	3	0.47
euro-medit	32	5.05
euro-medit-irano-turan	9	1.42
**Mediterraneans**		
**total**	**269**	**42.43**
circum-medit	61	9.62
E-medit	188	29.65
N-medit	10	1.58
medit-irano-turan	9	1.42
medit-asian	1	0.16
**Endemics**		
**total**	**256**	**40.38**
end Balkan	58	9.15
end Greece	39	6.15
end NC Greece	15	2.37
end CS Greece	48	7.57
end Sterea Ellas	16	2.52
end Peloponnese	45	7.10
end Euboea	6	0.95
end Ionian islands	7	1.10
end E-Aegean	15	2.37
end N-Aegean	7	1.10
**TOTAL**	**634**	**100**

**Table 3:** Endemic chorotypes of the investigated orophilous flora.

Chorotype	n.	%
end Balkan	58	22.66
end CS Greece	48	18.75
end Peloponnese	45	17.58
end Greece	39	15.23
end Sterea Ellas	16	6.25
end NC Greece	15	5.88
end E-Aegean	14	5.86
end Ionian islands	7	2.73
end N-Aegean	7	2.73
end Euboea	6	2.34
**TOTAL**	**256**	**100**

**Table A2:**
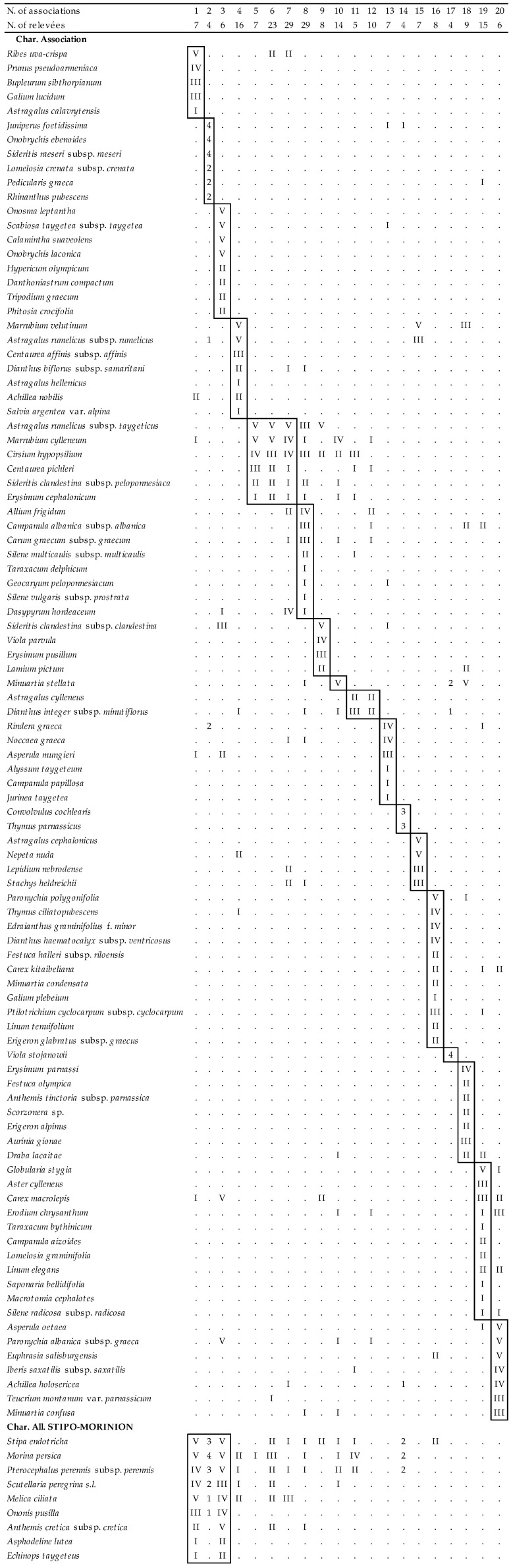

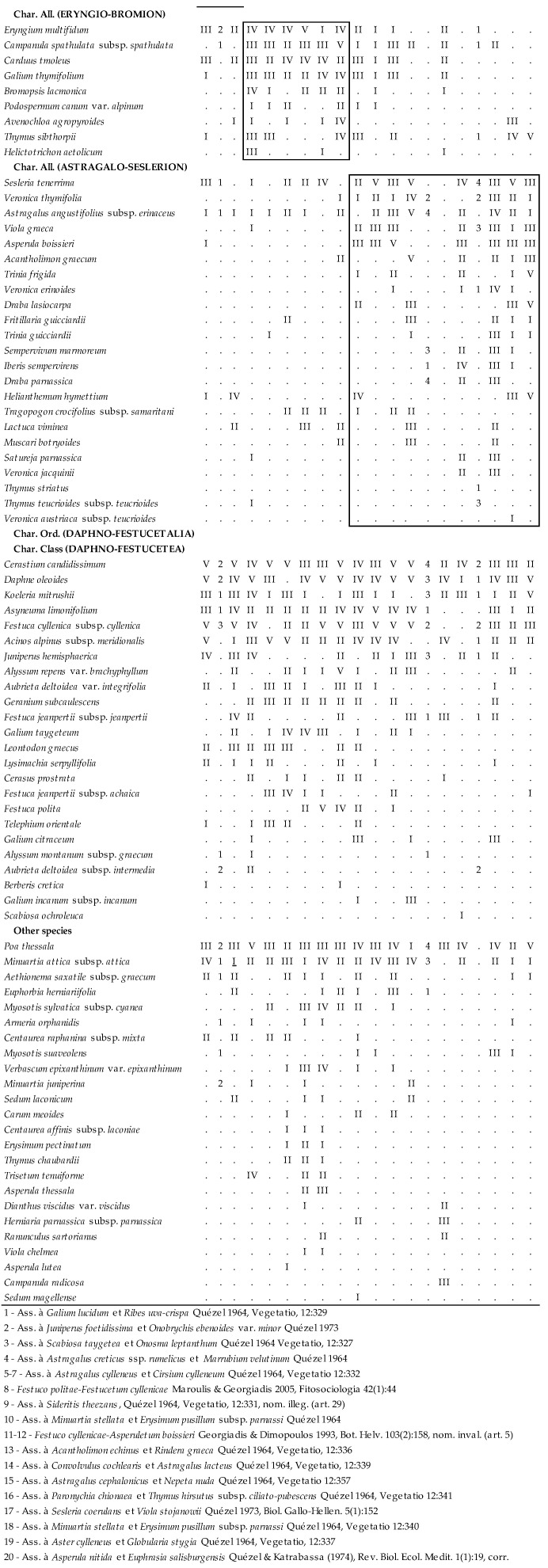
Synoptic table of the old associations arranged according to Quézel [35] traitment.

**Table A3:**
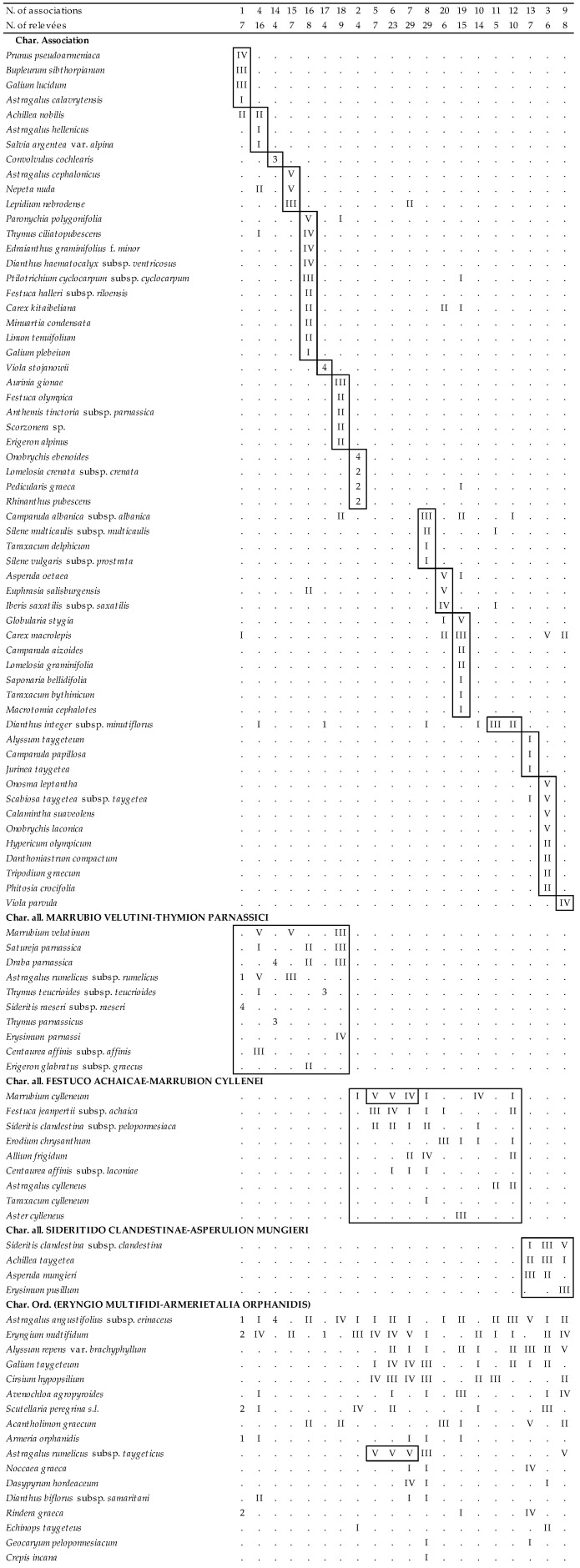

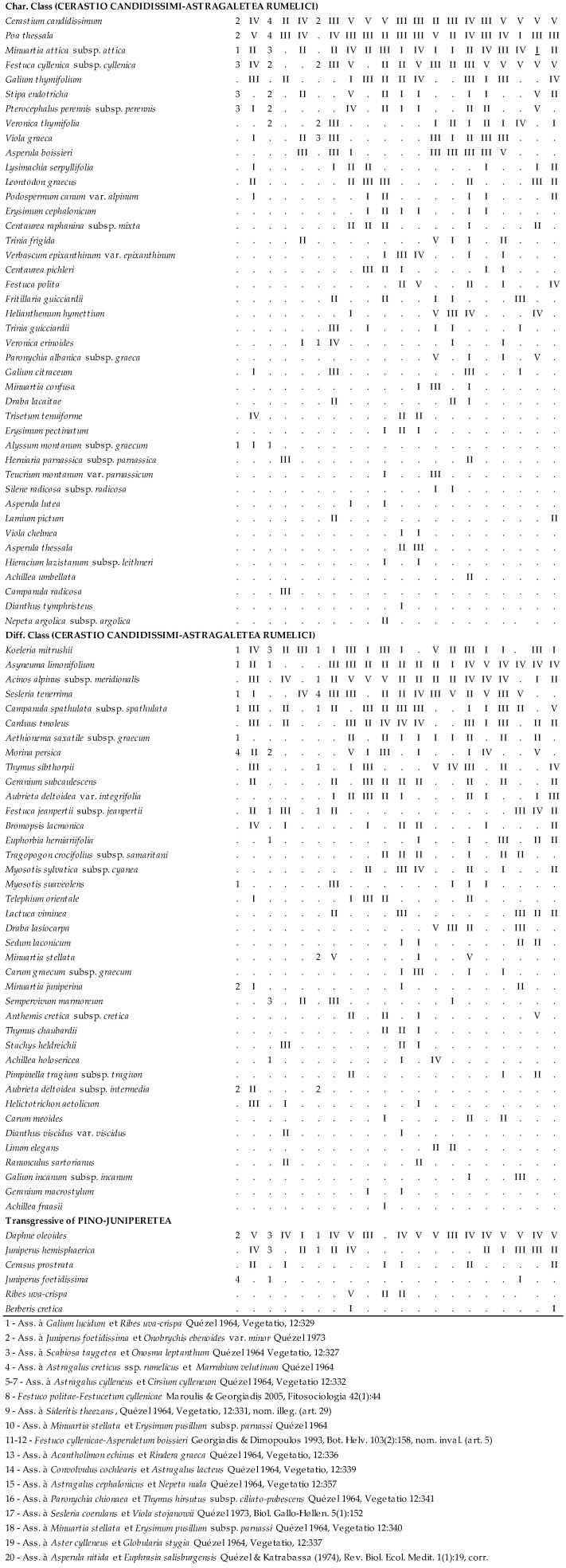
Synoptic table of the old associations reported in Table A2 arranged according to the new syntaxonomical traitment.

**Table A4:**
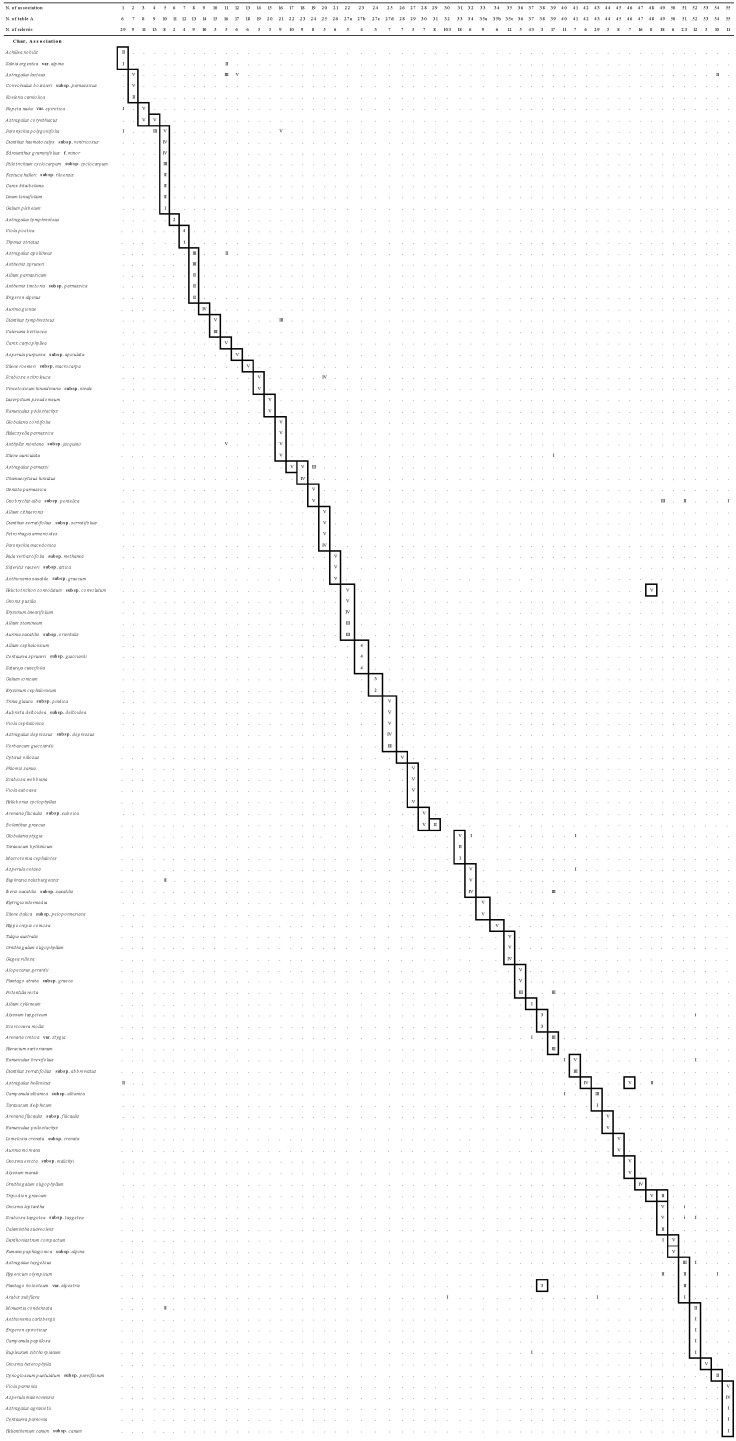

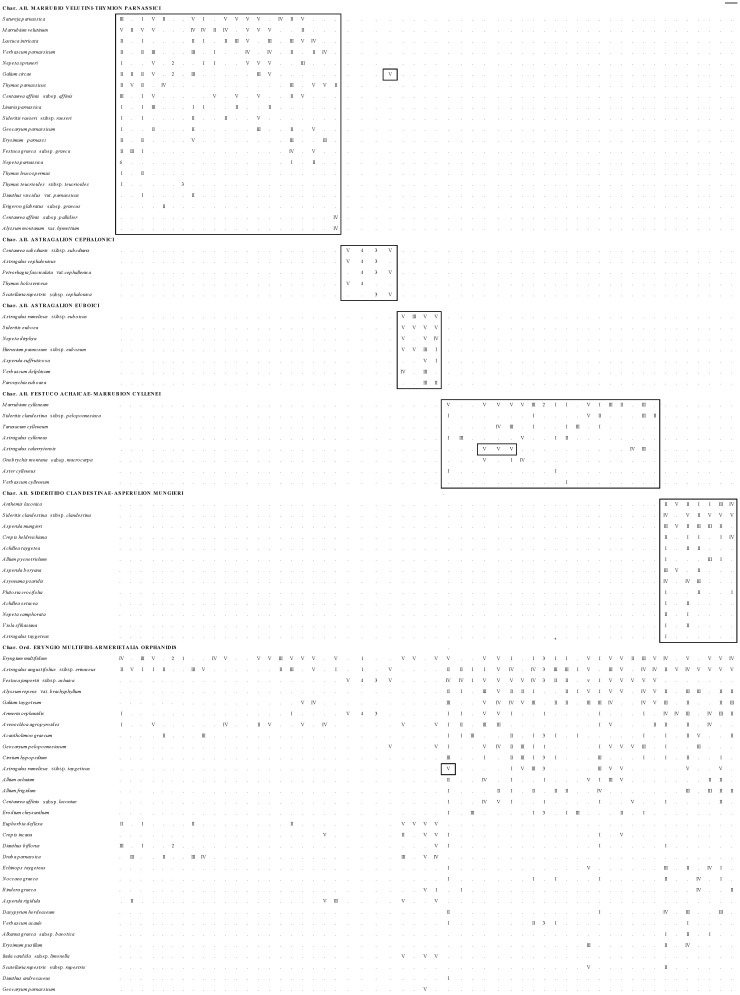

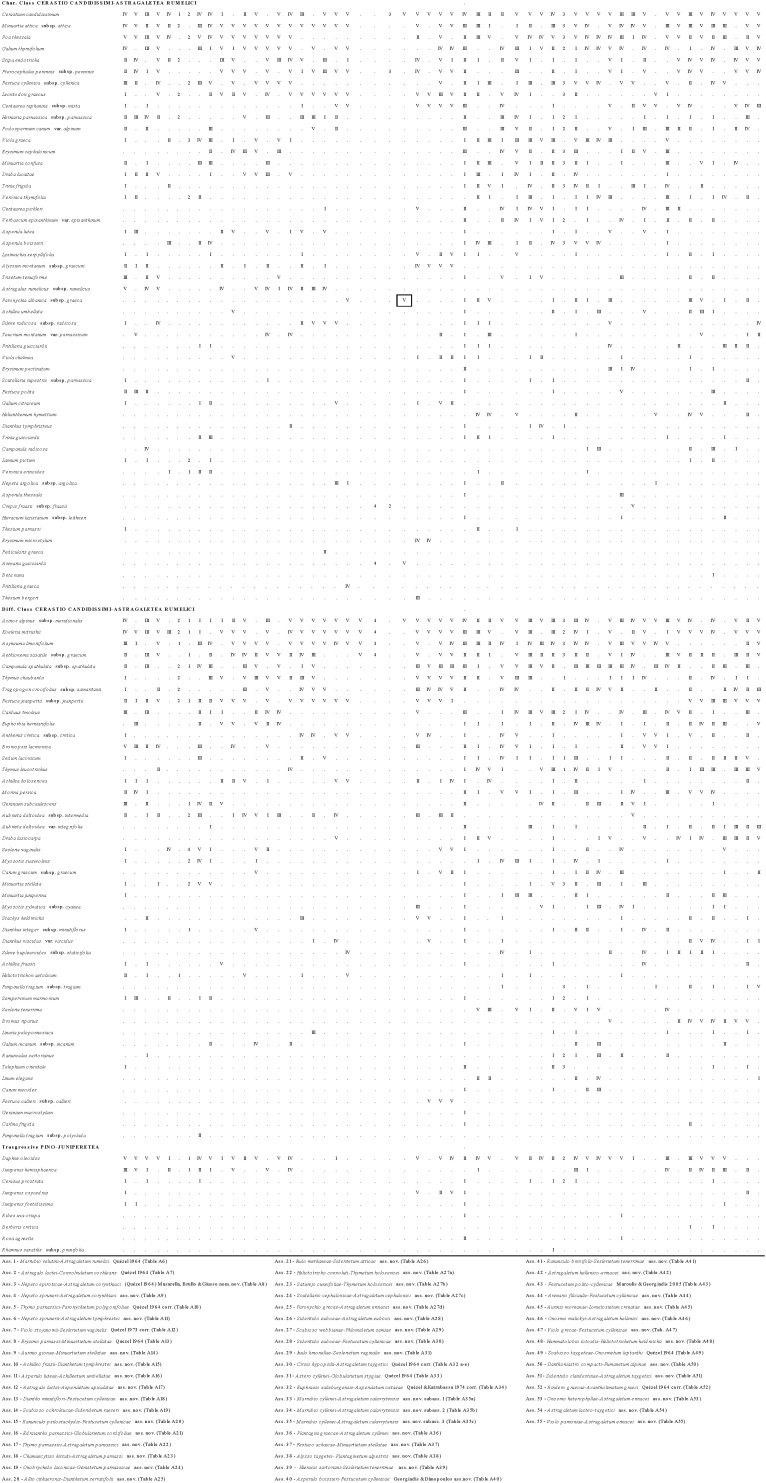
Synoptic table of the associations belonging to *Eryngio multifidi-Armerietalia orphanidis*.

**Table A5:**
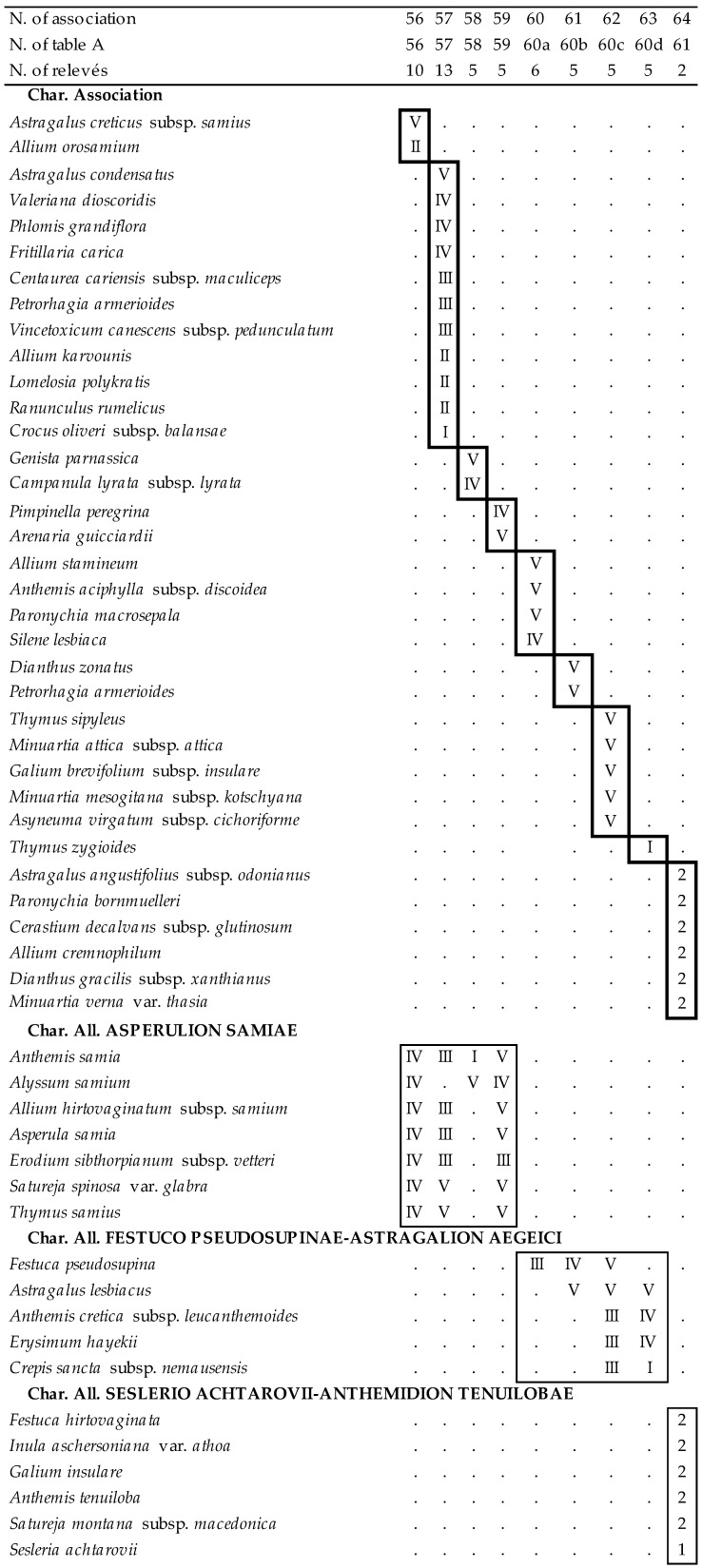

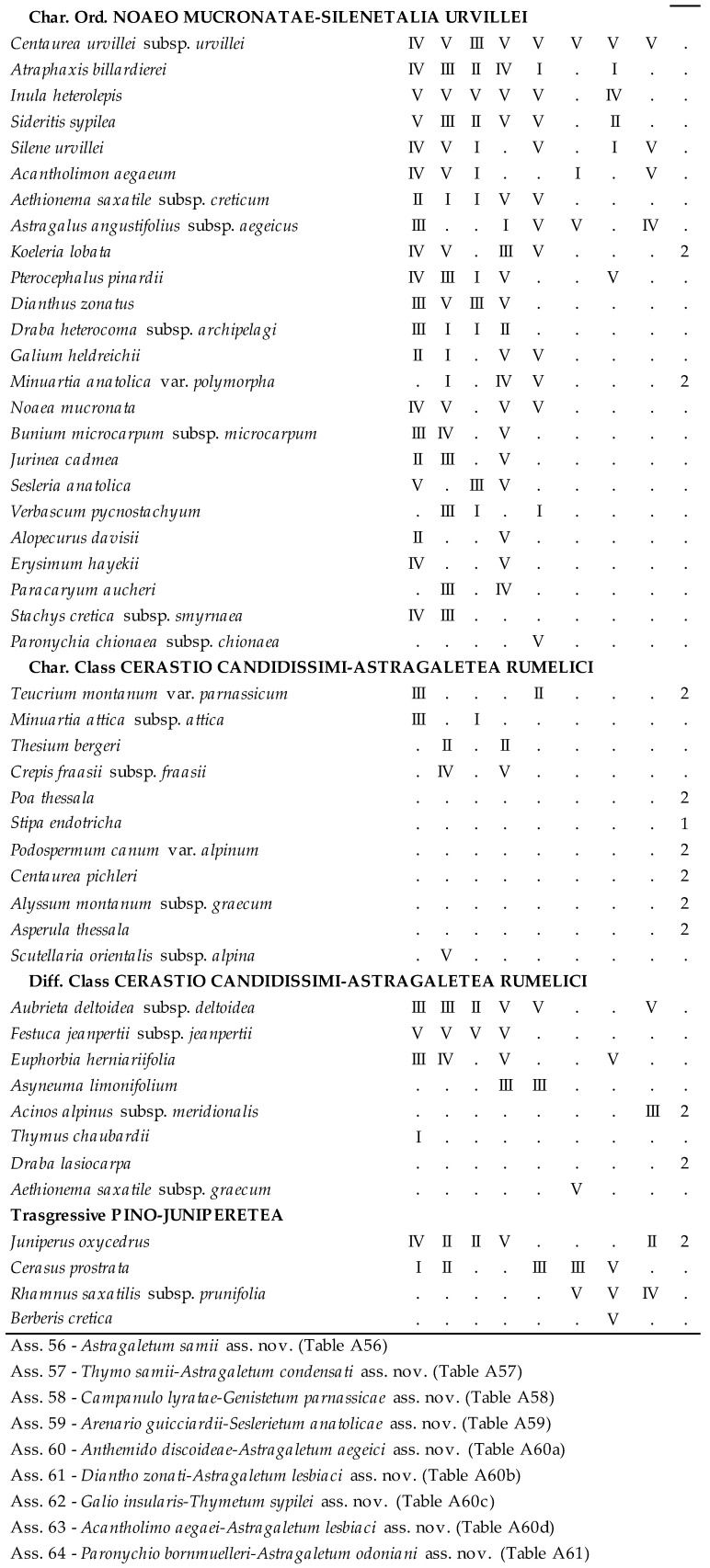
Synoptical tables of the associations belonging to *Noaeo mucronatae-Silenetalia urvillei* order.

**Table A6:**
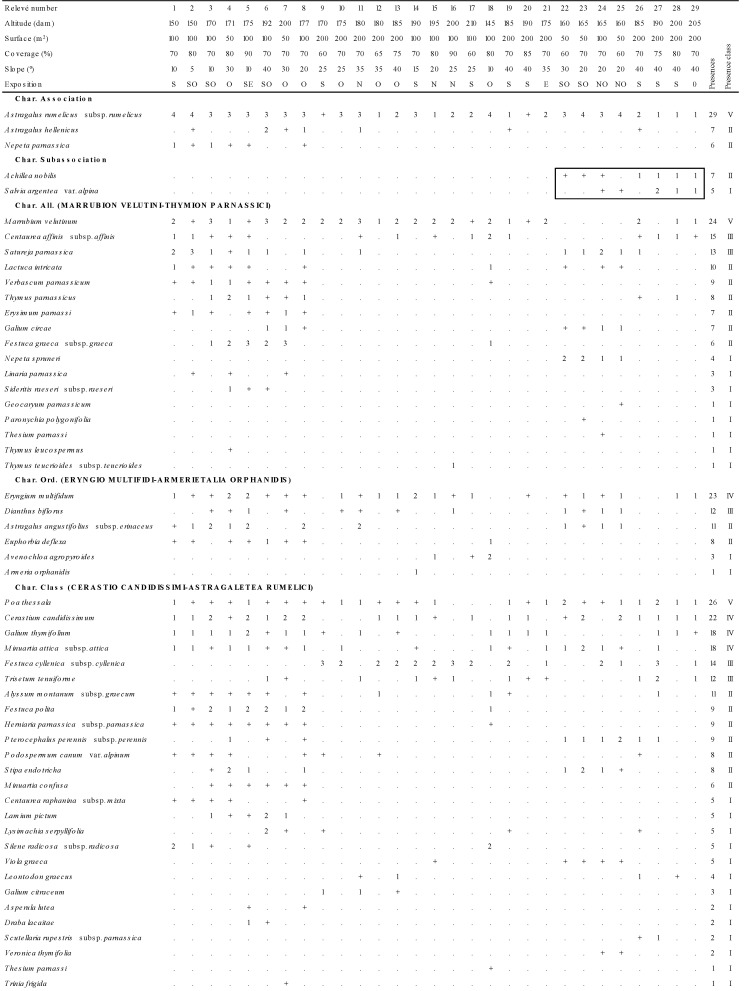

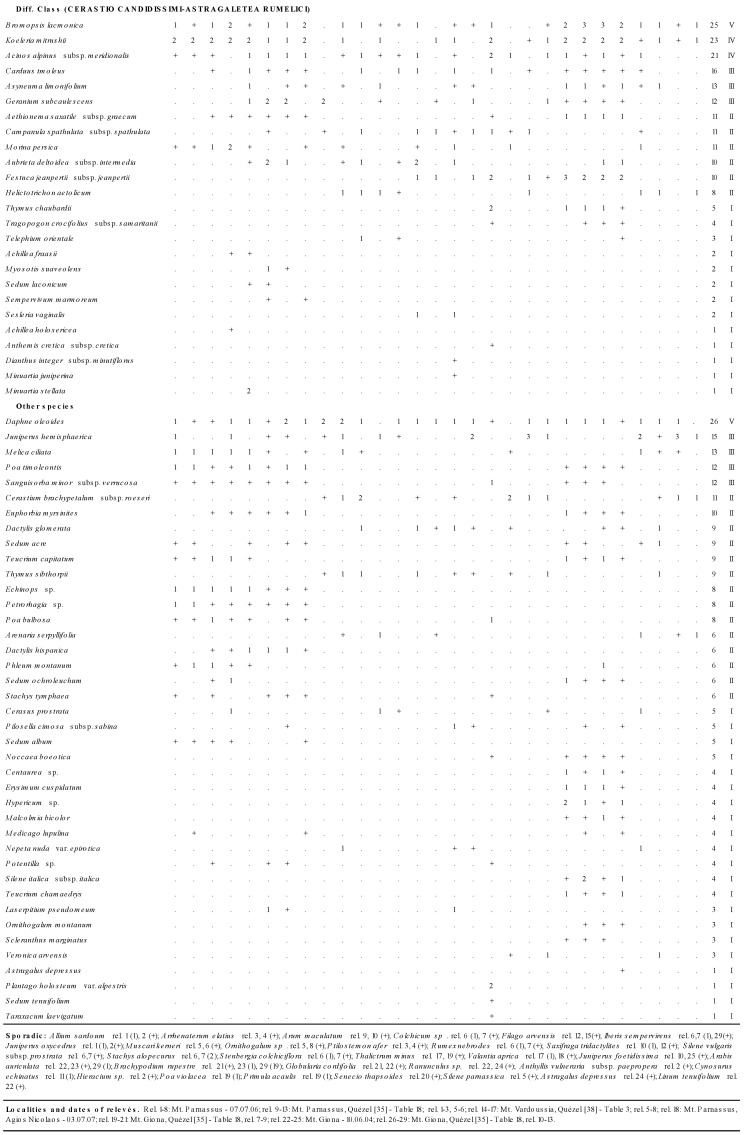
Marrubio velutini-Astragaletum rumelici Quézel 1964.

**Table A7:**
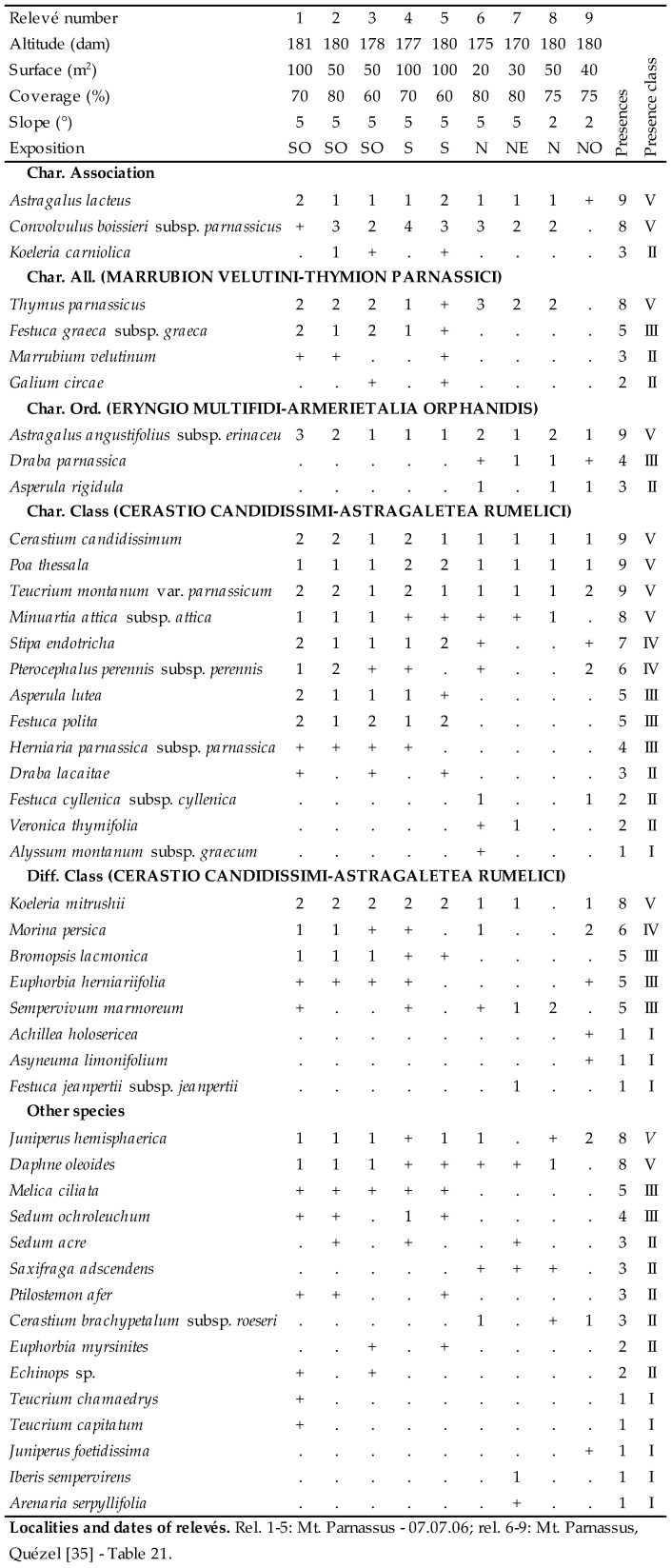
Astragalo lactei-Convolvuletum cochlearis Quézel 1964.

**Table A8:**
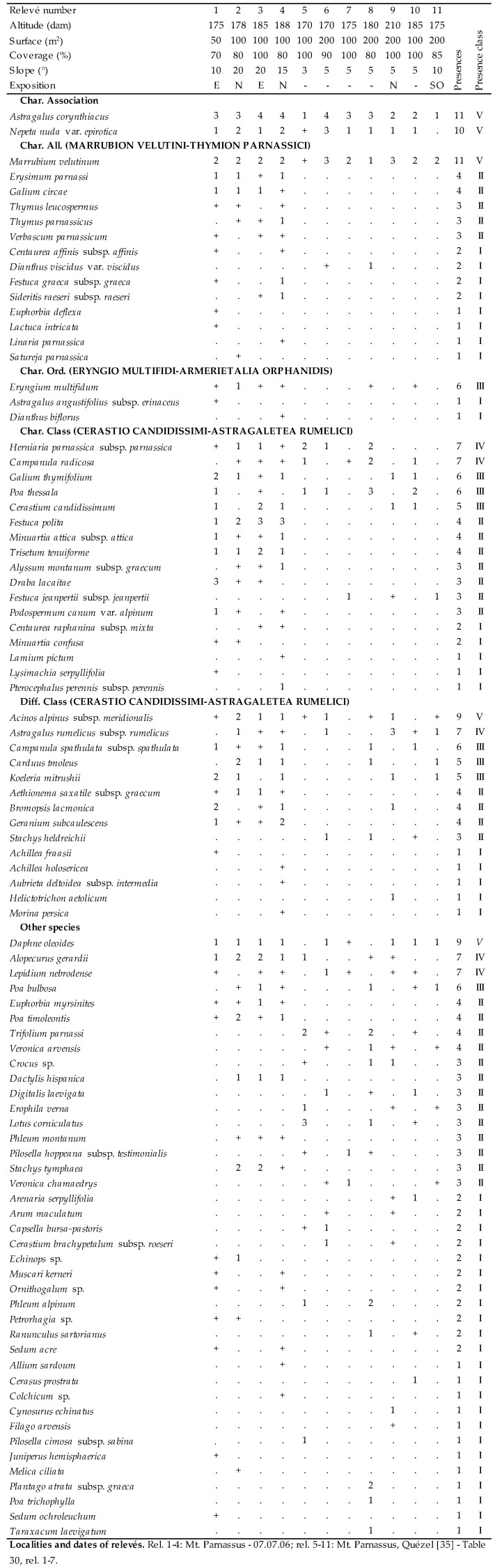
*Nepeto epiroticae-Astragaletum corynthiaci* (Quézel 1964) Musarella, Brullo & Giusso nom. nov.

**Table A9:**
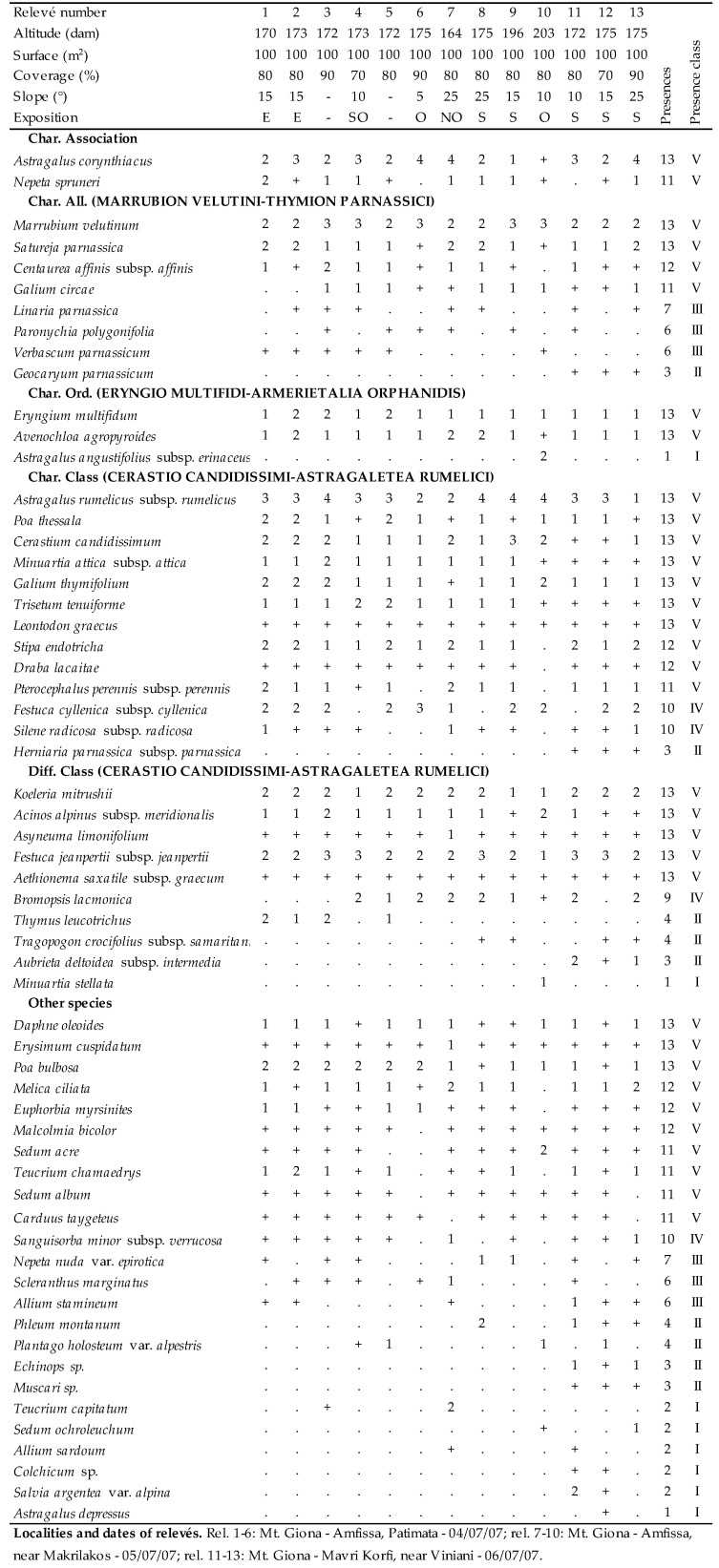
*Nepeto spruneri-Astragaletum corynthiaci* Musarella, Brullo & Giusso ass. nov.

**Table A10:**
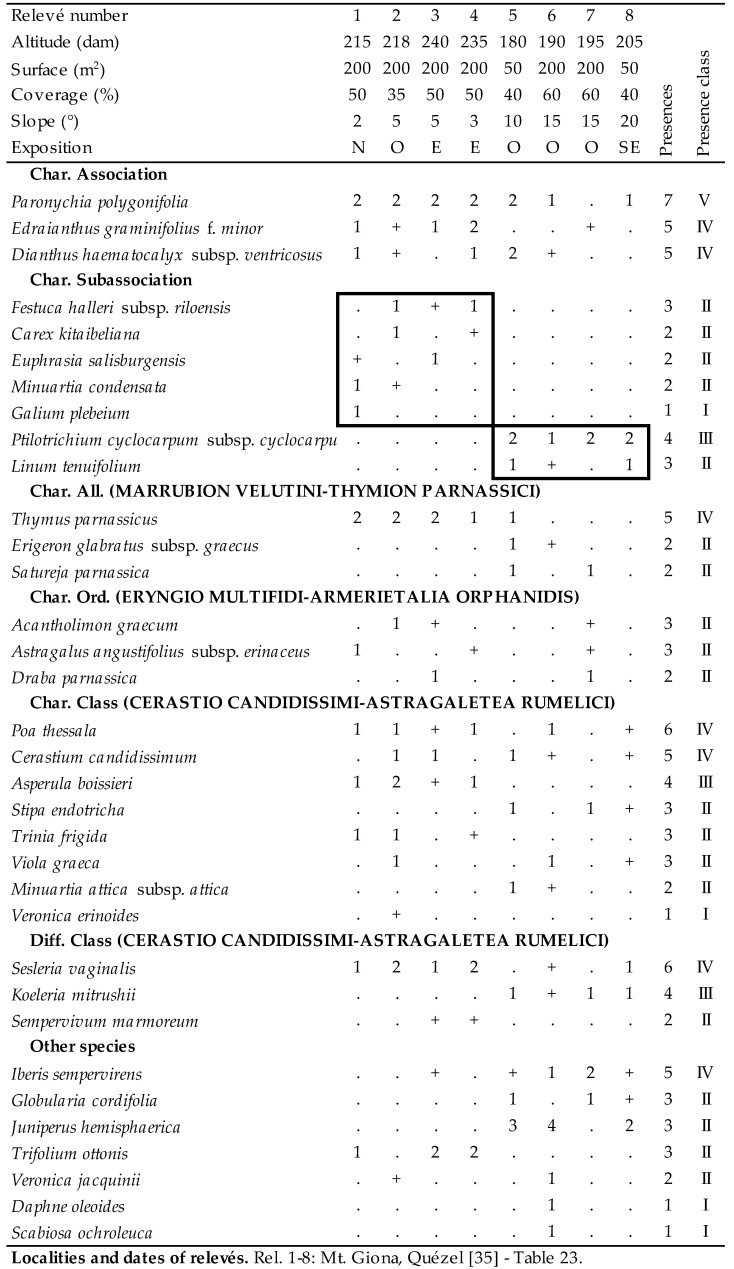
Thymo parnassici-Paronychietum polygonifoliae Quézel 1964 corr.

**Table A11:**
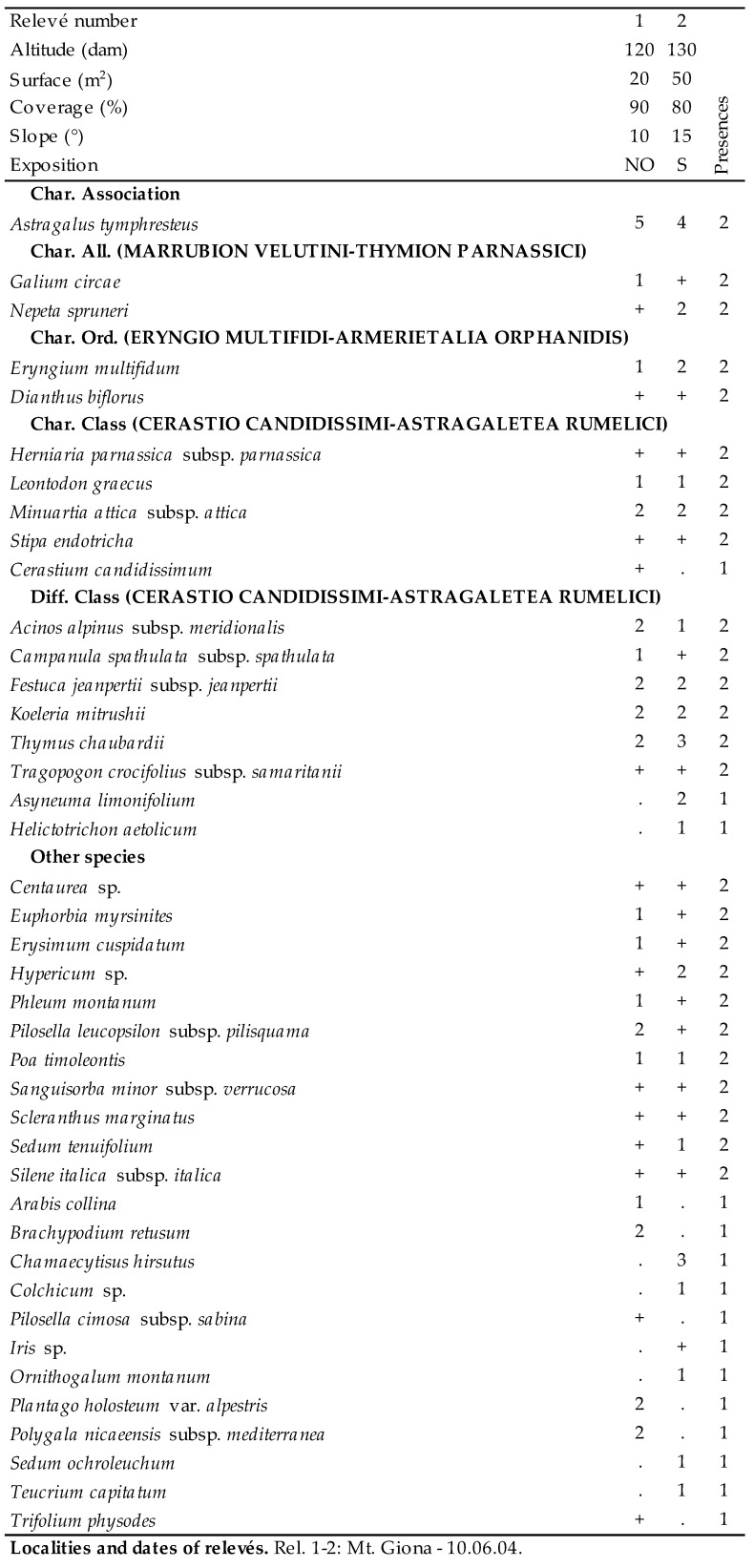
*Nepeto sprunerii-Astragaletum tymphrestei* Musarella, Brullo & Giusso ass. nov.

**Table A12:**
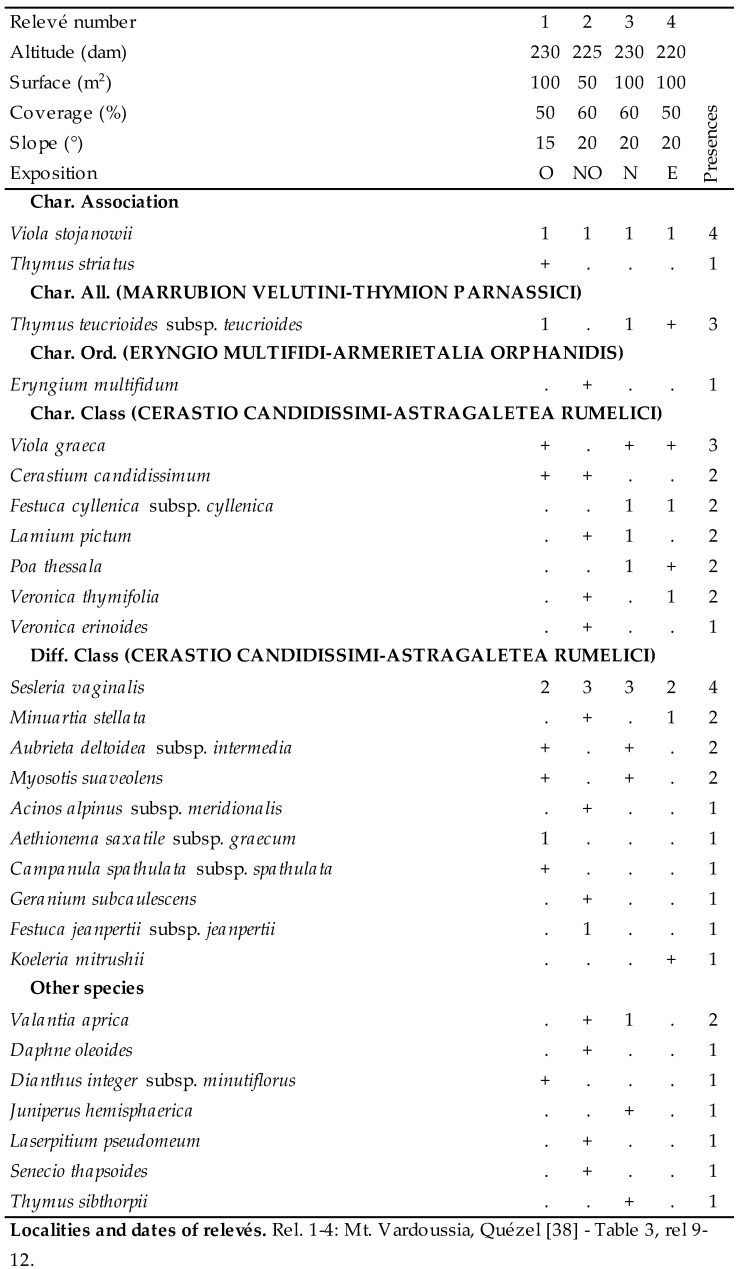
Violo stojanowii-Seslerietum vaginalis Quézel 1973 corr.

**Table A13:**
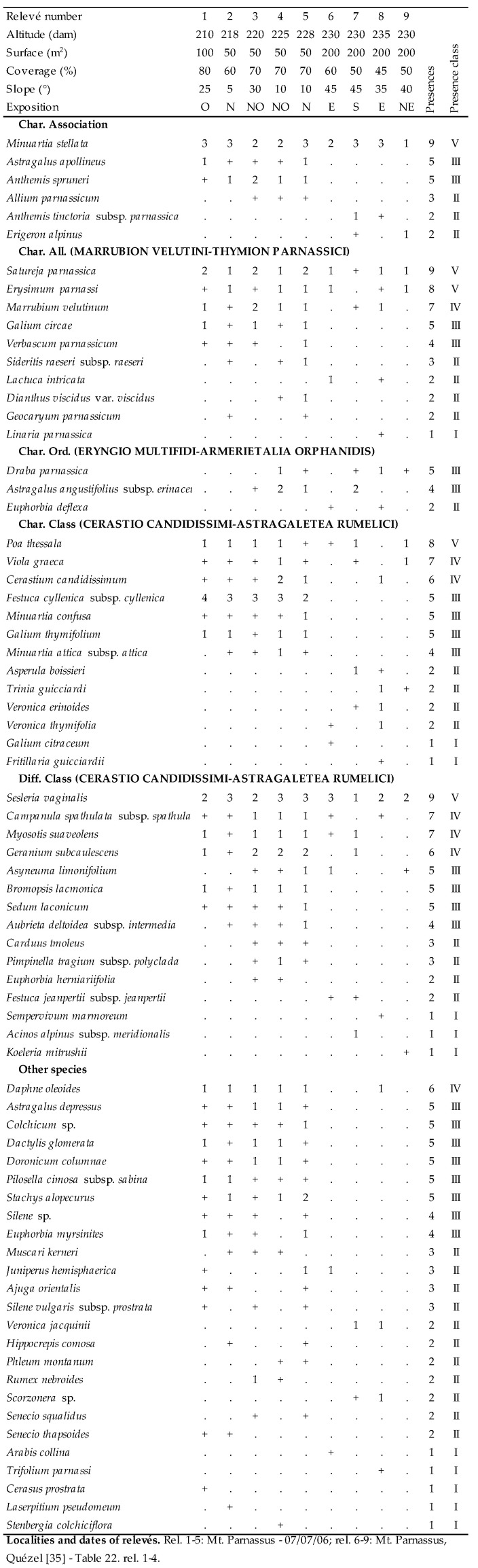
Erysimo parnassi-Minuartietum stellatae Quézel 1964.

**Table A14:**
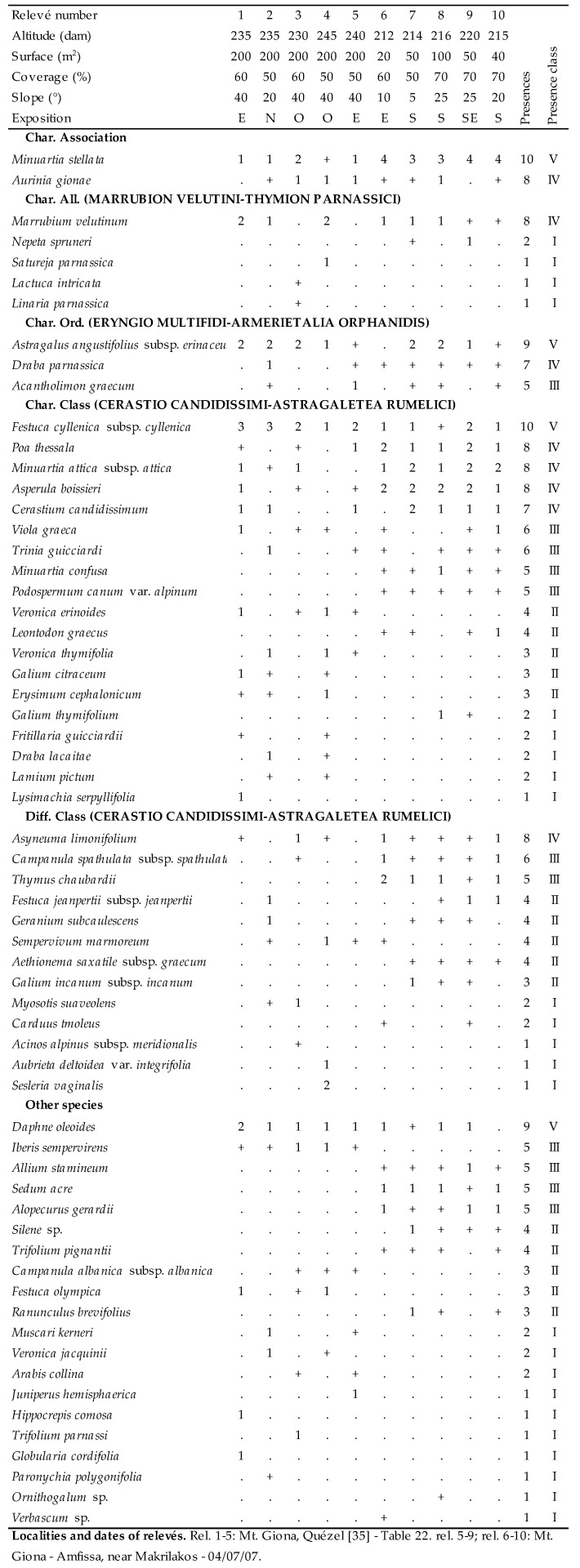
*Aurinio gionae-Minuartietum stellatae* Musarella, Brullo & Giusso ass. nov.

**Table A15:**
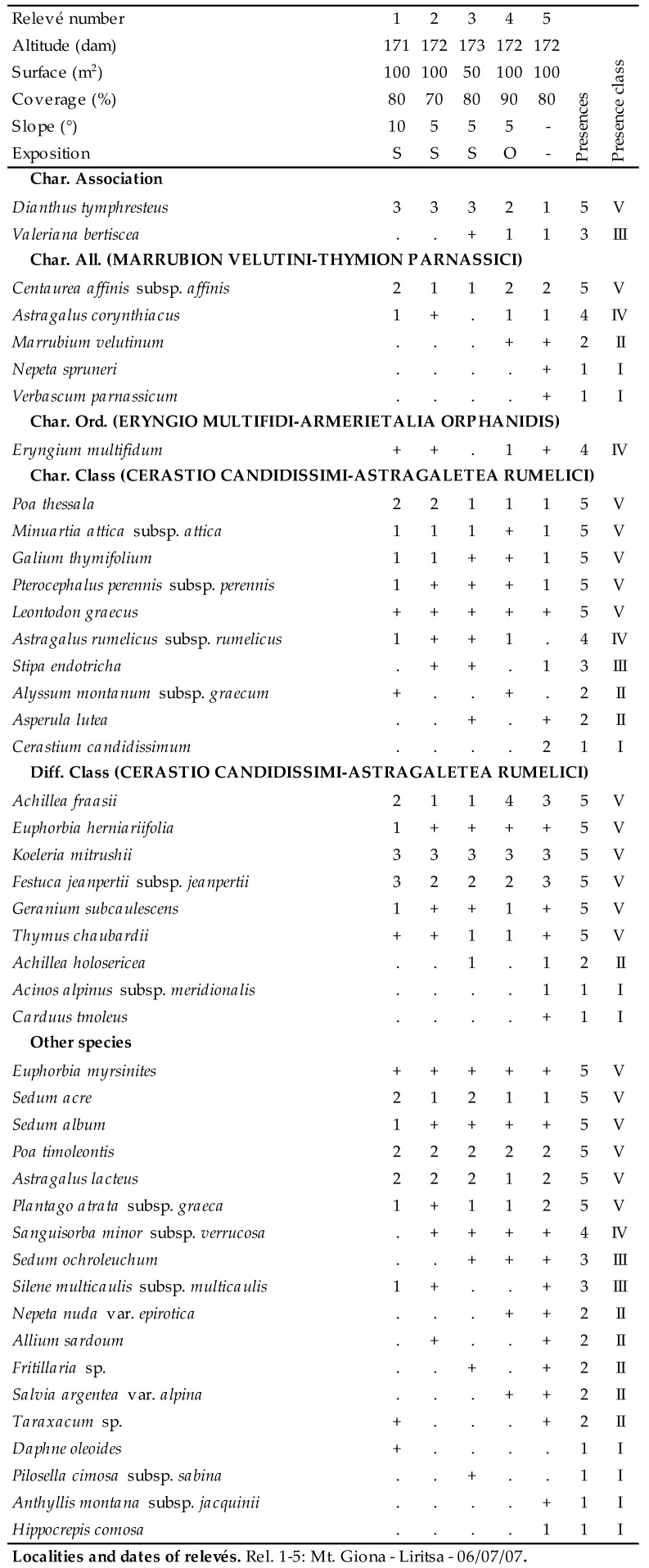
*Achilleo fraisii-Dianthetum tymphrestei* Musarella, Brullo & Giusso ass. nov.

**Table A16:**
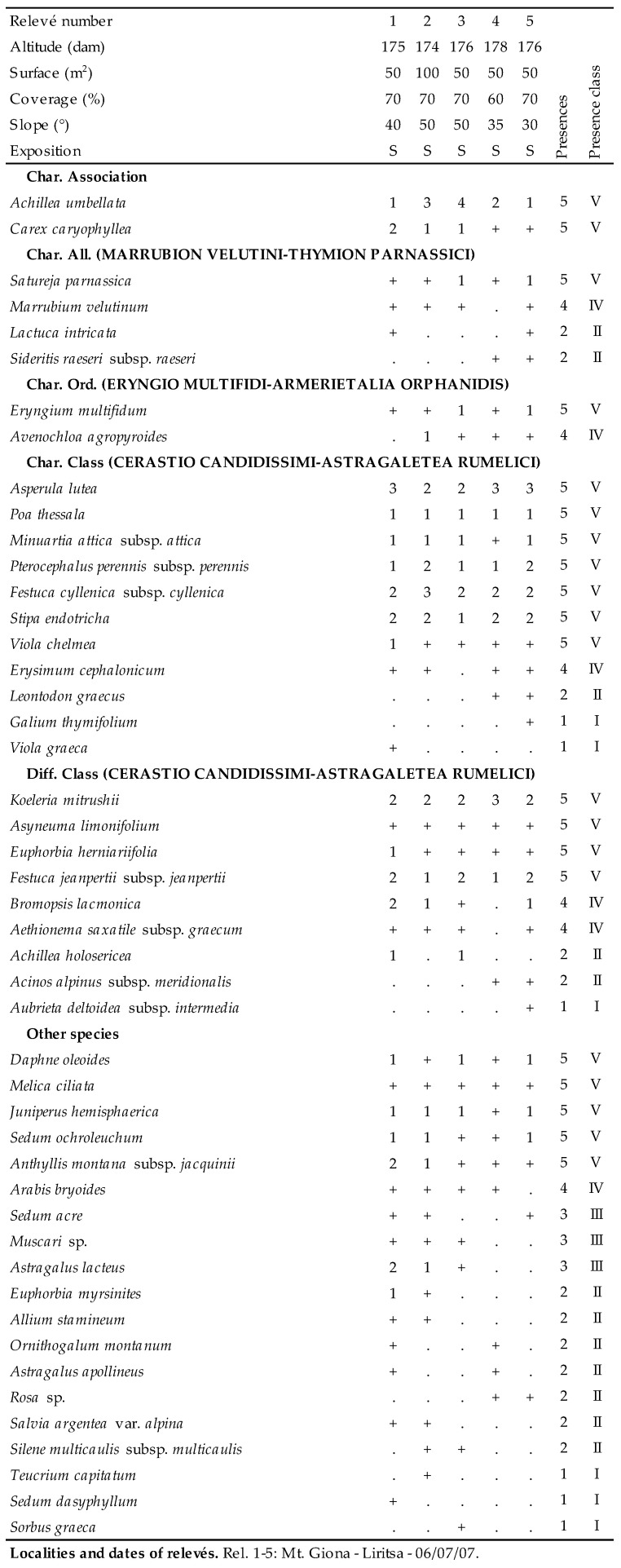
*Asperulo luteae-Achilleetum umbellatae* Musarella, Brullo & Giusso ass. nov.

**Table A17:**
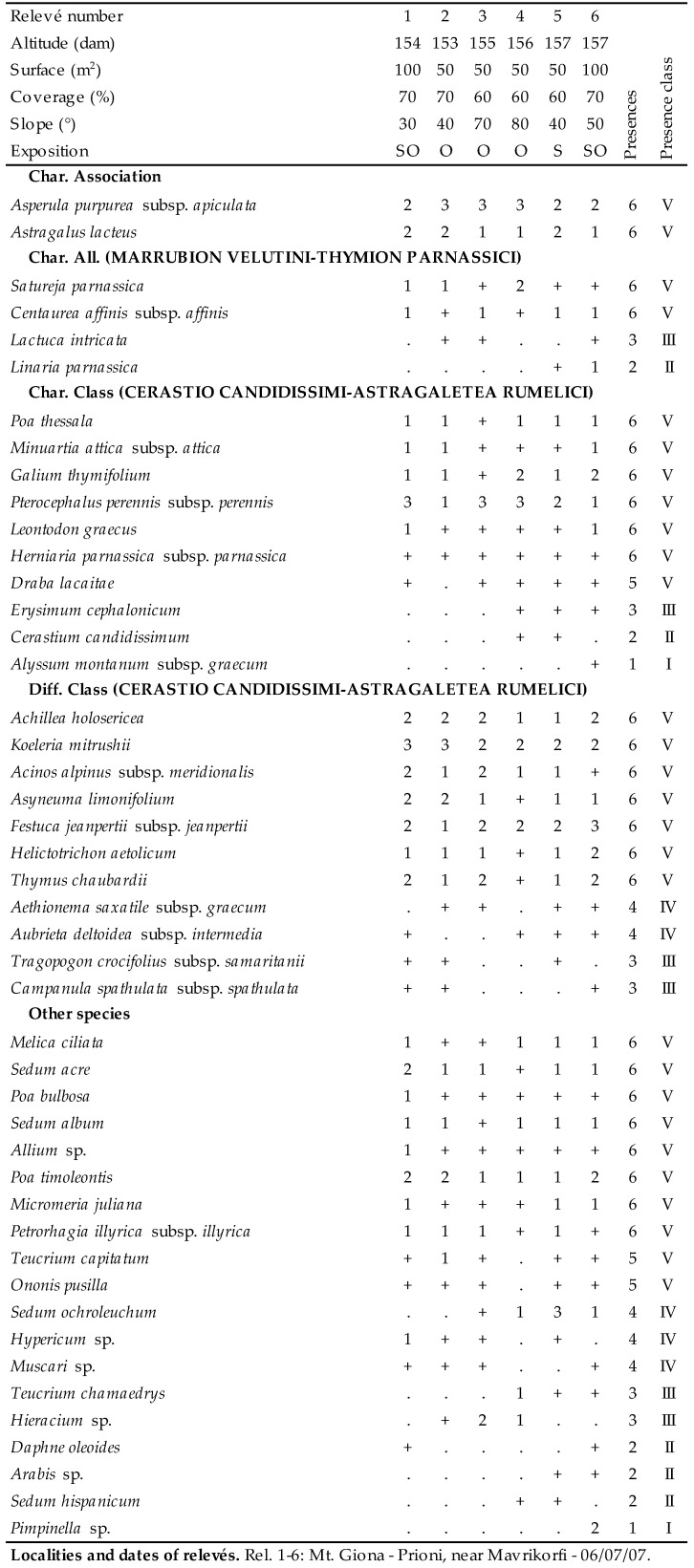
*Astragalo lactei-Asperuletum apiculatae* Musarella, Brullo & Giusso ass. nov.

**Table A18:**
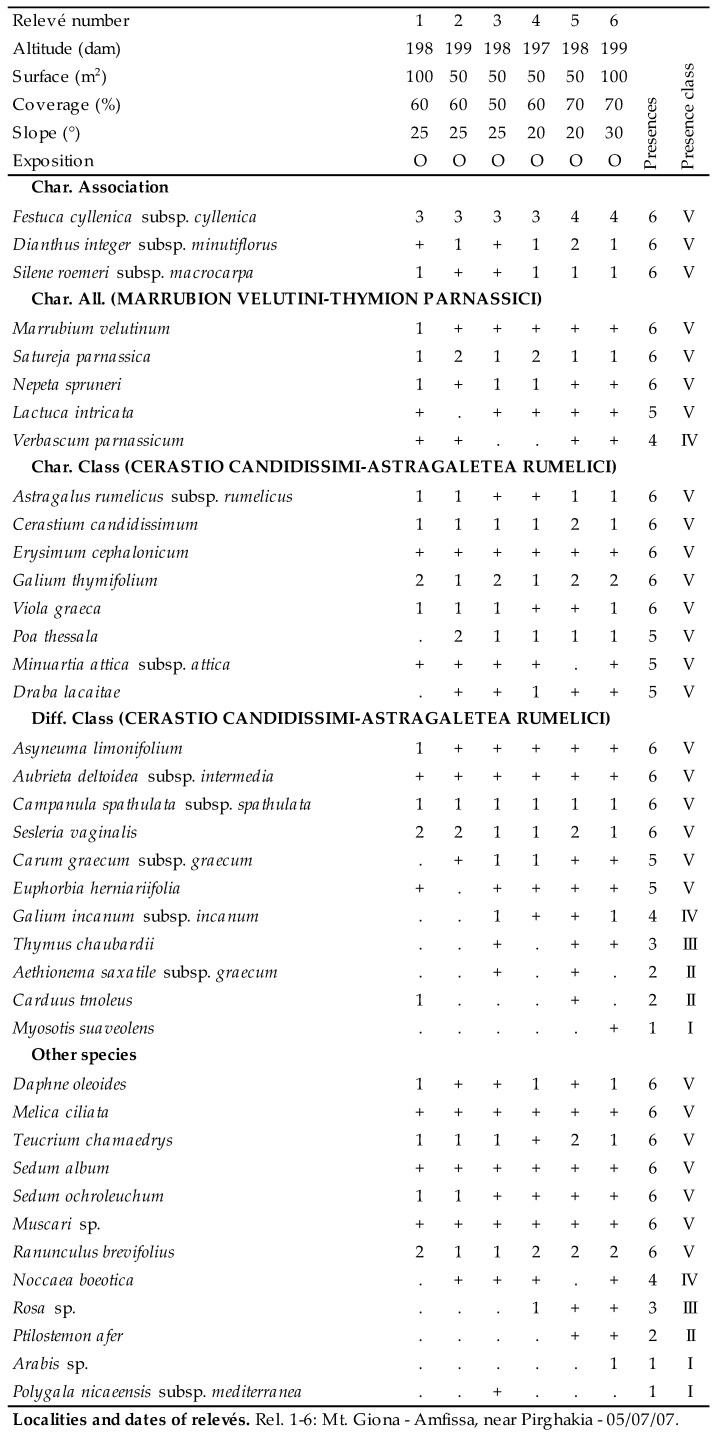
*Diantho minutiflori-Festucetum cyllenicae* Musarella, Brullo & Giusso ass. nov.

**Table A19:**
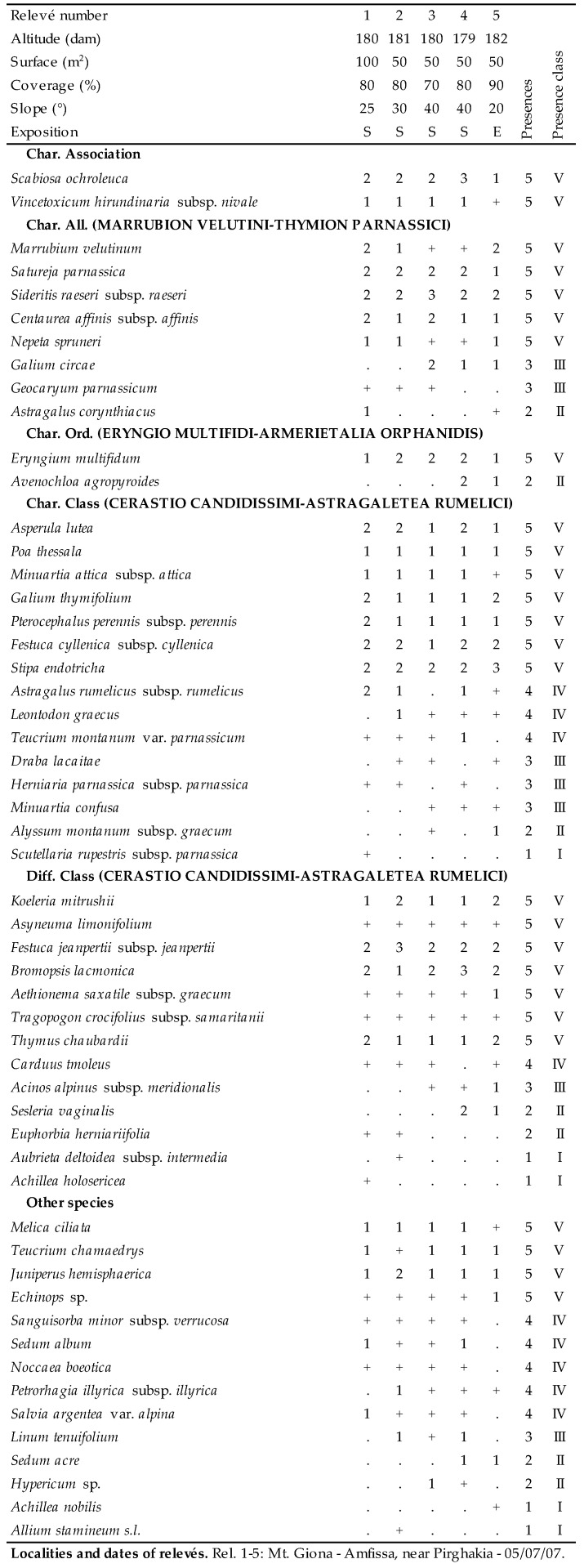
*Scabioso ochroleucae-Sideridetum raeseri* Musarella, Brullo & Giusso ass. nov.

**Table A20:**
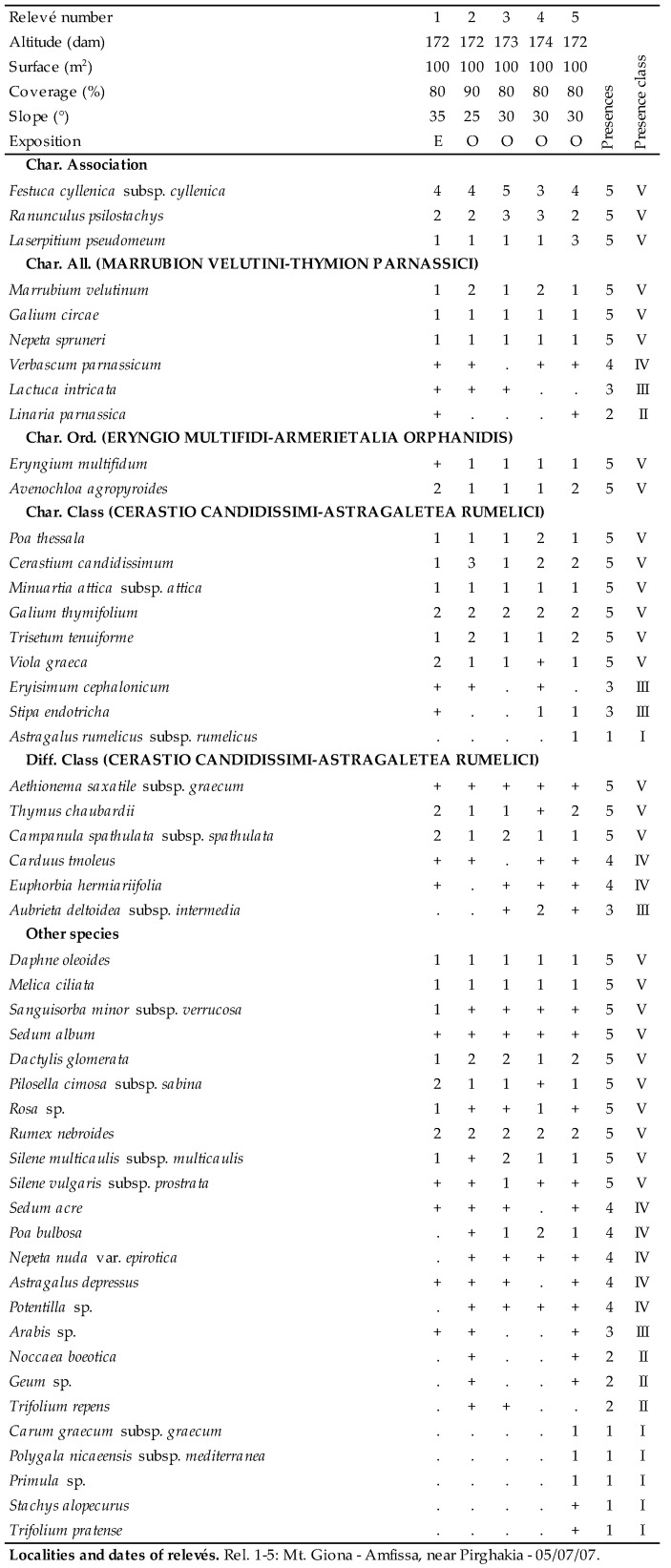
Ranunculo psilostachydis-Festucetum cyllenicae Musarella, Brullo & Giusso ass. nov.

**Table A21:**
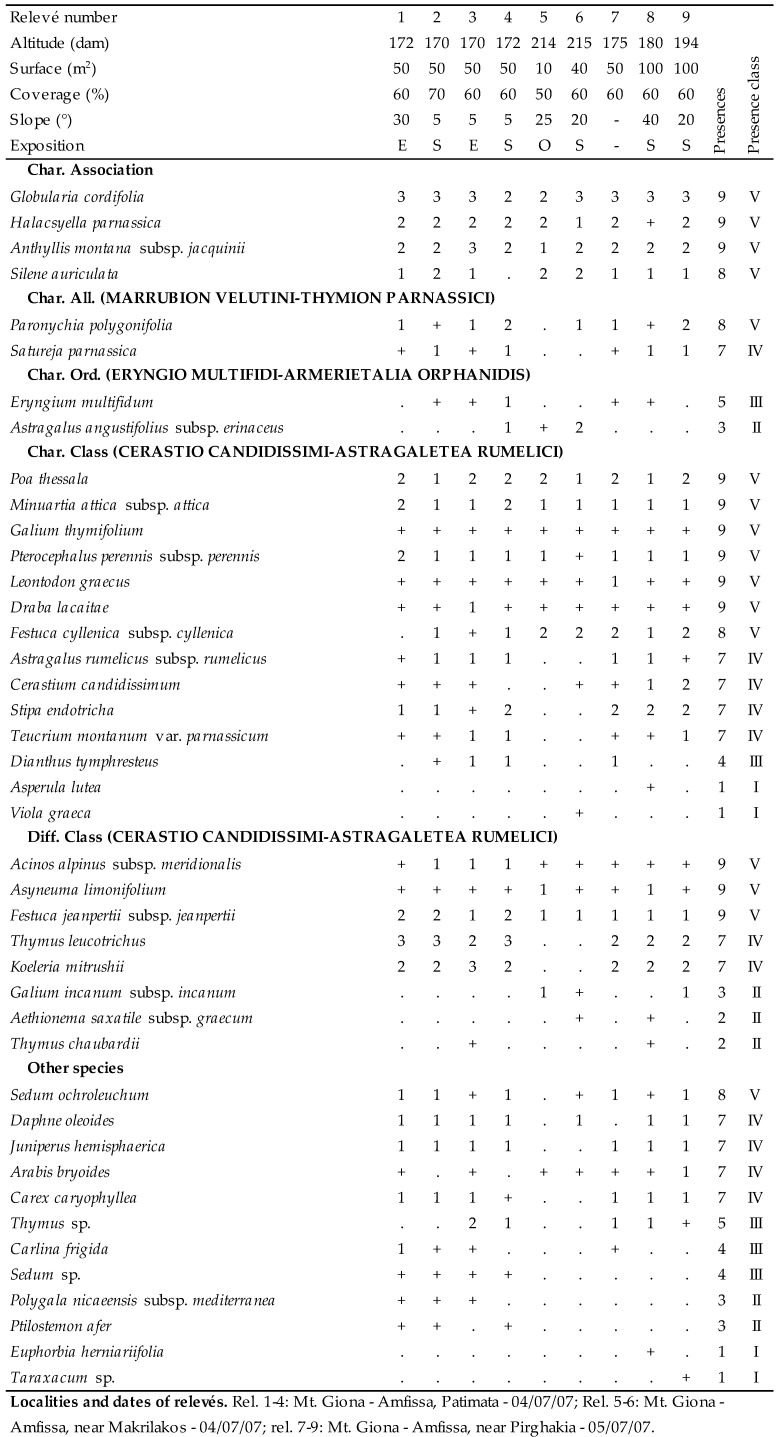
*Edraiantho parnassici-Globularietum cordifoliae* Musarella, Brullo & Giusso ass. nov.

**Table A22:**
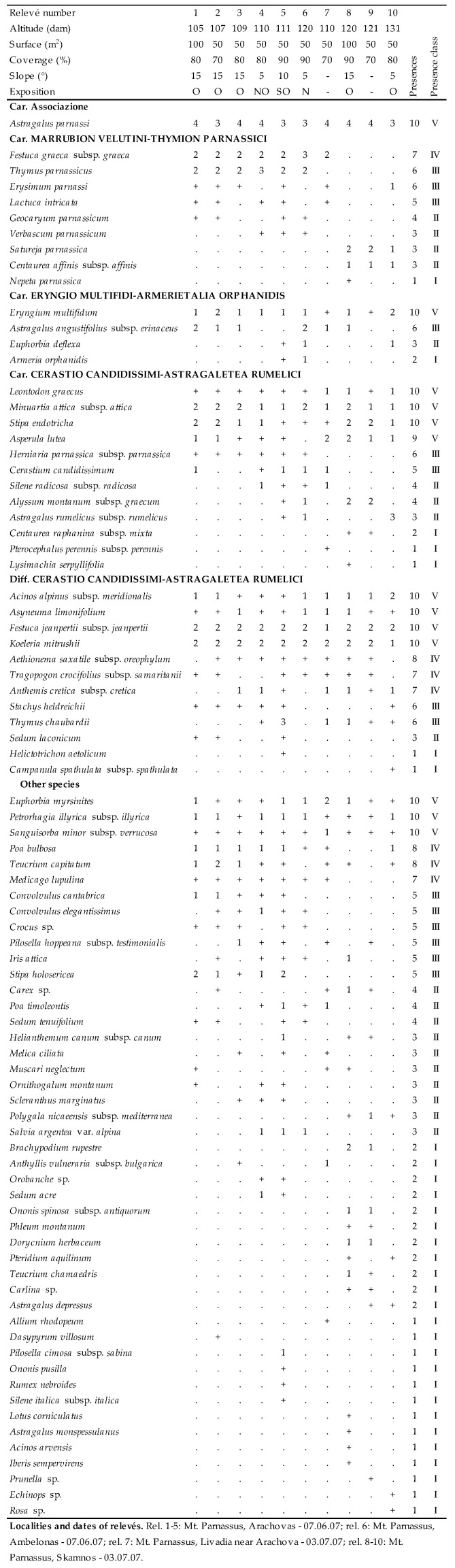
*Thymo parnassici-Astragaletum parnassi* Musarella, Brullo & Giusso ass. nov.

**Table A23:**
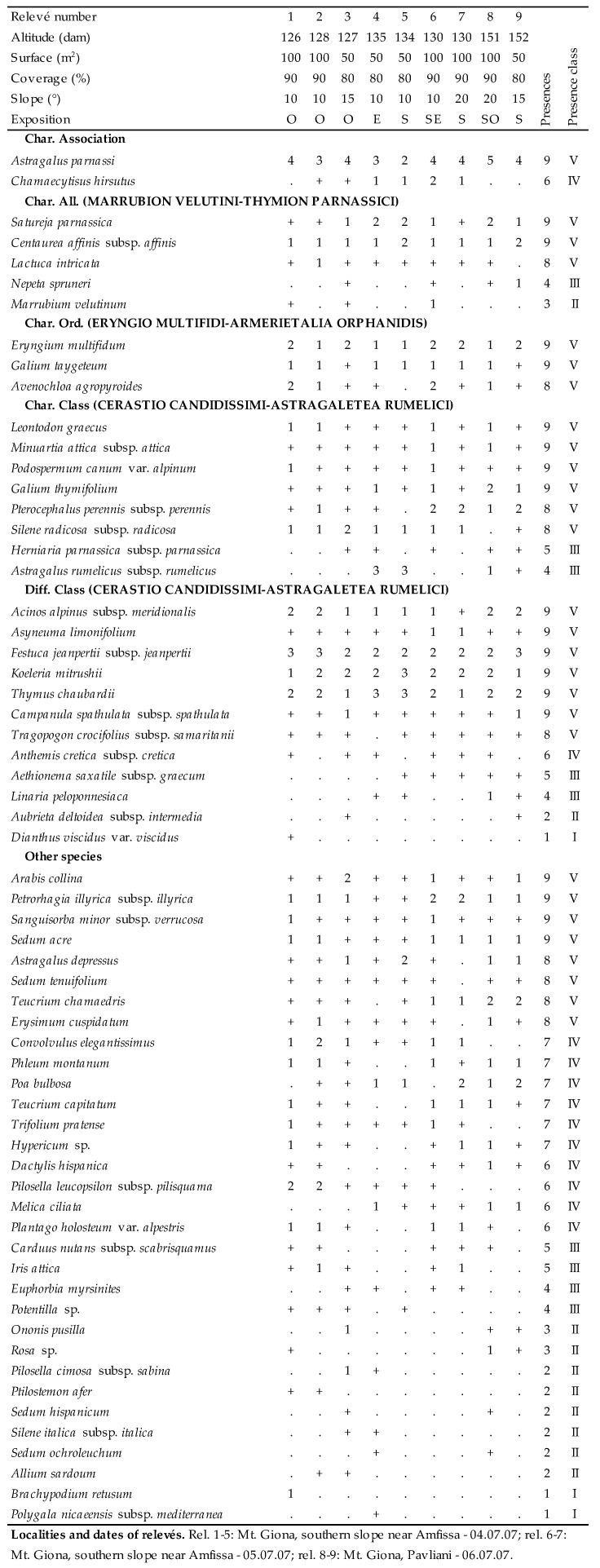
*Chamaecytiso hirsuti-Astragaletum parnassi* Musarella, Brullo & Giusso ass. nov.

**Table A24:**
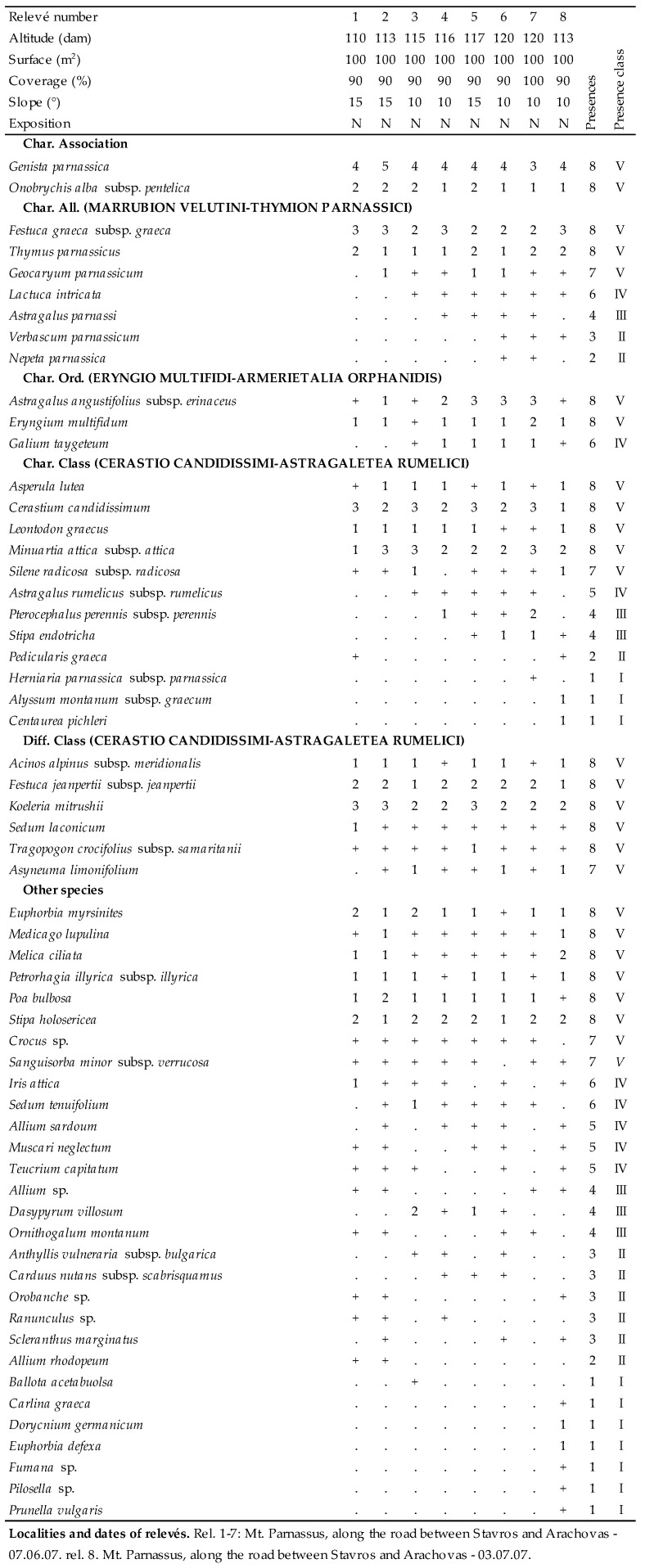
*Onobrychido laconicae-Genistetum parnassicae* Musarella, Brullo & Giusso ass. nov.

**Table A25:**
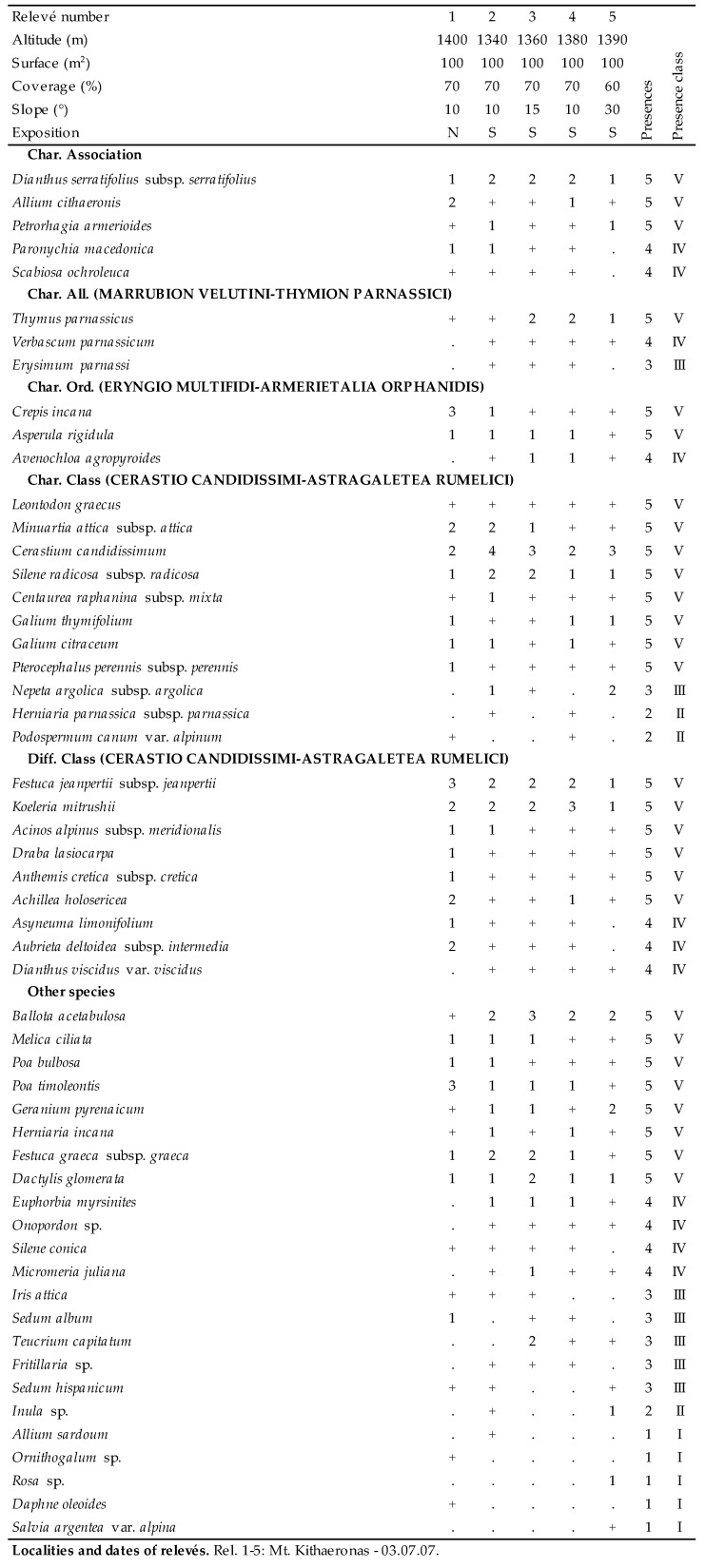
*Allio cithaeronis-Dianthetum serratifolii* Musarella, Brullo & Giusso ass. nov.

**Table A26:**
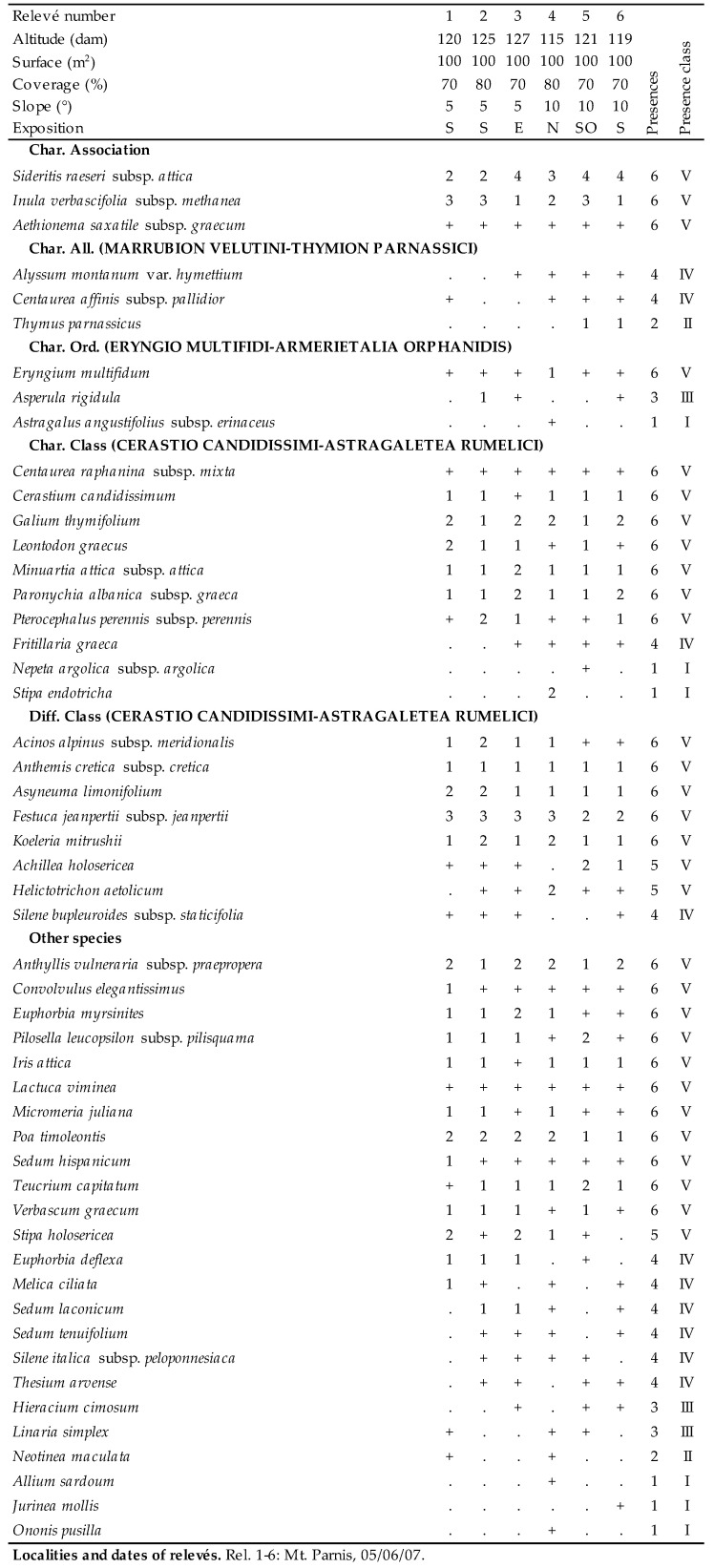
*Inulo methaneae-Sideritetum atticae* Musarella, Brullo & Giusso ass. nov.

**Table A27:**
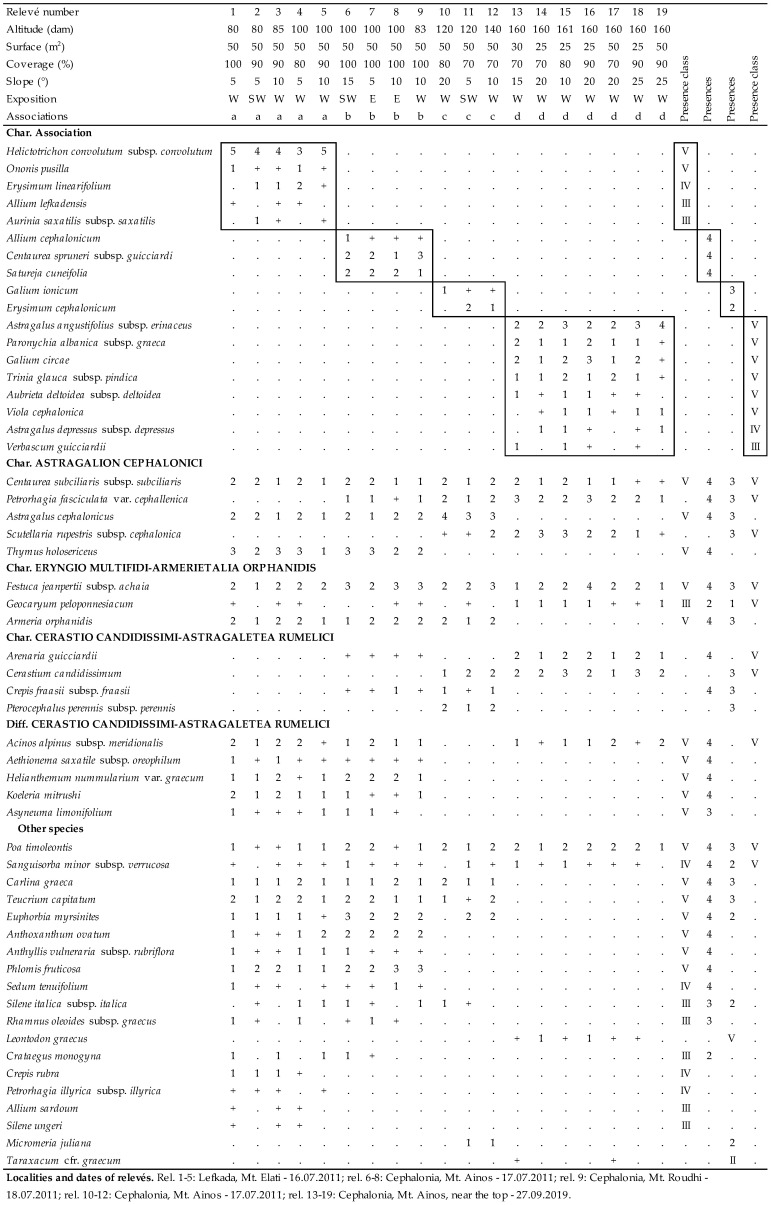
Helictotricho convoluti-Thymetum holosericei Musarella, Brullo & Giusso ass. nov. (a) rel. 1–5; Saturejo cuneifoliae-Thymetum holosericei Musarella, Brullo & Giusso ass. nov. (b) rel. 6–9; Scutellario cephalonicae-Astragaletum cephalonici Musarella, Brullo & Giusso ass. nov. (c) rel. 10–12; Paronychio graecae-Astragaletum erinacei Musarella, Brullo & Giusso ass. nov. (d) rel. 13–19.

**Table A28:**
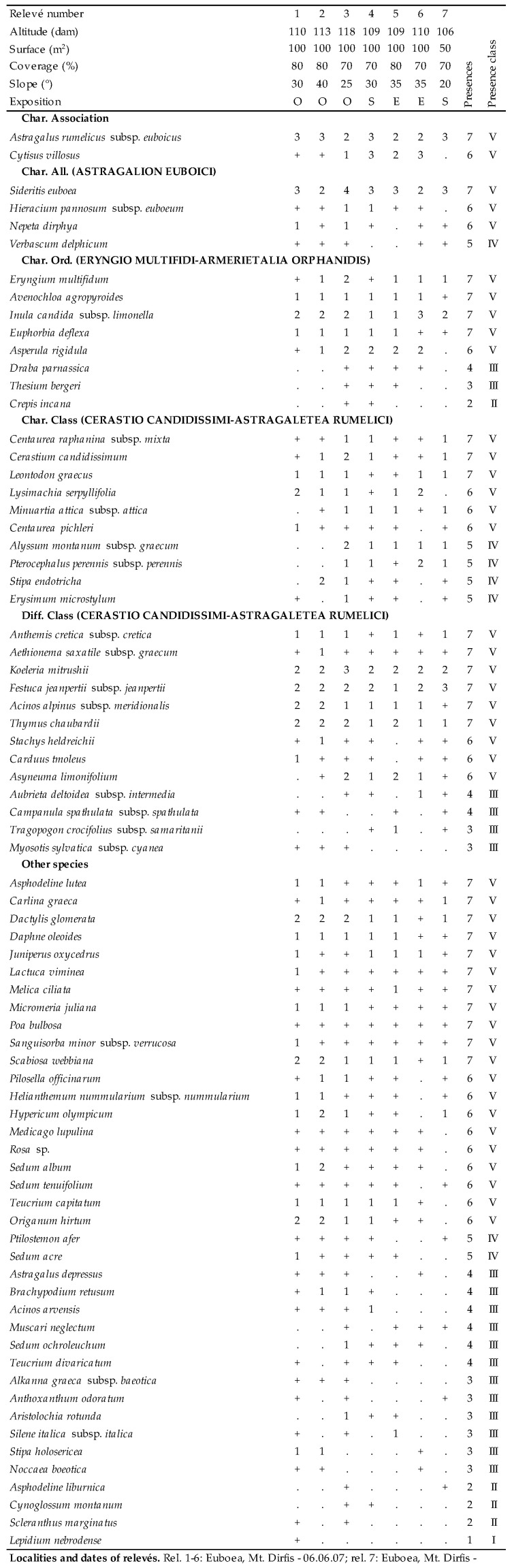
*Sideritido euboeae-Astragaletum euboici* Musarella, Brullo & Giusso ass. nov.

**Table A29:**
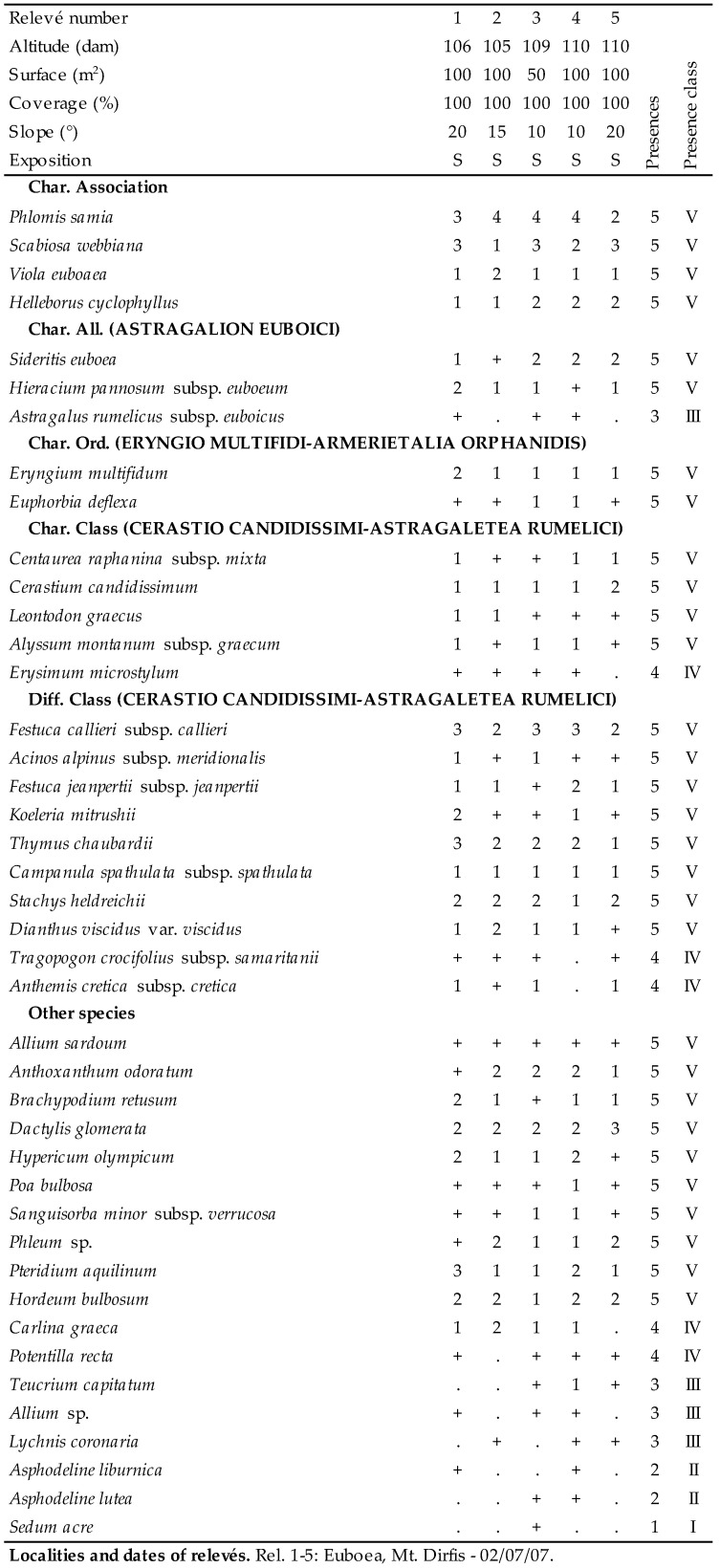
*Scabioso webbianae-Phlomidetum samiae* Musarella, Brullo & Giusso ass. nov.

**Table A30:**
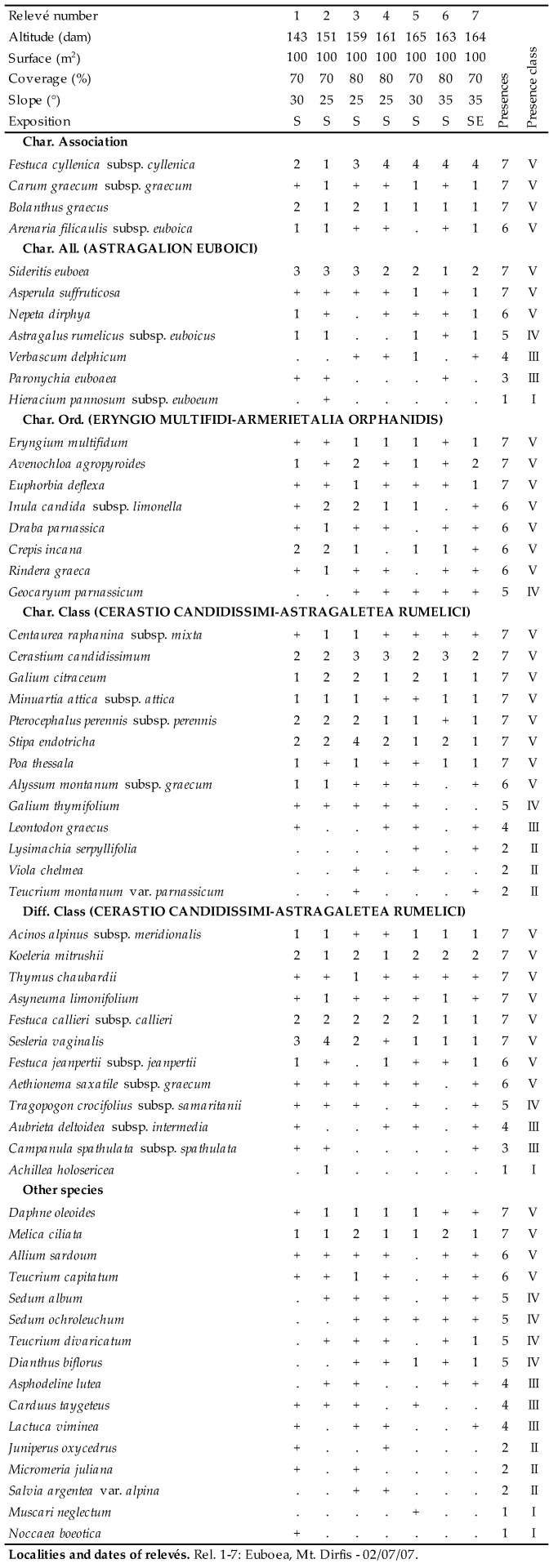
*Sideritido euboeae-Festucetum cyllenicae* Musarella, Brullo & Giusso ass. nov.

**Table A31:**
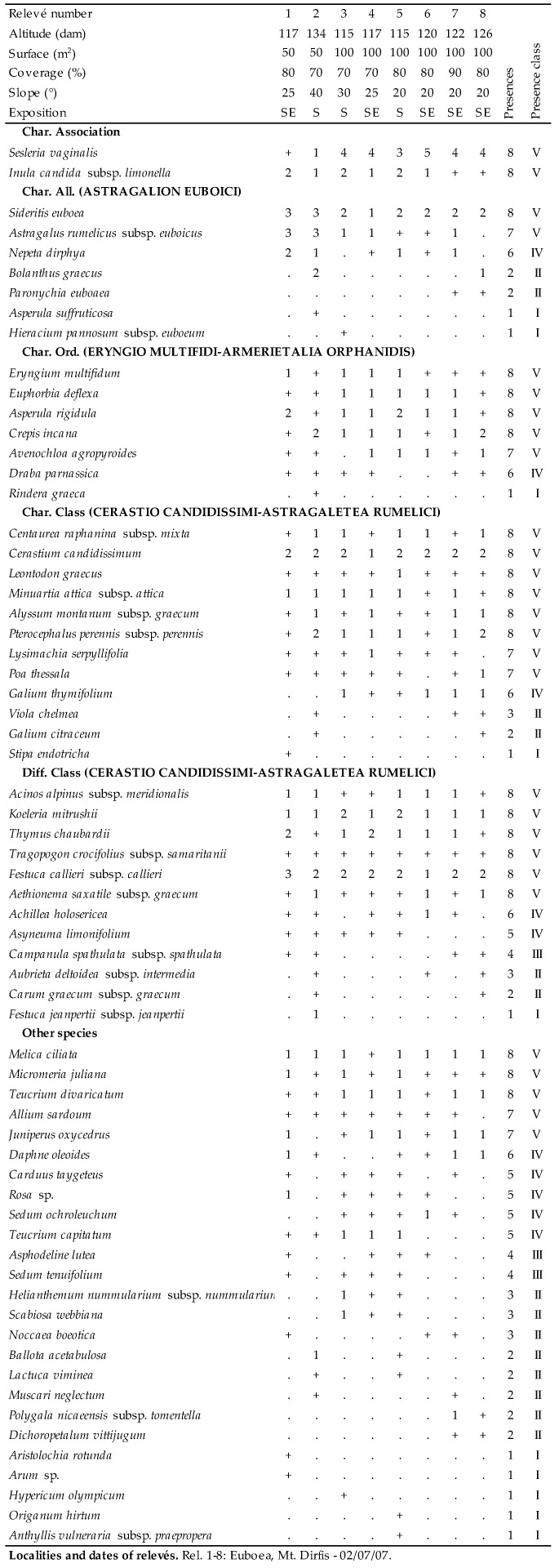
*Inulo limonellae-Seslerietum vaginalis* Musarella, Brullo & Giusso ass. nov.

**Table A32:**
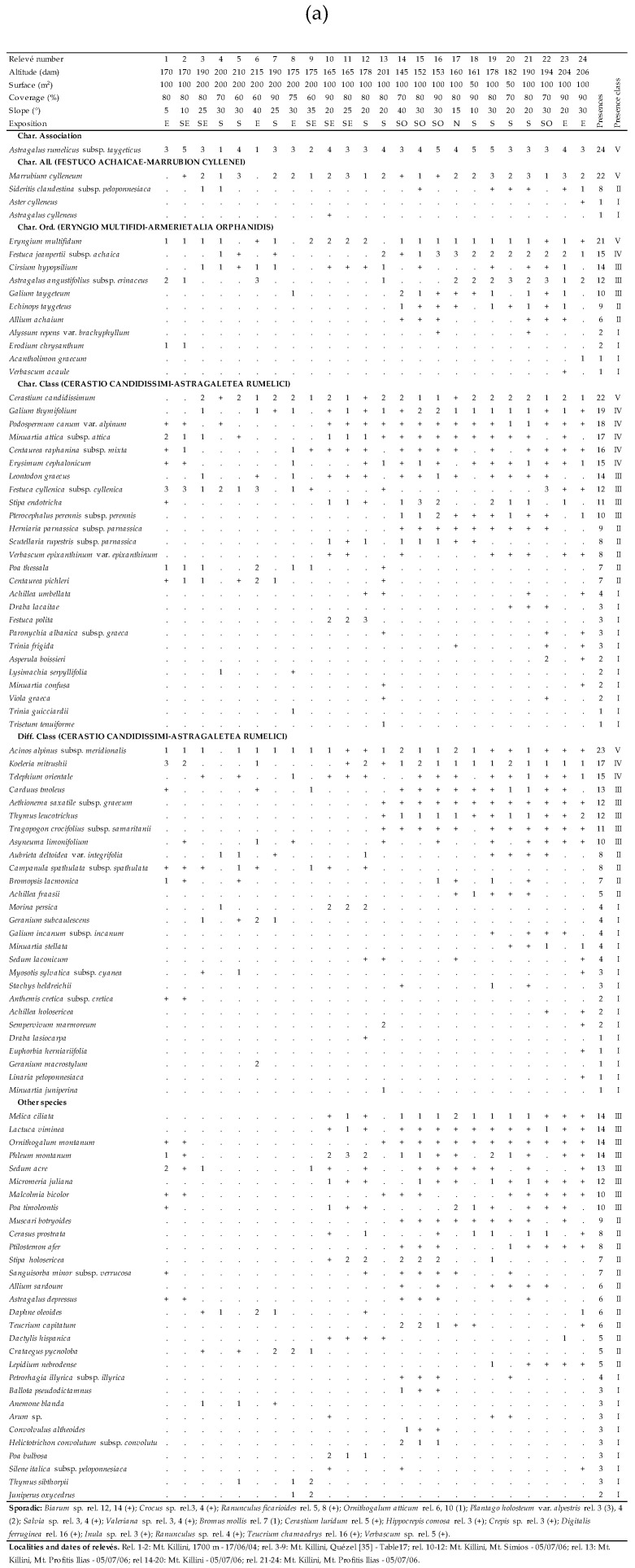

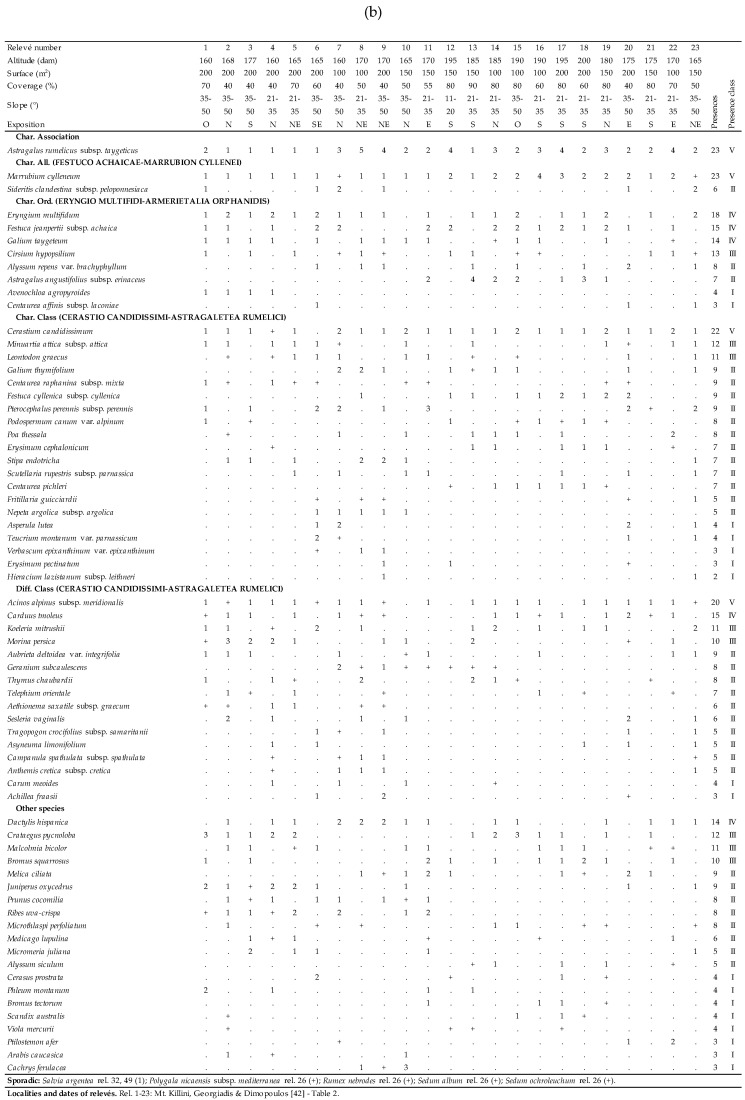

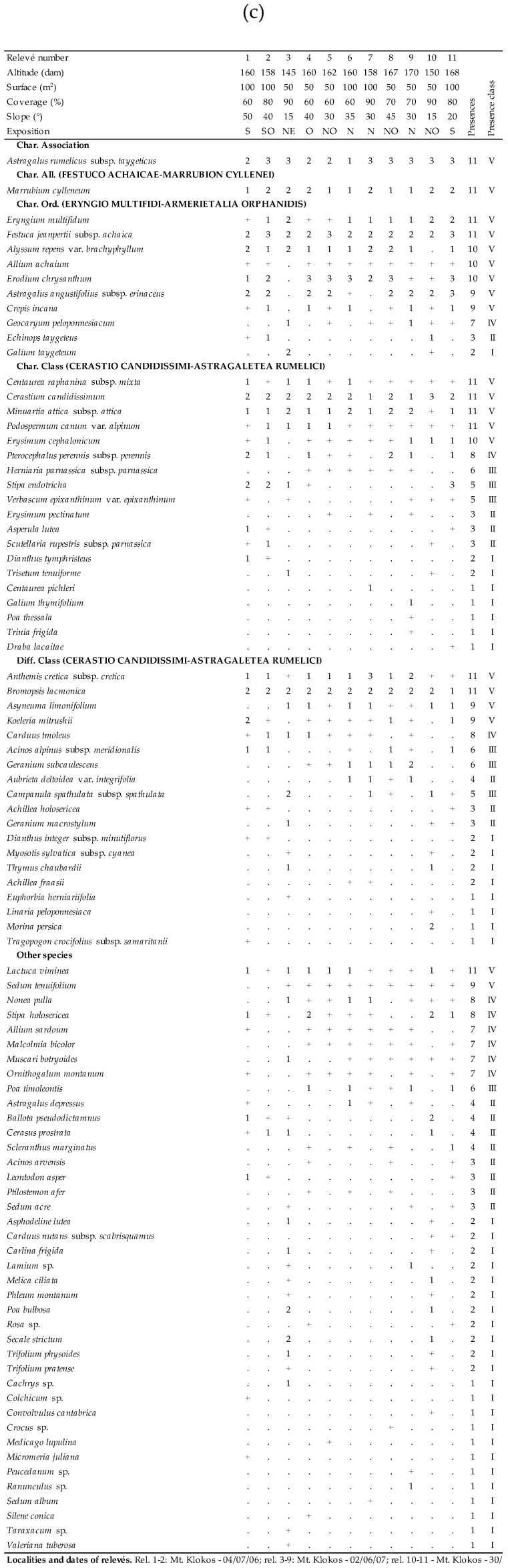

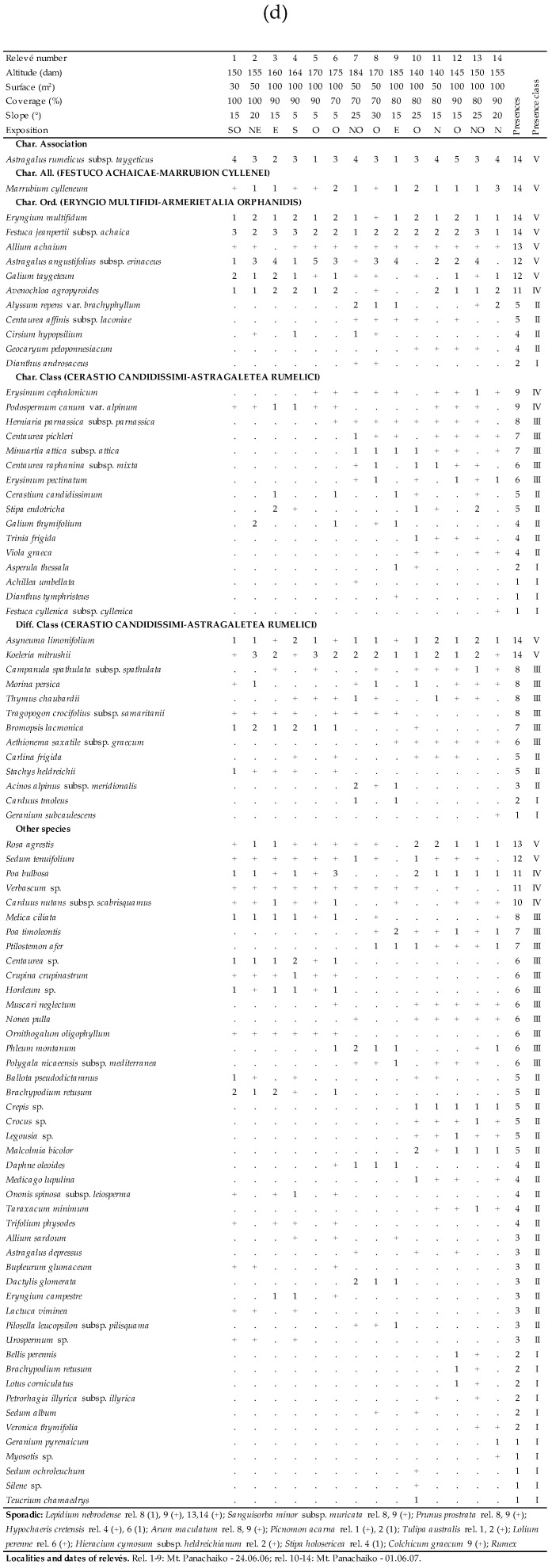

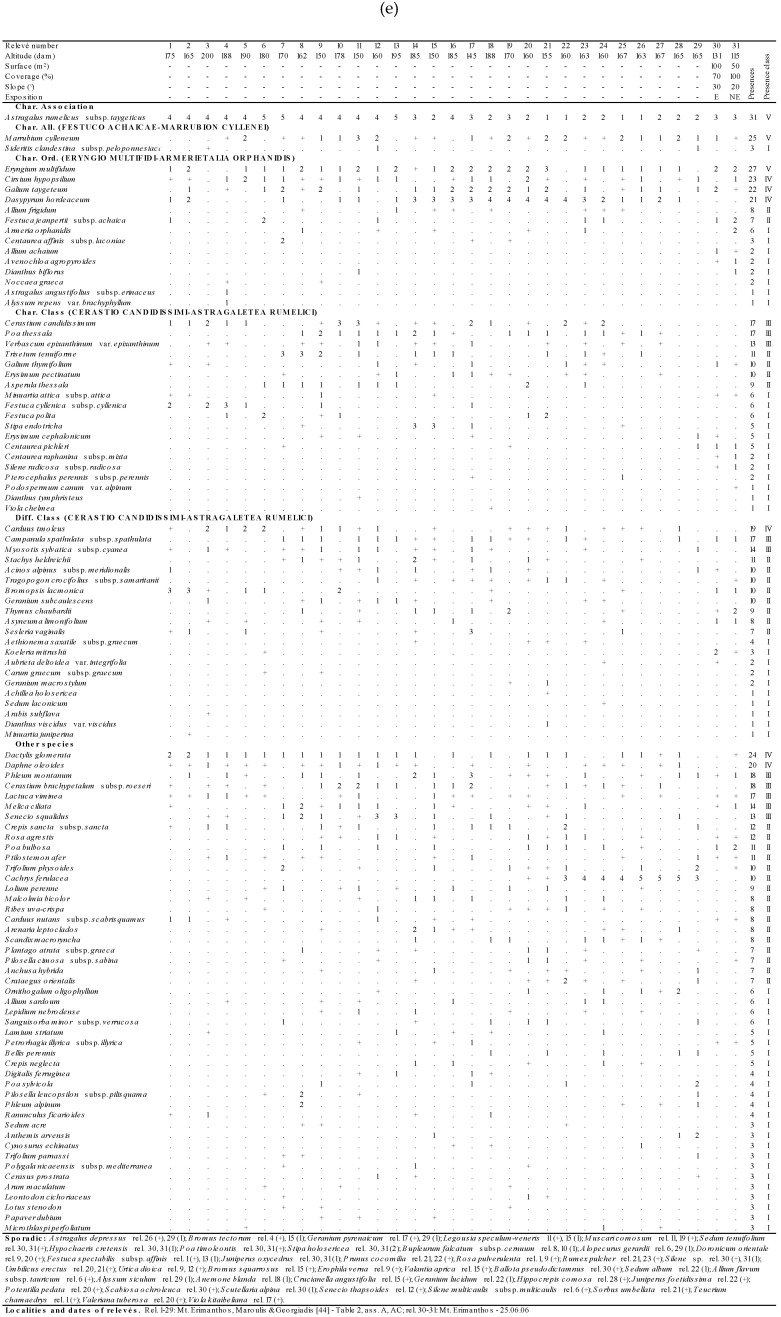
Cirsio hypopsilii-Astragaletum taygetici Quézel 1964 corr.

**Table A33:**
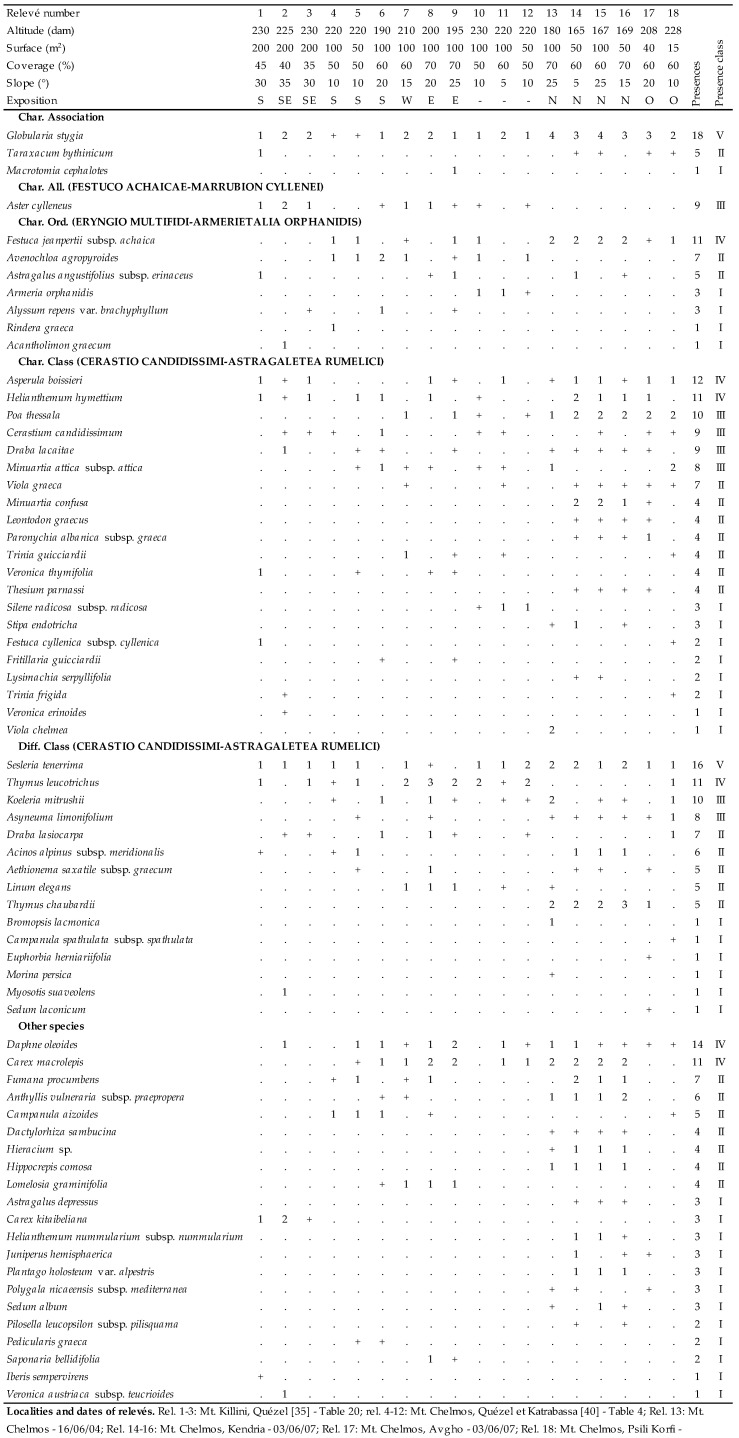
Asteri cyllenei-Globularietum stygiae Quézel 1964.

**Table A34:**
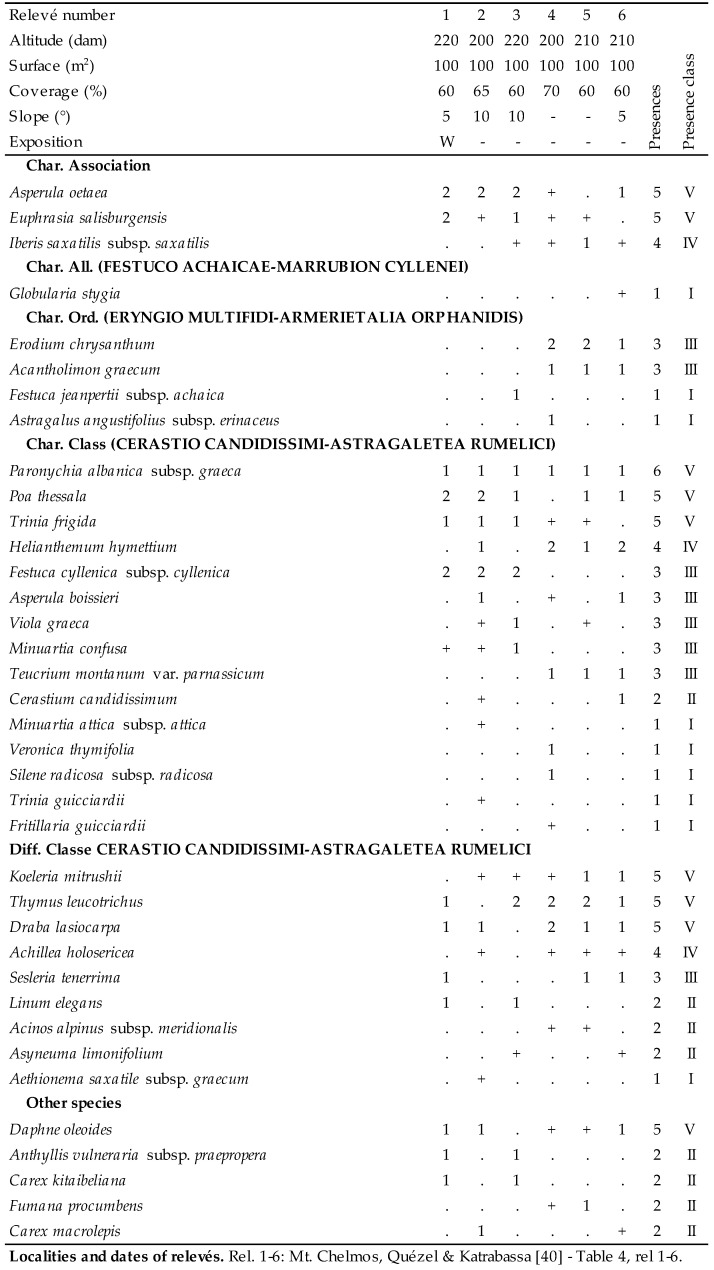
Euphrasio salisburgensis-Asperuletum oetaeae Quézel & Katrabassa 1974 corr.

**Table A35:**
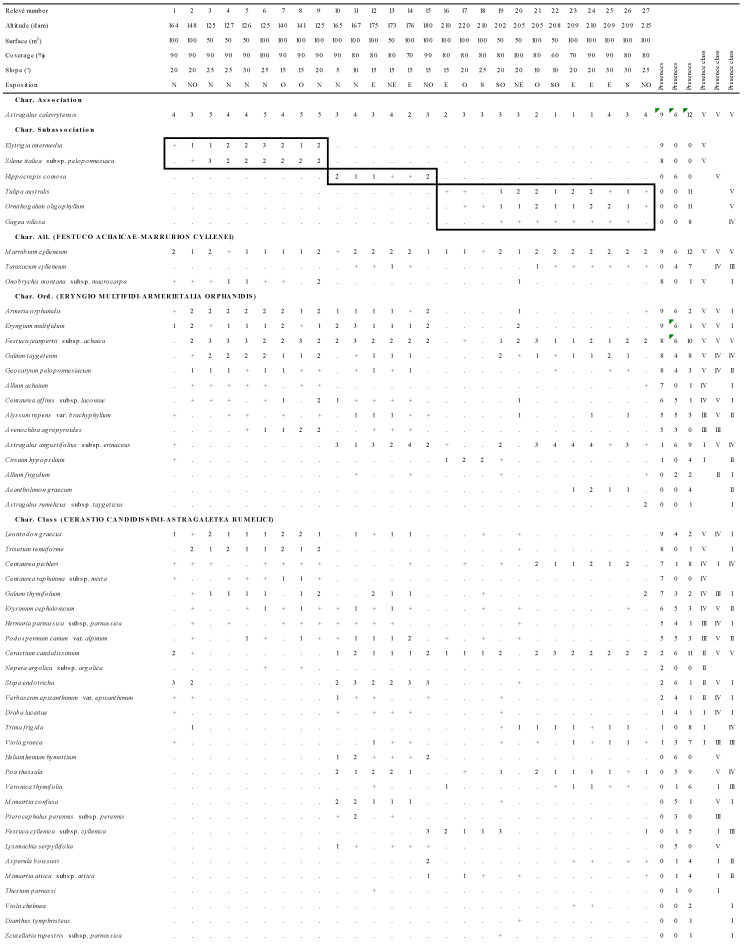

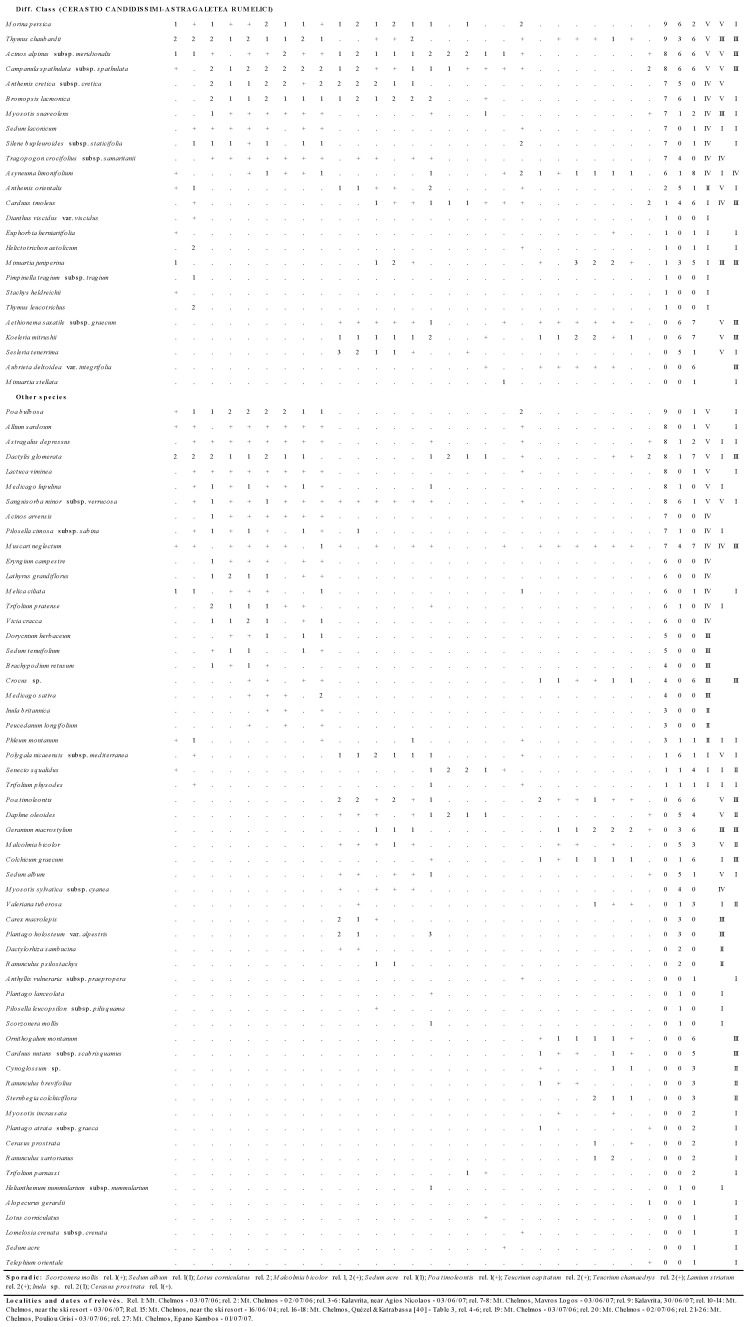
*Marrubio cyllenei-Astragaletum calavrytensis* Musarella, Brullo & Giusso ass. nov.

**Table A36:**
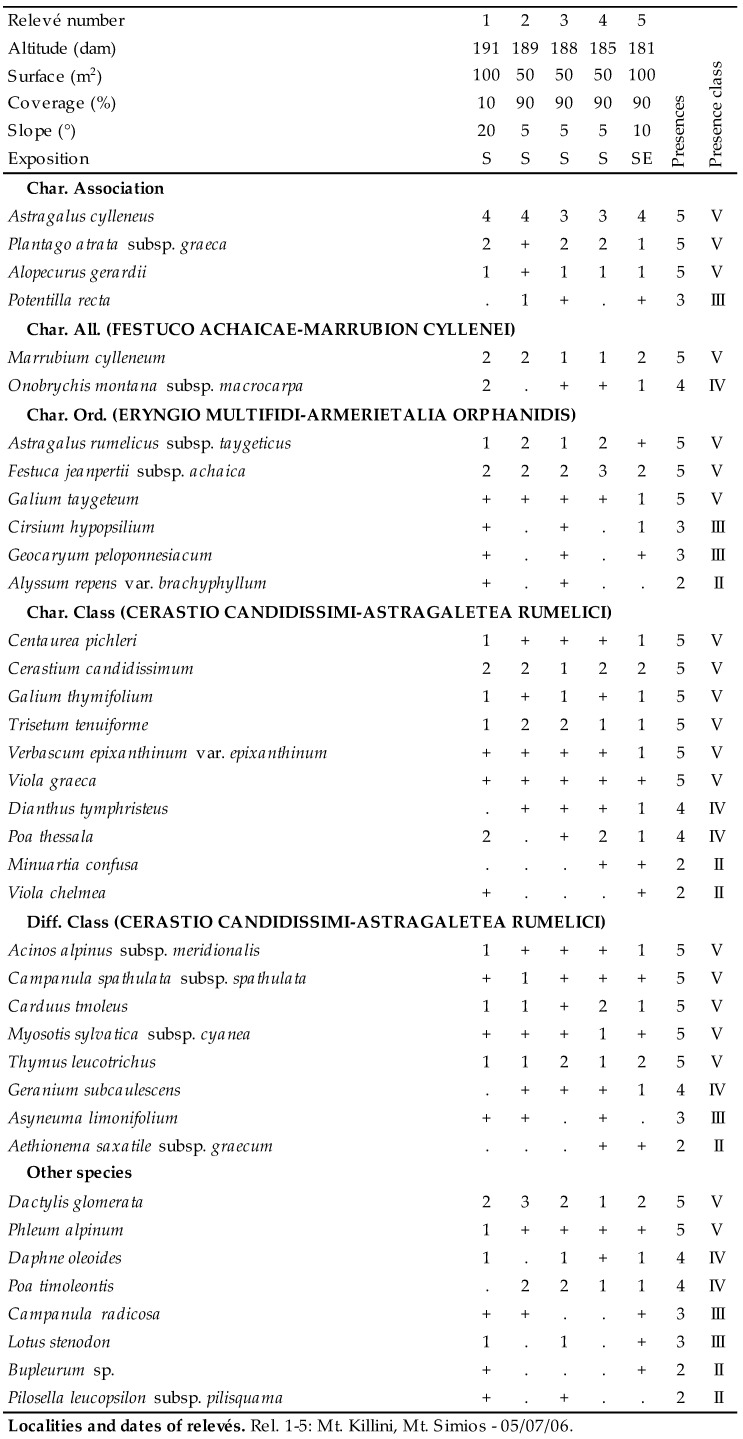
*Plantagini graecae-Astragaletum cyllenei* Musarella, Brullo & Giusso ass. nov.

**Table A37:**
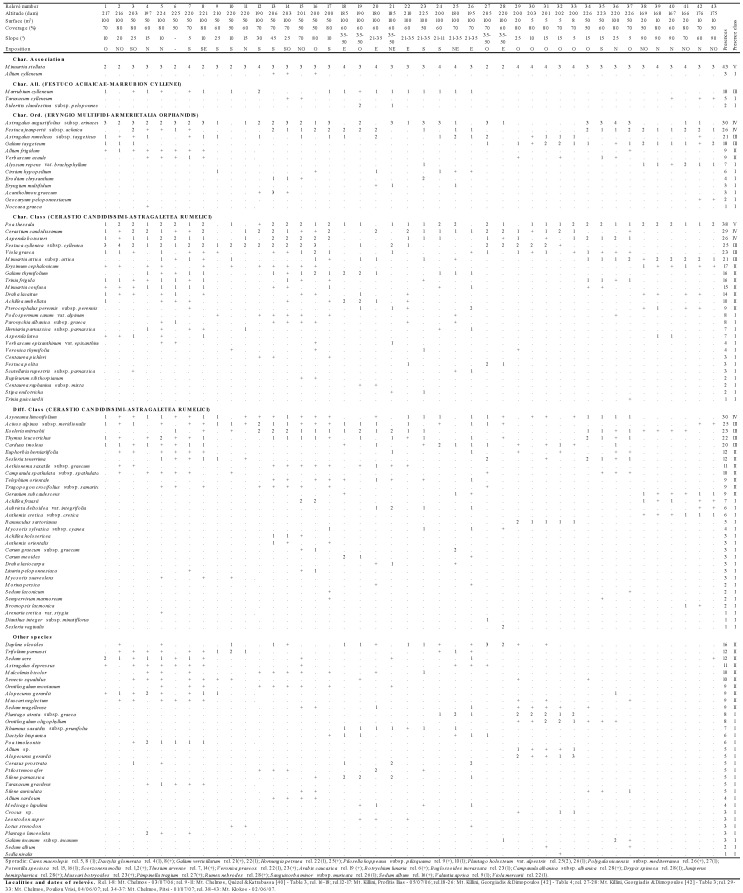
*Festuco achaicae-Minuartietum stellatae* Musarella, Brullo & Giusso ass. nov.

**Table A38:**
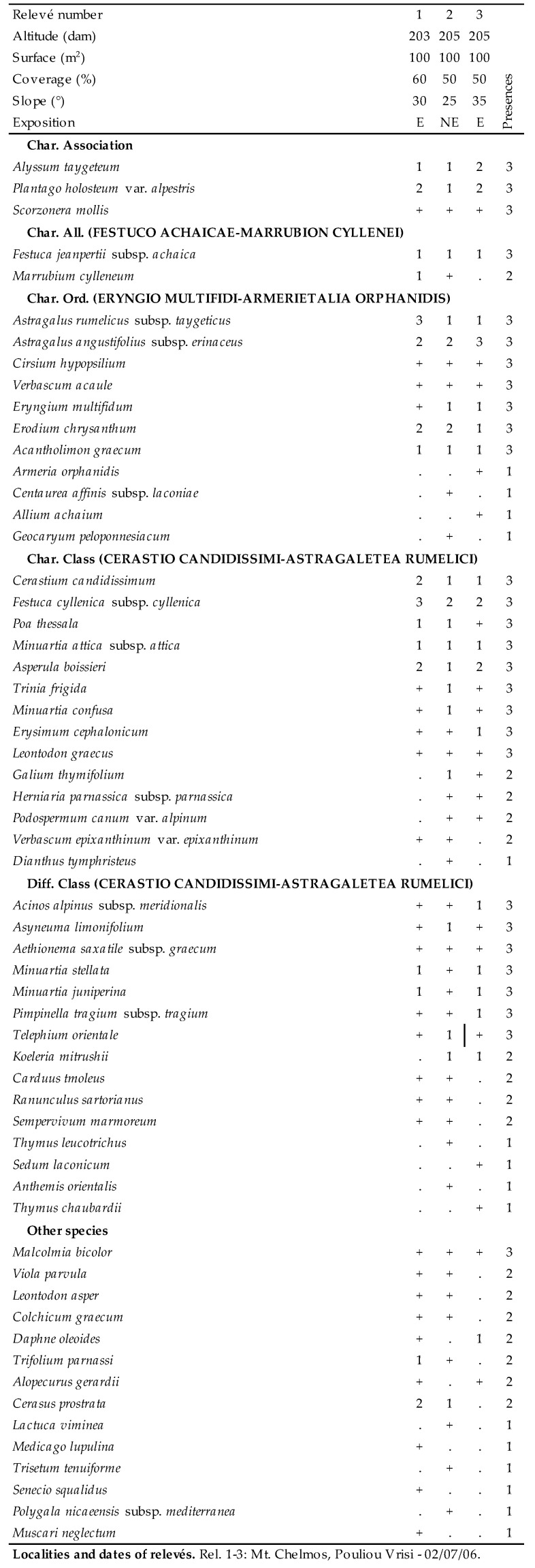
*Alysso taygetei-Plantaginetum alpestris* Musarella, Brullo & Giusso ass. nov.

**Table A39:**
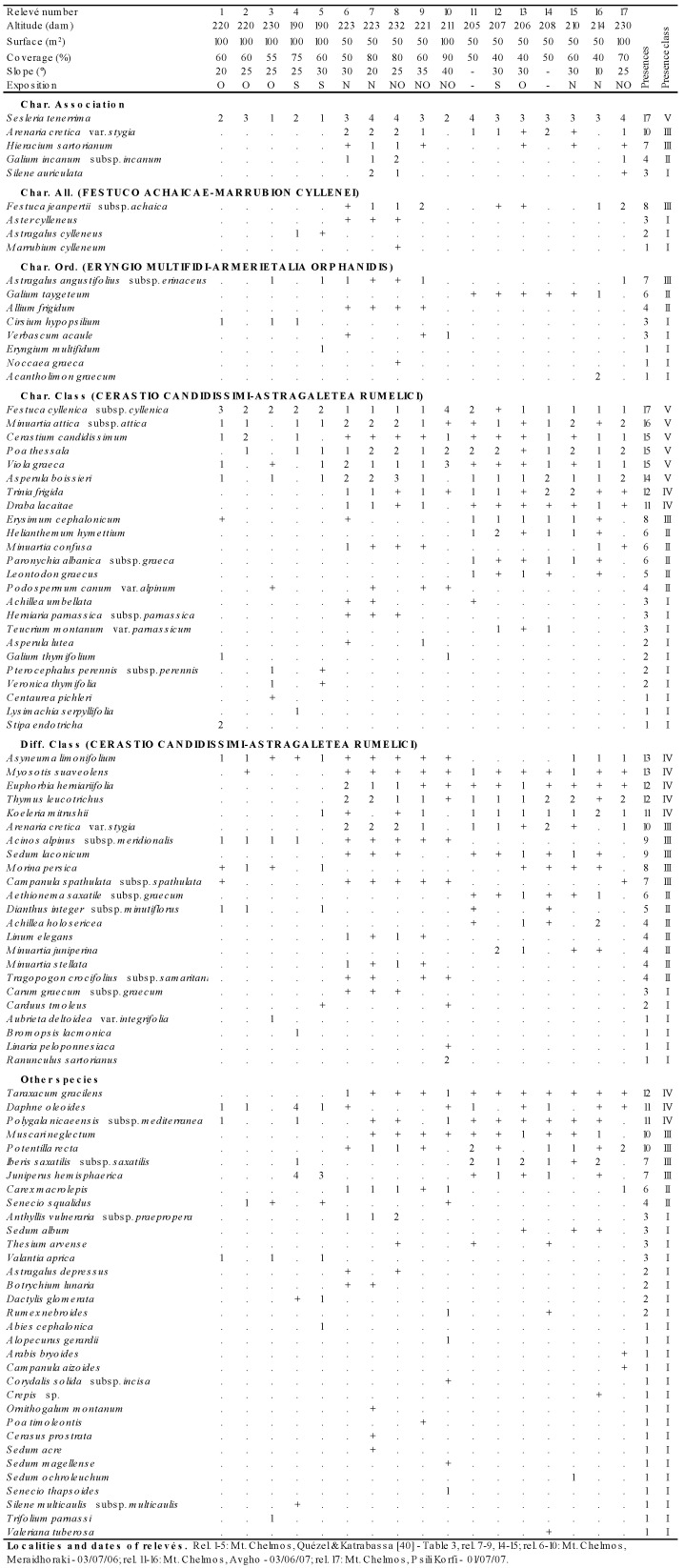
*Hieracio sartoriani-Seslerietum tenerrimae* Musarella, Brullo & Giusso ass. nov.

**Table A40:**
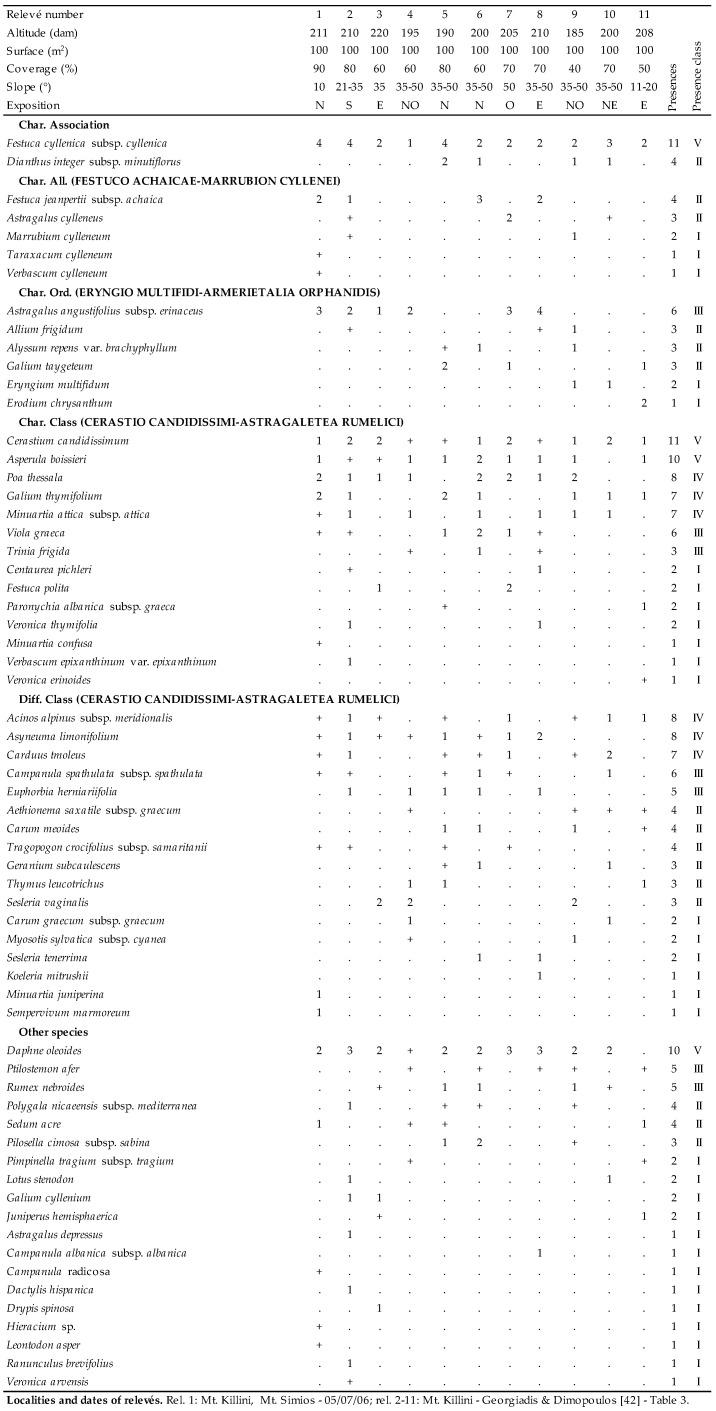
*Asperulo boissieri-Festucetum cyllenicae* Georgiadis & Dimopoulos ex Musarella, Brullo & Giusso ass. nov.

**Table A41:**
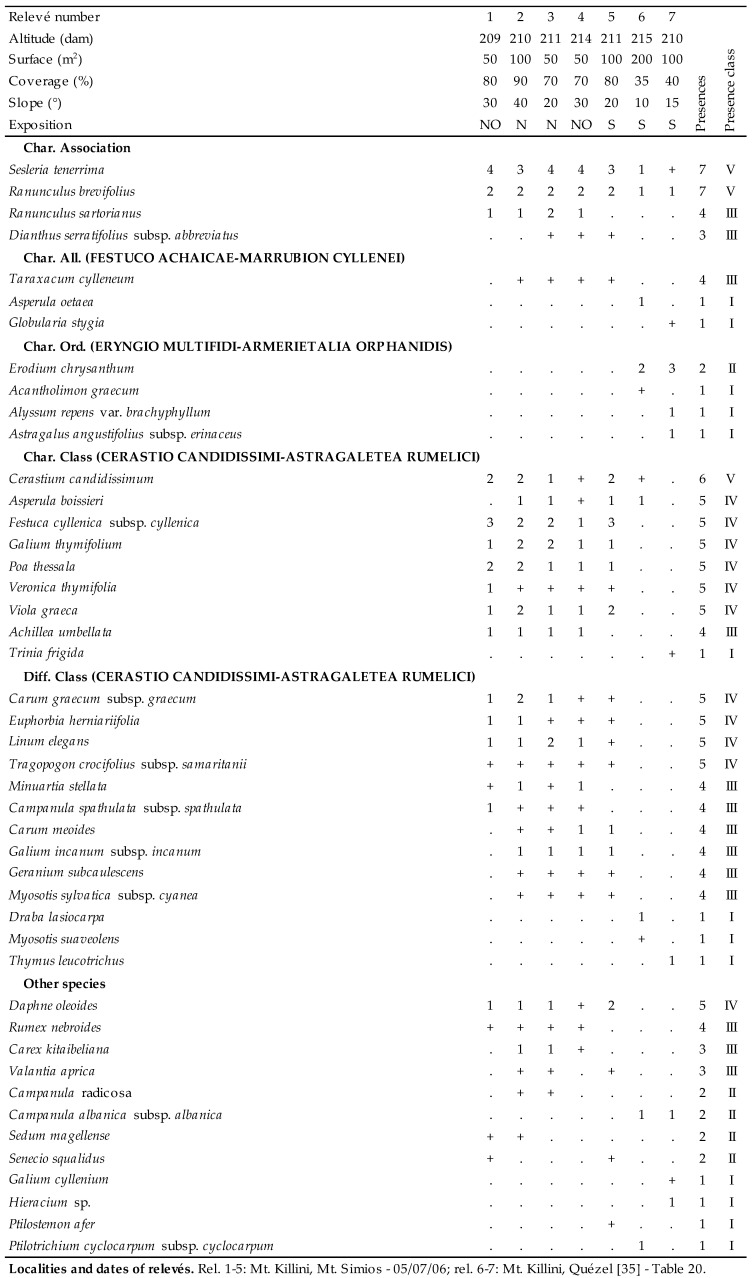
*Ranunculo brevifolii-Seslerietum tenerrimae* Musarella, Brullo & Giusso ass. nov.

**Table A42:**
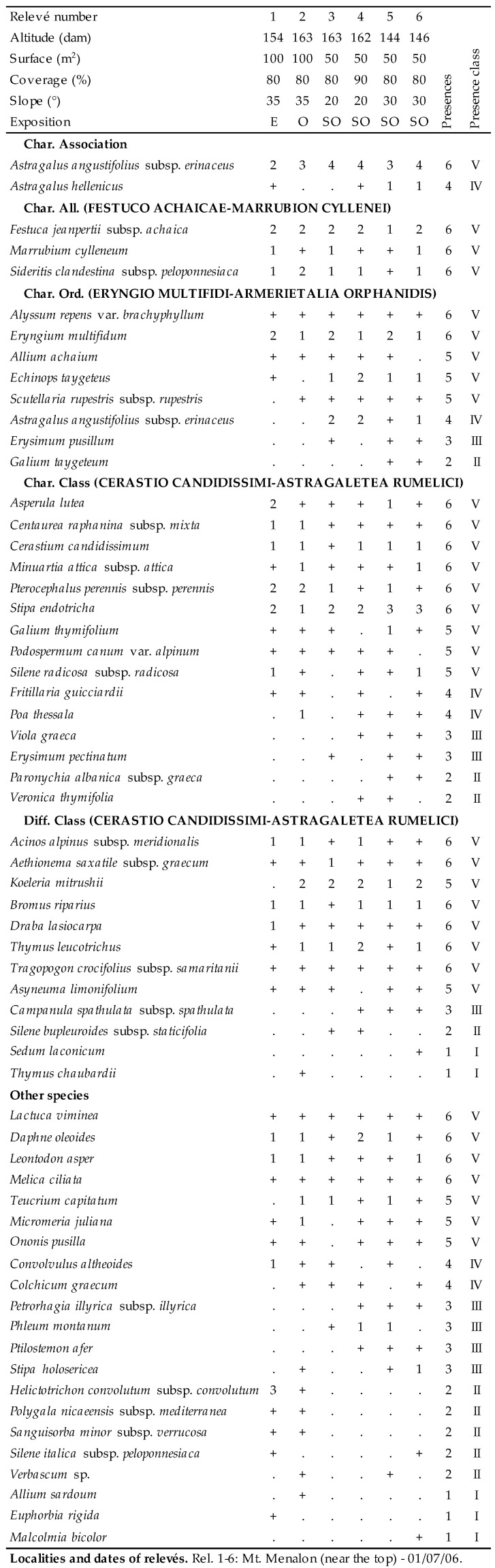
*Astragaletum hellenico-erinacei* Musarella, Brullo & Giusso ass. nov.

**Table A43:**
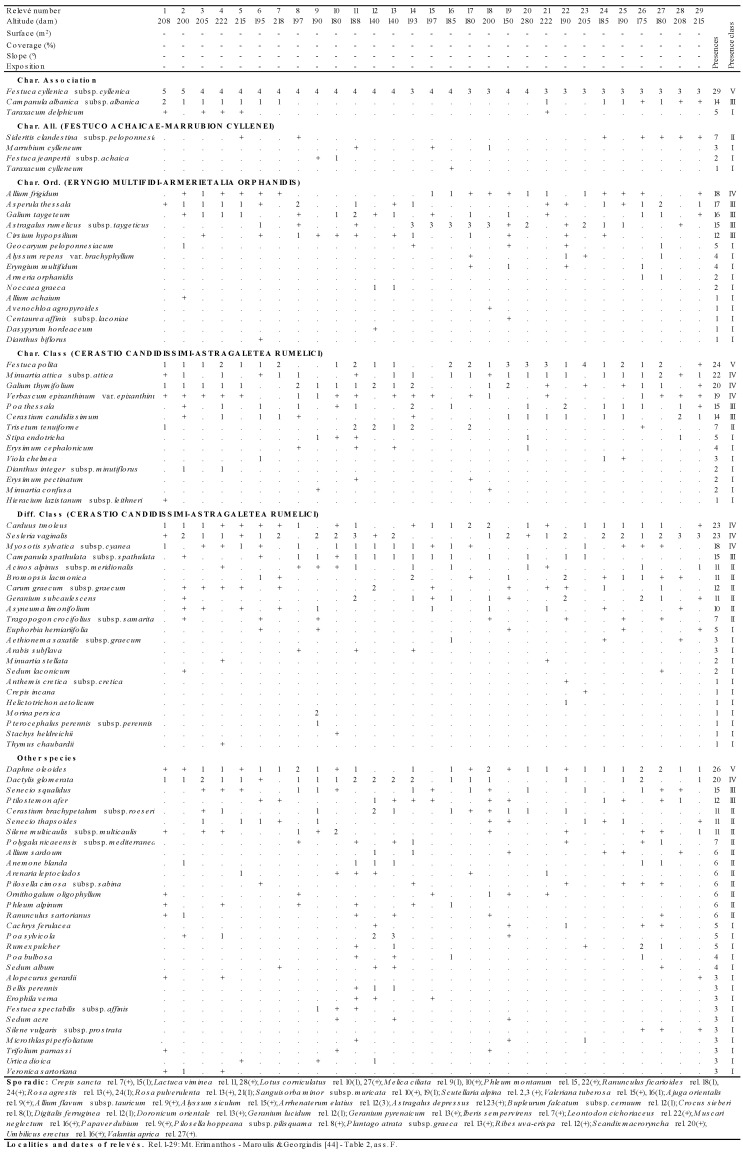
*Festucetum polito-cyllenicae* Maroulis & Georgiadis 2005.

**Table A44:**
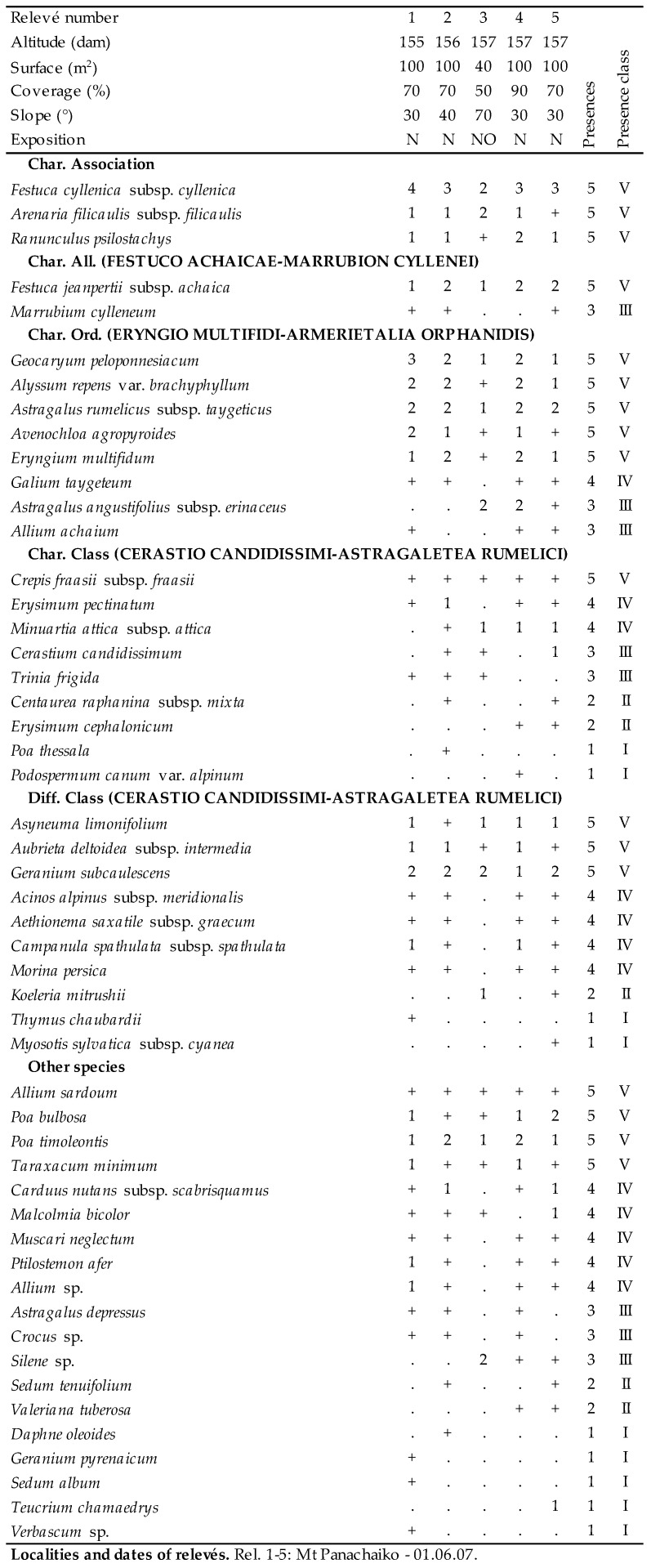
*Arenario filicaulis-Festucetum cyllenicae* Musarella, Brullo & Giusso ass. nov.

**Table A45:**
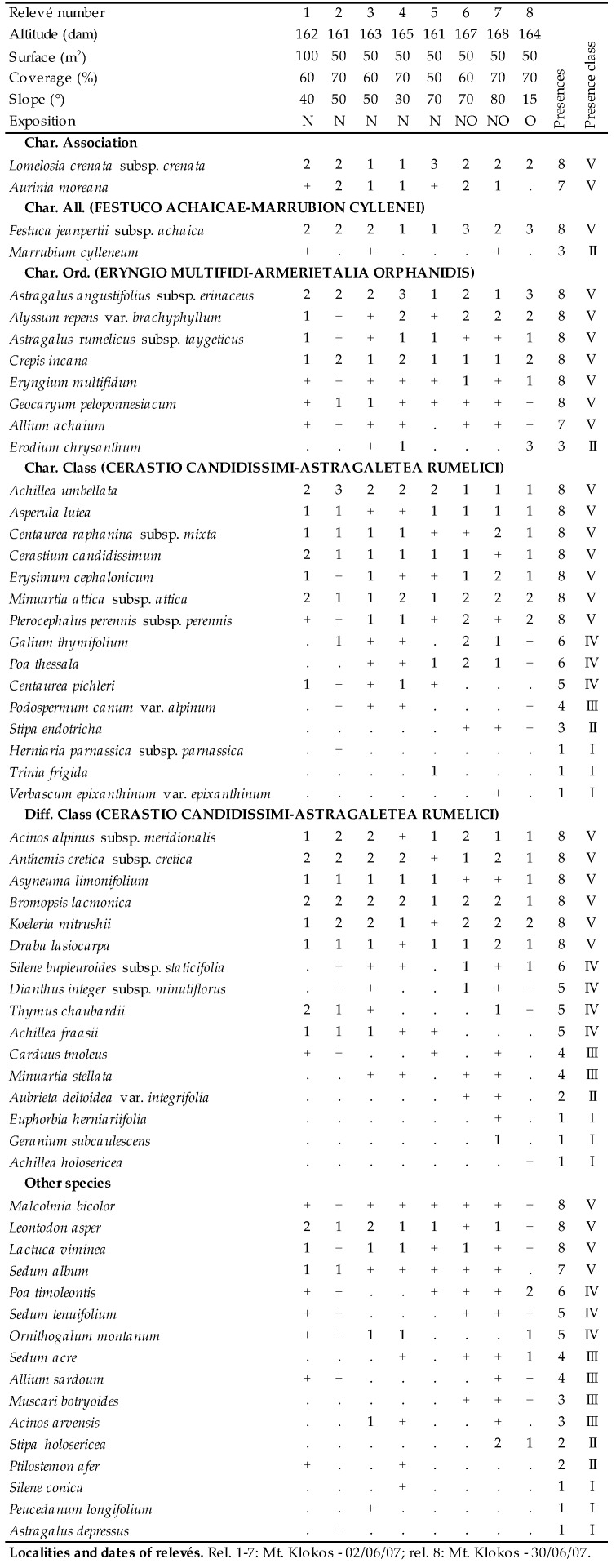
*Aurinio moreanae-Lomelosietum crenatae* Musarella, Brullo & Giusso ass. nov.

**Table A46:**
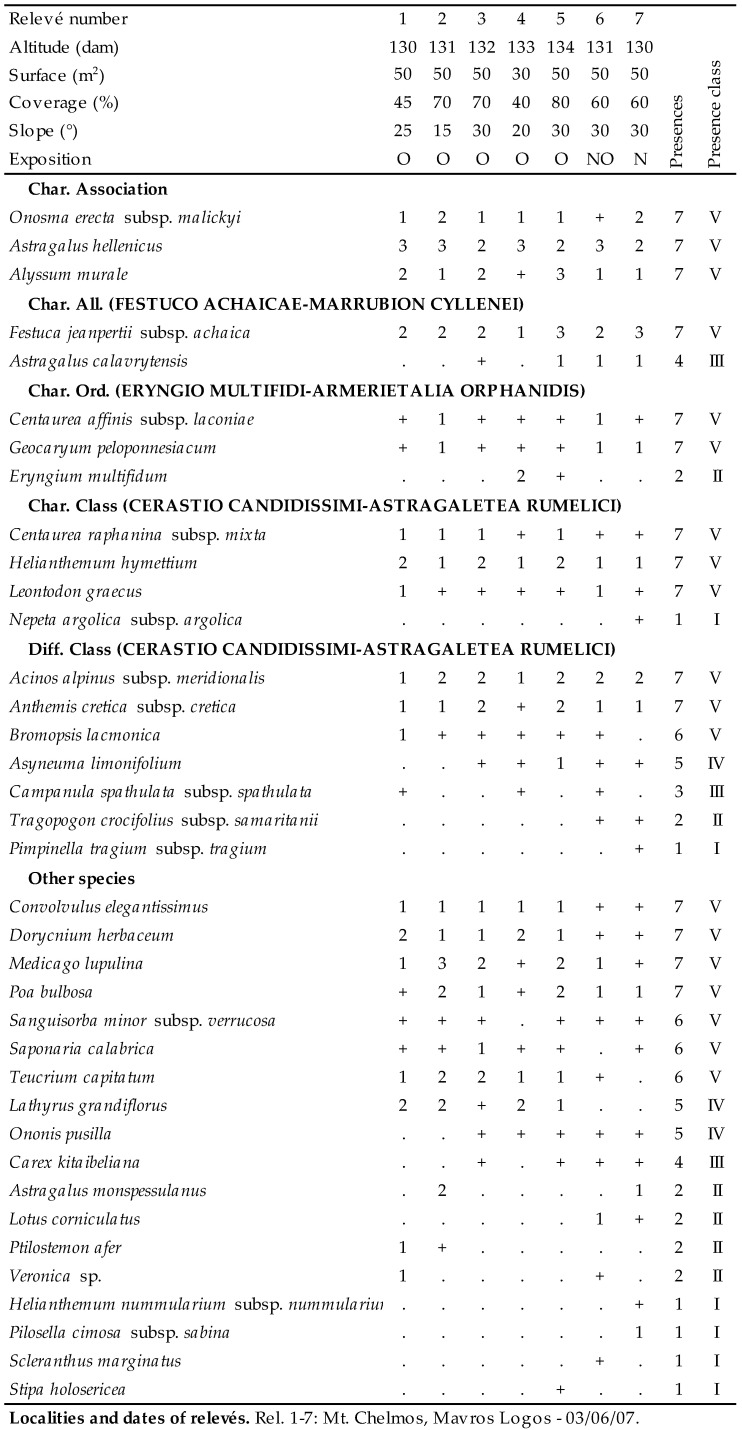
*Onosmo malickyi-Astragaletum hellenici* Musarella, Brullo & Giusso ass. nov.

**Table A47:**
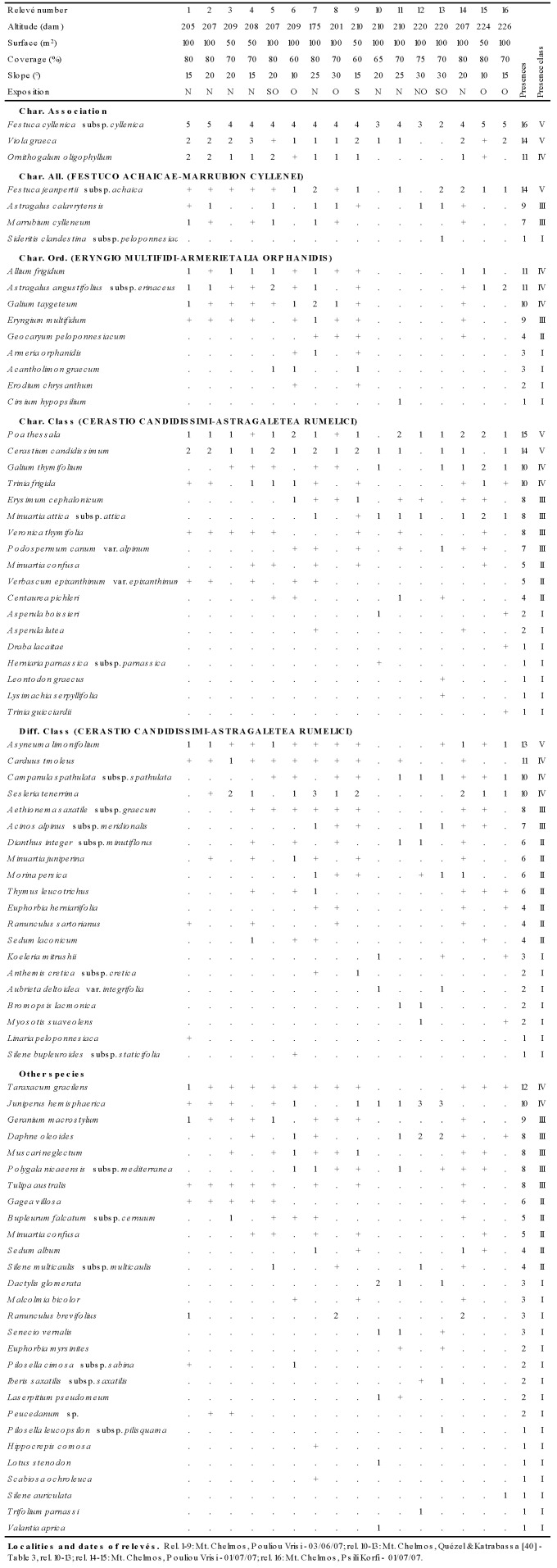
*Violo grecae-Festucetum cyllenicae* Musarella, Brullo & Giusso ass. nov.

**Table A48:**
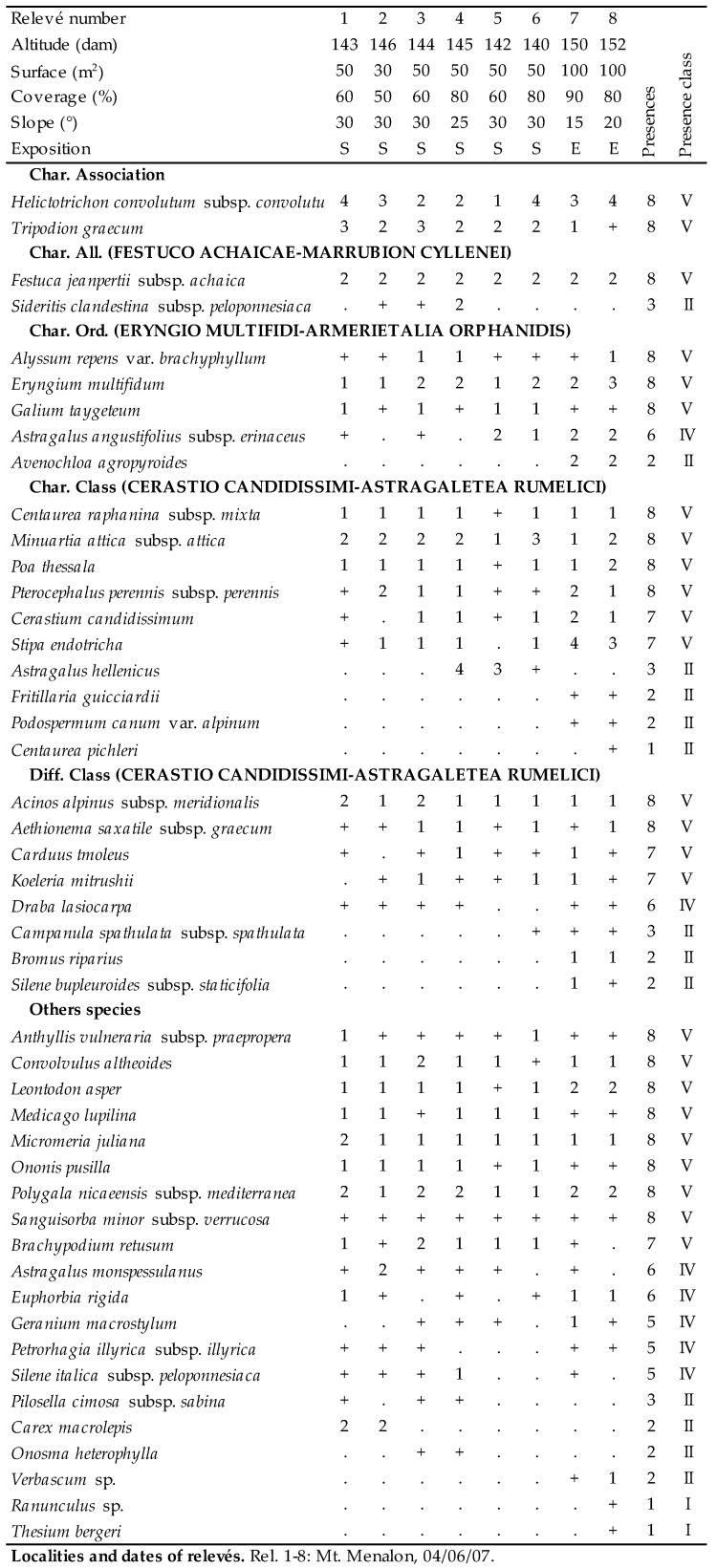
*Tripodio graeci-Helictotrichetum heldreichii* Musarella, Brullo & Giusso ass. nov.

**Table A49:**
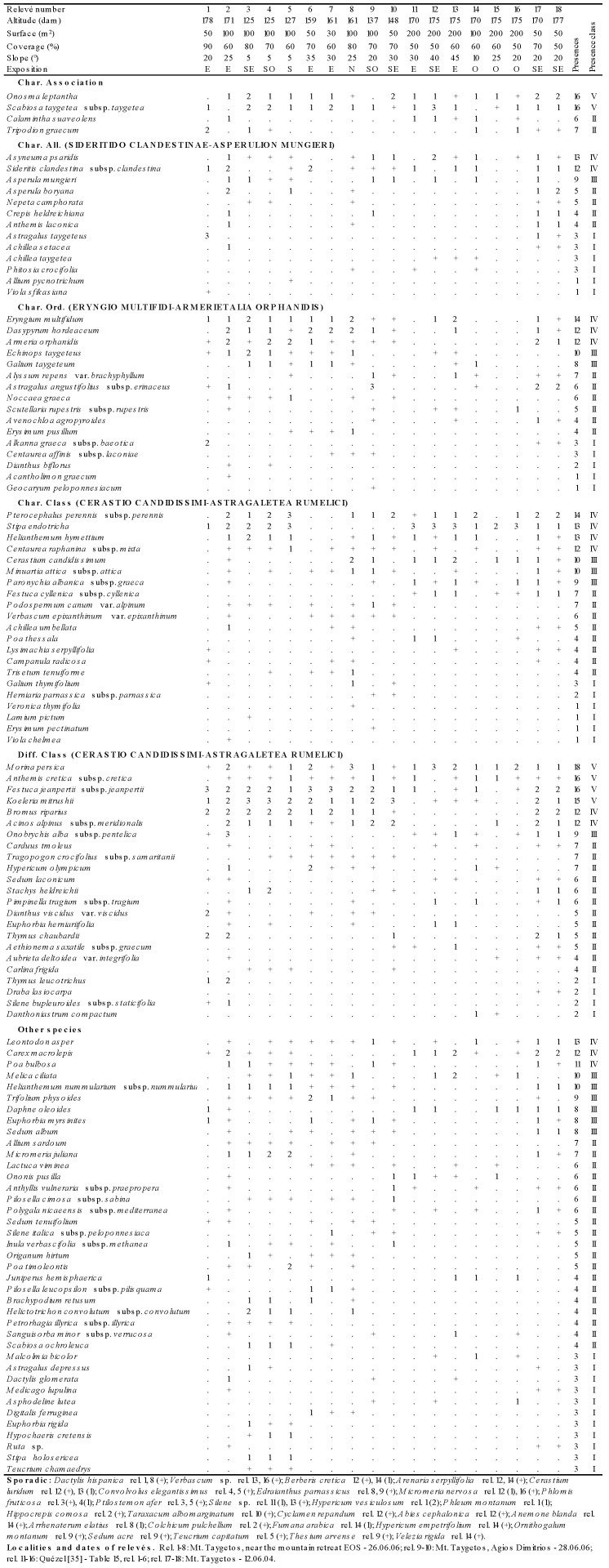
Scabioso taygeteae-Onosmetum leptanthae Quézel 1964.

**Table A50:**
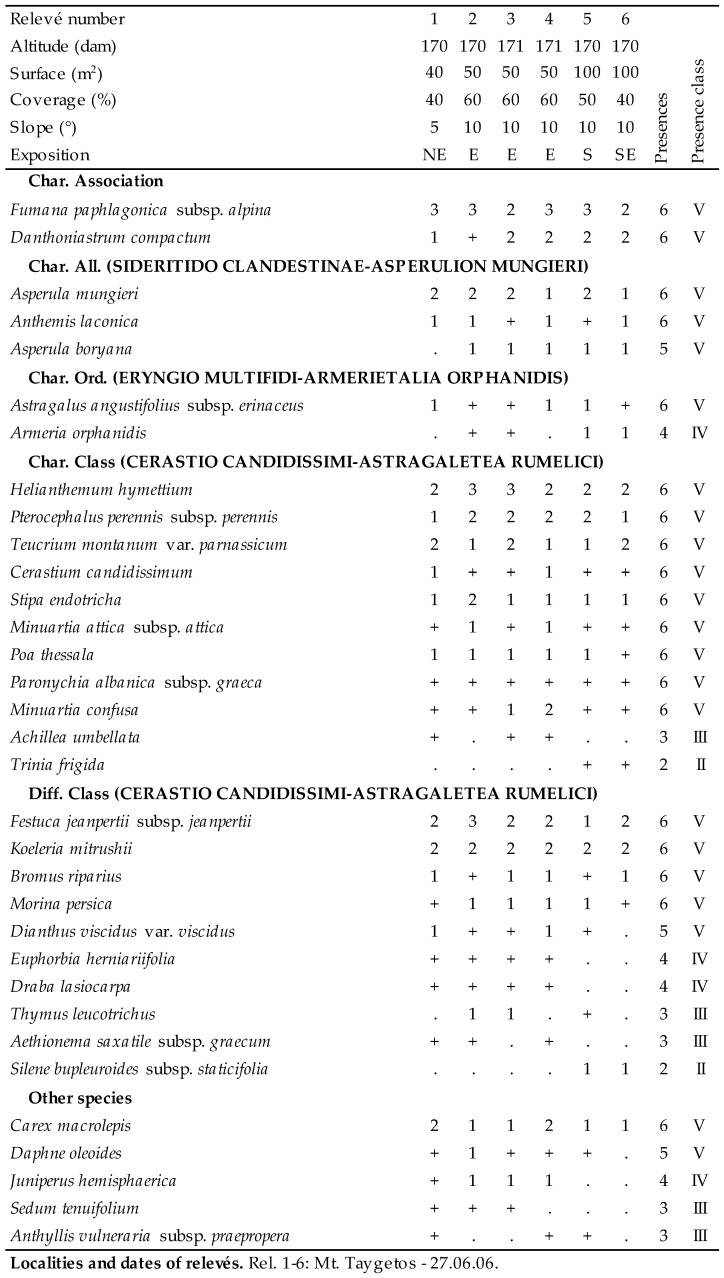
*Danthoniastro compacti-Fumanetum alpinae* Musarella, Brullo & Giusso ass. nov.

**Table A51:**
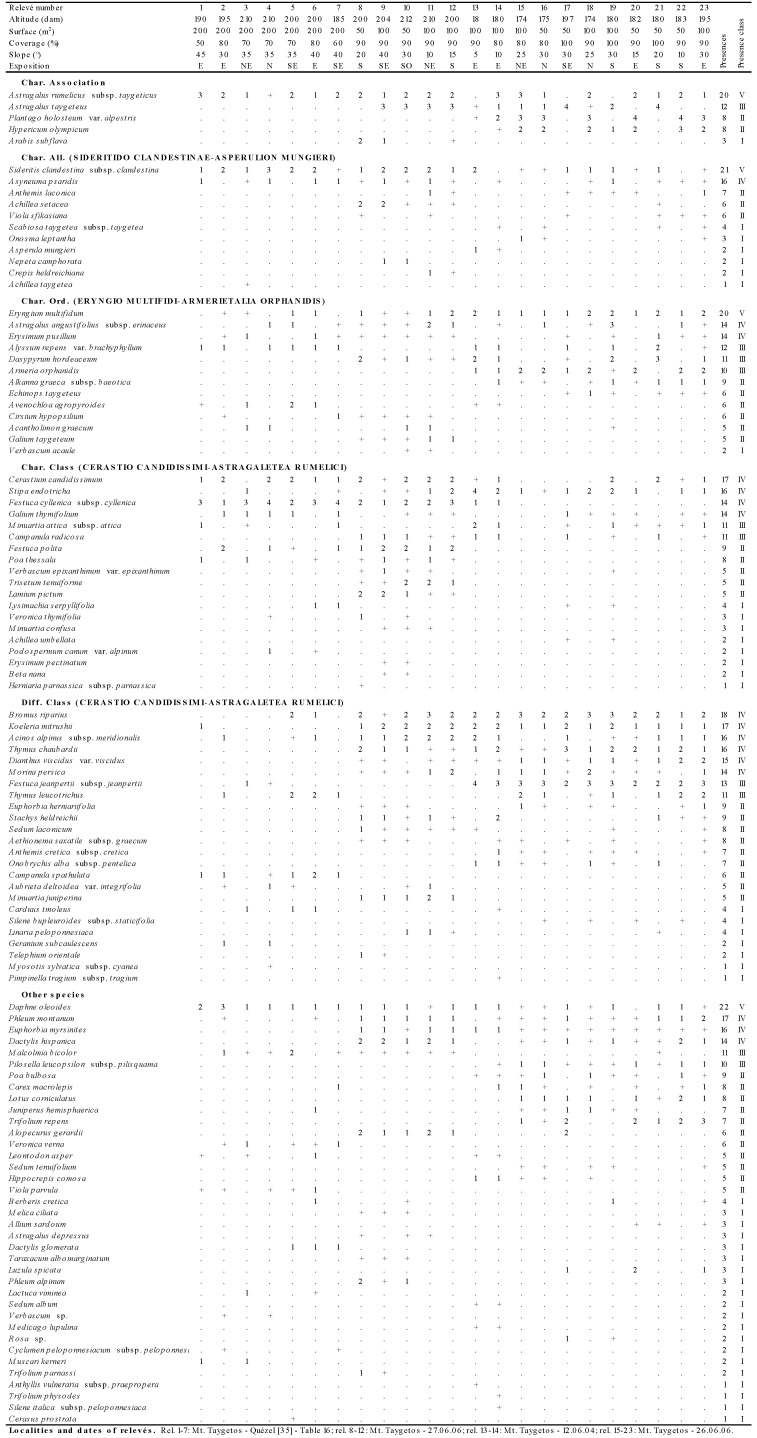
*Sideritido clandestinae-Astragaletum taygetici* Musarella, Brullo & Giusso ass. nov.

**Table A52:**
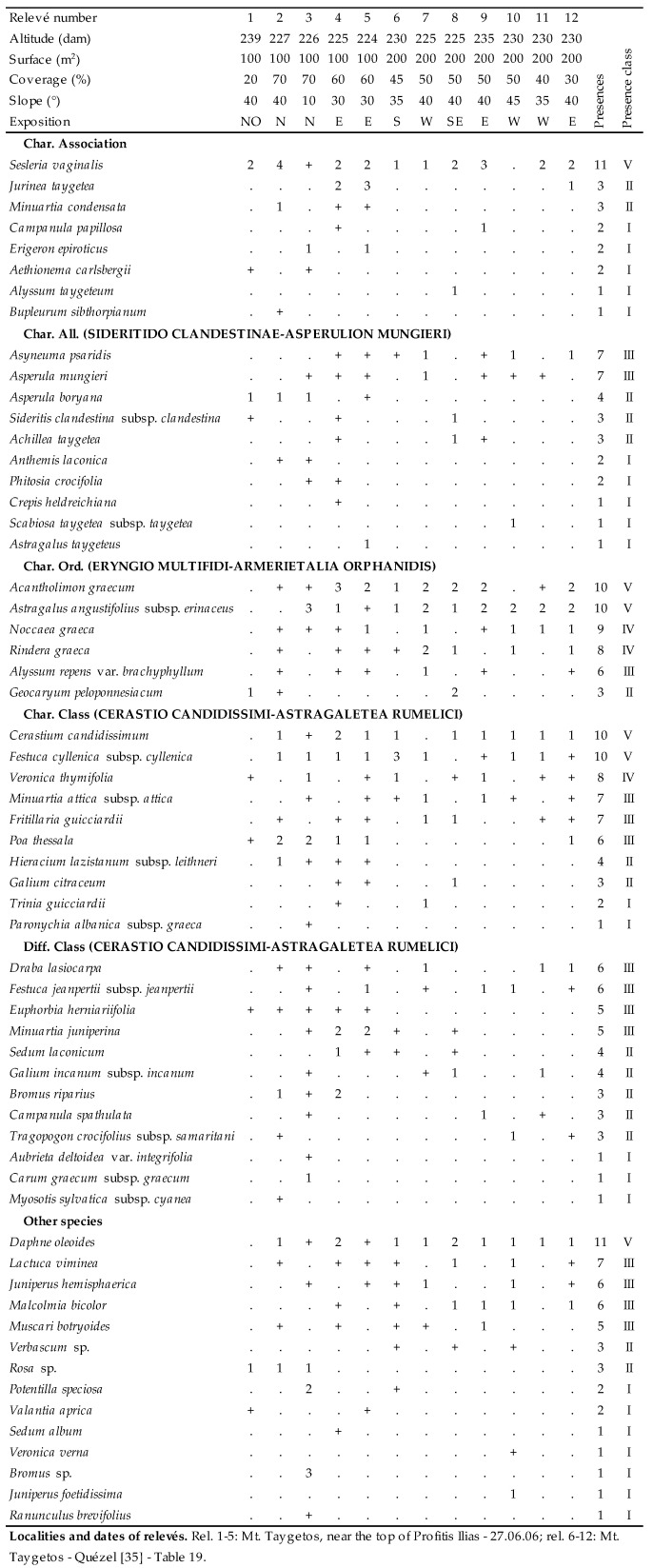
Rindero graecae-Acantholimetum graeci Quézel 1964 corr.

**Table A53:**
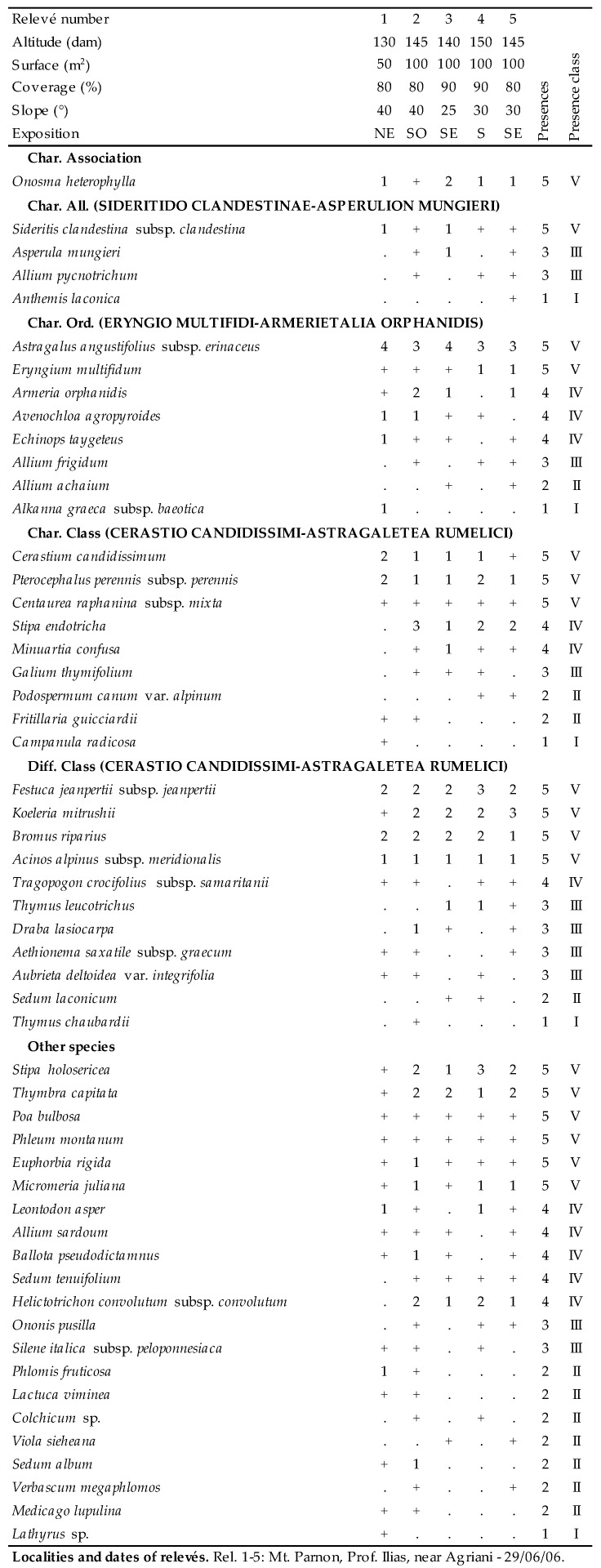
*Onosmo heterophyllae-Astragaletum erinacei* Musarella, Brullo & Giusso ass. nov.

**Table A54:**
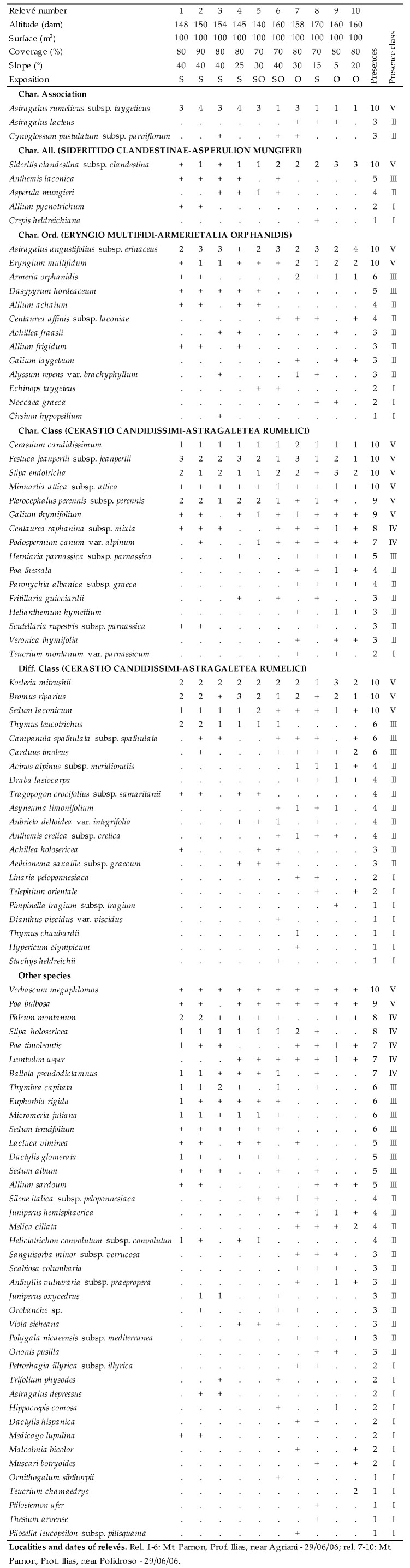
*Astragaletum lacteo-taygetici* Musarella, Brullo & Giusso ass. nov.

**Table A55:**
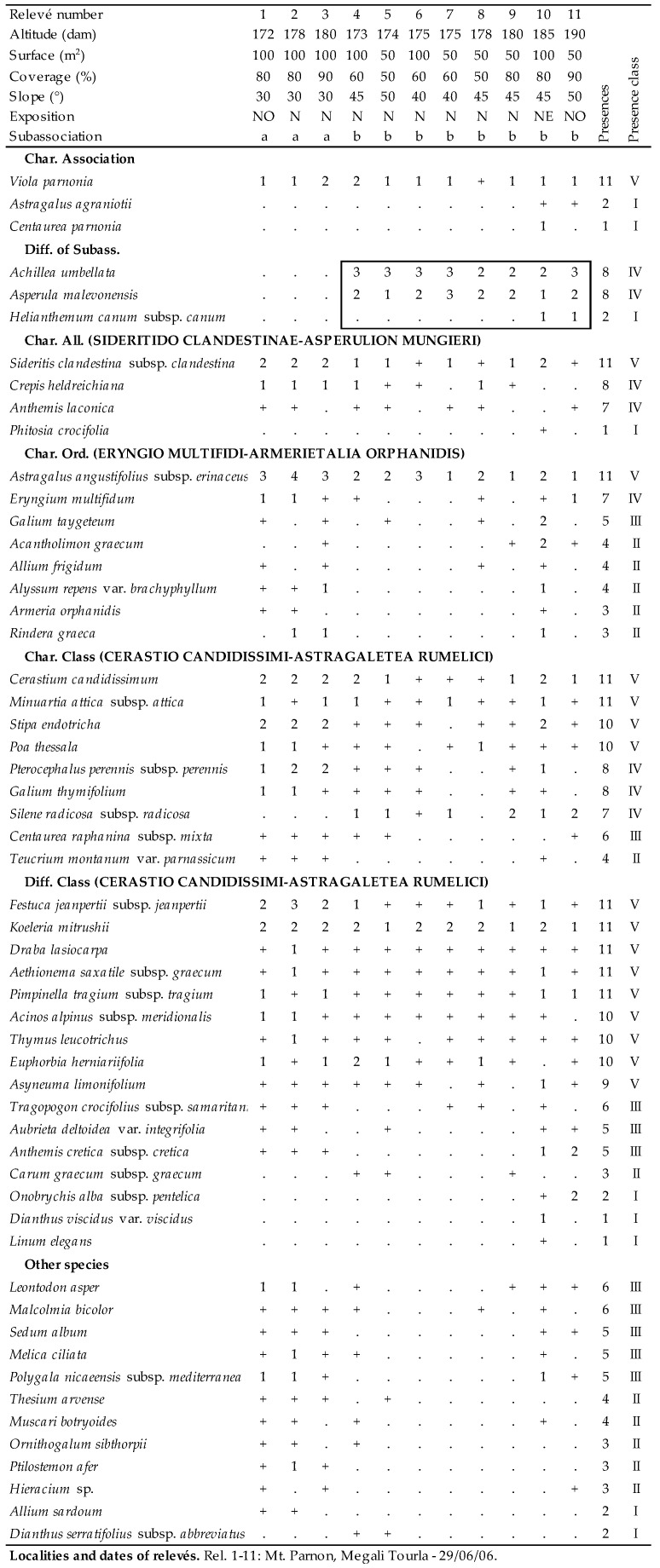
*Violo parnoniae-Astragaletum erinacei* Musarella, Brullo & Giusso ass. nov. (a) *astragaletosum erinacei* Musarella, Brullo & Giusso subass. nov. (b) *asperuletosum malevonensis* Musarella, Brullo & Giusso subass. nov.

**Table A56:**
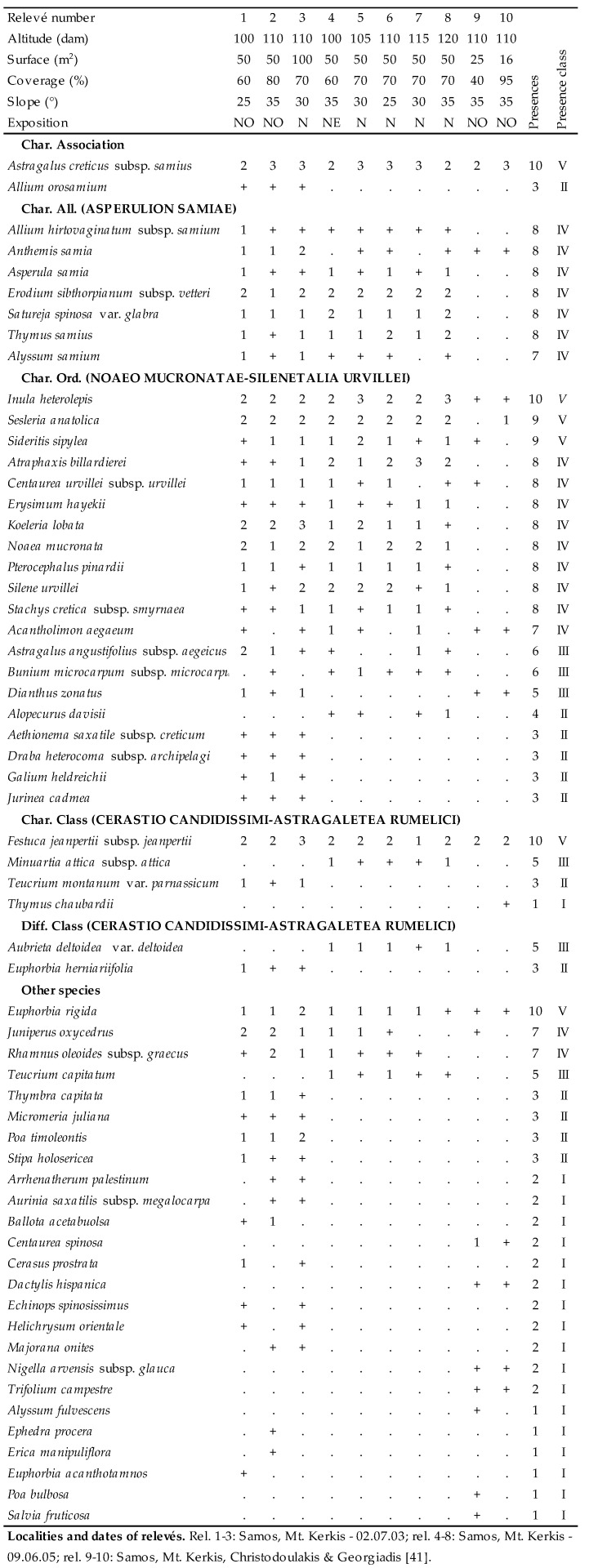
*Astragaletum samii* Musarella, Brullo & Giusso ass. nov.

**Table A57:**
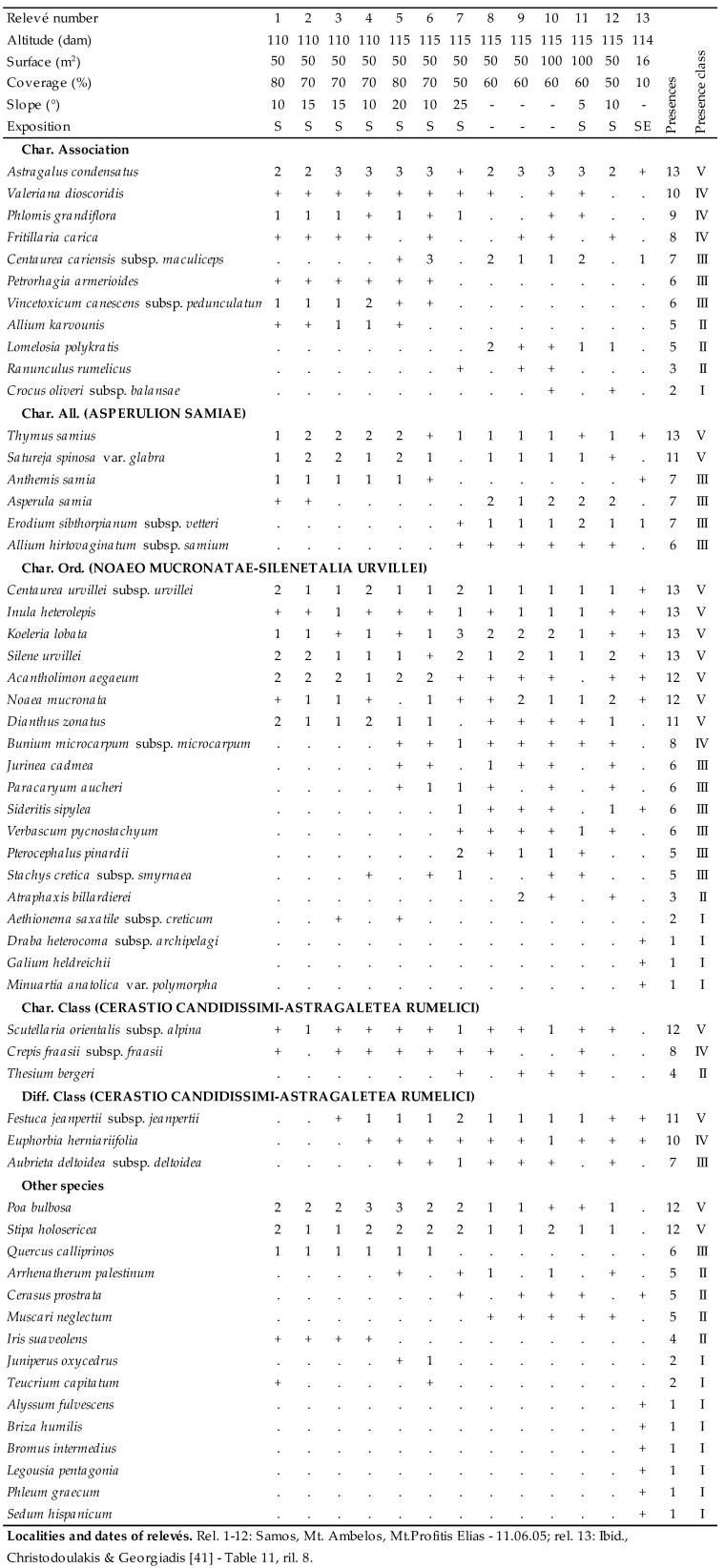
*Thymo samii-Astragaletum condensati* Musarella, Brullo & Giusso ass. nov.

**Table A58:**
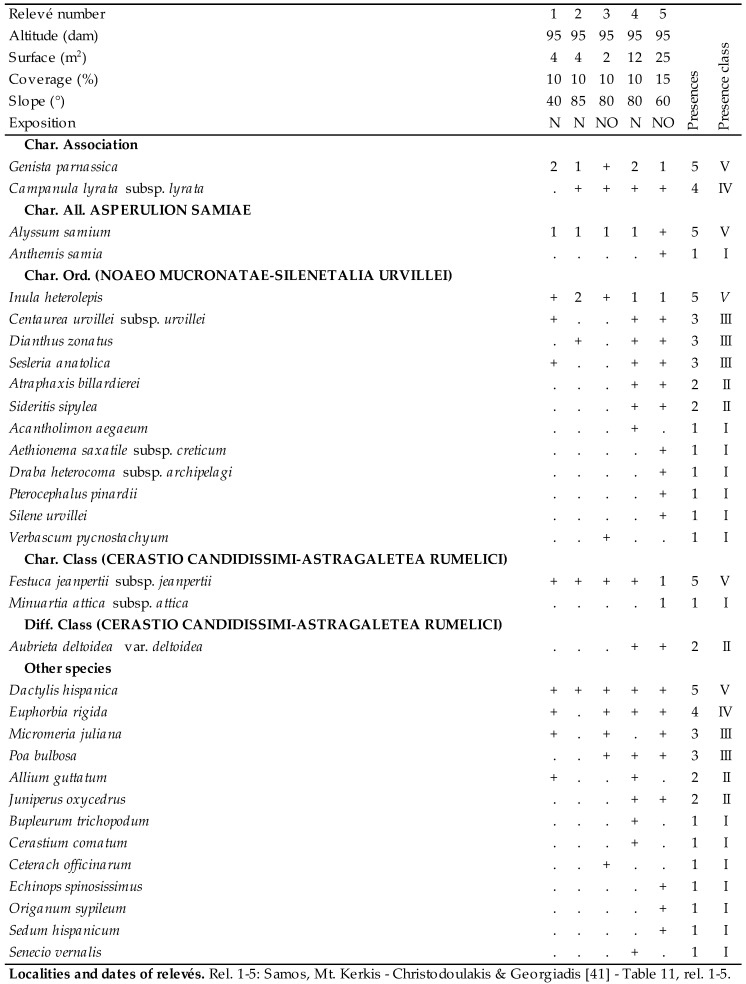
*Campanulo lyratae-Genistetum parnassicae* Musarella, Brullo & Giusso ass. nov.

**Table A59:**
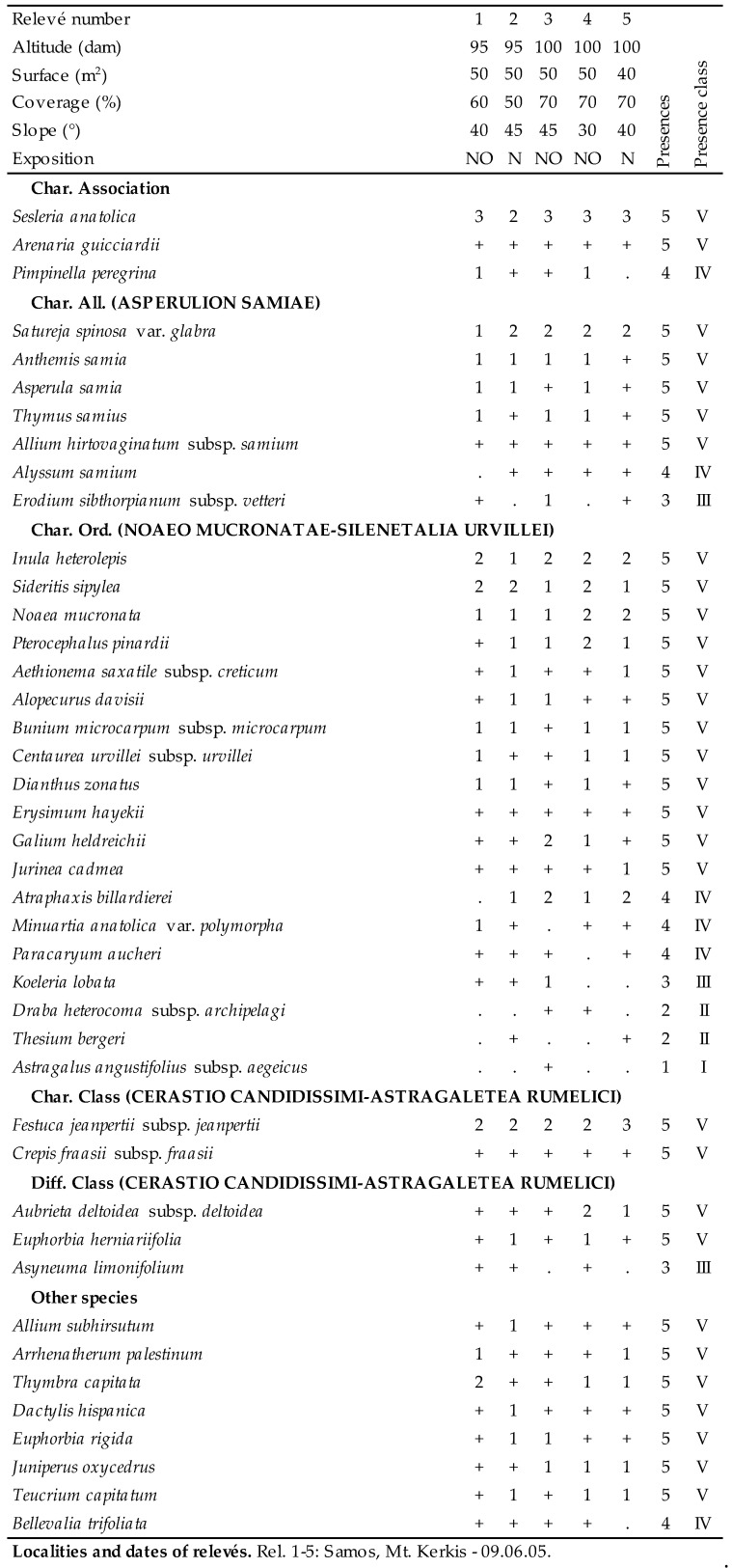
*Arenario guicciardii-Seslerietum anatolicae* Musarella, Brullo & Giusso ass. nov.

**Table A60:**
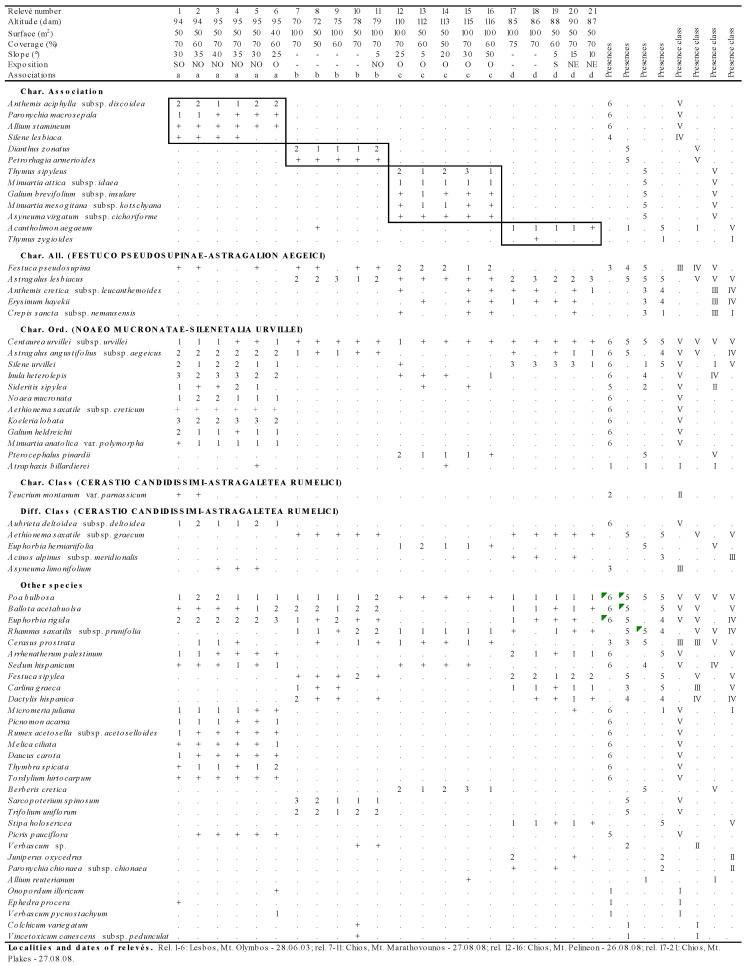
*Anthemido discoideae-Astragaletum aegeici* Musarella, Brullo & Giusso ass. nov. (a); *Diantho zonati-Astragaletum lesbiaci* Musarella, Brullo & Giusso ass. nov. (b); *Galio insularis-Thymetum sypilei* Musarella, Brullo & Giusso ass. nov. (c); *Acantholimo aegaei-Astragaletum lesbiaci* Musarella, Brullo & Giusso ass. nov. (d).

**Table A61:**
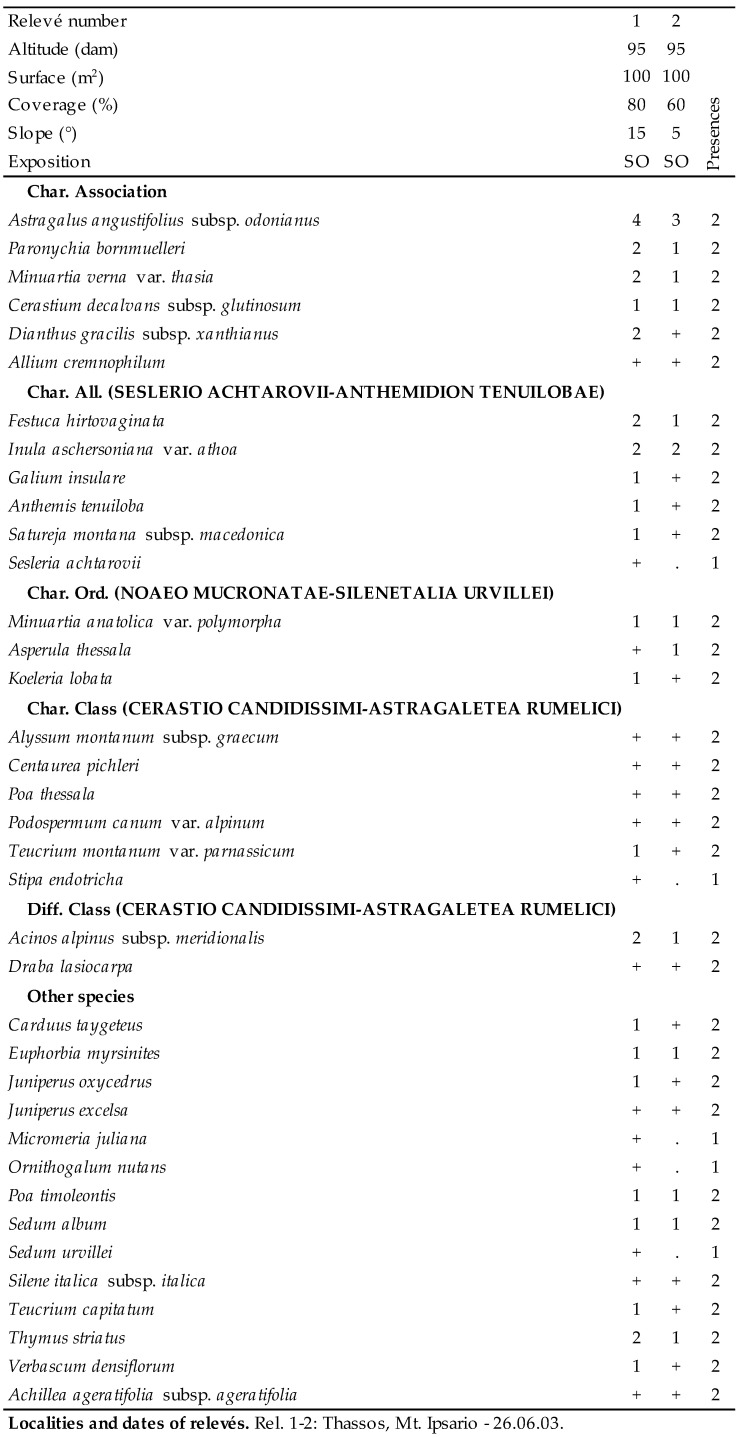
*Paronychio bornmuelleri-Astragaletum odoniani* Musarella, Brullo & Giusso ass. nov.

## Appendix A

**Table A1:** Checklist of the taxa occuring in the phytosociological relevés with their life forms and chorotypes (from [70,71,72,73,74,75,76,77]).

Taxon	Life Form	Chorotype
*Abies cephalonica* Loudon	P	end Greece
*Acantholimon aegaeum* F.K. Mey.	Ch pulv	end E-Aegean
*Acantholimon echinus* Boiss. subsp. *echinus*	Ch pulv	E-medit
*Acantholimon graecum* F.K. Mey.	Ch pulv	end Balkan
*Achillea ageratifolia* (Sm.) Benth. & Hook. f. subsp. *ageratifolia*	H caesp	end Balkan
*Achillea fraasii* Schultz Bip.	H caesp	E-medit
*Achillea holosericea* Sm.	H caesp	end Balkan
*Achillea nobilis* L.	H caesp	eurosiberian
*Achillea setacea* Waldst. & Kit.	H scap	E-medit
*Achillea taygetea* Boiss. & Heldr.	Ch suffr	end Peloponnese
*Achillea umbellata* Sm.	Ch suffr	end Greece
*Acinos alpinus* (L.) Moench subsp. *meridionalis* (Nyman) Ball	Ch suffr	circum-medit
*Acinos arvensis* (Lam.) Dandy	T scap	circum-medit
*Aethionema carlsbergii* Strid & Papanicolau	Ch suffr	end Peloponnese
*Aethionema saxatile* (L.) R. Br. subsp. *creticum* (Boiss. & Heldr.) Andersson et al.	Ch suffr	E-medit
*Aethionema saxatile* (L.) R. Br. subsp. *graecum* (Boiss. & Spruner) Hayek	Ch suffr	E-medit
*Ajuga orientalis* L.	H rhiz	E-medit
*Alkanna graeca* Boiss. & Spruner subsp. *baeotica* (DC.) Nyman	H caesp	end Greece
*Allium achaium* Boiss. & Orph.	G bulb	end Peloponnese
*Allium cephalonicum* Brullo, Pavone & Salmeri	G bulb	end Ionian islands
*Allium cithaeronis* Bogdanović et al.	G bulb	end Sterea Ellas
*Allium cremnophilum* Brullo, Pavone & Salmeri	G bulb	end N-Aegean
*Allium cylleneum* Brullo, Pavone & Salmeri	G bulb	end Peloponnese
*Allium frigidum* Boiss. & Heldr.	G bulb	end Peloponnese
*Allium guttatum* Steven	G bulb	circum-medit
*Allium hirtovaginatum* Kunth subsp. *samium* Brullo, Pavone & Salmeri	G bulb	end E-Aegean
*Allium karvounis* Brullo, Giusso del Galdo & Musarella	G bulb	end E-Aegean
*Allium orosamium* Brullo, Giusso del Galdo & Musarella	G bulb	end E-Aegean
*Allium parnassicum* (Boiss.) Halácsy	G bulb	end Sterea Ellas
*Allium pycnotrichum* Trigas, Kalpoutzakis & Constantinidis	G bulb	end Peloponnese
*Allium reuterianum* Boiss.	G bulb	end E-Aegean
*Allium rhodopeum* Velen.	G bulb	end Balkan
*Allium sardoum* Moris	G bulb	circum-medit
*Allium stamineum* Boiss. s.l.	G bulb	E-medit
*Allium subhirsutum* L.	G bulb	circum-medit
*Alopecurus davisii* Bor	H caesp	E-medit
*Alopecurus gerardii* Vill.	H caesp	circum-medit
*Alyssum fulvescens* Sm.	T scap	E-medit
*Alyssum montanum* L. subsp. graecum (Halácsy) Hayek	Ch suffr	E-medit
*Alyssum montanum* L. var. *hymettium* Boiss.	Ch suffr	end Sterea Ellas
*Alyssum murale* Waldst. & Kit.	H caesp	circum-medit
*Alyssum repens* Baumgt. var. *brachyphyllum* Halácsy	Ch suffr	end Peloponnese
*Alyssum samium* T.R.Dudley & Christod.	Ch suffr	end E-Aegean
*Alyssum siculum* Jordan	T scap	circum-medit
*Alyssum taygeteum* Heldr.	Ch suffr	end CS Greece
*Anchusa hybrida* Ten.	H scap	circum-medit
*Anemone blanda* Schott & Kotschy	G rhiz	E-medit
*Anthemis aciphylla* Boiss. subsp. *discoidea* Boiss.	Ch suffr	E-medit
*Anthemis arvensis* L.	T scap	cosmop
*Anthemis cretica* L. subsp. *cretica*	H caesp	E-medit
*Anthemis cretica* L. subsp. *leucanthemoides* (Boiss.) Grierson	H caesp	E-medit
*Anthemis laconica* Franzen	Ch suffr	end Peloponnese
*Anthemis orientalis* (L.) Degen	Ch suffr	E-medit
*Anthemis samia* Rech. fil.	Ch suffr	end E-Aegean
*Anthemis spruneri* Boiss. & Heldr.	Ch suffr	end Sterea Ellas
*Anthemis tenuiloba* (DC.) R. Fernandes	Ch suffr	E-medit
*Anthemis tinctoria* L. subsp. *parnassica* (Boiss. & Heldr.) Nyman	H caesp	end Balkan
*Anthoxanthum odoratum* L.	H caesp	cosmop
*Anthoxanthum ovatum* Lag.	T scap	circum-medit
*Anthyllis montana* L. subsp. *jacquinii* (A. Kern.) Hayek	Ch frut	circum-medit
*Anthyllis vulneraria* L. subsp. *bulgarica* (Sagorski) Cullen	H caesp	end Balkan
*Anthyllis vulneraria* L. subsp. *praepropera* (A. Kerner) Bornm.	H caesp	N-medit
*Anthyllis vulneraria* L. subsp. *rubriflora* (DC.) Arcang.	H caesp	circum-medit
*Arabis bryoides* Boiss.	H caesp	end Balkan
*Arabis caucasica* Willd.	H scap	E-medit
*Arabis collina* Ten.	H scap	circum-medit
*Arabis subflava* Jones	Ch frut	end CS Greece
*Arenaria cretica* Sprengel var. *stygia* Boiss. & Heldr.	Ch frut	E-medit
*Arenaria filicaulis* Fenzl subsp. *filicaulis*	Ch frut	end Balkan
*Arenaria filicaulis* Fenzl subsp. *graeca* (Boiss.) Mc Neill	Ch frut	end Balkan
*Arenaria guicciardii* Heldr. ex Boiss.	T scap	end Greece
*Arenaria leptoclados* (Reichenb.) Guss.	T scap	eurasian
*Arenaria serpyllifolia* L.	T scap	eurasian
*Aristolochia rotunda* L.	G rhiz	circum-medit
*Armeria orphanidis* Boiss.	Ch caesp	end CS Greece
*Arrhenatherum palestinum* Boiss.	H caesp	E-medit
*Arum maculatum* L.	G rhiz	euro-medit
*Asperula boissieri* Heldr. ex Boiss.	Ch pulv	end CS Greece
*Asperula boryana* (Walp.) Herendorf	Ch pulv	end Peloponnese
*Asperula lutea* Sibth. & Sm.	Ch suffr	end CS Greece
*Asperula malevonensis* Ehrend. & Schonb.-Temesy	Ch pulv	end Peloponnese
*Asperula mungieri* Boiss. & Heldr.	Ch suffr	end Peloponnese
*Asperula oetaea* (Boiss.) Halácsy	H caesp	end Greece
*Asperula purpurea* (L.) Ehrend. subsp. *apiculata* (Sm.) Ehrend.	Ch frut	end Balkan
*Asperula rigidula* Halácsy	Ch rept	end CS Greece
*Asperula samia* Christod. & Georgiadis	Ch suffr	end E-Aegean
*Asperula suffruticosa* Boiss. & Heldr.	Ch suffr	end NC Greece
*Asperula thessala* Boiss. & Heldr.	Ch suffr	end Greece
*Asphodeline liburnica* (Scop.) Rchb.	G rhiz	E-medit
*Asphodeline lutea* (L.) Reichenb.	G rhiz	euro-medit
*Aster cylleneus* (Boiss. & Orph.) Halácsy	H caesp	end Balkan
*Astragalus agraniotii* Orph.	Ch pulv	end Peloponnese
*Astragalus angustifolius* Lam. subsp. *aegeicus* Brullo, Giusso & Musarella	Ch pulv	end E-Aegean
*Astragalus angustifolius* Lam. subsp. *erinaceus* (C. Presl) Brullo, Giusso & Musarella	Ch pulv	end Greece
*Astragalus angustifolius* Lam. subsp. *odonianus* Brullo, Giusso & Musarella	Ch pulv	end N-Aegean
*Astragalus apollineus* Boiss. & Heldr.	H caesp	end CS Greece
*Astragalus calavrytensis* Beauverd & Topali	Ch pulv	end Peloponnese
*Astragalus cephalonicus* Presl	Ch pulv	end Ionian islands
*Astragalus condensatus* Ledeb.	Ch pulv	E-medit
*Astragalus corynthiacus* Brullo, Giusso & Musarella	Ch pulv	end Sterea Ellas
*Astragalus creticus* Lam. subsp. *samius* (Sirj. & Rech.) Ponert	Ch pulv	end E-Aegean
*Astragalus cylleneus* Boiss. & Heldr.	Ch pulv	end Peloponnese
*Astragalus depressus* L. subsp. *depressus*	H ros	circum-medit
*Astragalus hellenicus* Boiss.	H ros	circum-medit
*Astragalus lacteus* Heldr. & Sart.	H ros	end Balkan
*Astragalus lesbiacus* P. Candargy	Ch pulv	end E-Aegean
*Astragalus monspessulanus* L.	H caesp	circum-medit
*Astragalus parnassi* Boiss.	Ch pulv	end Sterea Ellas
*Astragalus rumelicus* Bunge subsp. *rumelicus*	Ch pulv	E-medit
*Astragalus rumelicus* Bunge subsp. *euboicus* (Širj.) Brullo, Giusso & Musarella	Ch pulv	end Euboea
*Astragalus rumelicus* Bunge subsp. *taygeticus* (Širj.) Brullo, Giusso & Musarella	Ch pulv	end Peloponnese
*Astragalus taygeteus* Persson & Strid	Ch pulv	end Peloponnese
*Astragalus tymphresteus* Boiss. & Spruner	Ch pulv	end NC Greece
*Asyneuma limonifolium* (L.) Jancken	H scap	E-medit
*Asyneuma psaridis* (Halácsy) Bornm.	H scap	end Peloponnese
*Asyneuma virgatum* (Labill.) Bornm. subsp. *cichoriforme* (Boiss.) Damboldt	H scap	E-medit
*Atraphaxis billardierei* Jaub. & Spach	Ch pulv	E-medit
*Aubrieta deltoidea* (L.) DC. subsp. *deltoidea*	Ch suffr	E-medit
*Aubrieta deltoidea* (L.) DC. var. *integrifolia* F. & M.	Ch suffr	E-medit
*Aubrieta deltoidea* (L.) DC. subsp. *intermedia* (Heldr. & Orph.) M. & P.	Ch suffr	E-medit
*Aurinia gionae* (Quézel & Contandr.) Greuter & Burdet	Ch frut	end NC Greece
*Aurinia moreana* Tzanoud. & Iatroù	H caesp	end Peloponnese
*Aurinia saxatilis* (L.) Desv. subsp. *megalocarpa* (Hausskn.) Dudley	Ch frut	E-medit
*Aurinia saxatilis* (L.) Desv. subsp. *orientalis* (Ard.) T.R. Dudley	H caesp	end Balkan
*Avenochloa agropyroides* (Boiss.) Holub	H caesp	E-medit
*Ballota acetabuolsa* (L.) Bentham	Ch frut	E-medit
*Ballota pseudodictamnus* Benth.	Ch frut	E-medit
*Bellevalia trifoliata* (Ten.) Kunth	G bulb	circum-medit
*Bellis perennis* L.	H ros	paleotemp
*Berberis cretica* L.	NP	E-medit
*Beta nana* Boiss. & Heldr.	H ros	end CS Greece
*Bolanthus graecus* (Schreb.) Barkoudah	Ch frut	end Greece
*Botrychium lunaria* (L.) Scwartz	T scap	cosmop
*Brachypodium retusum* P. Beauv.	H caesp	circum-medit
*Briza humilis* M. Bieb.	T scap	E-medit
*Bromus intermedius* Guss.	T scap	circum-medit
*Bromus lacmonicus* Hausskn.	H caesp	E-medit
*Bromus riparius* Rehm.	H caesp	E-medit
*Bromus squarrosus* L.	T scap	paleotemp
*Bromus tectorum* L.	T scap	paleotemp
*Bunium microcarpum* (Boiss.) Freyn subsp. *microcarpum*	G bulb	E-medit
*Bupleurum falcatum* L. subsp. *cernuum* (Ten.) Arcangeli	H scap	euro-medit
*Bupleurum glumaceum* Sibth. & Sm.	T scap	E-medit
*Bupleurum sibthorpianum* Sibth. & Sm.	Ch frut	end CS Greece
*Bupleurum trichopodum* Boiss. & Spruner	T scap	E-medit
*Cachrys ferulacea* (L.) Calestani	H scap	E-medit
*Calamintha suaveolens* Boiss.	Ch suffr	E-medit
*Campanula aizoides* Zaffran ex Greuter	H bienn	end Greece
*Campanula albanica* Witasek subsp. *albanica*	H scap	end Balkan
*Campanula lyrata* Lam. subsp. *lyrata*	H scap	E-medit
*Campanula papillosa* Halácsy	H scap	end Peloponnese
*Campanula radicosa* Bory & Chaub.	H rept	end CS Greece
*Campanula spathulata* Sibth. & Sm. subsp. *spathulata*	G rhiz	end Balkan
*Capsella bursa-pastoris* L.	T scap	euro-medit
*Carduus nutans* L. subsp. *scabrisquamus* Arènes	H bienn	circum-medit
*Carduus taygeteus* (Boiss. & Heldr.) Hayek	H bienn	circum-medit
*Carduus tmoleus* Boiss.	H bienn	end Balkan
*Carex caryophyllea* Latourr.	H caesp	eurasian
*Carex kitaibeliana* Degen ex Becherer	H caesp	circum-medit
*Carex macrolepis* DC.	H caesp	eurasian
*Carlina frigida* Boiss. & Heldr.	H scap	E-medit
*Carlina graeca* Heldr. & Sartori	H scap	end Balkan
*Carum graecum* Boiss. & Heldr. subsp. *graecum*	H scap	end Balkan
*Carum meoides* (Griseb.) Halacsy	H scap	end Balkan
*Centaurea affinis* Friv. subsp. *affinis*	H scap	end Balkan
*Centaurea affinis* Friv. subsp. *laconiae* Prodan	H scap	end CS Greece
*Centaurea affinis* Friv. subsp. *pallidior* (Halácsy) Hayek	H scap	end Balkan
*Centaurea cariensis* Boiss. subsp. *maculiceps* (Schwarz) Wagenitz	Ch suffr	E-medit
*Centaurea parnonia* Halácsy	H caesp	end Peloponnese
*Centaurea pichleri* Boiss.	H caesp	E-medit
*Centaurea raphanina* Sibth. & Sm. subsp. *mixta* (DC.) Runem.	H ros	end Greece
*Centaurea spinosa* L.	Ch frut	E-medit
*Centaurea spruneri* Boiss. & Heldr. subsp. *guicciardi* (Boiss.) Hayek	H caesp	end Balkan
*Centaurea subciliaris* Boiss. & Heldr. subsp. *subciliaris*	H caesp	end Balkan
*Centaurea urvillei* DC. subsp. *urvillei*	H caesp	E-medit
*Cerastium brachypetalum* Pers. subsp. *roeseri* (Boiss. & Heldr.) Nyman	T scap	E-medit
*Cerastium candidissimum* Correns	Ch frut	end Greece
*Cerastium decalvans* Schlosser & Wuk. subsp. *glutinosum* (Strid) Niketic	H caesp	end N-Aegean
*Cerastium illyricum comatum* Desv.	T scap	E-medit
*Cerasus prostrata* (Labill.) Ser.	NP	circum-medit
*Ceterach officinarum* Willd.	H ros	eurasian
*Chamaecytisus hirsutus* (L.) Link	Ch suffr	euro-medit
*Cirsium hypopsilium* Boiss. & Heldr.	H bienn	end Peloponnese
*Colchicum graecum* K. Persson	G bulb	end CS Greece
*Colchicum variegatum* L.	G bulb	E-medit
*Convolvulus althaeoides* L.	H rept	cosmop
*Convolvulus boissieri* Steud. subsp. *parnassicus* (Boiss. & Orph.) Kuzmanov	Ch rept	end Balkan
*Convolvulus cantabrica* L.	Ch frut	eurasian
*Convolvulus elegantissimus* Miller	H rept	circum-medit
*Corydalis solida* (L.) Clairv. subsp. *incisa* Liden	G bulb	end Balkan
*Crataegus monogyna* Jacq.	NP	paleotemp
*Crataegus orientalis* Pallas ex Bieb.	NP	euro-medit-irano-turan
*Crataegus pycnoloba* Boiss. & Heldr.	NP	eurasian
*Crepis fraasii* Schultz-Bip. subsp. *fraasii*	H ros	E-medit
*Crepis heldreichiana* (O. Kuntze) Greuter	H scap	end Peloponnese
*Crepis incana* Sm.	H scap	end CS Greece
*Crepis neglecta* L.	T scap	end Balkan
*Crepis rubra* L.	T scap	circum-medit
*Crepis sancta* (L.) Babcock subsp. *nemausensis*	T scap	E-medit
*Crepis sancta* (L.) Babcock subsp. *sancta*	T scap	medit-irano-turan
*Crocus oliveri* Gay subsp. *balansae* (Baker) Mathew	G bulb	E-medit
*Crupina crupinastrum* (Moris) Vis.	T scap	eurasian
*Cyclamen peloponnesiacum* (Grey-Wilson) Kit Tan subsp. *peloponnesiacum*	G bulb	end CS Greece
*Cynoglossum montanum* L.	H scap	eurasian
*Cynoglossum pustulatum* Boiss. subsp. *parviflorum* (Vis.) Sutory	H scap	end Balkan
*Cynosurus echinatus* L.	T scap	circum-medit
*Cytisus villosus* Pourr.	NP	circum-medit
*Dactylis glomerata* L.	H caesp	paleotemp
*Dactylis hispanica* Roth	H caesp	circum-medit
*Dactylorhiza sambucina* (L.) Soó	G bulb	european
*Danthoniastrum compactum* (Boiss. & Heldr.) Holub.	H caesp	E-medit
*Daphne oleoides* Schreber	Ch frut	circum-medit
*Dasypyrum hordeaceum* Cand.	H caesp	circum-medit
*Dasypyrum villosum* (L.) Borbás	T scap	medit-asian
*Daucus carota* L.	H bienn	paleotemp
*Dianthus androsaceus* (Boiss. & Heldr.) Hayek	Ch suffr	end Peloponnese
*Dianthus biflorus* Sm.	Ch suffr	end Greece
*Dianthus gracilis* Sm. subsp. *xanthianus* (Davidov) Tutin	Ch suffr	end NC Greece
*Dianthus haematocalyx* Boiss. & Heldr. subsp. *ventricosus* Maire & Petitm.	Ch suffr	end Sterea Ellas
*Dianthus integer* Vis. subsp. *minutiflorus* (Halácsy) Bornm.	Ch suffr	end Balkan
*Dianthus serratifolius* Sm. subsp. *abbreviatus* (Heldr. ex Halácsy) Strid	Ch suffr	end Peloponnese
*Dianthus serratifolius* Sm. subsp. *serratifolius*	Ch suffr	end CS Greece
*Dianthus tymphresteus* (Boiss. & Spruner) Boiss.	Ch suffr	end CS Greece
*Dianthus viscidus* Bory & Chaub. var. *viscidus*	Ch suffr	E-medit
*Dianthus viscidus* Bory & Chaub. var. *parnassicus* (Boiss. & Heldr.) Boiss.	Ch suffr	end Sterea Ellas
*Dianthus zonatus* Fenzl	Ch suffr	E-medit
*Dichoropetalum vittijugum* (Boiss.) Pimenov & Kljuykov	H scap	end Balkan
*Digitalis ferruginea* L.	H scap	medit-irano-turan
*Digitalis laevigata* Waldst. & Kit.	H scap	end Balkan
*Doronicum columnae* Ten.	G rhiz	euro-medit
*Dorycnium germanicum* (Gremli) Rikli	H caesp	european
*Dorycnium herbaceum* Vill.	H caesp	circum-medit
*Draba heterocoma* Fenzl subsp. *archipelagi* (O.E. Schulz) Buttler	Ch pulv	E-medit
*Draba lacaitae* Boiss.	Ch pulv	end Balkan
*Draba lasiocarpa* Rochel	Ch frut	european
*Draba parnassica* Boiss. & Heldr.	Ch pulv	end Sterea Ellas
*Drypis spinosa* L.	Ch pulv	european
*Echinops spinosissimus* Turra	H scap	circum-medit
*Echinops taygeteus* Boiss. & Heldr.	H scap	end CS Greece
*Edraianthus graminifolius* (L.) DC. f. *minor*	Ch suffr	end Balkan
*Elytrigia intermedia* (Host) Nevski	G rhiz	eurasian
*Ephedra procera* Fischer & C.A. Meyer	NP	E-medit
*Erica manipuliflora* Salisb.	Ch frut	E-medit
*Erigeron alpinus* L.	H scap	euro-medit
*Erigeron epiroticus* (Vierh.) Halácsy	H scap	E-medit
*Erigeron glabratus* Hoppe & Hornsc. ex Bluff & Fingerh. subsp. *graecus* Vierh.	H scap	end NC Greece
*Erodium chrysanthum* L’Her. ex DC.	H caesp	end CS Greece
*Erodium sibthorpianum* Boiss. subsp. *vetteri* (Barbey & Major) Davis	H caesp	end E-Aegean
*Erophila verna* (L.) Chevall.	T scap	paleotemp
*Eryngium campestre* L.	H scap	euro-medit
*Eryngium multifidum* Sibth. & Sm.	T scap	N-medit
*Erysimum cephalonicum* Polatschek	H scap	end Greece
*Erysimum cuspidatum* (Bieb.) DC.	H bienn	medit-irano-turan
*Erysimum hayekii* (Jav. & Rech. fil.) Polatschek	H scap	E-medit
*Erysimum linearifolium* Tausch	H caesp	end Balkan
*Erysimum microstylum* Ausskn.	H caesp	end Balkan
*Erysimum parnassi* (Boiss. & Heldr.) Hausskn.	Ch suffr	end Sterea Ellas
*Erysimum pectinatum* Bory & Chaub.	H scap	end Peloponnese
*Erysimum pusillum* Bory & Chaub.	Ch suffr	end Peloponnese
*Euphorbia acanthotamnos* Heldr. & Sart. ex Boiss.	Ch frut	E-medit
*Euphorbia deflexa* Sibth. & Sm.	Ch suffr	E-medit
*Euphorbia herniariifolia* Willd.	G rhiz	E-medit
*Euphorbia myrsinites* L.	Ch rept	E-medit
*Euphorbia rigida* Bieb.	Ch suffr	E-medit
*Euphrasia salisburgensis* Funk.	T scap	euro-medit
*Festuca callieri* (St.-Yves) Markgr. subsp. *callieri*	H caesp	eurasian
*Festuca cyllenica* Boiss. & Heldr. subsp. *cyllenica*	H caesp	end CS Greece
*Festuca graeaca* (Hackel) Markgraf subsp. *graeca*	H caesp	end NC Greece
*Festuca halleri* All. subsp. *riloensis* Hack.	H caesp	E-medit
*Festuca hirtovaginata* (Acht.) Markgr.-Dannenb.	H caesp	E-medit
*Festuca jeanpertii* (St.-Yves) Markgr. subsp. *achaica* (I.Markgraf- Dannenberg) Markgr.-Dann.	H caesp	E-medit
*Festuca jeanpertii* (St-Yves) Markgraf subsp. *jeanpertii*	H caesp	E-medit
*Festuca olympica* Vetter	H caesp	end NC Greece
*Festuca polita* (Halácsy) Tzelev	H caesp	end CS Greece
*Festuca pseudosupina* Vetter	H caesp	end E-Aegean
*Festuca sipylea* (Hack.) Markgr.-Dann.	H caesp	E-medit
*Festuca spectabilis* Jan subsp. *affinis* (Boiss. & Heldr. ex Hackel) Hackel	H caesp	E-medit
*Filago arvensis* L.	T scap	paleotemp
*Fritillaria carica* Rix	G bulb	E-medit
*Fritillaria graeca* Boiss. & Spruner var. *graeca*	G bulb	end Balkan
*Fritillaria guicciardii* Heldr. & Sart.	G bulb	end CS Greece
*Fumana paphlagonica* Bornm. & Janchen subsp. *alpina* (Janchen) Greuter	Ch suffr	E-medit
*Fumana procumbens* (Dunal) Gren. & Godron	Ch suffr	euro-medit-irano-turan
*Gagea villosa* (M. Bieb.) Sweet	G bulb	eurasian
*Galium brevifolium* Sm. subsp. *insulare* Ehrend. & Schönb.-Tem.	T scap	E-medit
*Galium circae* Krendl	Ch suffr	end Greece
*Galium citraceum* Boiss.	Ch suffr	end CS Greece
*Galium cyllenium* Boiss. & Heldr.	Ch pulv	end Peloponnese
*Galium heldreichii* Halácsy	H scap	E-medit
*Galium incanum* Sibth. & Sm. subsp. *incanum*	Ch pulv	E-medit
*Galium insulare* Krendl	Ch suffr	end N-Aegean
*Galium ionicum* Krendl	H caesp	end Ionian islands
*Galium plebeium* Boiss. & Heldr.	Ch pulv	E-medit
*Galium taygeteum* Krendl	Ch suffr	end Peloponnese
*Galium thymifolium* Boiss. & Heldr.	H caesp	end CS Greece
*Genista parnassica* Halácsy	Ch pulv	end Greece
*Geocaryum parnassicum* (Boiss. & Heldr.) Engstrand	G bulb	end CS Greece
*Geocaryum peloponnesiacum* Engstrand	G bulb	end Peloponnese
*Geranium macrostylum* Boiss.	G rhiz	E-medit
*Geranium pyrenaicum* Burm. fil.	H scap	paleotemp
*Geranium subcaulescens* L’Her.	H scap	E-medit
*Globularia cordifolia* L.	Ch frut	euro-medit
*Globularia stygia* Orph.	Ch frut	end Peloponnese
*Halacsyella parnassica* (Boiss. & Spruner) Janch.	Ch suffr	end CS Greece
*Helianthemum canum* (L.) Baumg. subsp. *canum*	Ch suffr	euro-medit
*Helianthemum hymettium* Boiss. & Heldr.	Ch suffr	E-medit
*Helianthemum nummularium* (L.) Miller subsp. *nummularium*	Ch suffr	euro-medit
*Helichrysum orientale* (L.) DC.	Ch suffr	E-medit
*Helictotrichon aetolicum* (Rech. fil.) Holub	H caesp	E-medit
*Helictotrichon convolutum* (Presl) Henrard subsp. *convolutum*	H caesp	E-medit
*Helictotrichon convolutum* (Presl) Henrard subsp. *heldreichii* (Parl.) Gervais	H caesp	E-medit
*Helleborus cyclophyllus* A. Brown	G rhiz	end Balkan
*Herniaria incana* Lam.	T scap	eurasian
*Herniaria parnassica* Heldr. & Sart. subsp. *parnassica*	Ch suffr	E-medit
*Hieracium lazistanum* Arv.-Touv. subsp. *leithneri* (Boiss.) Greuter	H scap	E-medit
*Hieracium pannosum* Boiss. subsp. *euboeum* (Halácsy) Zahn	H scap	end Greece
*Hieracium sartorianum* Boiss. & Heldr.	H ros	end CS Greece
*Hippocrepis comosa* L.	Ch rept	euro-medit
*Hordeum bulbosum* L.	H scap	paleotemp
*Hypericum olympicum* L.	Ch frut	end CS Greece
*Hypochaeris cretensis* (L.) Bory & Chaub.	H ros	E-medit
*Iberis saxatilis* L. subsp. *saxatilis*	Ch suffr	N-medit
*Iberis sempervirens* L.	Ch suffr	circum-medit
*Inula aschersoniana* Janka var. *athoa* Rech.	Ch suffr	end N-Aegean
*Inula britannica* L	H caesp	european
*Inula candida* (L.) Cass. subsp. *limonella* (Heldr.) Rech. f.	H caesp	end Greece
*Inula heterolepis* Boiss.	Ch suffr	E-medit
*Inula verbascifolia* (Willd.) Hausskn. subsp. *methanea* (Hausskn.) Tutin.	Ch suffr	end Greece
*Iris attica* Boiss. & Heldr.	G rhiz	E-medit
*Iris suaveolens* Boiss. & Reut.	G rhiz	E-medit
*Juniperus excelsa* Bieb.	NP	E-medit
*Juniperus foetidissima* Willd.	NP	E-medit
*Juniperus hemisphaerica* Presl	NP	circum-medit
*Juniperus oxycedrus* L.	P	circum-medit
*Jurinea cadmea* Boiss.	H ros	E-medit
*Jurinea mollis* (L.) Rchb.	H ros	european
*Jurinea taygetea* Halácsy	H ros	end Peloponnese
*Koeleria carniolica* A. Kerner	H caesp	E-medit
*Koeleria lobata* (Bieb.) Roemer & Schultes	H caesp	E-medit
*Koeleria mitrushii* Ujhelyi	H caesp	end Balkan
*Lactuca intricata* Boiss.	H scap	E-medit
*Lactuca viminea* (L.) J. & C. Presl	H scap	paleotemp
*Lamium pictum* Boiss. & Heldr.	H scap	end Greece
*Lamium striatum* Sibth. & Sm.	H scap	E-medit
*Laserpitium pseudomeum* Orph. & al.	H ros	end CS Greece
*Lathyrus grandiflorus* Sm.	H scap	circum-medit
*Legousia pentagonia* Druce	T scap	E-medit
*Leontodon asper* (Waldst. & Kit) Poiret	H ros	N-medit
*Leontodon cichoriaceus* (Ten.) Sanguinetti	H caesp	E-medit
*Leontodon graecus* Boiss. & Heldr.	H scap	E-medit
*Lepidium nebrodense* (Raphin.) Guss.	H scap	E-medit
*Linaria parnassica* Boiss. & Heldr.	H scap	E-medit
*Linaria peloponnesiaca* Boiss. & Heldr.	H scap	E-medit
*Linaria simplex* Desf.	T scap	circum-medit
*Linum elegans* Spruner ex Boiss.	Ch suffr	E-medit
*Linum tenuifolium* L.	Ch suffr	euro-medit
*Lolium perenne* L.	H caesp	circumboreal
*Lomelosia crenata* (Cirillo) Greuter & Burdet subsp. *crenata*	Ch frut	circum-medit
*Lomelosia graminifolia* (L.) Greuter & Burdet	H caesp	euro-medit
*Lomelosia polykratis* (Rech.f.) Greuter & Burdet	Ch frut	E-medit
*Lotus stenodon* (Boiss. & Heldr.) Heldr.	H scap	end Balkan
*Luzula spicata* (L.) DC.	H caesp	paleotemp
*Lychnis coronaria* (L.) Desr.	H scap	circum-medit
*Lysimachia serpyllifolia* Schreber	H scap	E-medit
*Macrotomia cephalotes* Boiss.	H scap	E-medit
*Majorana onites* Benth.	Ch suffr	E-medit
*Malcolmia bicolor* Bald.	T scap	end CS Greece
*Marrubium cylleneum* Boiss. & Heldr.	H scap	end Peloponnese
*Marrubium velutinum* Sibth. & Sm.	H scap	end NC Greece
*Medicago lupulina* L.	T scap	paleotemp
*Medicago sativa* L.	H caesp	eurasian
*Melica ciliata* L.	H caesp	euro-medit
*Micromeria juliana* (L.) Bentham ex Reichenb.	Ch suffr	circum-medit
*Minuartia anatolica* (Boiss.) Woron. var. *polymorpha* McNeill	Ch suffr	E-medit
*Minuartia attica* Vierh. subsp. *attica*	Ch suffr	E-medit
*Minuartia attica* Vierh. subsp. *idaea* (Halácsy) Kamari & Constantin	Ch suffr	E-medit
*Minuartia condensata* (J. & C. Presl) Hand.-Mazz.	Ch pulv	N-medit
*Minuartia confusa* (Boiss.) Maire & Petitm.	Ch rept	end CS Greece
*Minuartia juniperina* (L.) Maire & Petitm.	Ch pulv	E-medit
*Minuartia mesogitana* (Boiss.) Hand.-Mazz. subsp. *kotschyana* (Boiss.) McNeill	T scap	E-medit
*Minuartia stellata* (Clarke) Maire & Petitm.	Ch pulv	end Balkan
*Minuartia verna* (L.) Hiern var. *thasia* Stoj. & Kitanov	Ch suffr	end N-Aegean
*Morina persica* L.	Ch suffr	medit-irano-turan
*Muscari botryoides* (L.) Miller	G bulb	euro-medit
*Muscari kerneri* Marsh.	G bulb	euro-medit
*Muscari neglectum* Guss.	G bulb	circum-medit
*Myosotis incrassata* Guss.	T scap	circum-medit
*Myosotis suaveolens* Waldst. & Kit. ex Willd.	H scap	end Balkan
*Myosotis sylvatica* Hoffm. subsp. *cyanea* (Hayek) Vestergren	H scap	E-medit
*Neotinea maculata* (Desf.) Stern.	G bulb	circum-medit
*Nepeta argolica* Bory ex Chaub. subsp. *argolica*	Ch suffr	end CS Greece
*Nepeta camphorata* Boiss. & Heldr.	Ch suffr	end Peloponnese
*Nepeta dirphya* Boiss.	H scap	end Euboea
*Nepeta nuda* L. var. *epirotica* Halácsy	Ch suffr	end Sterea Ellas
*Nepeta parnassica* Heldr. & Sartr. ex Boiss.	Ch suffr	end CS Greece
*Nepeta spruneri* Boiss.	Ch suffr	end NC Greece
*Nigella arvensis* L. subsp. *glauca* (Boiss.) A. Terrac.	T scap	E-medit
*Noaea mucronata* (Forssk.) Aschers. & Schweinf.	Ch pulv	E-medit
*Noccaea graeca* (Jordan) Meyer	Ch caesp	end Peloponnese
*Nonea pulla* (L.) DC.	H scap	euro-medit
*Onobrychis ebenoides* Boiss. & Spruner	Ch suffr	end CS Greece
*Onobrychis laconica* Orph. ex Boiss.	Ch suffr	end Greece
*Onobrychis montana* DC. subsp. *macrocarpa* Strid	Ch suffr	end Greece
*Ononis pusilla* L.	H scap	euro-medit
*Ononis spinosa* L. subsp. *antiquorum* (L.) Arcang.	Ch caesp	european
*Ononis spinosa* L. subsp. *leiosperma* (Bois.) Sirj.	Ch suffr	euro-medit-irano-turan
*Onopordum illyricum* L.	H scap	circum-medit
*Onosma erecta* Sm. subsp. *malickyi* Teppner	H caesp	end Peloponnese
*Onosma heterophylla* Griseb.	Ch suffr	end Balkan
*Onosma leptantha* Heldr.	Ch suffr	end Peloponnese
*Origanum hirtum* Link	H scap	E-medit
*Origanum sypileum* L.	H caesp	E-medit
*Ornithogalum montanum* Ten.	G bulb	E-medit
*Ornithogalum nutans* L.	G bulb	E-medit
*Ornithogalum oligophyllum* E.D. Clarke	G bulb	E-medit
*Ornithogalum sibthorpii* Greuter	G bulb	E-medit
*Papaver dubium* L.	T scap	paleotemp
*Paracaryum aucheri* (A. DC.) Boiss.	Ch suffr	E-medit
*Paronychia albanica* Chaudri subsp. *graeca* Chaudri	Ch pulv	end CS Greece
*Paronychia bornmuelleri* Chaudhri	Ch rept	end N-Aegean
*Paronychia chionaea* Boiss. subsp. *chionaea*	H caesp	E-medit
*Paronychia euboaea* Beauverd & Topali	H caesp	end Euboea
*Paronychia macedonica* Chaudhri	H caesp	end Balkan
*Paronychia macrosepala* Boiss.	H caesp	E-medit
*Paronychia polygonifolia* (Vill.) DC.	H caesp	N-medit
*Pedicularis graeca* DC.	H scap	E-medit
*Petrorhagia armerioides* (Ser.) Ball & Heywood	Ch suffr	E-medit
*Petrorhagia fasciculata* (Margot & Reut.) Ball & Heywood var. *cephallenica* Damboldt & Phitos	Ch suffr	end Ionian islands
*Petrorhagia illyrica* (Ard.) Ball & Heywood subsp. *illyrica*	Ch suffr	end Balkan
*Petrorhagia illyrica* (Ard.) Ball & Heywood subsp. *taygetea* (Boiss.) Ball & Heywood	Ch suffr	end Greece
*Peucedanum longifolium* Waldst. & Kit.	H scap	E-medit
*Phitosia crocifolia* (Sibth. & Sm.) Kamari & Greuter	Ch suffr	end Peloponnese
*Phleum alpinum* L.	H caesp	euro-medit
*Phleum graecum* Boiss. & Heldr.	T scap	E-medit
*Phleum montanum* C. Koch	H caesp	paleotemp
*Phlomis fruticosa* L.	Ch frut	circum-medit
*Phlomis grandiflora* Thompson	Ch frut	E-medit
*Phlomis samia* L.	Ch suffr	end Balkan
*Picnomon acarna* (L.) Cass.	T scap	euro-medit-irano-turan
*Picris pauciflora* Willd.	T scap	E-medit
*Pilosella cimosa* (L.) Schultz & Sch. Bip. subsp. *sabina* (Sebast.) Fuchs	H scap	eurasian
*Pilosella hoppeana* F. W. Schultz & Sch. Bip. subsp. *testimonialis* (Nag. ex Pet.) Sell & C.West	H ros	euro-medit
*Pilosella leucopsilon* (Arv.-Touv.) Gottschl. subsp. *pilisquama* (Nageli & Peter) Gottschl.	H ros	euro-medit
*Pilosella officinarum* Vaill.	H ros	eurosiberian
*Pimpinella peregrina* L.	H scap	euro-medit
*Pimpinella polyclada* Boiss. & Heldr.	H scap	E-medit
*Pimpinella tragium* Vill. subsp. *tragium*	Ch suffr	N-medit
*Plantago atrata* Hoppe subsp. *graeca* (Halácsy) Holub	H scap	end Greece
*Plantago holosteum* Scop. var. *alpestris* (Griseb.) Rech. f.	Ch pulv	end CS Greece
*Plantago lanceolata* L.	H ros	circumboreal
*Poa bulbosa* L.	H caesp	paleotemp
*Poa sylvicola* Guss.	H caesp	medit-irano-turan
*Poa thessala* Boiss. & Orph.	H caesp	end Balkan
*Poa timoleontis* Heldr. ex Boiss.	H caesp	E-medit
*Poa trichophylla* Heldr. & Sart.	H caesp	end Sterea Ellas
*Podospermum canum* C.A. Meyer var. *alpinum* Boiss.	H scap	end Greece
*Polygala nicaeensis* Koch subsp. *mediterranea* Chodat	H scap	circum-medit
*Polygala nicaeensis* Koch subsp. *tomentella* (Boiss.) Chodat	H scap	end Greece
*Potentilla recta* L.	H rept	eurasian
*Potentilla speciosa* Willd.	Ch suffr	E-medit
*Prunella vulgaris* L.	H rept	eurasian
*Prunus cocomilia* Ten.	NP	E-medit
*Pteridium aquilinum* (L.) Kuhn	G rhiz	cosmop
*Pterocephalus perennis* Coult. subsp. *perennis*	Ch pulv	end CS Greece
*Pterocephalus pinardii* Boiss.	Ch pulv	E-medit
*Ptilostemon afer* (Jacq.) Greuter	H bienn	end Balkan
*Ptilotrichium cyclocarpum* Boiss. subsp. *cyclocarpum*	Ch suffr	E-medit
*Quercus calliprinos* Webb	P	E-medit
*Ranunculus brevifolius* Ten.	G rhiz	E-medit
*Ranunculus ficarioides* Bory & Chaub.	G rhiz	E-medit
*Ranunculus psilostachys* Griseb.	H ros	end Balkan
*Ranunculus rumelicus* Griseb.	G rhiz	E-medit
*Ranunculus sartorianus* Boiss. & Heldr.	G rhiz	E-medit
*Rhamnus oleoides* L. subsp. *graecus* (Boiss. & Reut.) Holmboe	NP	E-medit
*Rhamnus saxatilis* Jacq. subsp. *prunifolia* (Sm.) Aldén	NP	european
*Rhinanthus pubescens* (Sterneck) Boiss. & Heldr. ex Soó	T scap	end NC Greece
*Ribes uva-crispa* L.	NP	euro-medit-irano-turan
*Rindera graeca* (A. DC.) Boiss. & Heldr.	Ch suffr	end Greece
*Rosa agrestis* Savi	NP	european
*Rumex acetosella* L. subsp. *acetoselloides* (Balansa) den Nijs	H scap	E-medit
*Rumex nebroides* Campd.	H scap	circum-medit
*Rumex pulcher* L.	H scap	circum-medit
*Salvia argentea* L. var. *alpina* Heldr.	H scap	end Greece
*Salvia fruticosa* Mill.	NP	E-medit
*Sanguisorba minor* Scop. subsp. *verrucosa* (G. Don) Cout.	H scap	cosmop
*Saponaria bellidifolia* Sm.	H rept	N-medit
*Saponaria calabrica* Guss.	T scap	circum-medit
*Sarcopoterium spinosum* (L.) Spach	Ch pulv	E-medit
*Satureja cuneifolia* Ten.	Ch suffr	E-medit
*Satureja montana* L. subsp. *macedonica* (Formanek) Baden	Ch suffr	end NC Greece
*Satureja parnassica* Heldr. et Sart.	Ch suffr	end CS Greece
*Satureja spinosa* L. var. *glabra* W. Barbey & Major	Ch pulv	E-medit
*Saxifraga adscendens* L.	H bienn	euro-medit
*Scabiosa columbaria* L.	H scap	eurasian
*Scabiosa ochroleuca* L.	H scap	eurosiberian
*Scabiosa taygetea* Boiss. & Heldr. subsp. *taygetea*	H caesp	end CS Greece
*Scabiosa webbiana* D. Don	H caesp	end Balkan
*Scandix australis* L.	T scap	circum-medit
*Scandix macroryncha* C. A. Meyer	T scap	N-medit
*Scilla nivalis* L.	G bulb	end Greece
*Scleranthus marginatus* Guss.	H caesp	E-medit
*Scorzonera mollis* Bieb.	G rhiz	medit-irano-turan
*Scutellaria orientalis* L. subsp. *alpina* (Boiss.) Schwarz	Ch suffr	medit-irano-turan
*Scutellaria rupestris* Boiss. & Heldr. subsp. *rupestris*	H scap	end Peloponnese
*Scutellaria rupestris* Boiss. & Heldr. subsp. *cephalonica* (Rech. f.) Greuter & Burdet	H caesp	end Ionian islands
*Secale strictum* (Presl) Strobl	H caesp	circum-medit
*Sedum acre* L.	Ch succ	paleotemp
*Sedum album* L.	Ch succ	paleotemp
*Sedum dasyphyllum* L.	Ch succ	circum-medit
*Sedum hispanicum* L.	T scap	E-medit
*Sedum laconicum* Boiss. & Heldr.	Ch succ	E-medit
*Sedum magellense* Ten.	Ch succ	circum-medit
*Sedum ochroleuchum* Chaix	Ch succ	euro-medit
*Sedum tenuifolium* (Sm.) Strobl	Ch succ	circum-medit
*Sedum urvillei* DC.	Ch succ	E-medit
*Sempervivum marmoreum* Griseb.	Ch succ	euro-medit
*Senecio squalidus* L.	H scap	circum-medit
*Senecio thapsoides* DC.	H scap	end Balkan
*Senecio vernalis* Wladst. & Kit.	T scap	paleotemp
*Sesleria achtarovii* Deyl	H caesp	circum-medit
*Sesleria anatolica* Deyl	H caesp	E-medit
*Sesleria krajinae* Deyl	H caesp	end Euboea
*Sesleria tenerrima* (Frirsch) Hayek	H caesp	end Balkan
*Sesleria vaginalis* Boiss. & Orph.	H caesp	end Greece
*Sideritis clandestina* (Bory & Chaub.) Hayek subsp. *clandestina*	Ch suffr	end Peloponnese
*Sideritis clandestina* (Bory & Chaub.) Hayek subsp. *peloponnesiaca* (Boiss. & Heldr.) Baden	Ch suffr	end Peloponnese
*Sideritis euboea* Heldr.	Ch suffr	end Euboea
*Sideritis raeseri* Boiss. & Heldr. subsp. *raeseri*	Ch suffr	E-medit
*Sideritis raeseri* Boiss. & Heldr. subsp. *attica* (Heldr.) Papan. & Kokkini	Ch suffr	end Sterea Ellas
*Sideritis sipylea* Boiss.	Ch suffr	E-medit
*Silene auriculata* Sibth. & Sm.	Ch pulv	end CS Greece
*Silene bupleuroides* (L.) subsp. *staticifolia* (Sm.) Chowdhuri	Ch suffr	E-medit
*Silene conica* L.	T scap	eurasian
*Silene italica* (L.) Pers. subsp. *italica*	Ch caesp	euro-medit
*Silene italica* (L.) Pers. subsp. *peloponnesiaca* Greuter	Ch caesp	end CS Greece
*Silene lesbiaca* Cand.	Ch caesp	end E-Aegean
*Silene multicaulis* Guss. subsp. *multicaulis*	H scap	E-medit
*Silene parnassica* Boiss. & Spruner	Ch suffr	end Greece
*Silene radicosa* Boiss. & Heldr. subsp. *radicosa*	Ch pulv	end Balkan
*Silene roemeri* Friv. subsp. *macrocarpa* (Vandas) Greuter	H caesp	end Balkan
*Silene ungeri* Fenzl	T scap	E-medit
*Silene urvillei* Schott	Ch pulv	E-medit
*Silene vulgaris* (Moench) Garcke subsp. *prostrata* (Gaudin) Chater & Walters	H scap	end CS Greece
*Sorbus graeca* (Spach) Lodd. ex S. Schauer	NP	eurasian
*Stachys alopecurus* (L.) Bentham.	H scap	euro-medit
*Stachys cretica* L. subsp. *smyrnaea* Rech. fil.	H scap	E-medit
*Stachys heldreichii* Boiss.	H scap	end Balkan
*Stachys tymphaea* Hausskn.	H scap	E-medit
*Stenbergia colchiciflora* Waldst. & Kit	G bulb	N-medit
*Stipa endotricha* Martinovský	H caesp	end CS Greece
*Stipa holosericea* Trin.	H caesp	medit-irano-turan
*Taraxacum albomarginatum* Richards	H ros	end Greece
*Taraxacum bythinicum* DC.	H ros	E-medit
*Taraxacum cfr. graecum* Dahlst.	H ros	end Greece
*Taraxacum cylleneum* Furnkranz	H ros	end Peloponnese
*Taraxacum delphicum* Dahlst.	H ros	end Greece
*Taraxacum gracilens* Dahlst.	H ros	E-medit
*Taraxacum laevigatum* DC.	H ros	euro-medit
*Taraxacum minimum* (Guss.) N. Terracc.	H ros	circum-medit
*Telephium orientale* Boiss.	Ch suffr	medit-irano-turan
*Teucrium capitatum* L.	Ch suffr	circum-medit
*Teucrium chamaedrys* L.	Ch suffr	euro-medit
*Teucrium divaricatum* Heldr.	Ch frut	E-medit
*Teucrium montanum* L. var. *parnassicum* Čelak.	Ch suffr	end Greece
*Thesium arvense* Horvatovszky	Ch rept	E-medit
*Thesium bergeri* Zucc.	Ch rept	E-medit
*Thesium parnassi* A. DC.	Ch rept	E-medit
*Thlaspi ochroleuchum* Boiss. & Heldr.	Ch suffr	E-medit
*Thlaspi perfoliatum* L.	T scap	paleotemp
*Thymbra capitata* (L.) Cav.	Ch frut	circum-medit
*Thymbra spicata* L.	T scap	E-medit
*Thymus chaubardii* (Boiss. & Heldr.) Celak	Ch suffr	E-medit
*Thymus holosericeus* Celak.	Ch suffr	end Ionian islands
*Thymus leucospermus* Haltvig	Ch suffr	end NC Greece
*Thymus leucotrichus* Halácsy	Ch suffr	E-medit
*Thymus parnassicus* Halácsy	Ch suffr	end Sterea Ellas
*Thymus samius* Ronniger & Rech. fil.	Ch suffr	end E-Aegean
*Thymus sibthorpii* Benth.	Ch suffr	E-medit
*Thymus sipyleus* Boiss.	Ch suffr	E-medit
*Thymus striatus* Vahl	Ch suffr	E-medit
*Thymus teucrioides* Boiss. & Spruner subsp. *teucrioides*	Ch suffr	end NC Greece
*Thymus zygioides* Griseb.	Ch suffr	end Balkan
*Tordylium hirtocarpum* Cand.	T scap	E-medit
*Tragopogon crocifolius* L. subsp. *samaritanii* (Boiss.) Richardson	H ros	E-medit
*Trifolium campestre* Schreb.	T scap	paleotemp
*Trifolium ottonis* Sprun.	H caesp	end CS Greece
*Trifolium parnassi* Boiss. & Spruner	Ch caesp	end Greece
*Trifolium physodes* Steven ex Bieb.	H scap	E-medit
*Trifolium pignantii* Fauché & Chaub.	G rhiz	end Balkan
*Trifolium pratense* L.	H scap	cosmop
*Trifolium repens* L.	H rept	cosmop
*Trifolium uniflorum* L.	H caesp	end Balkan
*Trinia frigida* (Boiss. & Heldr.) Drude	H scap	end CS Greece
*Trinia glauca* (L.) Dumort. subsp. *pindica* Hartvig	H scap	european
*Trinia guicciardii* (Boiss. & Heldr.) Drude	H scap	end CS Greece
*Tripodium graecum* (Boiss.) Lassen	Ch suffr	E-medit
*Trisetum tenuiforme* Jonsell	H caesp	E-medit
*Tulipa australis* Link	G bulb	euro-medit-irano-turan
*Urtica dioica* L.	H rhiz	cosmop
*Valantia aprica* (Sibth. & Sm.) Boiss. & Heldr.	H scap	E-medit
*Valeriana bertiscea* Pancic	H scap	end Sterea Ellas
*Valeriana dioscoridis* Sm.	H rhiz	E-medit
*Valeriana tuberosa* L.	H scap	paleotemp
*Verbascum acaule* (Bory & Chaub.) Kuntze	H ros	end Peloponnese
*Verbascum cylleneum* (Boiss. & Heldr.) O. Kuntze	H scap	end Peloponnese
*Verbascum delphicum* Boiss. & Heldr.	H scap	end Greece
*Verbascum densiflorum* Bertol.	H bienn	euro-medit-irano-turan
*Verbascum epixanthinum* Boiss. & Heldr. var. *epixanthinum*	H scap	end Greece
*Verbascum graecum* Heldr. & Sartori	H scap	end Balkan
*Verbascum guicciardii* Heldr.	H bienn	end Balkan
*Verbascum megaphlomos* (Boiss. & Heldr.) Hal.	H bienn	euro-medit-irano-turan
*Verbascum parnassicum* Halácsy	H scap	end Sterea Ellas
*Verbascum pycnostachyum* Boiss. & Heldr.	H bienn	E-medit
*Veronica arvensis* L.	T scap	paleotemp
*Veronica austriaca* L. subsp. *teucrioides* (Boiss. & Heldr.) Hal.	H scap	end NC Greece
*Veronica chamaedrys* L.	H scap	euro-medit
*Veronica erinoides* Boiss.& Spruner	Ch suffr	end CS Greece
*Veronica jacquinii* Baumg.	H scap	E-medit
*Veronica sartoriana* Boiss. & Heldr.	T scap	end Greece
*Veronica thymifolia* Sibth. & Sm.	Ch suffr	E-medit
*Veronica verna* L.	T scap	euro-medit-irano-turan
*Vicia cracca* L.	H rept	circumboreal
*Vincetoxicum hirundinaria* Medicus subsp. *nivale* (Boiss. & Heldr.) Markgraf	H scap	E-medit
*Vincetoxicun canescens* (Willd.) Decne subsp. *peduncolatum* Browicz	H scap	E-medit
*Viola cephalonica* Bornm.	H rept	end Ionian islands
*Viola chelmea* Boiss. & Heldr.	H rept	end CS Greece
*Viola euboaea* (Halácsy) Halácsy	H rept	end Euboea
*Viola graeca* (Becker) Halácsy	H rept	end Greece
*Viola mercurii* Halácsy	H rept	end CS Greece
*Viola parnonia* Tan, Sfikas & Vold	H scap	end Peloponnese
*Viola parvula* Tin.	T scap	circum-medit
*Viola sfikasiana* Erben	H scap	end Peloponnese
*Viola sieheana* Becker	H caesp	E-medit
*Viola stojanowii* W. Becker	H scap	end NC Greece
